# Enhancing platinum-based chemotherapy efficacy and safety through combination therapy-mediated remodeling of autophagic homeostasis in gastric cancer

**DOI:** 10.1038/s41419-026-08703-3

**Published:** 2026-04-22

**Authors:** Guangzhao Pan, Qianqian Xu, Kui Zhang, Xin Hu, Chongyang Li, Changhong Li, Haoyan Ji, Xiaosong Hu, Shaomin Shi, Renjian Hu, Chaowei Deng, Erhu Zhao, Jinfei Chen, Hongjuan Cui

**Affiliations:** 1 State Key Laboratory of Resource Insects, Medical Research Institute, Southwest University, Chongqing, China; 2 Center for Innovative Drug Research, Hangzhou Institute of Medicine (HIM), Chinese Academy of Sciences, Hangzhou, China; 3 Jinfeng Laboratory, Chongqing, China; 4 Chongqing Engineering and Technology Research Center for Silk Biomaterials and Regenerative Medicine, Chongqing, China; 5 School of Basic Medical Sciences, Zhejiang Chinese Medical University, Hangzhou, China; 6 Department of Oncology, The First Affiliated Hospital of Wenzhou Medical University, Wenzhou, China; 7 Collaborative Innovation Center for Cancer Personalized Medicine, Nanjing Medical University, Nanjing, China

**Keywords:** Chemotherapy, Macroautophagy

## Abstract

Platinum-based drugs exhibit potent anti-tumor efficacy but are limited by low bioavailability, severe toxicity, and resistance. Current therapeutic strategies lack effective solutions due to the unclear molecular mechanisms. Autophagy, with its dual protective/destructive roles, offers potential to enhance platinum-based chemotherapy, yet its clinical translation for optimizing therapeutic outcomes remains underexplored. To address this challenge, we screened a Natural Compounds Library (NCL) to identify low-toxicity agents that synergize with Cisplatin (Cis). Among 285 autophagy-related candidates, Catharanthine (CA) emerged as a specific autophagy activator model molecule that reduced toxicity and synergistically suppressed gastric cancer (GC) when combined with Cis. Mechanistically, CA promoted organ protection via endoplasmic reticulum stress (ERS)/AMPKα-dependent autophagy activation. The CA-Cis combination induced tumor-suppressive effects, including ERS, autophagosome accumulation, and cytoskeletal impairment in cancer cells. Conversely, CA-mediated autophagy protected normal cells, as AMPKα knockdown abolished this protection, resulting in DNA damage and apoptosis. These results highlight the dual autophagic flux regulation: tumor cells undergo destructive autophagy, while normal cells experience protective autophagy, establishing a favorable therapeutic balance. We confirmed that the CA-Cis combination activates autophagy through the AMPKα-ULK1 pathway in both tumor and non-tumor tissues and differentially regulates phosphorylation at serine 757 of ULK1, this differentiation can dramatically modulate autophagy activity, thereby mediating context-dependent dual outcomes of autophagic protection and detrimental effects. These findings elucidate a mechanism whereby CA enhances platinum efficacy by remodeling of autophagic homeostasis in organisms, providing a theoretical basis for optimizing platinum-based regimens. Our study bridges autophagy’s dual functionality with clinical strategy, proposing the combination of specific autophagy activators as a promising approach to overcome platinum resistance and toxicity in GC treatment.

## Introduction

Gastric cancer (GC) ranks as the fifth most frequently diagnosed cancer, accounting for 4.9% of all cancer cases and 6.8% of cancer mortalities (ranking fifth in mortality) [1, 2]. Despite recent advances in GC management, including surgery combined with radiotherapy and chemotherapy, the prognosis for advanced GC patients remains poor. Chemotherapy is an effective and commonly used GC treatment method involving in the use of one or more chemotherapeutic or alkylating agents [3]. However, the lack of effective, low-toxicity, and specific anti-GC agents is a problem that needs to be addressed urgently.

Cisplatin (Cis) is an effective, metal-based chemotherapeutic drug used for treating various solid tumors [4–6]. However, its clinical use is limited by its severe, dose-dependent toxic side effects. There are approximately 40 different toxic effects of Cis, including ototoxicity, neurotoxicity, gastrointestinal toxicity, hematological toxicity, cardiotoxicity, and hepatotoxicity. The most common of these is nephrotoxicity [7–10]. Cis-induced proximal tubular cell injury involves oxidative stress, inflammation, mitochondrial dysfunction, MAPK signaling activation, and DNA damage [11–13]. Recently, autophagy has emerged as a central stress-adaptive process in Cis toxicity. It acts predominantly as a cytoprotective mechanism in normal tissues and as a therapeutic target to improve efficacy [14–16]. Targeting AMPK or mTOR to enhance autophagy has been shown to alleviate Cis-induced organ injury, highlighting autophagy modulation as a promising strategy to mitigate drug toxicity [16].

Autophagy is a highly dynamic, multi-step process responsible for degrading and recycling intracellular components. It is best described by the concept of autophagic flux, encompassing the formation, maturation, and fusion of autophagosomes with lysosomes, as well as subsequent degradation [17, 18]. Blocking any step of this process disrupts flux and profoundly affects cell fate [19, 20]. Autophagy can function as a survival mechanism by maintaining metabolic homeostasis, or as a cell death mechanism when excessively activated or dysregulated [21, 22]. In cancer cells, autophagy often supports tumor survival under therapeutic stress. Consequently, most prior combination strategies have focused on autophagy inhibition, such as chloroquine and its derivatives, to enhance chemotherapy efficacy [23, 24]. However, autophagy inhibition represents only one side of autophagy biology. Emerging evidence suggests that excessive autophagy activation or sustained autophagic flux blockade can drive tumor cell death itself [24]. Based on this concept, we propose the following scientific inquiry: Can combined therapeutic strategies remodel autophagic status in chemotherapy patients to improve treatment outcomes by leveraging the dual biological functions of autophagy, thereby simultaneously improving physiological conditions and enhancing the efficacy of chemotherapy? Exploring the optimal activation of autophagy in drug combinations that mitigate Cis’s toxicity to normal cells while enhancing its efficacy against tumor cells is crucial.

We screened a Natural Compounds Library (NCL) to identify autophagy modulators with low-toxicity that could improve the therapeutic index of Cis. Catharanthine was identified as a model compound that activates ERS-AMPKα-ULK1-dependent autophagy in both cancerous and normal cells. Interestingly, under identical treatment conditions, catharanthine induces destructive autophagy with flux blockade in GC cells while maintaining protective, functional autophagic flux in normal cells. This simultaneously enhances antitumor efficacy and reduces systemic toxicity. These findings propose a novel therapeutic strategy for GC patients, offering critical insights into optimizing drug efficacy and safety profiles in chemotherapy.

## Materials and methods

### Cell culture

The human GC cell lines NUGC4 (RRID: CVCL_3082), MKN1 (RRID: CVCL_1415), HGC-27 (RRID:CVCL_1279) and MKN-45 (RRID: CVCL_0434), human gastric mucosal epithelial cells GES-1 (RRID:CVCL_EQ22), and human embryonic renal cell line 293 FT (RRID:CVCL_6911) were purchased from Cobioer Biosciences Co., Ltd. (Nanjing, China); Human kidney 2 (HK-2) cell line (RRID:CVCL_0302) was purchased from the American Type Culture Collection (ATCC, USA) and stored in our laboratory. The NUGC4, MKN1 and MKN-45 cells were cultured in the RPMI Medium 1640 basic (1×) (Gibco, USA) containing 10% fetal bovine serum (FBS, Gibco) with 1% penicillin-streptomycin (P/S; Invitrogen, USA). The HK-2 cells were cultured in Dulbecco’s Modified Eagle Medium (DMEM; Gibco, USA) supplemented with 10% FBS, and 1% P/S. The 293FT cells were cultured in DMEM with 10% FBS, 1% P/S, and1% G418 (Invitrogen, USA), to this, 2% glutamine (Invitrogen, USA), 1% non-essential amino acids (Invitrogen, USA), and 1% sodium pyruvate (Invitrogen, USA) were added. The MKN-45 drug-resistant (MKN-45 DR) cell line was cultured in a complete 1640 medium that contained 30 μM Cis. All cells were standardized in a humidified incubator (ThermoFisher SCIENTIFIC, USA) at 37 °C with 5% CO_2_.

### Transfection and infection

The shRNA primers for human PERK, AMPKα and ULK1 were designed using the GPP Web Portal (https://portals.broadinstitute.org/gpp/public/). After primer annealing, the target sequences were cloned into the pLKO.1 vector, and then the correct target sequences were obtained from a sequencing company (Beijing Genomics Institute). Lentiviral production, infection, and the establishment of stable PERK- and ULK1-knockdown cell lines were performed as described in a previous study [25]. The mRFP-eGFP-LC3B and GPF-LC3B adenoviruses were purchased from HanBio Therapeutics (Shanghai, China). One day before infection, 15,000 cells were plated in 24-well plates and incubated at 37 °C overnight. Then, 500 μL of the 1640 culture medium containing 0.5 μL of adenovirus was added to each well. The cells were incubated at 37 °C for 24 h, and the culture medium was replaced with a fresh medium. The cells were observed using a fluorescence microscope.

### Specific autophagy activators screening

All autophagy activators were screened from theNCL, an in-house curated collection of approximately 1437 monomers (Supplementary Information Table 4). Preliminary screening processes involved evaluating monomer solubility, stability, and toxicity. Non-compliant candidates were then excluded. The solubility of library candidate drugs was preliminarily queried mainly through MedChemExpress (MCE, https://www.medchemexpress.cn/) and Selleck.cn (https://www.selleck.cn/). We eliminated drugs with solubility <0.1 mg/mL, which are poorly soluble or insoluble. We made a preliminary judgment on the stability of candidate molecules based on their chemical structures. Finally, we conducted a query and screening by combining the Chemical Substance Toxicity Database (https://www.drugfuture.com/toxic/), the Acute Toxicity Database (http://www.cerc.usgs.gov/data/acute/acute.html), and the Compound Toxicity Database of the US National Library of Medicine (https://pubchem.ncbi.nlm.nih.gov/). Subsequent steps utilized the eGFP-mRFP-LC3B plasmid system, with cells seeded in 96-well plates for drug administration. Autophagy activators were further screened based on LC3B levels. In this process, LC3B protein expression levels were automatically recorded via high-content screening (MD ImageXpress Micro Confocal). A minimum of four fields of view per well were imaged for LC3B fluorescence quantification, and the results were ranked to clarify the intensity of autophagy activation. A preliminary assessment of autophagic flux blockade can be performed by calculating the ratio of green-labeled GFP-LC3B to red-labeled mRFP-LC3B. A ratio of GFP-/mRFP-LC3B less than 1 indicates active autophagic flux, while a ratio of 1 or greater demonstrates inhibited flux. Primary autophagy activators identified through screening were validated by direct visualization via confocal microscopy. We assessed the inhibitory activity and combination effects of the selected activators using CCK-8 assay and the Jin’s modified Bürgi’s formula. We further evaluated the synergistic effects using the Chou-Talalay method. The compounds demonstrating the highest potential underwent validation in animal models to confirm therapeutic efficacy and safety profiles.

### Drug response testing

Briefly, the cell viability was investigated by the MTT assay as previously described [25]. The combined effect of the indicated drugs was evaluated through Jin’s modified Bürgi’s formula (Details were shown as follows).

$$\begin{array}{l}{\rm{q}}=\frac{{\rm{Q}}({\rm{a}}+{\rm{b}})}{\mathrm{Qa}+\mathrm{Qb}-\mathrm{QaxQb}}\\ {\rm{q}}.\,\mathrm{Combined\; effects}\\ \begin{array}{l}{\rm{Q}}\left({\rm{a}}+{\rm{b}}\right).\,\mathrm{Effectiveness\; of\; two\; drugs\; when\; in\; combination}\\ \mathrm{Qa\; and\; Qb}.\,\mathrm{Effects\; of\; single\; drugs\; a\; or\; b\; when\; used}\end{array}\end{array}$$

The *q* > 1.15 indicates a synergistic effect, 0.85 < *q* < 1.15 indicates an additive effect, *q* < 0.85 indicates an antagonistic effect of the combination of the two drugs. We used Jin’s modified version of Bürgi’s formula to initially assess the combined effect of the two drugs. Then, the Combination Indices (CI) was used to further confirm the combined effects. The CompuSyn software was applied to calculated the combination indices [26].

### RNA sequencing, data analysis and RT-qPCR

After treatment with CAS (Catharanthine sulfate, CAS) or DMSO at 37 °C for 48 h, the cells were harvested and washed with 1× PBS buffer thrice, and total mRNA was isolated from the MKN-45 cells using TRIzol reagent according to the manufacturer’s instructions. Partial RNA samples were sent to Novogene (Beijing, China) for transcriptome sequencing. All transcriptome data were acquired from Novogene Company after sequencing and analysis. The analytical methods are based on our previous study [27, 28]. The remaining samples were reverse transcribed into cDNA using the GoScript^TM^ Reverse Transcription System following the instructions in the operation manual (A5001) (Promega). qPCR was performed as described in the instructions for the GoTaq® qPCR Master Mix (Promega) (A6002). The relevant primer sequences are presented in Supplementary Information Table 1.

### Flow cytometry

For the cell cycle assay, the cells were treated with DMSO or CAS at 37 °C for 48 h. Then, the cells were harvested, washed three times with 1× PBS, and fixed in ice-cold 75% ethanol at 4 °C for 24 h. After washing and resuspension in 1× PBS, the cells were incubated with PI and RNase A (Sigma Aldrich, USA) at 37 °C for 45 min. Finally, the samples were analyzed using a FACS C6 (BD, USA) with FlowJo software (Three Star, Ashland, OR, USA). For the cell apoptosis assay, the cells were harvested and resuspended in 1× binding buffer. Then, they were incubated with FITC-labeled Annexin V (BD, San Jose, CA, USA) according to the manufacturer’s instructions. The samples were detected by FACS C6, and the results were analyzed using the FlowJo software.

### Western blot analysis

MKN-45 and other reconstructive cell lines were treated with CAS for the indicated time or dose. The cells were harvested, washed three times with ice-cold PBS buffer, and suspended in RIPA lysis buffer (Beyotime, Shanghai, China) with phosphatase inhibitors (Sigma Aldrich, St. Louis, MO, USA) and a complete protease inhibitor cocktail (Roche) according to the manufacturer’s instructions. Protein concentrations were measured using a BCA protein assay kit (Beyotime, Shanghai, China). Cell lysates (50 μg) were subjected to 10% or 12% SDS-PAGE according to the proteins’ molecular weight and transferred onto PVDF membranes (Millipore, USA). The membranes were then blocked with 5% skim milk powder (Cat. No. 1172GR100) (BioFROxx, Germany) in 1× TBST buffer at room temperature for 2 h. Next, the PVDF membranes were incubated with primary antibodies (Supplementary Information Table 2) against human detection proteins at 4 °C overnight. After washing three times with 1× TBST buffer, the membranes were incubated with secondary antibodies (horseradish peroxidase (HRP)-labeled goat anti-mouse IgG (H + L) or goat anti-rabbit IgG (H + L)) at room temperature for 2 h. Finally, the results were analyzed using Supersignal West Femto Maximum Sensitivity Substrate (Thermo Fisher Scientific, Waltham, MA, USA), and luminescence images were collected using a Western blot detection instrument (Clinx Science, Shanghai, China).

For tumor tissue analysis, the samples were collected and stored in liquid nitrogen for 15 min. After the tissues solidified, the samples were transferred to a mortar and ground rapidly. The tumor tissue samples were then treated and analyzed as described above.

### Animal studies

#### Subcutaneous xenograft model (CDX)

All animal procedures were conducted in accordance with the Committee for the *Animal Protection and Utilization of Southwest University* and the Guidelines for *Animal Health and Use* (Ministry of Science and Technology, China, 2006). Five-week-old NOD/SCID mice (Huafukang Biotech) underwent a 1-week acclimatization period in SPF facilities before receiving a subcutaneous injection of 2 × 10^6^ GC cells suspended in 100 μL of serum-free RPMI-1640 medium. After injection, the animals were maintained in controlled SPF conditions until tumor establishment. Then, they received intraperitoneal administration of Cis (4 mg/kg), CAS monotherapy (25 mg/kg in saline solution), or combination therapies (25 mg/kg CAS + 4 mg/kg Cis) every 48 h. Tumor volumes were monitored every 2 days using standardized measurement protocols. Terminal cervical dislocation was performed after seven treatment cycles. Carcasses underwent cryopreservation at −20 °C before professional incineration through Laibite Biotech, while excised tumors were preserved for subsequent analysis.

#### Subcutaneous xenograft model (PDX)

With permission from the patients and *the Medical Ethics Committee of Chongqing Ninth People’s Hospital*, GC patient tumors were acquired from the Ninth People’s Hospital of Chongqing (the Affiliated Hospital of Southwest University, Beibei, China). GC patient tumors were acquired (See Supplementary Information Table 3 for patient information) and cut into approximately 2 mm × 2 mm × 2 mm pieces. Then, the tumors were implanted into both flanks of each mouse, and the wounds were sutured with absorbable medical sutures. Once the tumors reached a certain volume (approximately 100 mm³), intraperitoneal administration of Cis (4 mg/kg), CAS monotherapy (25 mg/kg in saline solution), and combination therapies (25 mg/kg CAS + 4 mg/kg Cis) were administered every 48 h. Tumor volumes were monitored every 2 days using standardized measurement protocols. Terminal cervical dislocation was performed after the ninth treatment cycle. The mice were killed by cervical dislocation, and the bodies were frozen at −20 °C before being transfer to Laibite Biotech Inc. (Chongqing, China) for incineration. The tumors were collected and analyzed. The experimental design prioritized isoflurane’s rapid metabolic clearance to avoid pharmacological interference, maintained strict SPF environments to eliminate confounding variables‌, and aligned dosing intervals with pharmacokinetic optimization principles‌. Ethical compliance was ensured through pain-minimization protocols.

#### Orthotopic xenograft tumor model

Briefly, GC patient tumors were cut into approximately 2 mm × 2 mm × 2 mm pieces. Then, the stomach blood vessels of the mice were punctured with a syringe and the tumor was affixed to punctured area with bioglue. Ten (GAM-AD)/or 18th days (GAM-0125) after the operation, followed by intraperitoneal administration of Cis (4 mg/kg), CAS monotherapy (25 mg/kg in saline solution), and combination therapies (25 mg/kg CAS + 4 mg/kg Cis) every 48 h. After thirteen injections, the mice were killed by cervical dislocation. The bodies were then frozen at −20 °C before being transferred to Laibite Biotech Inc. (Chongqing, China) for incineration. The tumors and other tissues were collected and analyzed. For mice in the free survival assessment group, tumor-bearing mice were treated with the same dosing regimen as described above until the study was terminated at 85 days.

### Histology and tissue injury score assessment

The tissue samples were fixed in 4% paraformaldehyde in 1× PBS buffer at 4 °C for 2 days. The paraffin-embedded samples were sectioned at 2 μm, mounted on microscopic slides, and dried at 65 °C for 4 h in an oven. Then, the slides were treated with different concentrations of xylene to remove the paraffin. The samples were stained with hematoxylin and eosin (H&E). Photographs were taken using a digital microscope. To determine tissue injury, a semi-quantitative scoring method was used. Scores ranged from 0 to 4. Score 0 indicated an injured area of less than 10%. Scores 1, 2, 3, or 4 indicated injured areas covering 10–25%, 25–50%, 50–75%, and >75% of the field, respectively. At least ten randomly selected fields under were evaluated under a microscope at 400× magnification for each mouse, and an average score was calculated.

### Immunofluorescence (IF)

Briefly, cells were seeded onto glass cover slips in 24-well plates. After treatment, the cells were fixed with 4% paraformaldehyde in 1× PBS buffer at room temperature for 30 min. The slips were then washed three times with 1× PBS and incubated with 0.3% Triton X-100 in 1× PBS buffer for 15 min. The cells were blocked with 5% goat serum (Sigma-Aldrich, G9023) at room temperature for 2 h and then incubated with the indicated primary antibody overnight at 4 °C. The cells were washed with 1× PBS three times and subsequently incubated with the secondary antibody (Invitrogen, 35552 for DyLight 488-conjugated goat anti-rabbit IgG and 35511 for DyLight 594-conjugated goat anti-mouse IgG) at room temperature for 2 h. Finally, the nuclei were finally stained with DAPI for 20 min. The samples were placed under a laser confocal microscope for observation.

### Immunohistochemistry

The paraffin-embedded samples were sectioned at 2 μm, mounted on microscopic slides, and dried at 65 °C for 4 h in an oven. Then, the slides were infiltrated with xylene of varying concentrations to remove the paraffin. After dehydration, heat-induced epitope retrieval was performed in a 10 mM citrate buffer solution at pH 7.2 in a microwave. The sections were then incubated with the primary antibodies LC3B (1:200, Abcam, ab192890), Ki67 (1:200, BD Biosciences, 550609), GRP78 (1:300, CST, #3177), cleaved PARP (1:50, CST, #5625), and γ-H2A.X (1:500, CST, #9718) at 4 °C overnight. Visualization was performed using diaminobenzidine (ZSGB-BIO, ZLI-9033) as the substrate, and the slides were counterstained with Mayer’s hematoxylin (Beyotime, C0107). Photographs were obtained using a digital microscope.

### Transmission electronic microscopy

The cells were treated with CAS. Then, the samples were harvested and fixed with a glutaraldehyde solution at 4 °C. Two days later, all samples were sent to Biomisp Biological Technology Company (Wuhan, China; http://www.biomisp.com**)** for sectioning and photography.

### Statistical analysis

The GraphPad Prism 9.0 software (GraphPad Software, La Jolla, CA, USA) was used to perform statistical analyses. All experiments were conducted with three technical and three biological replicates. The results are presented as the mean ± standard deviation (SD). Differences between means were evaluated using unpaired Student’s *t*-tests. Differences between groups were considered to be statistically significant at *p < 0.05, **p < 0.01, ***p < 0.001.

### Ethics approval and consent to participate

All animal protocols were approved by the Committee for Animal Protection and Utilization of Southwest University (Approval no. IACUC-20240102-02, date: 29 April 2024). All human specimens utilized in this study were residual samples from clinical diagnostics and treatments. Patients provided written informed consent authorizing the use of their residual specimens for scientific research. All patient’ samples used in this study was approved by the Medical Ethics Committee of Chongqing Ninth People’s Hospital (Approval no. 2019 (Lun Shen)-015, date: 2 January 2019).

## Results

### Multiple conditional screens revealed several potential specific autophagy activators based on NCL

**Fig. 1:**
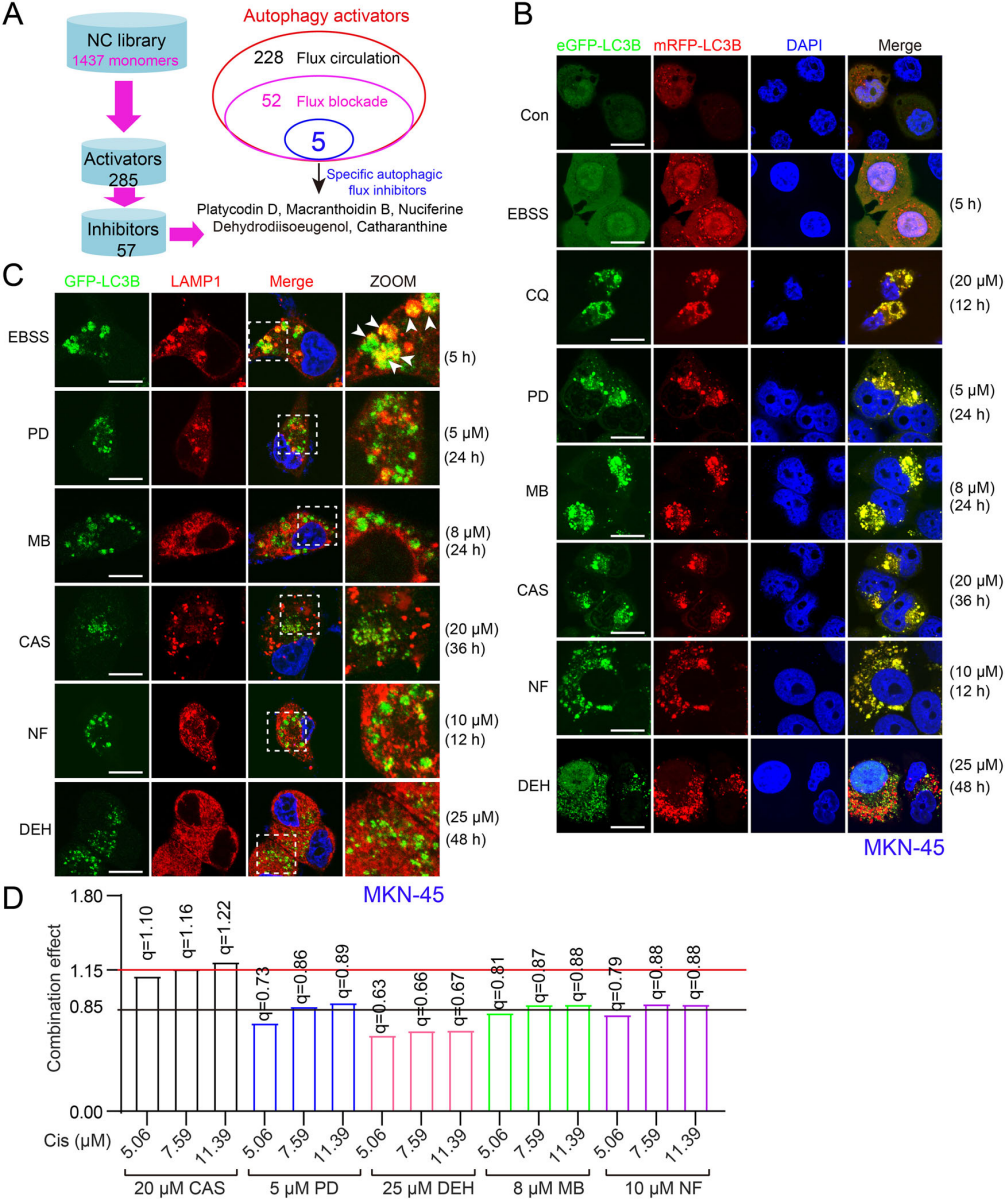
Multiple conditional screens identified potential autophagy activators based on NCL. **A** Schematic workflow and results of autophagy-specific activator screening. **B** Co-localization analysis of eGFP-LC3B and mRFP-LC3B puncta in MKN-45 cells treated with DMSO, EBSS, CQ (20 μM), PD (5 μM), MB (8 μM), CAS (20 μM), NF (10 μM), and DEH (25 μM) for indicated durations. Scale bar = 100 μm. **C** Co-localization analysis of GFP-LC3B and LAMP1 puncta in MKN-45 cells treated with EBSS, PD (5 μM), NF (10 μM), MB (8 μM), and DEH (25 μM) for indicated durations. The white arrows indicate colocalized target proteins. Scale bar = 100 μm. **D** The combination effect of the indicated drugs combined with Cis through Jin’s modified Bürgi’s formula. The *q* > 1.15 indicates a synergistic effect, 0.85 < *q* < 1.15 indicates an additive effect, *q* < 0.85 indicates an antagonistic effect of the combination of the two drugs.

Initial screening from our Natural Compounds Library (NCL) (Supplementary Information Table 4) used criteria such as autophagy activation, autophagic flux status, inhibitory effects, and individual toxicity to identify specific autophagic flux inhibitors (Fig. S1A). Approximately 285 autophagy activators were identified, including 57 candidate inhibitors, but most exhibited significant toxicity in combination therapies. Among these, platycodin D (PD) emerged as a potential candidate alongside catharanthine (CA), macranthoidin B (MB), nuciferine (NF), and dehydrodiisoeugenol (DEH) (Fig. 1A). Using the GFP-mRFP-LC3B reporter system [29], we found that cells treated with inhibitors showed elevated GFP/RFP-LC3B puncta compared to control starved of EBSS (under normal conditions, cells generate flowing autophagic flux within a limited time of starvation [30]). This indicates autophagosome accumulation (Fig. 1B). Co-localization analysis with LAMP1 (lysosomal inner membrane protein[31]) confirmed that these inhibitors blocked the fusion of autophagosomes and lysosomes, indicating blockade of autophagic flux (Fig. 1C). Synergy evaluation via Jin’s modified Bürgi’s formula revealed an additive effect for most inhibitors (0.85 < *q* < 1.15). DEH showed antagonism (*q* < 0.85). Notably, CA demonstrated concentration-dependent synergy with Cis (*q* > 1.15) [32] (Fig. 1D). CA is a precursor to vincristine (VCR), which is clinically relevant because both Cis and VCR are established antitumor agents [33] (Fig. S1B). However, the pharmacological interactions and toxicity profiles of CA-Cis combination therapy remain uncharacterized, underscoring the novelty of this mechanistic and therapeutic exploration.

We compared the pharmacological profiles of CA and VCR, using sulfate-modified CA (Catharanthine sulfate, CAS) for improved solubility (Fig. S1B). Cellular structural phenotype analysis revealed distinct effects of CAS and VCR on cell morphology: CAS induced smooth, crumpled cell membranes, while VCR produced characteristic tentacles derived from the cell membrane that resemble neural outgrowths (Fig. S1C). Functionally, CAS arrested the cell cycle at the G0/G1 phase without inducing obvious apoptosis (Fig. S1D, E), contrasting with VCR’s M-phase arrest and autophagic flux inhibition [34] (Fig. S1F). Cytoskeletal impacts diverged. When living cells were placed in a mixture of ice and water, CAS treatment remodeled microtubule organization centers without depolymerization. In contrast, VCR caused significant microtubule breakdown (Fig. S1G). VCR also rapidly accumulated autophagosomes, a phenomenon absent in CAS-treated cells because CAS takes longer to complete (Fig. S1F and Fig. 1B, C). These findings highlight fundamental mechanistic differences between the two agents. VCR acts as a classical microtubule-targeting inhibitor [35], while CAS exerts physiological regulation through distinct pathways. These results underscore CA’s unique therapeutic potential, diverging from conventional Vinca alkaloid mechanisms.

To determine whether CA directly affects lysosomal function, we first assessed lysosomal acidity using acridine orange (Ao) staining [15]. Fluorescence imaging revealed preserved red fluorescence in CA-treated cells, indicating intact acidic compartments (Fig. S1H). Next, we quantified lysosomal pH with the ratiometric probe LysoSensor Yellow/Blue DND‑160. This probe showed consistent pH values of approximately 4.2 under both control and CA-treated conditions (Fig. S1I). Finally, immunoblot analysis of cathepsins B, D, and L demonstrated that CA did not affect their expression or maturation (Fig. S1J). Collectively, these results indicate that CA does not impair lysosomal acidification or protease activity, but rather, it blocks autophagic flux by disrupting the fusion of autophagosomes and lysosomes.

### Identification of Cis combined with CAS synergistically inhibited GC in vitro and in vivo

**Fig. 2:**
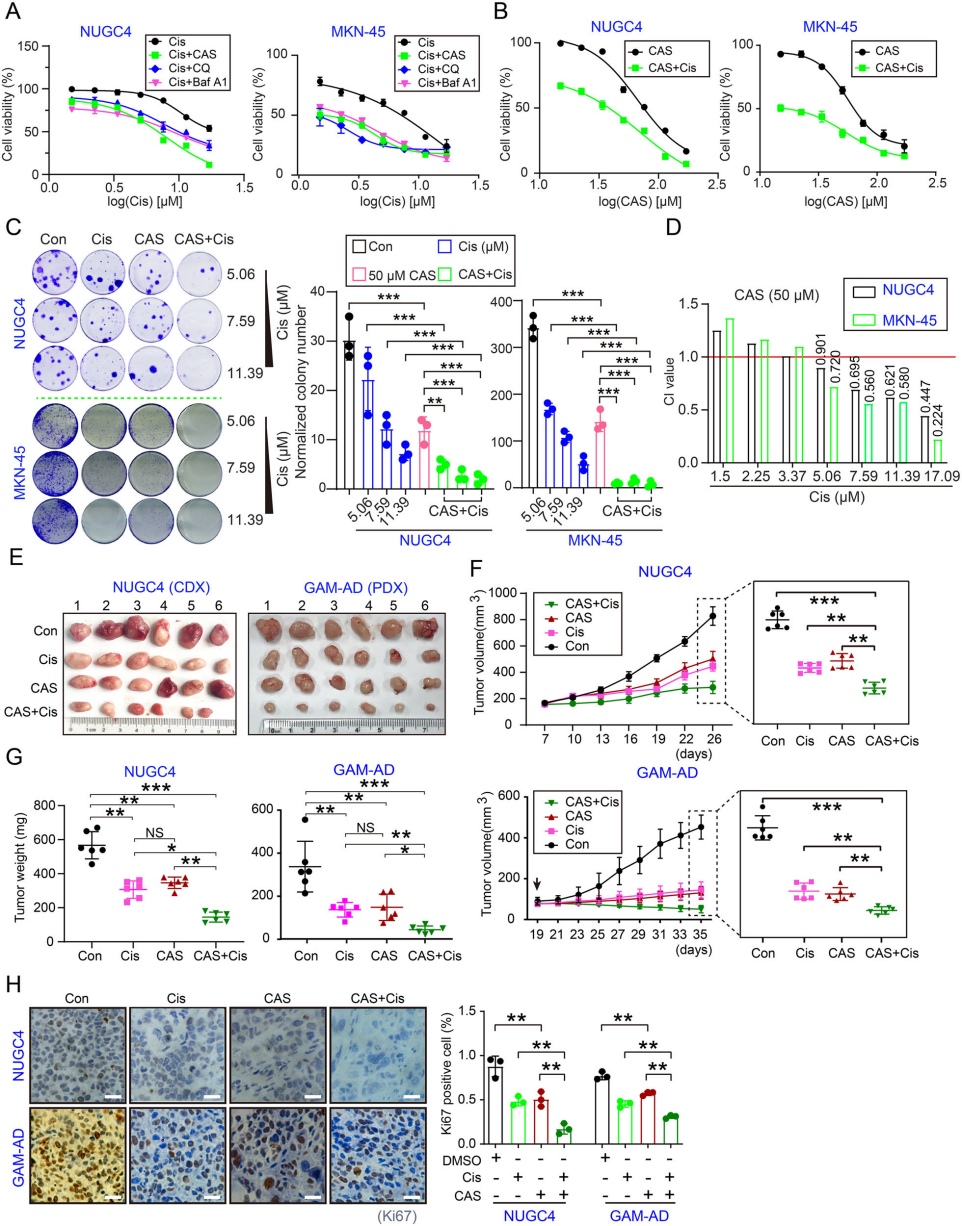
Synergistic suppression of GC cell proliferation and tumor growth by CAS combined with Cis. **A** GC cell lines (representative lines indicated) treated with varying concentrations of Cis and CQ (5 μM) or Baf A1 (300 nM) for 48 h. Cell proliferation was assessed by MTT assay (2 h incubation), with normalized absorbance measured at 490 nm. **B** GC cell lines (representative lines indicated) treated with CAS (15–170.85 μM) and a fixed Cis dose (7 μM) for 48 h. MTT assay results (normalized to DMSO controls) show absorbance at 490 nm. **C** Colony formation assay of GC cells treated with CAS (50 μM) and Cis (5.06–11.39 μM) alone or in combination. Crystal violet-stained colonies were quantified after 13 days; column chart shows mean colony numbers. **D** Combination index (CI) values calculated via CompuSyn software. GC cells were exposed to Cis (1.5–17.09 μM) and CAS (50 μM) for 48 h. **E** The pictures show tumor masses harvested at the experimental endpoint from CDX and PDX subcutaneous xenograft models treated with saline solution, 4 mg/kg Cis, 25 mg/kg CAS, and 4 mg/kg Cis plus 25 mg/kg CAS. Tumor volume (**F**) and weight (**G**) measurements at indicated time points. **H** Ki67 immunohistochemical staining in the tumors from (**E**). Scale bar = 100 μm. All data are shown as the mean ± standard deviation (SD) and are representative of three independent experiments. *P < 0.05, **P < 0.01, ***P < 0.001. All data represent three independent experiments. NS no significance.

We next systematically evaluated the anti-GC effects of CAS combined with Cis. Initial IC_50_ assays revealed greater drug resistance in normal gastric mucosal epithelial cells (GES-1) than in GC cell types (Fig. S1K). Since no significant cell death was observed in CAS-treated cell populations, we subsequently determined the EC_50_ values (half-maximal effective concentration) following pharmacological intervention in NUGC4 and MKN-45 cell lines (Fig. S1L). We attempted to investigate the combinatorial effects of different autophagy inhibitors (chloroquine [CQ], a late-stage autophagic flux blocker, and bafilomycin A1 [Baf A1], an inhibitor that disrupts autophagosome-lysosome fusion) with Cis. The results demonstrated that these autophagy inhibitors could enhance Cis chemotherapeutic efficacy to varying degrees (Fig. 2A). Notably, within certain concentration ranges of Cis or CAS, the CAS exhibited more favorable therapeutic outcomes (Fig. 2A–C). To investigate whether the combination of CAS and Cis exert synergistic inhibitory effects on GC, we evaluated their combination index (CI) using the Chou-Talalay method [36]. The results revealed synergistic effects across a broad concentration range of Cis (CI value < 1) (Fig. 2D).

We next examined the treatment effects of the combination treatment on cell line-derived xenograft (CDX) and patient-derived tumor xenograft (PDX) models. Subcutaneous xenograft experiments demonstrated significantly reduced tumor sizes (Fig. 2E), volumes (Fig. 2F), and weights (Fig. 2G) in co-treated mice compared to single-agent groups. Additionally, Ki67-positive rates in co-treated tumors were markedly lower than those in monotherapy cohorts (Fig. 2H).

The most challenging issue for patients undergoing long-term chemotherapy is the development of resistance to the treatment, which results in diminished efficacy [37]. We wondered whether this combination strategy could increase the sensitivity of the Cis-resistant model. Using a dose-interrupted pulse method, we established the MKN-45 DR (drug resistance) cell line (resistance index, RI = 5.01), which exhibits a certain level of resistance to Cis (Fig. S2A). To validate the robustness of the drug-resistant phenotype, we collected cells after multiple passages (cell generations labeled as G1, G2, G3, G6, and G9) and profiled key resistance markers across functional categories, including drug efflux transporters: ABCB1, and ABCC2; DNA repair machinery: RAD51, BRCA1 and anti-apoptotic regulators: Bcl-2 and Bax)[38–40]. Our results showed that all markers exhibited sustained overexpression or obvious downregulation (Bax), without significant variation between passages, confirming phenotypic stability (Fig. S2B). Notably, compared to monotherapy, the combination of CAS and Cis significantly inhibited the proliferation of resistant cells (Fig. S2C–E). Furthermore, synergistic inhibitory effects were maintained across a broad Cis concentration range (Fig. S2F). Taken together, the CAS-Cis combination synergistically suppresses gastric tumor growth and restores Cis sensitivity in low-level resistance models.

### The combinatorial strategy antagonized the toxicity of Cis in tumor-bearing mice

**Fig. 3:**
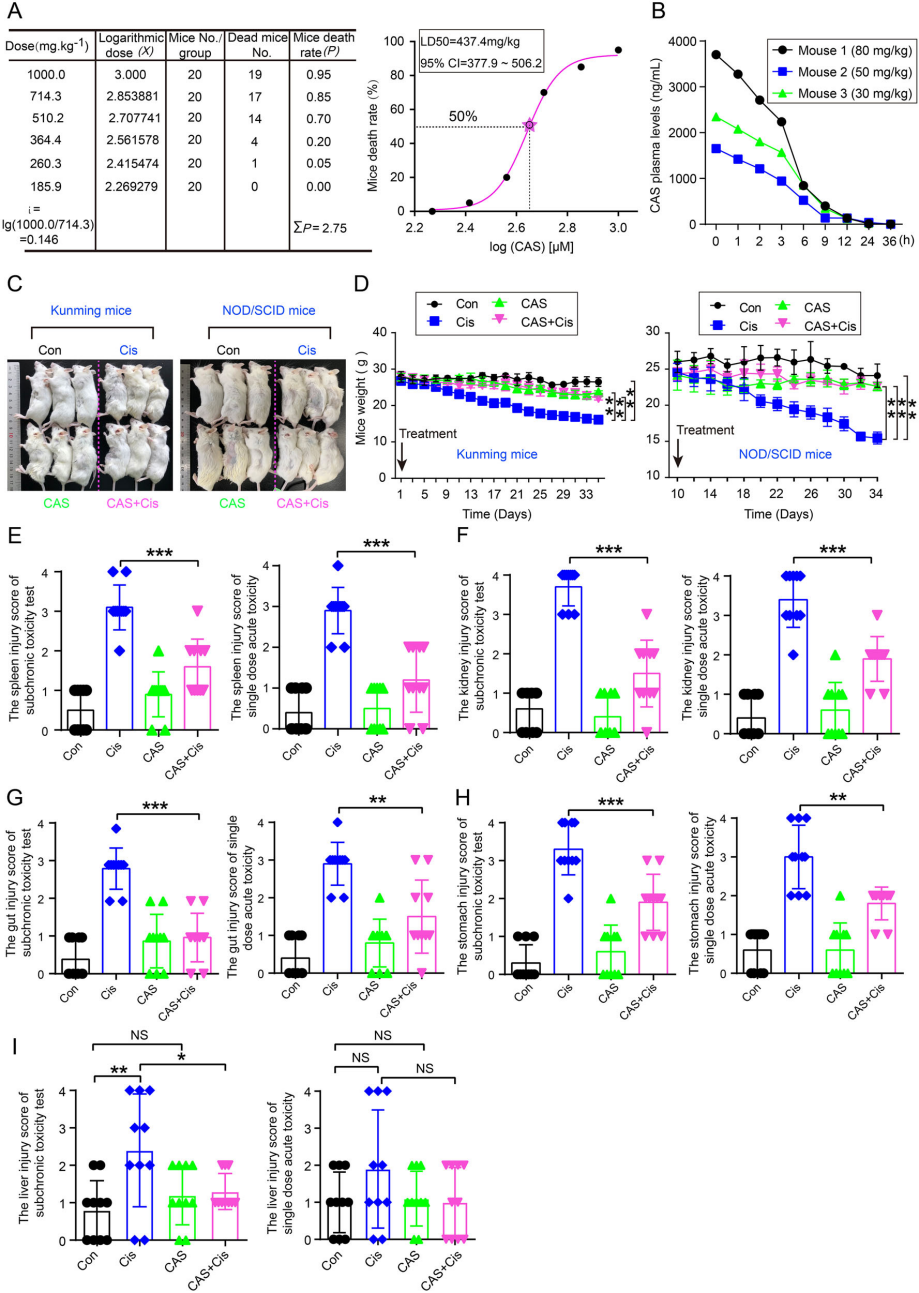
CAS combined with Cis mitigates Cis toxicity in mice. **A** LD_50_ determination in Kunming mice via Kärber assay, with mortality rates plotted as a dose-response curve. **B** Plasma concentration-time profiles of CAS in SD rats analyzed by HPLC (Tail vein injection of CAS (30, 50, 80 mg/kg). Blood sampling via retro-orbital bleeding). **C** Body morphology changes in mice were treated with saline solution, CAS (25 mg/kg), Cis (4 mg/kg), and Cis-CAS (4 mg/kg Cis+25 mg/kg CAS) combination during subchronic toxicity assessment. **D** Body weight dynamics under CAS/Cis monotherapy or combination therapy (time-course data). H&E-stained spleen (**E**), kidney (**F**), Gut (**G**), stomach (**H**) and liver (**I**) sections from Kunming mice treated with CAS or Cis alone or in combination in both subchronic and acute toxicity assessments. The graph shows the injury scores for organs damage within groups in the subchronic toxicity test and single-dose acute toxicity assessment. For the single dose toxicity, mice were treated with 150 mg/kg CAS, 12 mg/kg Cis, alone or in combination, the effects were observed within 24 h. All data are shown as the means ± SD. *P < 0.05, **P < 0.01, ***P < 0.001. NS no significance.

To determine whether the combination of CAS and Cis would induce minor or greater toxic side effects, we first determined the half lethal dose (LD50) of CAS as 437.4 mg/kg and the safe dose as >185.9 mg/kg through single-dose acute toxicity tests in Kunming mice (Fig. 3A). Pharmacokinetic analysis via high-performance liquid chromatography (HPLC) in SD rats revealed a plasma half-life of ~5 h for CAS, with complete metabolism occurring within 24 h post-injection (Fig. 3B). We also assessed the metabolic profile of CAS in mouse plasma using high-performance liquid chromatography-mass spectrometry (HPLC-MS) to further elucidate the pharmacokinetic mechanisms underlying its anti-tumor effects. The MS data (Supplementary Table) demonstrate that CAS undergoes extensive metabolism, generating hundreds of metabolites (after excluding background plasma components). Notably, although CA serves as a synthetic precursor, no metabolites that are structurally analogous to or identical with VCR (C₄₆H₅₈N₄O₉) have been detected in our studies. These findings are consistent with earlier research on the functional divergence of two compounds (Fig. S1C–G).

To establish an optimal dosing schedule for combination therapy, we first confirmed an adequate therapeutic window that would maintain mouse viability and physiological function during repeated treatments. Using an orthotopic xenograft model with 48 h dosing intervals, liquid chromatography-tandem mass spectrometry (LC-MS/MS) analysis demonstrated sustained, quantifiable concentrations of CAS in both tumor and kidney tissues throughout this period. This finding supports the feasibility of this regimen (Fig. S3A). Notably, co-treated mice exhibited superior body weight maintenance and vitality compared to Cis monotherapy in tumor-bearing models (Fig. 3C, D). To validate these observations, we conducted parallel toxicological assessments were conducted in both immunocompetent (Kunming) and immunodeficient (NOD/SCID) mice. Subchronic toxicity testing demonstrated the following: (1) improved physiological profiles in co-treated groups via body contour analysis, (2) reduced weight fluctuation with combination therapy compared to Cis alone (Fig. 3C, D). These findings collectively indicate that the CAS-Cis regimen maintains favorable safety parameters while mitigating chemotherapy-associated physiological deterioration. The metabolic profile and toxicity data provide critical pharmacodynamic references for clinical translation.

We next analyzed the pathological features of different organs and tissues in mice. A pathological analysis of multiple organs (heart, liver, spleen, lungs, kidneys, brain, muscles, and gut) revealed different toxicity profiles (Fig. S3B–F). Cis monotherapy had mild effects on the heart, lungs, brain, and muscles in acute and chronic tests (Fig. S3B, C). However, severe, organ-specific damage emerged, including liver injury (Fig. S3D), spleen injury (Fig. 3E and Fig. S3E, F), acute kidney injury (AKI) (Fig. 3F and Fig. S3E, F), and gastrointestinal lesions (Fig. 3G, H and Fig. S3E, F). Interestingly, the combination therapy attenuated these pathological alterations (Fig. 3E–H and Fig. S3B–F). Quantitative analysis revealed 58.6% reduction in spleen injury (single-dose acute toxicity tests) and a 48.6% reduction (subchronic toxicity tests) (Fig. 3E). Additionally, there was a 44.1% and 54.1% decrease in kidney injury, respectively, across toxicity tests (Fig. 3F), Furthermore, mucosal integrity was partially restored in gastrointestinal tissues (Fig. S3E, F). Notably, CAS treatment at the established dosage did not induce significant brain tissue damage (Fig. S3B, C), demonstrating a favorable neurotoxicity profile compared to vinca alkaloids, such as vinblastine [41]. Also, CAS monotherapy demonstrated no obvious toxicity in the other examined organs (Fig. 3I and Fig. S3B, C).

In a subsequent subacute toxicity study, we systematically evaluated hematological parameters, organ function biomarkers, and inflammatory markers at baseline (Day 1) and after five consecutive administrations (Day 10). Neither CAS monotherapy nor combination therapy significantly altered hematological indices, indicating favorable hematological safety (Fig. S3G). In contrast, on Day 10, Cis monotherapy significantly elevated markers of hepatic (ALT, TBIL) and renal (BUN, CREA) dysfunction markers (Fig. S3H, I), accompanied by increased IL-6 levels (Fig. S3J). This confirms Cis-induced organ damage and an inflammatory response. While CAS monotherapy exhibited minimal hepatotoxicity (ALT: *p* = 0.1432; TBIL: *p* = 0.0804), whereas the combination treatment markedly reduced all functional marker elevations induced by Cis (Fig. S3G–J). These findings collectively suggest that CAS-Cis combination mitigates Cis-induced multiorgan toxicity through unidentified protective mechanisms.

### Combination therapy enhanced Cis efficacy and improved survival in tumor-bearing mice

**Fig. 4:**
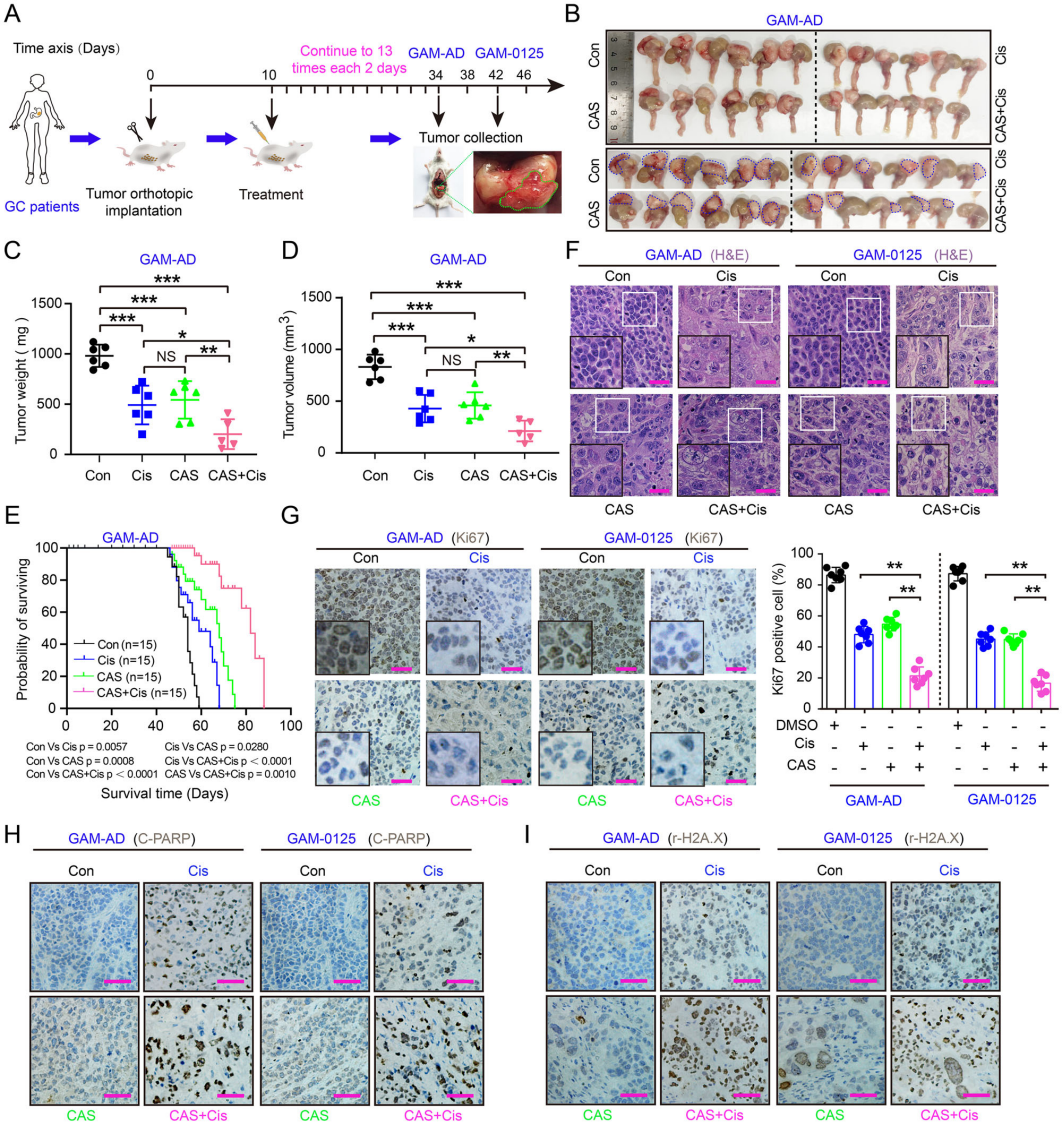
Combinatorial therapy enhances Cis efficacy and prolongs survival in PDX models. **A** Drug administration scheme in PDX models. **B** Representative tumors harvested from PDX model. The blue dashed lines indicate the location of the gastric tumor, (*n* = 6/group). Tumor weight (**C**) and volume (**D**) at indicated timepoints (scheme as **A**). **E** Kaplan-Meier survival curves of PDX mice in the natural growth conditions treated with saline solution, CAS (25 mg/kg), Cis (4 mg/kg) or both combinations (25 mg/kg CAS + 4 mg/kg Cis every 2 days) (*n* = 15/group). **F** H&E staining of tumors (Tumors were obtained from **B** and Fig. S4A). Black boxes highlight magnified regions. Scale bar = 150 μm. **G** Ki67 IHC with quantitation (bar chart), Black boxes highlight magnified regions. Scale bars = 100 μm. Cleaved PARP (C-PARP) (**H**) and γ-H2A.X (**I**) IHC in PDX tumors (Tumors were obtained from **B** and Fig. S4A). Scale bars = 150 μm. All data are shown as the mean ± SD and are representative of three independent experiments. *P < 0.05, **P < 0.01, and ***P < 0.001. NS no significance.

The survival benefit of combination therapy in tumor-bearing mice remain to be comprehensively elucidated. We therefore established PDX models of two GC patients to systematically evaluate treatment feasibility. After successful tumor engraftment, the mice receive administration of monotherapy or combination regimens administered every 2 days (Fig. 4A). Dual-therapy groups exhibited marked tumor regression across both PDX models, with complete tumor eradication observed in select cohorts (Fig. 4B and Fig. S4A). Quantitative analysis confirmed significant reductions in tumor weight (Fig. 4C and Fig. S4B) and volume (Fig. 4D and Fig. S4C) compared to monotherapy controls (*p* < 0.01).

To further investigate the impact of combination therapy on survival, we reestablished the orthotopic xenograft tumor models to evaluate the natural survival outcomes of tumor-bearing mice under various therapeutic regimens. Survival analysis revealed a longer median survival duration in the combination therapy group compared to single-agent groups (Fig. 4E and Fig. S4D). Histopathological assessment revealed pronounced histopathological lesions induced by combination therapy, particularly nuclear envelope disintegration (Fig. 4F). Proliferation marker analysis further showed lower Ki-67 indices in dual-treated tumors than in monotherapy counterparts (Fig. 4G). These data collectively validate the synergistic antitumor efficacy of the combinatorial approach.

### The combinatorial strategy increased the chemotherapeutic sensitivity of GC cells compared to Cis monotherapy

Our prior findings demonstrated the synergistic tumor-suppressing effects of Cis and CAS in GC models, both in vitro and in vivo (Fig. 2). To elucidate the underlying mechanism of this combinatorial efficacy, we examined CAS’s role in potentiating Cis-induced apoptosis. Flow cytometric analysis revealed enhanced drug sensitivity and apoptosis with CAS in combinations with Cis (Fig. S5A). This effect was further supported by elevated PARP cleavage in both cellular and xenograft models (Fig. 4H and Fig. S5B, C), confirming amplified apoptotic priming. Together, these data establish the superior cytotoxicity of the combination therapy compared to monotherapy at equivalent doses.

Cis demonstrates broad-spectrum antitumor efficacy by mechanistically mediating DNA purine base crosslinking and disrupting repair pathways, which triggers apoptosis through the accumulation of genomic lesions [25, 42]. To delineate the molecular basis of combinatorial synergism, we systematically investigated drug-induced DNA damage cascades. Integrated immunoblotting (Fig. S5B) analyses revealed a significant increase in H2A.X phosphorylation (γ-H2A.X) with combined therapy, as confirmed by IHC and IF assays (Fig. 4I and Fig. S5C). These findings suggest that CAS-Cis synergy enhances apoptosis by exacerbating DNA damage, establishing escalated DNA damage as a pivotal factor in combinatorial cytotoxicity.

### Synergistic amplification of Cis efficacy through ERS-AMPKα axis-driven autophagic overactivation and cytoskeletal destabilization

**Fig. 5:**
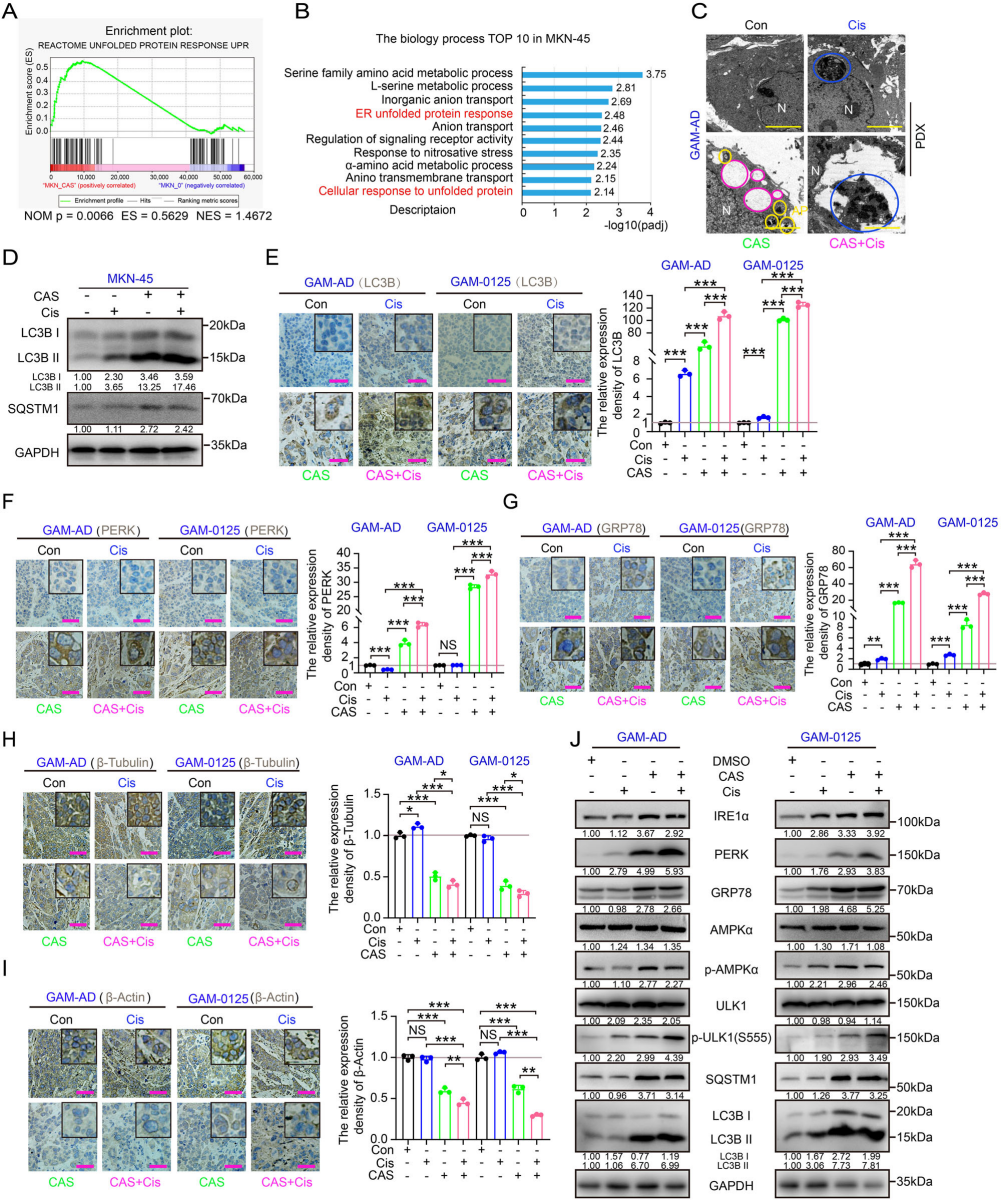
Combinatorial therapy enhances Cis efficacy via ERS-AMPKα axis-mediated autophagy accumulation and cytoskeletal disruption. **A** GSEA of UPR in MKN-45 cells treated with CAS (50 μM, 48 h) or DMSO (RNA-seq data). GSEA: gene set enrichment analysis; UPR: unfolded protein response. **B** Top 10 altered biological processes (BPs) in CAS-treated (50 μM) and MKN-45 cells (RNA-seq enrichment analysis). **C** TEM images of apoptotic bodies in tumor tissues (PDX model), Blue circles: apoptotic bodies; pink circles: dilated ER; yellow circles: autophagosomes. AP: autophagosomes. Scale bar = 2 μm. **D** LC3B and SQSTM1 were examined in MKN-45 cells treated with CAS (50 μM) or Cis (5 μM) alone or in combination. Relative protein expression levels were quantified based on band intensity. IHC staining of LC3B (**E**), PERK (**F**), GRP78 (**G**), β-tubulin (**H**) and β-actin (**I**) in PDX tumors (GAM-AD/GAM-0125. Scare bar = 150 μm. **J** Western blot analysis of ERS-AMPKα-ULK1 pathway proteins in PDX tumors (GAM-AD/GAM-0125). ‌Relative protein expression levels were quantified based on band intensity. All data are shown as the mean ± SD and are representative of three independent experiments. *P < 0.05, **P < 0.01, and ***P < 0.001. NS no significance.

To further delineate the molecular basis of combinatorial treatment in GC, we systematically investigated the physiological perturbations induced by CAS monotherapy. Transcriptomic profiling revealed that CAS monotherapy triggers the unfolded protein response (UPR) signaling through gene set enrichment analysis (Fig. 5A, B). Furthermore, multiple genes involved in ERS-associated physiological processes exhibited significant upregulation, including heat shock protein family members (HYOU1and DNAJB9) and transcriptional activator factors (ATF3 and ATF4) (Fig. S6A, B). The Transmission Electron Microscope (TEM) visualization also demonstrated ER expansion and cytoplasmic vesiculation in the cellular model and PDX tissues (Fig. 5C and Fig. S6C). Meanwhile, the parallel analyses identified cytoskeletal destabilization as a hallmark of CAS intervention. Significant downregulation of microfilament proteins, microtubule components, and keratin networks was confirmed via RNA-sequencing and subsequent RT-qPCR validation (Fig. S6D, E).

We next examined whether co-treating cells with CAS and Cis also caused above physiological changes. Co-treatment significantly increased LC3B-II and SQSTM1 (Inhibition of autophagic flux can cause SQSTM1 accumulation [43]) levels in GC cells and PDX tissues (Fig. 5D, E). TEM visualization of autophagosome accumulation (AP, autophagosome) corroborated these results (Fig. 5C). These results suggest that the combination treatment can lead to autophagy and block the flux. Mechanistic analysis revealed AMPKα activation (Thr172 phosphorylation) in the CAS-treated system (Fig. S6F). Parallel upregulation of ULK1 (Ser555 phosphorylation) coordinated with LC3B conversion, while mTORC1 pathway phosphorylation persisted, confirming AMPKα/ULK1 axis dominance over mTORC1 in autophagy induction. AMPKα can activate autophagy through activation of ULK1 or by inhibiting the mTORC1 signaling pathway, ULK1 can induce autophagy by phosphorylating Beclin-1 [44]. Notably, Genetic silencing of AMPKα or ULK1 substantially attenuated LC3B lipidation (Fig. S6G, H). Given established ER-autophagy crosstalk [45, 46]. We investigated the involvement of the ERS pathway. Dual blockade of the AMPKα, PERK, and IRE1α pathways (via shRNAs or the pharmacological inhibitors 4μ8C and STF-083010) synergistically reduced LC3B conversion more than single-pathway inhibition did (Fig. S6I, J). Taken together, these results indicate that CAS-mediated autophagy depends on the activation of the ERS-AMPKα axis.

We further investigated the effects of CAS-Cis co-treatment in PDX models, revealing enhanced ERS activation versus monotherapies via IHC (Fig. 5F, G). Combined therapy amplified cytoskeletal destruction (Fig. 5H, I), confirming that CAS-mediated autophagy inhibition and structural disruption are primary antitumor mechanisms. Pathway analysis showed that the ERS-AMPKα axis is persistently dependent on autophagic flux blockade, as evidenced by the hyperphosphorylation of core pathway proteins (Fig. 5J). This molecular dysregulation culminated in elevated DNA damage and apoptotic markers (Fig. 4I and Fig. S5C, S6K). Overall, the combination therapy enhances the efficacy of Cis through ERS-AMPKα-driven autophagic flux arrest and cytoskeletal collapse.

### The combinatorial strategy combats the individual toxicity of Cis through AMPKα-mediated autophagy homeostasis

**Fig. 6:**
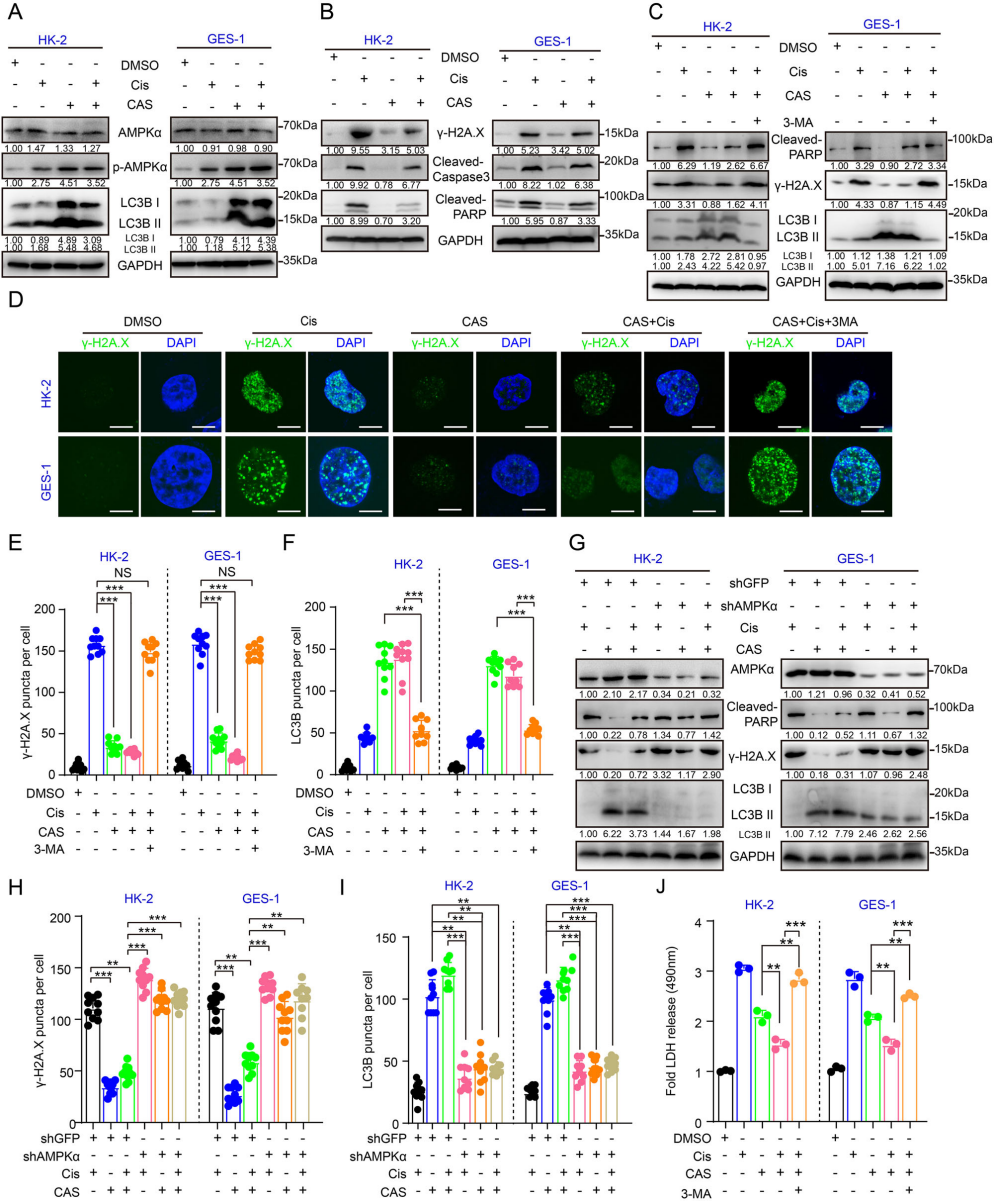
CAS mitigates Cis toxicity via AMPKα-mediated cytoprotective autophagy. **A** Western blot analysis of AMPKα, p-AMPKα, and LC3B in HK-2 and GES-1 cells treated with CAS (50 μM) and Cis (7 μM), alone or combined for 12 h. Relative protein expression levels were quantified based on band intensity. **B** Western blot analysis of γ-H2A.X, Cleaved-Caspase3, and Cleaved PARP in HK-2 and GES-1 cells treated with CAS (50 μM) and Cis (7 μM), alone or combined for 12 h. Relative protein expression levels were quantified based on band intensity. **C** Western blot analysis of Cleaved PARP, γ-H2A.X, and LC3B in HK-2 and GES-1 cells treated with CAS (50 μM), Cis (7 μM) and 3-MA (5 mM), alone or combined for 12 h. Relative protein expression levels were quantified based on band intensity. **D** Immunofluorescence analysis of γ‑H2A.X puncta per cell in HK‑2 and GES‑1 cells treated with CAS (50 μM), Cis (7 μM), and 3-MA (5 mM), alone or in combination for 12 h. Quantification of γ-H2A.X (**E**) and LC3B (**F**) puncta per cell in HK-2 and GES-1 cells treated with CAS (50 μM), Cis (7 μM), and 3-MA (5 mM), alone or combined for 12 h. **G** Western blot analysis of AMPKα, Cleaved PARP, γ-H2A.X and LC3B in GFP- or AMPKα-knockdown HK-2 and GES-1 cells treated with CAS (50 μM) and Cis (7 μM), alone or in combination for 12 h. Relative protein expression levels were quantified based on band intensity. Quantification of γ-H2A.X (**H**) and LC3B (**I**) puncta per cell in AMPKα-knockdown and control cells treated with CAS (50 μM) and Cis (7 μM), alone or in combination for 12 h. **J** The release of LDH was detected at 490 nm in AMPKα-knockdown and control cells treated with CAS (50 μM), Cis (7 μM), and 3-MA (5 mM), alone or in combination for 12 h. All data are shown as the mean ± SD and are representative of three independent experiments. *P < 0.05, **P < 0.01, and ***P < 0.001. NS no significance.

Our previous findings have demonstrated that the CAS-Cis combination can reduce Cis toxicity in non-cancerous tissues by activating AMPKα-mediated autophagy, which contrasts with its tumor-suppressive effects (Figs. 3, 5, and Fig. S6). To investigate autophagy’s role in toxicity mitigation, two non-cancerous cell lines, HK-2 (an immortalized proximal tubule epithelial cell line derived from a normal adult human kidney [47]) and the GES-1, as the models for toxicological evaluation. Western blotting and immunofluorescence revealed increase level of p-AMPKα and LC3B levels under combination treatment, confirming activation of the AMPKα-autophagy pathway (Fig. 6A). These findings underscore the pivotal role of AMPKα signaling activation in orchestrating downstream mechanistic cascades and hint at its regulatory potential in reducing Cis toxicity. Unlike tumor cell responses, this regimen suppressed Cis-induced DNA damage (γ-H2A.X) and apoptosis (cleaved PARP) while improving cell viability (Fig. 6B). This discovery precisely elucidates why the CAS-Cis combination reduces organ toxicity yet fails to resolve the mechanistic divergence in autophagic regulation between cancerous and non-cancerous cells. Interestingly, we found that blocking autophagy with 3-MA reversed the protective effects and elevated the levels of the cytotoxicity markers γ-H2A.X and cleaved PARP (Figs. 6C–F, J, and Fig. S7A). AMPKα knockdown similarly exacerbated DNA damage and apoptosis (Fig. 6G–I, and Fig. S7B, C), which validates the regulatory role of autophagy.

We further speculated that the combination therapy reduced toxicity and enhanced chemotherapeutic efficacy because the fact that autophagy plays different roles in the normal and tumor tissues. To address this issue, we investigated the effect of co-treatment on the autophagy activation status of no-cancerous and cancer cells under the same treatment conditions. The treatment duration and dose remained consistent with previous studies of tumor cells. Using the fluorescent labeling to detect LC3B and LAMP1, we observed a significant colocalization of LC3B and LAMP1 in the GES-1 cell line, indicating a circulating autophagic flux. In contrast, we did not observe this colocalization in the cancer cell lines under the same treatment conditions (Fig. S7D), indicating that the autophagic flux was blocked. These results establish a dual mechanism. The CAS-Cis-induced ERS/AMPKα axis protects normal cells by neutralizing Cis toxicity through autophagic regulation, while inducing cancer cell death via sustained autophagic flux blockade. These findings highlight cell type-specific autophagic modulation as the molecular foundation underlying the therapeutic superiority of combination therapy.

Previous studies have shown that under conditions of glucose deprivation, AMPKα inhibits the mTOR pathway and activates autophagy by phosphorylating ULK1 at Ser317, Ser777, and Ser555. Conversely, under nutrient-rich conditions, highly active mTOR inhibits ULK1-mediated autophagy by phosphorylating ULK1 at Ser757, which disrupts the ULK1-AMPKα interaction. These two states work together to regulate cellular the initiation of autophagy [48]. We wondered whether this coordinated mechanism could explain the differential autophagy states in normal versus tumor tissues during combined therapy. To validate the mechanistic differences in autophagy activation under these two distinct genetic backgrounds, we simultaneously examined changes in the AMPKα-ULK1 (Ser757) axis following CAS treatment alone or in combination. Our results demonstrate that autophagy activation in both cell types indeed depends on the AMPKα-ULK1 axis (with both p-AMPKα and p-ULK1 being upregulated) (Fig. S7E, F). However, there is a fundamental difference in the intensity of autophagy activation. In tumor cells‌, both CAS monotherapy and combination treatment maintained a certain level of mTOR activity. This indeed mediated an upregulation in p-ULK1 (Ser757) levels (Fig. S7E). In non-cancer cells, mTOR activity was significantly suppressed (with p-mTOR S2448 downregulated) by both CAS monotherapy and combination treatment, while p-ULK1 (Ser757) levels decreased significantly, indicating that autophagy was maintained at a relatively high level (Fig. S7F). Finally, we summarized the differences in autophagic flux across various cell types under combination therapy to further elucidate the dual roles of autophagy as “protective” and “destructive” in these cells (Fig. S7G).

## Discussion

Autophagy plays a dual role in cancer therapy. Moderate autophagy preserves cellular homeostasis by clearing damaged organelles and proteins, thereby promoting survival under chemotherapeutic stress, whereas excessive autophagy activation or persistent disruption of autophagic flux can trigger cell death [24, 49]. Most previous combination strategies have therefore focused on autophagy inhibition to overcome tumor resistance. For example, CQ and HCQ, currently the only FDA-approved autophagy inhibitors with demonstrated in vivo efficacy and clinical trial safety, primarily inhibit autophagic flux by suppressing autophagosome fusion and degradation, thereby inducing tumor cell death through autophagy [50, 51]. This research aims to enhance chemotherapy by dual mechanisms: (1) Enhance cytoprotection in normal tissues through autophagy-mediated clearance of Cis-induced toxicants, reducing organ damage. (2) Amplify lethality in tumors via tumor-selective autophagic flux blockade, exacerbating damage accumulation. The approach seeks to achieve toxicity reduction and efficacy enhancement simultaneously. Critical challenges lie in developing activators with tissue-specific regulatory capacity. Successful implementation requires precise control over autophagy dynamics, ensuring differential responses between malignant and non-malignant cells.

In present study, we used several evaluation criteria to screen the specific autophagic flux inhibitors from our constructed NCL. CA was ultimately validated as a potential candidate molecule that reduces Cis toxicity while enhancing chemotherapeutic efficacy. In mouse models, acute/subacute and chronic toxicity studies revealed that combination therapy dramatically alleviates Cis-mediated tissue toxicity (e.g., hepatic/renal injury, gastrointestinal damage, and IL-6 elevation) at observable Cis-induced damage doses. In orthotopic survival assays, the Cis-CA combination therapy cohort exhibited significantly prolonged survival. These results demonstrate that CA validates the aforementioned hypothesis regarding selective protective mechanisms, highlighting its potential translational and mechanistic significance.

We utilized CA as a model molecule to conduct exploratory research on its dual functions of toxicity reduction and therapeutic efficacy enhancement in Cis-based chemotherapy applications. Coincidentally, growing evidence confirmed that autophagy has become the most promising one among many detoxification mechanisms of Cis [16, 52–56]. However, these studies were only based on the explanation of the phenomenon, the specific and detailed mechanisms of action remain undetermined. CA is a synthetic precursor of the clinical antitumor drugs VCR and vinorelbine (VRB), literature indicating that CA (as a monomeric Vinca alkaloid) exhibits minimal tubulin-binding and cytotoxic activity compared to dimeric VCR/vinblastine. In fact, CA (or CAS) alone only weakly affects microtubule polymerization and spindle formation [57]. Additionally, we found that CA affects microfilament-associated proteins and keratins. Thus, it is unlikely that microtubule damage is the primary mechanism of its anticancer activity. This inference is consistent with our observations, if CA were acting like a classic microtubule poison, it would likely harm normal proliferating cells, contrary to our finding that it preferentially protects normal cells. Although CAS has some effect on the cytoskeleton, we conclude that CA’s enhancement of Cis efficacy is predominantly through autophagy modulation, rather than completely dependent on cytoskeleton disruption.

CA exhibits pharmacological properties, particularly in modulating autophagy through distinct molecular mechanisms. Through a series of experiments, we confirmed that CA induces ERS-dependent autophagy by simultaneously activating the PERK-AMPKα-ULK1 and IRE1α signaling pathways. Autophagy’s role is context-dependent: basal or moderate autophagy typically protects stressed cells by clearing damaged organelles and proteins, whereas excessive or sustained autophagy can become cytotoxic, leading to cell death [24]. This dichotomy may depend on factors such as autophagic flux intensity, magnitude, and cellular apoptotic pathway integrity. We further investigated divergences in the biological states and regulatory mechanisms of autophagic flux across diverse cellular contexts during combination therapy. These findings provide critical insights into the functional roles of autophagy in tumor cells versus normal tissues. HK-2 and GES-1 cells served as non-cancerous models to assess autophagic flux and cell survival. Results indicated that combination treatment activated AMPKα-ULK1-dependent autophagy in HK-2 and GES-1 cells. Notably, unlike in tumor cells, the CA-cisplatin combination protected normal cells by mitigating DNA damage and apoptosis while enhancing survival, demonstrating selective toxicity reduction. This protective effect was abolished upon inhibition of ERS, AMPKα, or autophagy. Autophagy facilitates the organized degradation and recycling of cellular components, serving as an adaptive response to maintain energy homeostasis under stress in both normal and malignant cells [11]. Here, we reveal that distinct autophagic flux states are concurrently activated in non-cancerous versus cancerous tissues. Despite identical drug exposure, CA fulfills key requirements for cisplatin-based chemotherapy by preserving cellular homeostasis in normal tissues while compromising tumor cell viability—a phenomenon underpinned by fundamentally divergent autophagic flux patterns. Specifically, while combination therapy activated autophagy in both cell types, autophagic flux status and intensity differed markedly: in cancer cells (treated with CA monotherapy or combination), mTOR activity persisted, but p-ULK1 (S757) levels increased and autophagic flux was obstructed, indicating partial autophagy suppression. In non-cancerous cells, autophagic flux remained active with suppressed p-mTOR and p-ULK1 (S757) activity, suggesting enhanced autophagic capacity. Regarding why mTOR activity is partially maintained in tumor cells, we propose the following logically inferred explanation grounded in empirical evidence and experimental observations: the intrinsically dysregulated oncogenic signaling pathways in tumor cells may confer a certain “stubbornness” to mTOR activity. For instance, many cancer cells exhibit PTEN loss or constitutive activation of the PI3K/Akt pathway [58, 59]. As a potent mTORC1 activator, sustained Akt signaling likely counteracts, to some extent, the inhibitory effects induced by AMPK activation. This results in mTOR activity persisting in a diminished yet not fully quiescent plateau phase. While this residual mTOR activity may be insufficient to block AMPK-driven ULK1 (Ser555) phosphorylation and autophagy initiation, its lingering effects-mediated through phosphorylation of alternative sites such as ULK1 Ser757-may impose restrictive fine-tuning on autophagic flux intensity or progression. Physiologically, functional autophagic flux enables rapid clearance of damaged components, whereas its blockade exacerbates cellular stress. These differential responses likely explain the contrasting survival outcomes across genetic contexts. As an autophagic flux inhibitor, CA primarily impedes autophagosome-lysosome fusion without altering lysosomal pH or function—a prerequisite for autophagy-mediated protection in normal tissues, which requires intact lysosomal activity. However, the mechanisms driving these context-dependent autophagic flux states warrant further investigation.

**Fig. 7:**
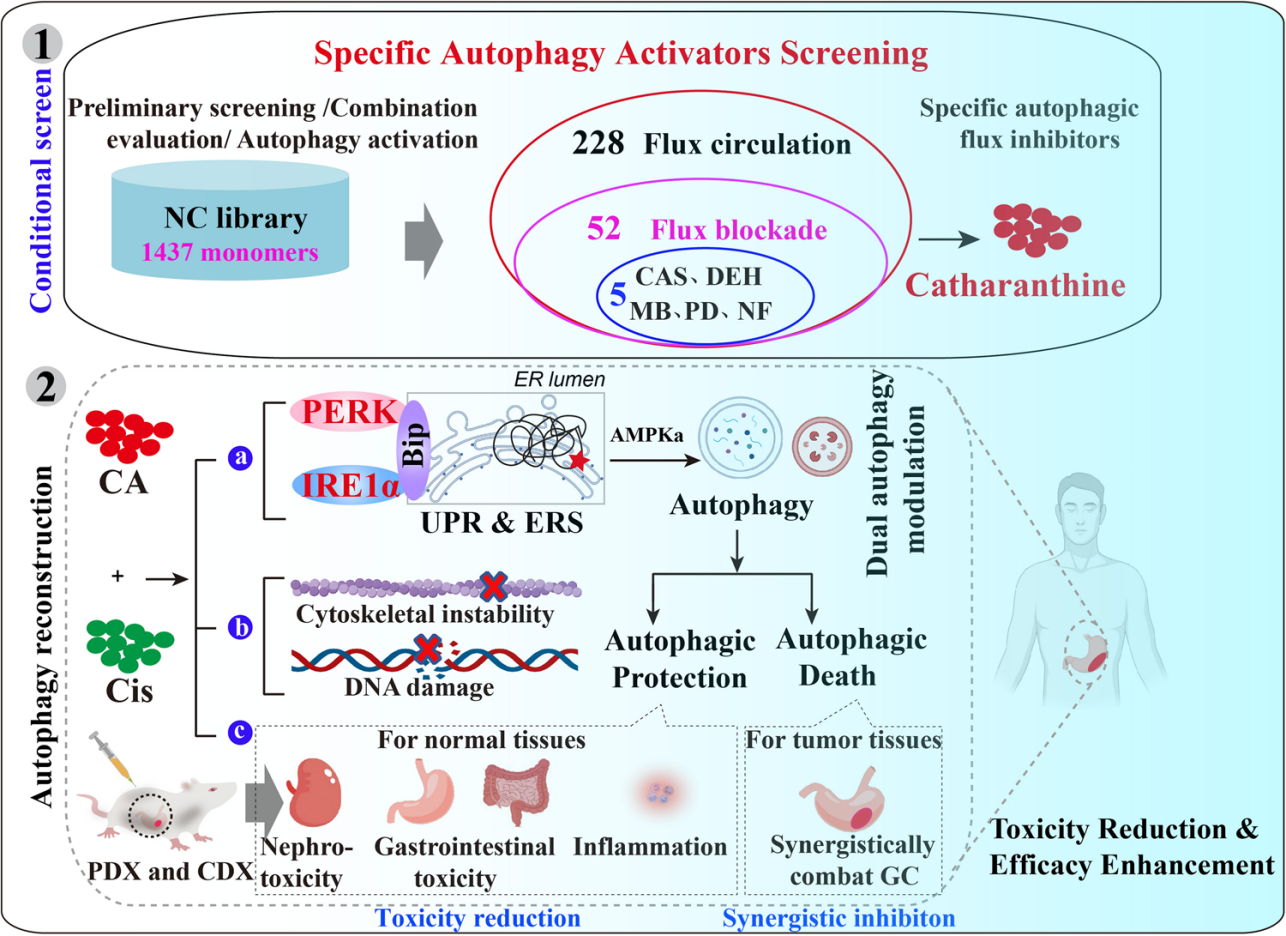
The diagram showing the study workflow and the dual role of CA-Cis combination therapy in protecting normal tissues and causing tumor autophagic damage via reestablishing autophagic homeostasis.

Overall, our findings (Fig. 7) demonstrate that multi-drug combination therapy can simultaneously enhance platinum-based chemotherapy efficacy and mitigate individual toxicity through ERS/AMPKα axis-driven autophagy activation and homeostasis remodeling. In this study, by establishing a hypothesis-driven conditional screening, we identified CAS as a dual-function autophagy modulator that enhances Cis-induced tumor suppression while preserving physiological homeostasis. Mechanistic studies revealed tissue-specific autophagy competency as the molecular basis: in normal cells, CAS activates ERS/AMPKα-dependent cytoprotective autophagy for detoxification, whereas in tumor cells with compromised autophagic flux, it induces lethal stress through cytoskeletal impairment and DNA damage amplification. Validated across CDX/PDX models and toxicity assessments. Our work demonstrates that autophagy modulators exhibit dual functionality in combination therapy-improving chemotherapeutic indices while preserving host physiological functions. These findings establish a conceptual framework for refining Cis-based regimens through autophagy competency-based modulation.

## Supplementary information

Supplementary Information

Supplementary Table

Fig. S1 Discovery of CAS as a specific autophagic flux inhibitor distinct from VCR.

Fig.S2 Cis-CAS combination acts synergistically to inhibit the proliferation of cisplatin-resistant cancer cells.

Fig.S3 Cis-CAS combination alleviates the individual toxicity induced by Cis chemotherapy in mice.

Fig.S4 Combinatorial therapy prolongs tumor-bearing mice survival in PDX models.

Fig.S5 Cis-CAS combination suppresses tumor growth via DNA damage-mediated apoptosis.

Fig. S6 CAS is a newly ERS/AMPKα-dependent autophagy activator.

Fig.S7 CAS alleviates cisplatin-induced toxicity by activating AMPKα-mediated protective autophagy.

Original western blots

aj-checklist

## Data Availability

All datasets used and/or analyzed during the current study are available from the corresponding author on reasonable request.
