# Supplementary material for: Enhancing platinum-based chemotherapy efficacy and safety through combination therapy-mediated remodeling of autophagic homeostasis in gastric cancer
Source: Cell Death Dis. 2026 Apr 22;17(1):532. doi: 10.1038/s41419-026-08703-3 (PMC13234414; doi:10.1038/s41419-026-08703-3)
Supplement: Supplementary file 1 — Supplementary Information [file 41419_2026_8703_MOESM1_ESM.doc]

**Enhancing platinum-based chemotherapy efficacy and safety through combination therapy-mediated** **remodeling of autophagic homeostasis in gastric cancer**

Guangzhao Pan^1,2,4,5#^, Qianqian Xu^2,3#^, Kui Zhang^1,4,5#^, Xin Hu^1,4,5#^, Chongyang Li^1,4^, Changhong Li^1,4^, Haoyan Ji^1, 4^, Xiaosong Hu^1, 4, 5^, Shaomin Shi^1^, Renjian Hu^1^, Chaowei Deng^1^, Erhu Zhao^1, 4, 5^, Jinfei Chen^6, 7*^ and Hongjuan Cui^1, 4, 5*^

**Contents**

**1. Supplementary Materials and Methods**

Reagents, Primers (**Table 1**), Antibodies (**Table 2**), Patient information (**Table 3**), Patient and Public Involvement Statement, Cell viability and proliferation assays, Single-dose acute toxicity, Subchronic toxicity study, Interspecies equivalent dose conversion, Lysosomal pH measurements, Subacute toxicity study (biochemical parameters, hematological profiles and markers of inflammation), and Plasma metabolic profiling and tissue distribution of CAS.

**2. References**

**3. Supplementary Figure Legends**

Fig.S1 Discovery of CAS as a specific autophagic flux inhibitor distinct from VCR.

Fig.S2 Cis-CAS combination acts synergistically to inhibit the proliferation of cisplatin-resistant cancer cells.

Fig.S3 Cis-CAS combination alleviates the individual toxicity induced by Cis chemotherapy in mice.

Fig.S4 Combinatorial therapy prolongs tumor-bearing mice survival in PDX models.

Fig.S5 Cis-CAS combination suppresses tumor growth via DNA damage-mediated apoptosis.

Fig. S6 CAS is a newly ERS/AMPKα-dependent autophagy activator.

Fig.S7 CAS alleviates cisplatin-induced toxicity by activating AMPKα-mediated protective autophagy.

**4. The detailed monomer information of the NCL**

**Table 4**

**Supplementary Materials and Methods**

**Reagents**

The Annexin V-FITC Apoptosis Detection Kit and propidium iodide (PI) were purchased from BD Pharmingen (BD, San Jose, CA, USA). Secondary antibodies were obtained from SeraCare Life Sciences, Inc. (USA): goat anti-mouse IgG (H+L) human serum adsorbed (Cat. No: 5220-0341) and goat anti-rabbit IgG (H+L) (Cat. No: 5220-0336). A small amount of catharanthine sulfate was obtained from our own laboratory and a large amount was purchased from the Chengdu MUST Biotechnology (A0672). Vincristine (A0674) was also purchased from Chengdu MUST Biotechnology. 4μ8C (HY-19707), STF-083010 (HY-15845) and cisplatin (HY-17394) were purchased from MedChemExpress Company (MCE). Chloroquine diphosphate salt (C662), rapamycin (R17), 3-methyladenine (M9281), 3-(4, -5-dimethylthiazol-2-yl]-2), 5-diphenyltetrazolium bromide (MTT, M5655) and dimethyl sulfoxide (DMSO, D5879) were purchased from Sigma-Aldrich. Bafilomycin A1 (S1413) and wortmannin (S2758) were purchased from Selleck Chemicals. The Lipofectamine 2000 transfection reagent (11668019) was purchased from Thermo Fisher Scientific, and a hematoxylin and eosin staining kit was purchased from Beyotime (C0105).

**Primers**

Primer information is shown in **Table 1.**

**Table 1** Detailed information on the primers used in this study

| **Targets** | **Primer sequences** | **Properties** |
| --- | --- | --- |
| shPERK 1# | forward sequence: 5'-CCGGTTTGTCCCTGGCGGGTAAATTCTCGAGAATTTACCCGCCAGGGACAAATTTTTG-3’  reverse sequence： 5'-AATTCAAAAATTTGTCCCTGGCGGGTAAATTCTCGAGAATTTACCCGCCAGGGACAAA-3’ | shRNA |
| shPERK 2# | forward sequence: 5'-CCGGCGGCAGGTCATTAGTAATTATCTCGAGATAATTACTAATGACCTGCCGTTTTTG-3'  reverse sequence： 5'-AATTCAAAAACGGCAGGTCATTAGTAATTATCTCGAGATAATTACTAATGACCTGCCG-3' | shRNA |
| shIRE1α | forward sequence:  5'-CCGGACGCTTGGAAGCAAGAATAATCTCGAGATTATTCTTGCTTCCAAGCGTTTTTTG-3'  reverse sequence  5'-AATTCAAAAAACGCTTGGAAGCAAGAATAATCTCGAGATTATTCTTGCTTCCAAGCGT-3' | shRNA |
| shBeclin-1 | forward sequence:  5'-CCGGGCTTGGGTGTCCTCACAATTTCTCGAGAAATTGTGAGGACACCCAAGCTTTTTG-3'  reverse sequence  5'-AATTCAAAAAGCTTGGGTGTCCTCACAATTTCTCGAGAAATTGTGAGGACACCCAAGC-3' | shRNA |
| shULK1 | forward sequence:  5'-CCGGCTTCCAGGAAATGGCTAATTCCTCGAGGAATTAGCCATTTCCTGGAAGTTTTTG-3'  reverse sequence  5'-AATTCAAAAACTTCCAGGAAATGGCTAATTCCTCGAGGAATTAGCCATTTCCTGGAAG-3' | shRNA |
| shAMPKα | forward sequence:  5'-CCGGGTTGCCTACCATCTCATAATACTCGAGTATTATGAGATGGTAGGCAACTTTTTG-3'  reverse sequence  5'-AATTCAAAAAGTTGCCTACCATCTCATAATACTCGAGTATTATGAGATGGTAGGCAAC-3' | shRNA |
| shATF4 | forward sequence:  5'-CCGGCCTAGGTCTCTTAGATGATTACTCGAGTAATCATCTAAGAGACCTAGGTTTTTG-3'  reverse sequence  5'-AATTCAAAAACCTAGGTCTCTTAGATGATTACTCGAGTAATCATCTAAGAGACCTAGG-3' | shRNA |
| GAPDH | forward sequence: GGAGCGAGATCCCTCCAAAAT  reverse sequence: GGCTGTTGTCATACTTCTCATGG | RT-qPCR |
| DDIT3(CHOP) | forward sequence: GGAAACAGAGTGGTCATTCCC  reverse sequence: CTGCTTGAGCCGTTCATTCTC | RT-qPCR |
| IGFBP1 | forward sequence: TTGGGACGCCATCAGTACCTA  reverse sequence: TTGGCTAAACTCTCTACGACTCT | RT-qPCR |
| PPP1R15A | forward sequence: ATGATGGCATGTATGGTGAGC  reverse sequence: AACCTTGCAGTGTCCTTATCAG | RT-qPCR |
| HERPUD1 | forward sequence: CCGGTTACACACCCTATGGG  reverse sequence: TGAGGAGCAGCATTCTGATTG | RT-qPCR |
| HSPA5(GRP78) | forward sequence: GAAAGAAGGTTACCCATGCAGT  reverse sequence: CAGGCCATAAGCAATAGCAGC | RT-qPCR |
| ATF4 | forward sequence: CCCTTCACCTTCTTACAACCTC  reverse sequence: TGCCCAGCTCTAAACTAAAGGA | RT-qPCR |
| ATF3 | forward sequence: CCTCTGCGCTGGAATCAGTC  reverse sequence: TTCTTTCTCGTCGCCTCTTTTT | RT-qPCR |
| NCK2 | forward sequence: GTGATAGCCAAGTGGGACTACA  reverse sequence: TAGTTGGACGGTACATAGCCC | RT-qPCR |
| ASNS | forward sequence: CATTACAACAGTTCGTGCTTCAG  reverse sequence: CACCACGCTATCTGTGTTCTT | RT-qPCR |
| IRE1α(ERN1) | forward sequence: AGAGAAGCAGCAGACTTTGTC  reverse sequence: GTTTTGGTGTCGTACATGGTGA | RT-qPCR |
| DNAJB9 | forward sequence: TCTTAGGTGTGCCAAAATCGG  reverse sequence: TGTCAGGGTGGTACTTCATGG | RT-qPCR |
| HYOU1 | forward sequence: GAGGAGGCGAGTCTGTTGG  reverse sequence: GCACTCCAGGTTTGACAATGG | RT-qPCR |
| GFPT1 | forward sequence: GGAATAGCTCATACCCGTTGG  reverse sequence: TCGAAGTCATAGCCTTTGCTTT | RT-qPCR |
| WIPI1 | forward sequence: ACTAAAGCCGGGTATAAGCTGT  reverse sequence: CGGGATTTCATTGCTTCCGTG | RT-qPCR |
| SYVN1 | forward sequence: CTTCACCGTTTTTCGGGATGA  reverse sequence: CCAGGAGGAACATAAGAGAGACA | RT-qPCR |
| TPP1 | forward sequence: GTTTCATCACTATGTGGGAGGAC  reverse sequence: GTATCGCTTACGGATCACAGAG | RT-qPCR |
| PREB | forward sequence: TGTGTGCTTCAACCACGATAAT  reverse sequence: CATCAGGCCCTAAAGCCAGG | RT-qPCR |
| SERP1 | forward sequence: AAATGCCCCCGAAGAGAAGG  reverse sequence: TCTGGAAAATTGCAGAACCACA | RT-qPCR |
| PPP2R5B | forward sequence: GCGTGAGTACCTCAAGACCAT  reverse sequence: TTGCACTGTTTGCGGATGTAG | RT-qPCR |
| ACTB (β-Actin) | forward sequence: CATGTACGTTGCTATCCAGGC  reverse sequence: CTCCTTAATGTCACGCACGAT | RT-qPCR |
| ACTG1 | forward sequence: CCGAGCCGTGTTTCCTTCC  reverse sequence: GCCATGCTCAATGGGGTACT | RT-qPCR |
| ACTR2 | forward sequence: CACCTGTGGGACTACACATTTG  reverse sequence: TGGTTGGGTTCATAGGAGGTTC | RT-qPCR |
| ARPC3 | forward sequence: GTGCAATTCCAAAAGCCAAGG  reverse sequence: GGCTCTCATCACTTCATCTTCC | RT-qPCR |
| TUBB4B | forward sequence: GGACAACTTCGTTTTCGGTCA  reverse sequence: CCTTTCTCACAACATCCAGCAC | RT-qPCR |
| TUBB | forward sequence: AAGATCCGAGAAGAATACCCTGA  reverse sequence: CTACCAACTGATGGACGGAGA | RT-qPCR |
| NDC80 | forward sequence: CCTCTCCATGCAGGAGTTAAGA  reverse sequence: GGTCTCGGGTCCTTGATTTTCT | RT-qPCR |
| TPM1 | forward sequence: TTGAGAGTCGAGCCCAAAAAG  reverse sequence: CATATTTGCGGTCGGCATCTT | RT-qPCR |
| TPM2 | forward sequence: CTGAGACCCGAGCAGAGTTTG  reverse sequence: TGAATCTCGACGTTCTCCTCC | RT-qPCR |
| TPM3 | forward sequence: TGAAAACCGGGCCTTAAAAGAT  reverse sequence: GATCACCAACTTACGAGCCAC | RT-qPCR |
| TPM4 | forward sequence: GAGGTAGCTCGTAAGCTGGTC  reverse sequence: ACCGTTCTCTCTGCAAATTCAG | RT-qPCR |
| MYL6 | forward sequence: GAAGACCAGACCGCAGAGTTC  reverse sequence: TCCAGCACCTTCACATTCATC | RT-qPCR |
| MYO1C | forward sequence: TGGGCAACATCCACTTTGCT  reverse sequence: GGAGCCTGGTCAGATACTTGAG | RT-qPCR |
| MYO1E | forward sequence: AAGGAGCGGCACAGTATGAAA  reverse sequence: TCACCACTGATAATGACGCAC | RT-qPCR |
| MYH9 | forward sequence: CCTCAAGGAGCGTTACTACTCA  reverse sequence: CTGTAGGCGGTGTCTGTGAT | RT-qPCR |
| MYLK | forward sequence: CCCGAGGTTGTCTGGTTCAAA  reverse sequence: GCAGGTGTACTTGGCATCGT | RT-qPCR |
| MYLK3 | forward sequence: GCGGATTTCCTCATGCAGG  reverse sequence: AGCACATGCTTTGGTTTTCCT | RT-qPCR |
| MYLIP | forward sequence: CATCTTACAGGAGCAGACTAGGC  reverse sequence: TTGGCAGTGTTCTGGTTGTAG | RT-qPCR |
| KRT15 | forward sequence: GACGGAGATCACAGACCTGAG  reverse sequence: CTCCAGCCGTGTCTTTATGTC | RT-qPCR |
| KRT17 | forward sequence: GGTGGGTGGTGAGATCAATGT  reverse sequence: CGCGGTTCAGTTCCTCTGTC | RT-qPCR |
| Actin | forward sequence: CCGAGCCGTGTTTCCTTCC  reverse sequence: GCCATGCTCAATGGGGTACT | RT-qPCR |
| B2M | forward sequence: GAGGCTATCCAGCGTACTCCA  reverse sequence: CGGCAGGCATACTCATCTTTT | RT-qPCR |

**Antibodies**

The antibodies used in this study are shown in **Table 2**.

**Table 2** Detailed information on the primary antibodies used in this study

| **Antibodies** | **SOURCE** | **IDENTIFIER** | |
| --- | --- | --- | --- |
| CTSB | Proteintech | 12216-1-AP | |
| CTSD | Cell Signaling Technology | | #2284 |
| CTSL | Santacruz | sc-32801 | |
| ABCB1 | Santacruz | sc-13131 | |
| ABCG2 | Santacruz | sc-69988 | |
| RAD51 | Proteintech | 14961-1-AP | |
| BRCA1 | Proteintech | 83390-6-RR | |
| Bcl-2 | Proteintech | 12789-1-AP | |
| Bax | Proteintech | 50599-2-Ig | |
| GAPDH | Cell Signaling Technology | #5174 | |
| LC3B | Abcam | ab192890 | |
| LC3B | Cell Signaling Technology | #3868 | |
| SQSTM1 | Cell Signaling Technology | #88588 | |
| α-Actin | Beyotime | AA132 | |
| β-Actin | Abcam | ab88224 | |
| F-Actin | Abcam | ab130935 | |
| PERK | Cell Signaling Technology | #5683 | |
| GRP78 | Cell Signaling Technology | #3177 | |
| CHOP | Cell Signaling Technology | #2895 | |
| IRE1α | Cell Signaling Technology | #3294 | |
| Beclin-1 | Cell Signaling Technology | #3495 | |
| LAMP1 | Cell Signaling Technology | #9091 | |
| mTOR | Cell Signaling Technology | #2983 | |
| p-mTOR Ser2448 | Cell Signaling Technology | #5536 | |
| AMPKα | Cell Signaling Technology | #5832 | |
| p-AMPKα Thr172 | Cell Signaling Technology | #8208 | |
| ULK1 | Cell Signaling Technology | #8054 | |
| p-ULK1Ser555 | Cell Signaling Technology | #5869 | |
| p-ULK1Ser757 | Cell Signaling Technology | #6888T | |
| PARP | Cell Signaling Technology | #9532 | |
| Cleaved-PARP | Cell Signaling Technology | #5625 | |
| H2A.X | Cell Signaling Technology | #2595 | |
| γ-H2A.X Ser139 | Cell Signaling Technology | #9718 | |
| γ-H2A.X Ser139 | Cell Signaling Technology | #80312 | |
| Caspase3 | Cell Signaling Technology | #9579 | |
| Ki67 | BD Biosciences | 550609 | |

**Patient information**

Patient information is shown in Table 3.

**Table 3 Detailed information on the patient's tumor tissues used in this study**

| **Patient 1**  **Name: GAM-AD** | |
| --- | --- |
| **Diagnosis time** | 2019/06/05 |
| **Diagnostic hospital** | The Ninth People’s Hospital of Chongqing (the Affiliated Hospital of Southwest University), Beibei, China |
| **Tumor type** | Gastric cancer |
| **Gender** | Male |
| **Patient age** | 65 |
| **Stage** | T4N1M0 |
| **Grade** | Ⅱ-Ⅲ |
| **Clinical latency** | Unknown |
| **Pathology diagnosis** | Moderately-poorly differentiated adenocarcinoma of the gastric body (ulcerative type); corpus gastric carcinoma. Adenocarcinoma of corpus gastric, ulcerative type, moderately and poorly differentiated, tumor mass 3.5 cm×3.5 cm×1.5 cm，invading into adipose tissue out. |
| **Chinese diagnosis** | Diagnosed as gastric body cancer; the site of the examination was the billrothii; pathological diagnosis: Gastric corpus carcinoma. Adenocarcinoma of the gastric corpus, ulcerative type, moderately and poorly differentiated, tumor size 3.5 cm × 3.5 cm × 1.5 cm, invading into adipose tissue. No cancer was found on the upper and lower cut edges. Metastatic carcinoma was seen in the lymph nodes of the gastric lesser curvature (5/5), and no metastatic carcinoma was seen in the lymph nodes of the gastric greater curvature (0/2). |
| **Other information** | Kras (WT) PIK3CA (WT) |

| **Patient 2**  **Name: GAM-0125** | |
| --- | --- |
| **Diagnosis time** | Unclear |
| **Diagnostic hospital** | The Ninth People’s Hospital of Chongqing (the Affiliated Hospital of Southwest University), Beibei, China |
| **Tumor type** | Gastric cancer |
| **Gender** | Male |
| **Patient age** | 31 |
| **Stage** | T4N1M0 Ⅲ |
| **Grade** | Ⅲ |
| **Clinical latency** | Unknown |
| **Pathology diagnosis** | Poorly differentiated adenocarcinoma on lesser curvature side of esophagogastric junction (ulcerative); Adenocarcinoma derived from lesser gastric curvature of juncture of stomach and esophagus, protrude, ulcerative and poorly differentiated, tumor mass 6 cm. |
| **Chinese diagnosis** | An ulcerative poorly differentiated adenocarcinoma with a size of 6 × 5 × 1.5 cm is raised on the lesser curvature of the gastroesophageal junction. The tumor invades the entire thickness of the stomach wall and involves the oesophagus. Cancer is seen in the lymph nodes of the minor curve (3/14). Immunohistochemical staining showed tumor cells: HER-1 (+), HER-2 (-), p53 (+ 25-50%), p170 (-), Ki-67 (+ 25-50%), VEGF (+ +), Top-Ⅱα (+ about 10%), p16 (-). |
| **Other information** | Unknown |

**Patient and Public Involvement Statement**

The patients promised to voluntarily donate their pathological tissues (the names of which were provided by the authors) for scientific research by scientists under anonymous conditions. The authors declare that the patients had a relatively good understanding of the study's content, had carefully read the final manuscript, and had agreed to the publication of the research results.

**Cell viability and proliferation assays**

A total of 800-1,000 cancer cells were seeded in 96-well plates and treated with CAS in a time- or dose-dependent manner. MTT (20 μL, 5 mg/mL) was added to each well and incubated at 37 ℃ for 3 h. Each well was then supplemented with 200 μL of DMSO to dissolve the formazan. OD values were measured by a microplate reader (Thermo Fisher, Waltham, MA, USA) at 490 nm and room temperature. Finally, cell viability was normalized to the control group and analyzed.

**Single-dose** **acute toxicity**

The LD_50_ of CAS in mice was determined using the modified Kärber assay [1]. Healthy Kunming mice (8 weeks old) housed in a standard specific pathogen-free (SPF) -grade animal rooms were randomly divided into six groups (n = 20 per group). CAS administration concentrations were set at 1000, 714.3, 510.2, 364.4, 260.3, and 185.9 mg/kg, respectively. After single-dose intraperitoneal injection, the physiological status and mortality of the mice were monitored and recorded for 14 days to enable the statistical analysis of the relevant indicators. The LD_50_ of the drug was calculated using the modified Kärber assay [2].

To further validate the time-response characteristics of the Cis-CAS combination therapy strategy for reducing toxicity, Kunming mice were randomly assigned to four groups and received single-dose treatments of an equal volume of either saline (control), 150 mg/kg CAS alone, 12 mg/kg Cis alone, or a combination of 150 mg/kg CAS and 12 mg/kg Cis. Observations were made at 24 h post-treatment, and organ tissues were collected for pathological examination to assess short-term tissue damage.

**Subchronic toxicity study**

To mitigate potential discrepancies in toxicity evaluation attributable to immune deficiencies, NOD/SCID (immunodeficient) and Kunming (immunocompetent) mice were employed concurrently for subchronic toxicity assessment. Specifically, the mice were randomized into four treatment groups, each receiving an equal volume of saline (control), 4 mg/kg Cis alone, 25 mg/kg CAS alone, or a combination of 4 mg/kg Cis and 25 mg/kg CAS. Treatments were administered via ‌intraperitoneal injection every 48 h for over 34 days. Following treatment, major organs were harvested for comprehensive ‌histopathological analysis‌.

**Interspecies equivalent dose conversion**

Interspecies dose conversion was performed using body surface area-based normalization (BSA-based normalization), in accordance with FDA guidelines and ICH S9 principles for the equivalent dosing of anticancer drugs [3]. The formula for calculating the human equivalent dose (HED) is as follows:


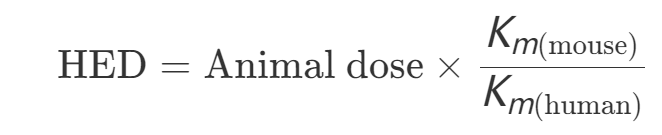


*Km* indicates body surface area per unit body weight; *Km*(Adults)≈37, *Km* (mouse) ≈3[4].

**Lysosomal pH measurements**

Lysosomal pH was measured by LysoSensor Yellow/Blue DND-160 (Molecular Probes) staining, as previously reported [5]. In brief, MKN1 and MKN-45 cells were labeled with 5 µM LysoSensor Yellow/Blue DND-160 for 5 min at 37 °C, followed by two PBS washes. The labeled cells were then treated with 10 µM monensin (Santa Cruz Biotechnology, sc-200109) and 10 µM nigericin (Invitrogen, N1495) in a calibration buffer containing 25 mM 2-(N-morpholino) ethanesulfonic acid (MES; Amresco, X218), 5 mM NaCl, 115 mM KCl and 1.2 mM MgSO₄ (pH 3.5-6.0) for 2 min on ice. Fluorescence emission at 535 nm was measured upon excitation at 340 nm and 380 nm. A pH calibration curve was generated by plotting the ratio of emission intensities (340 nm/380 nm excitation) against pH values. This curve was subsequently applied to determine the lysosomal pH in MKN1 and MKN-45 cells.

**Subacute toxicity study (biochemical parameters, hematological profiles and markers of inflammation)**

Healthy 9-week-old Kunming mice were housed in a standard SPF facility. Following environmental acclimation, the mice were randomly divided into four groups (n = 6 per group):‌ Control group‌ (intraperitoneal saline injection), Cis monotherapy group‌ (4 mg/kg single dose), CAS monotherapy group‌ (25 mg/kg single dose) and Cis-CAS combination group‌ (4 mg/kg Cis + 25 mg/kg CAS). At 24 h‌ post-initial dosing, blood was collected from all mice using microsampling, with the sampling day designated as Day 1. Subsequent doses were administered at 48 h intervals for a total of five doses, followed by terminal blood collection. The following were monitored and recorded after each administration: general appearance, behavioral activity, mental status, visible secretions and excretions, mortality, and toxic reaction symptoms (including onset time, severity, duration, reversibility, and recovery time). Blood samples were promptly delivered to Sevier Biotechnology Co., Ltd. for biochemical and ELISA analysis. Complete blood cell (CBC) analysis was performed using a Mindray Veterinary Automated Hematology Analyzer (Model: BC-2800vet). Plasma biochemical analysis was conducted according to the operational manual of Sevier Biotechnology Co., Ltd., utilizing automated biochemistry analyzers (Models: Chemray 240 and Chemray 800, Rayto Life and Analytical Sciences Co., Ltd., Shenzhen, China).

**Plasma metabolic profiling and tissue distribution of CAS**

In brief, plasma metabolic profiling was performed on Kunming mice following a single administration of CAS (i.p., 25 mg/kg). Blood samples were collected 24 h after treatment and sent to Sevier Biotechnology Co., Ltd. for HPLC-MS analysis. The results were then cross-referenced against an established metabolite library to identify the compounds) (For plasma metabolite identification results from CAS analyses, please refer to the Supplementary Table). For CAS tissue distribution analysis: Orthotopic tumor-bearing mice were administered CAS (i.p., 25 mg/kg). Kidney and tumor tissue samples were collected 48 h after dosing and submitted to Sevier Biotechnology Co., Ltd. for HPLC-MS analysis.

**References**

1. Akhila J, Alwar M. Acute toxicity studies and determination of median lethal dose. Curr Sci. 2007;93.

2. LD50 (Median Lethal Dose). In: Pant AB, editor. Dictionary of Toxicology. Singapore: Springer Nature Singapore; 2024. p. 559-.

3. Ponce R. ICH S9: Developing anticancer drugs, one year later. Toxicol Pathol. 2011;39:913-5.

4. Jacob S, Nair A, Morsy M. Dose Conversion Between Animals and Humans: A Practical Solution. Indian Journal of Pharmaceutical Education and Research. 2022;56:600-7.

5. Zhou J, Li G, Zheng Y, Shen HM, Hu X, Ming QL, et al. A novel autophagy/mitophagy inhibitor liensinine sensitizes breast cancer cells to chemotherapy through DNM1L-mediated mitochondrial fission. Autophagy. 2015;11:1259-79.

**Supplementary Figure Legends**

**Fig.S1 Discovery of CAS as a specific autophagic flux inhibitor distinct from VCR. A** Screening workflow for identifying Cis-synergistic autophagic flux inhibitors from the NC library. **B** Chemical structures of CA and VCR. **C** Representative images of cellular morphology following 24 h exposure to CA or VCR. Scale bar = 150 μm in the original image, and 100 μm in the magnified view. **D** Analysis of cell apoptosis by flow cytometry following treatment with 20 μM CAS (the sulfate form of CA) for 48 h. **E** Analysis of cell cycle by flow cytometry following treatment with the indicated concentrations of CAS for 48 h. **F** Co-localization analysis of eGFP-LC3B (green) and mRFP-LC3B (red) puncta in GES-1 and MKN-45 cells treated with VCR (0.5 μM) for 6 h. Scale bar = 50 μm. **G** Immunofluorescence staining was performed to observe the morphology of the microtubule after treatment with CAS (50 μM for 24 h), VCR (0.5 μM for 6 h), and ice-water mixture (30 min). **H** Representative fluorescence micrographs of acridine orange (Ao) staining in MKN1 and MKN-45 cells following 24 h exposure to 50 μM CAS. Scale bar = 50 μm. **I** Lysosomal pH quantification using LysoSensor Yellow/Blue DND‑160 in MKN1 and MKN‑45 cells treated with or without CAS (50 μM) for 48 h. **J** Western blot analysis of pro-CTSB, mature-CTSB, pro-CTSD, mature-CTSD, pro-CTSL, and mature-CTSL in MKN1 and MKN-45 cells treated with CAS 50 μM for 48 h. Relative protein expression levels were quantified based on band intensity. CTSB, cathepsin B; CTSD, cathepsin D; CTSL, cathepsin L. **K** MTT assay to investigate the cell viability of MKN-45, MKN1, HGC27, NUGC4 and GES-1 cells treated with the indicated concentrations of CAS for 24 h. **L** MTT assay to determine the EC_50_ value of NUGC4 and MKN-45 cells treated with different concentrations of CAS for 48 h. All data are shown as the mean ± SD. *P < 0.05, **P < 0.01, and ***P < 0.001. NS = no significance.

**Fig.S2 Cis-CAS combination acts synergistically to inhibit the proliferation of cisplatin-resistant cancer cells. A** Detection of the Cis resistance index (RI) using MTT assay in Cis-resistant cell line MKN-45 DR. **B** ‌Western blot analysis of ABCB1, ABCG2, RAD51, BRCA1, Bcl-2, and Bax expression in MKN-45 WT and MKN-45DR cells at different passages (G1-G9). Relative protein expression levels were quantified based on band intensity. WT: wild type; G1-G9: generation 1-9. **C** Cell proliferation assessed by MTT assay in MKN-45DR cells treated with the indicated concentrations of Cis or CAS, alone or in combination with 50 μM CAS for 48 h. **D, E** Colony formation assay of MKN-45DR cells treated with 50 μM CAS and the indicated concentrations of Cis (5.06, 7.59, and 11.39 μM), alone or in combination. Representative images of crystal violet-stained colonies on day 15 are shown (**D**). Quantified colony numbers from three technical replicates are presented as mean ± SD. (**E**). **F** Combination effect of 50 μM CAS with the indicated concentrations of Cis (5.06, 7.59, and 11.39 μM) was evaluated using Jin's modified Bürgi formula. *P < 0.05, **P < 0.01, and ***P < 0.001. NS = no significance.

**Fig.S3 Cis-CAS combination alleviates the individual toxicity induced by Cis chemotherapy in mice.** **A** Analysis of CAS residues in kidney and gastric tumor tissues from PDX mice 48 h after a single administration of CAS (25 mg/kg), as determined by HPLC-MS. **B, C** H&E staining showing pathological alterations in the heart, lung, brain, and muscle tissues from mice in the single-dose acute (**B**; CAS, 150 mg/kg; Cis, 12 mg/kg) and subchronic (**C**; CAS, 25 mg/kg; Cis, 4 mg/kg) toxicity studies, administered alone or in combination. Scale bars = 200 μm. **D** H&E staining of liver from mice in the single-dose acute (CAS, 150 mg/kg; Cis, 12 mg/kg) and subchronic (CAS, 25 mg/kg; Cis, 4 mg/kg) toxicity studies, administered alone or in combination. The yellow arrows indicate damaged hepatic lobules. Scale bars = 200 μm. **E, F** H&E staining of spleen, kidney, gut, and stomach tissues from mice in the single-dose acute (**F**; CAS, 150 mg/kg; Cis, 12 mg/kg) and subchronic (**E**; CAS, 25 mg/kg; Cis, 4 mg/kg) toxicity studies, administered alone or in combination. The yellow arrows indicate representative sites of tissue injury. Scale bars = 200 μm. **G** Complete blood count analysis of mice from the subacute toxicity study following treatment with saline, Cis (4 mg/kg), CAS (25 mg/kg), or their combination (Cis 4 mg/kg + CAS 25 mg/kg). **H, I** Serum biochemical parameters assessing liver (**H**) (ALT and TBIL) and kidney (**I**) (BUN and CREA) function in mice from the subacute toxicity study treated with saline, Cis (4 mg/kg), CAS (25 mg/kg), or their combination. ALT, alanine aminotransferase; TBIL, total bilirubin; BUN, blood urea nitrogen; CREA, creatinine. J Serum IL-6 levels in mice measured by ELISA following treatment with saline, Cis (4 mg/kg), CAS (25 mg/kg), or their combination in the subacute toxicity study. *P < 0.05, **P < 0.01, and ***P < 0.001, NS = no significance.

**Fig.S4 Combinatorial therapy prolongs tumor-bearing mice survival in PDX models. A** Representative tumor images harvested from the GAM-0125 PDX model. The blue dashed lines indicate the location of the gastric tumor, n = 3/group). **B, C** Tumor weight (**B**) and volume (**C**) at indicated timepoints (scheme as **Fig. 4A**). **D** Kaplan-Meier survival curves of PDX mice treated with saline solution, CAS (25 mg/kg), Cis (4 mg/kg) or their combination (25 mg/kg CAS + 4 mg/kg Cis every two days) (n = 3/group). *P < 0.05, **P < 0.01, and ***P < 0.001, NS = no significance.

**Fig.S5 Cis-CAS combination suppresses tumor growth via DNA damage-mediated apoptosis. A** Flow cytometric analysis of apoptosis in MKN-45 cells treated with Cis (5 μM) and/or CAS (50 μM) for 24 h. **B** Western blot analysis of PARP, cleaved-PARP, H2A.X, and γ-H2A.X in MKN-45 cells treated with Cis (5 μM) and/or CAS (50 μM) for 48 h. Relative protein expression levels were quantified based on band intensity. **C** Immunofluorescence visualization of γ-H2A.X foci (green) in MKN-45 cells treated with Cis (5 μM) and/or CAS (50 μM) for 24 h. Nuclei were counterstained with DAPI (blue). The graph shows the quantified number of γ-H2A.X foci per cell. Scale bar = 50 μm. *P < 0.05, **P < 0.01, and ***P < 0.001.

**Fig. S6 CAS is a newly ERS/AMPKα-dependent autophagy activator. A** Heat map depicting relative mRNA levels of ERS-related genes in MKN-45 cells treated with DMSO or CAS, as determined by RNA-seq. ERS, endoplasmic reticulum stress. **B** Relative mRNA levels of ERS-related genes in MKN-45 cells treated with DMSO or CAS (50 μM, 48 h), measured by RT-qPCR. **C** Representative TEM images showing ER morphology in GAM-AD tumor tissues (35 mg/kg) and MKN-45 cells treated with CAS (50 μM, 48 h). N, nucleus; blue circles indicate dilated ER vesicles. Scale bar = 5 μm. **D** Heat map of relative mRNA levels of genes related to the structural constituents of the cytoskeleton in MKN-45 cells treated with DMSO or CAS, analyzed by RNA-seq. **E** Relative mRNA levels of genes encoding structural constituents of the cytoskeleton in MKN-45 cells treated with DMSO or CAS (50 μM, 48 h), assessed by RT-qPCR. **F** Western blot analysis of AMPKα, p-AMPKα (Thr172), ULK1, p-ULK1 (Ser555), mTOR, p-mTOR (Ser2448), and LC3B levels in MKN-45 cells treated with DMSO or the indicated concentrations of CAS for 48 h. Relative protein expression levels were quantified based on band intensity. **G**-**I** Western blot analysis of LC3B levels in AMPKα- **(G)**, ULK1- (**H**), and PERK- (**I**) knockdown MKN-45 cells treated with DMSO or CAS (50 μM) for 48 h. Relative protein expression levels were quantified based on band intensity. **J** Western blot analysis of LC3B levels in AMPKα-knockdown MKN-45 cells treated with DMSO, CAS (50 μM) alone, or in combination with the IRE1α inhibitors STF-083010 (30 μM) or 4μ8C (3 μM) for 48 h. Relative protein expression levels were quantified based on band intensity. **K** Western blot analysis of PARP, cleaved-PARP, H2A.X, and γ-H2A.X levels in tumor tissues from orthotopic PDX models (GAM-AD and GAM-0125) treated with the indicated drugs. Relative protein expression levels were quantified based on band intensity. *P < 0.05, **P < 0.01, and ***P < 0.001.

**Fig.S7 CAS alleviates cisplatin-induced toxicity by activating AMPKα-mediated protective autophagy. A** Immunofluorescence staining showing the number of LC3B puncta in HK-2 and GES-1 cells treated with CAS (50 μM) and Cis (5 μM), either alone or in combination with 3-MA (5 mM) for 12 h. Scale bar = 20 μm. **B, C** Immunofluorescence staining showing the number of γ-H2A.X (**B**) and LC3B (**C**) puncta in AMPKα-knockdown HK-2 and GES-1 cells treated with CAS (50 μM) and Cis (5 μM), alone or in combination for 12 h. Scale bar = 20 μm. **D** Co-localization analysis of GFP-LC3B and LAMP1 puncta in GES-1 and MKN-45 cells treated with DMSO, Cis (5 μM), CAS (50 μM), or both agents for the indicated durations. Scale bar = 50 μm. **E** Western blot analysis of AMPKα, p-AMPKα (Thr172), p-ULK1 (Ser555), p-mTOR (Ser2448), and p-ULK1(Ser757) levels in MKN-45 cells treated with DMSO, Cis, CAS or Cis-CAS combination for 48 h. Relative protein expression levels were quantified based on band intensity. **F** Western blot analysis of AMPKα, p-AMPKα (Thr172), p-ULK1 (Ser555), p-mTOR (Ser2448), and p-ULK1(Ser757) levels in HK-2 and GES-1 cells treated with DMSO, Cis, CAS or Cis-CAS combination for 48 h. Relative protein expression levels were quantified based on band intensity. **G** Schematic diagram illustrating the dual regulatory mechanism of protective autophagy in normal tissues and destructive autophagy in tumor tissues established by combination therapy.

Table 4 The detailed monomer information of the NCL

| **Plant name** | | **Num.** | | **English name** | | **Purity** | |
| --- | --- | --- | --- | --- | --- | --- | --- |
| Glycyrrhiza uralensis Fisch | A0037 | | Glycyrrhizic acid | | HPLC≥98% | |  |
|  | A0038 | | Glycyrrhetinic acid | | HPLC≥98% | |  |
|  | A0039 | | Monoammoniumglycyrrhizinate | | HPLC≥98% | |  |
|  | A0040 | | Liquiritin | | HPLC≥98% | |  |
|  | A0041 | | Isoliquiritin | | HPLC≥98% | |  |
|  | A0042 | | Liquiritigenin | | HPLC≥98% | |  |
|  | A0559 | | Licochalcone B | | HPLC≥98% | |  |
|  | A0463 | | Isoliquiritigenin | | HPLC≥98% | |  |
|  | A0554 | | Echinatin | | HPLC≥98% | |  |
|  | A0558 | | Licochalcone A | | HPLC≥98% | |  |
|  | A0416 | | Glabridin | | HPLC≥98% | |  |
|  | A0560 | | Licochalcone C | | HPLC≥98% | |  |
|  | A1198 | | Neoisoliquiritin;  isoneoliquiritin | | HPLC≥98% | |  |
|  | A1197 | | Choerospondin；  5-hydroxyliquiritin | | HPLC≥98% | |  |
|  | A1203 | | neoliquiritin | | HPLC≥98% | |  |
|  | A1204 | | Glycyroside | | HPLC≥98% | |  |
|  | A1214 | | Isoliquiritin apioside | | HPLC≥98% | |  |
|  | A1215 | | liquiritin apioside | | HPLC≥98% | |  |
|  | A1216 | | licraside | | HPLC≥98% | |  |
|  | A1217 | | Liquiritigenin-7-O-apiosyl (1-2)-glucoside | | HPLC≥98% | |  |
|  | A1218 | | Licorice glycoside C2 | | HPLC≥98% | |  |
|  | A1227 | | Semilicoisoflavone B | | HPLC≥98% | |  |
|  | A1228 | | neoglycyrol | | HPLC≥98% | |  |
|  | A1229 | | Licoisoflavone A | | HPLC≥98% | |  |
|  | A1230 | | Licoisoflavone B | | HPLC≥98% | |  |
|  | A1231 | | Licoflavonol | | HPLC≥98% | |  |
| Aconitum carmichaeli Debx | A0196 | | Aconitine | | HPLC≥98% | |  |
|  | A0608 | | Mesaconitine | | HPLC≥98% | |  |
|  | A0609 | | Hypaconitine | | HPLC≥98% | |  |
|  | A0631 | | Benzoylaconine | | HPLC≥98% | |  |
|  | A0632 | | Benzoylmesaconine | | HPLC≥98% | |  |
|  | A0633 | | Benzoylhypacoitine | | HPLC≥98% | |  |
|  | A1090 | | Benzoylmesaconine-7-Palmitate | | HPLC≥98% | |  |
|  | A0824 | | Songorine | | HPLC≥99% | |  |
|  | A0403 | | Bulleyaconi cine A | | HPLC≥98% | |  |
|  | A0610 | | Indaconitine | | HPLC≥98% | |  |
|  | A0611 | | Yunaconitine | | HPLC≥98% | |  |
|  | A1102 | | 8-Deacetyl yunaconitine | | HPLC≥98% | |  |
|  | A0641 | | Aconine | | HPLC≥98% | |  |
|  | A0642 | | Mesaconine | | HPLC≥98% | |  |
|  | A0643 | | Hypaconine | | HPLC≥98% | |  |
|  | A0676 | | Lappaconitine | | Titration≥98% | |  |
|  | A0851 | | Karacoline | | HPLC≥98% | |  |
|  | A0793 | | Fuziline | | HPLC≥98% | |  |
|  | A0803 | | Crassicauline A | | HPLC≥98% | |  |
|  | A0896 | | Talatisamine | | HPLC≥98% | |  |
|  | A0897 | | Deltaline | | HPLC≥98% | |  |
|  | A0898 | | Delsoline | | HPLC≥98% | |  |
|  | A0899 | | Bullatine B;  Neoline | | HPLC≥98% | |  |
|  | A0957 | | 10-hydroxy mesaconitine | | HPLC≥96% | |  |
|  | A0900 | | 13-Dehydroxyindaconintine | | HPLC≥98% | |  |
| A.membranaceus(Fisch.) Bunge | A0069 | | Astragaloside | | ≥98% | |  |
|  | A0070 | | Astragaloside IV | | HPLC≥98% | |  |
|  | A0071 | | Astragaloside I | | HPLC≥98% | |  |
|  | A0072 | | Astragaloside II | | HPLC≥98% | |  |
|  | A0073 | | Astragaloside III | | HPLC≥95% | |  |
|  | A0875 | | Isoastragaloside I | | HPLC≥98% | |  |
|  | A0876 | | Isoastragaloside II | | HPLC≥98% | |  |
|  | A1081 | | Isoastragaloside IV | | HPLC≥98% | |  |
|  | A1110 | | 9-O-Methylnissolin 3-O-glucoside | | HPLC≥98% | |  |
|  | A1111 | | Astraisoflavan-7-O-β-D-glucoside | | HPLC≥98% | |  |
|  | A0877 | | β-D-Glucopyranoside, (3β,6α,16β,20R,24S)-3-[(3,4-di-O-acetyl-β-D-xylopyranosyl)oxy]-20, 24-epoxy-16,25-dihydroxy-9,19-cyclolanostan-6-yl | | HPLC≥97% | |  |
|  | A0878 | | Cyclocephaloside II | | HPLC≥98% | |  |
|  | A0232 | | Formononetin | | HPLC≥98% | |  |
|  | A0511 | | Ononin | | HPLC≥98% | |  |
|  | A0514 | | Calycosin | | HPLC≥98% | |  |
|  | A0515 | | Calycosin-7-glucoside | | HPLC≥98% | |  |
|  | A0550 | | Cycloastragenol | | HPLC≥98% | |  |
|  | A0535 | | Complanatuside | | HPLC≥98% | |  |
|  | A1427 | | polysachoride of Astragalus mongholicus | | UV≥95% | |  |
| Momordica grosvenori Swingle | A0225 | | Mogroside V | | HPLC≥98% | |  |
|  | A0840 | | Mogroside IVa | | HPLC≥98% | |  |
|  | A0841 | | Isomogroside V | | HPLC≥98% | |  |
|  | A1179 | | Mogroside IIA | | HPLC≥96% | |  |
|  | A1365 | | Mogroside IA | | HPLC≥98% | |  |
|  | A0843 | | Mogroside IIIA1 | | HPLC≥98% | |  |
|  | A1166 | | Mogroside IIA1 | | HPLC≥98% | |  |
|  | A1155 | | Mogroside IIIA2 | | HPLC≥98% | |  |
|  | A0844 | | Mogroside IIA2 | | HPLC≥97% | |  |
|  | A0845 | | Mogroside IIIe | | HPLC≥98% | |  |
|  | A1174 | | Mogroside IIe | | HPLC≥98% | |  |
|  | A0846 | | Grosvenorine | | HPLC≥98% | |  |
|  | A0711 | | Mogroside IV | | HPLC≥98% | |  |
|  | A0712 | | 11-oxo-mogroside V | | HPLC≥98% | |  |
|  | A0713 | | Siamenoside I | | HPLC≥98% | |  |
|  | A0772 | | Mogroside VI | | HPLC≥97% | |  |
|  | A1177 | | Mogroside VI A | | HPLC≥97% | |  |
|  | A1178 | | Mogroside VI B | | HPLC≥98% | |  |
|  | A0773 | | Mogroside III | | HPLC≥98% | |  |
|  | A1167 | | 11-deoxymorgroside V | | HPLC≥95% | |  |
|  | A1173 | | 11-epi-morgroside V | | HPLC≥98% | |  |
|  | A1175 | | 11-O-Siamenoside I | | HPLC≥94% | |  |
|  | A1176 | | 11-oxo-morgroside VI | | HPLC≥96% | |  |
|  | A1273 | | 11-O-Mogroside IIIA1 | | HPLC≥95% | |  |
|  | A1281 | | 7β-Methoxy-Mogroside V | | HPLC≥94% | |  |
|  | A1327 | | 11-Oxomogroside III | | HPLC≥98% | |  |
|  | A1328 | | 11-Oxomogroside II A1 | | HPLC≥98% | |  |
|  | A1329 | | 11-Oxomogroside II A2 | | HPLC≥97% | |  |
|  | A1330 | | 11-Oxomogroside IVa | | HPLC≥97% | |  |
|  | A1356 | | Mogroside IE | | HPLC≥98% | |  |
|  | A1364 | | Mogrol | | HPLC≥98% | |  |
| Punica granatum L. | A0226 | | Punicalagin | | HPLC≥98% | |  |
|  | A0293 | | Punicalin | | HPLC≥98% | |  |
| Panax ginseng C. A. Mey. | A0233 | | Pseudoginsenoside- F_11_ | | HPLC≥98% | |  |
|  | A0234 | | Ginsenoside Rb1 | | HPLC≥98% | |  |
|  | A0235 | | Ginsenoside Rb2 | | HPLC≥98% | |  |
|  | A0236 | | Ginsenoside Rb3 | | HPLC≥98% | |  |
|  | A0237 | | Ginsenoside Rg1 | | HPLC≥98% | |  |
|  | A0238 | | Ginsenoside Rg2 | | HPLC≥98% | |  |
|  | A0239 | | Ginsenoside Rg3 | | HPLC≥98% | |  |
|  | A0240 | | Ginsenoside Rh1 | | HPLC≥98% | |  |
|  | A0241 | | Ginsenoside Rh2 | | HPLC≥98% | |  |
|  | A0904 | | Pseudoginsenoside Rh2 | | HPLC≥98% | |  |
|  | A0242 | | Ginsenoside Rh3 | | HPLC≥98% | |  |
|  | A0243 | | Ginsenoside Rc | | HPLC≥98% | |  |
|  | A0244 | | Ginsenoside Re | | HPLC≥98% | |  |
|  | A0245 | | Ginsenoside Rd | | HPLC≥98% | |  |
|  | A1006 | | Ginsenoside Rd2 | | HPLC≥98% | |  |
|  | A1004 | | 5,6-Dehydrogensenoside Rd | | HPLC≥98% | |  |
|  | A0246 | | Ginsenoside- Rf | | HPLC≥98% | |  |
|  | A0247 | | Pseudoginsenoside-RT5 | | HPLC≥98% | |  |
|  | A0249 | | Protopanaxatriol | | HPLC≥98% | |  |
|  | A0313 | | Ginsenoside Rh1 | | HPLC≥98% | |  |
|  | A0328 | | Ginsenoside Rh2 | | HPLC≥96% | |  |
|  | A0999 | | Ginsenoside Rh4 | | HPLC≥98%） | |  |
|  | A1003 | | Gensenoside Rh7 | | HPLC≥96% | |  |
|  | A1011 | | Ginsenoside Rh8 | | HPLC≥98% | |  |
|  | A0350 | | Ginsenoside Rg2 | | HPLC≥97% | |  |
|  | A0359 | | Ginsenoside Rg3 | | HPLC≥98% | |  |
|  | A0360 | | Protopanaxtriol | | HPLC≥98% | |  |
|  | A0448 | | Ginsenoside F1 | | HPLC≥98% | |  |
|  | A0449 | | Ginsenoside F2 | | HPLC≥98% | |  |
|  | A0648 | | Ginsenoside F3 | | HPLC≥98% | |  |
|  | A0991 | | Ginsenoside F4 | | HPLC≥98% | |  |
|  | A0992 | | Ginsenoside F5 | | HPLC≥98% | |  |
|  | A0450 | | Compound K | | HPLC≥98% | |  |
|  | A0460 | | Protopanaxdiol | | HPLC≥95% | |  |
|  | A0522 | | Ginsenoside Ro | | HPLC≥98% | |  |
|  | A0994 | | Ginsenoside Rg5 | | HPLC≥98% | |  |
|  | A0774 | | Ginsenoside Rg6 | | HPLC≥90% | |  |
|  | A0775 | | Ginsenoside Rk1 | | HPLC≥98% | |  |
|  | A1015 | | Ginsenoside Rk2 | | HPLC≥98% | |  |
|  | A1016 | | Ginsenoside Rk3 | | HPLC≥90% | |  |
|  | A0250 | | Panaxadiol | | HPLC≥98% | |  |
|  | A0251 | | Panaxtriol | | HPLC≥98% | |  |
|  | A0248 | | Protopanaxdiol | | HPLC≥98% | |  |
|  | A0997 | | Notoginsenoside Fe | | HPLC≥98% | |  |
|  | A1005 | | Vina-ginsenoside R3 | | HPLC≥94% | |  |
|  | A1007 | | Vina-ginsenoside R4 | | HPLC≥98% | |  |
|  | A1008 | | Vina-ginsenoside R8 | | HPLC≥98% | |  |
|  | A1025 | | Ginsenoside Ra1 | | HPLC≥98% | |  |
|  | A1027 | | Ginsenoside Ra2 | | HPLC≥95% | |  |
|  | A1040 | | Ginsenoside Ra3 | | HPLC≥98% | |  |
|  | A1363 | | Ginsenoside Ra6 | | HPLC≥98% | |  |
|  | A1038 | | Panasenoside;Kaempferol-3-O-glucosyl（1-2）galactoside | | HPLC≥92% | |  |
|  | A1362 | | Ginsenoside Rs1 | | HPLC≥98% | |  |
|  | A1340 | | Ginsenoside Rs2 | | HPLC≥98% | |  |
|  | A1343 | | Quinquenoside R1 | | HPLC≥88% | |  |
|  | A1361 | | 20-gluco-ginsenoside Rf | | HPLC≥98% | |  |
| Bupleurum chinense DC. | A0257 | | Saikosaponin A | | HPLC≥98% | |  |
|  | A0258 | | Saikosaponin C | | HPLC≥98% | |  |
|  | A0259 | | Saikosaponin D | | HPLC≥98% | |  |
|  | A0260 | | Saikosaponin B1 | | HPLC≥98% | |  |
|  | A0261 | | Saikosaponin B2 | | HPLC≥98% | |  |
|  | A1054 | | Saikosaponin B3 | | HPLC≥98% | |  |
|  | A1055 | | Saikosaponin B4 | | HPLC≥97% | |  |
|  | A1062 | | Saikosaponin F | | HPLC≥98% | |  |
|  | A1063 | | Saikosaponin H | | HPLC≥98% | |  |
|  | A1064 | | Saikosaponin I | | HPLC≥97% | |  |
|  | A1065 | | Saikosaponin G | | HPLC≥98% | |  |
|  | A1066 | | 11(α)-methoxysaikosaponin F | | HPLC≥98% | |  |
|  | A0442 | | Kaempferitrin;Lespedin; Lespenephryl | | HPLC≥98% | |  |
|  | A1272 | | Tibesaikosaponin V | | HPLC≥90% | |  |
| Panax Notoginseng | A0273 | | Notoginsenoside R1 | | HPLC≥98% | |  |
|  | A0634 | | Notoginsenoside Ft1 | | HPLC≥98% | |  |
|  | A0635 | | 20(R)-Notoginsenoside R2 | | HPLC≥98% | |  |
|  | A0857 | | 20(S)-NotoginsenosideR2 | | HPLC≥98% | |  |
|  | A1094 | | Notoginsenoside Fa | | HPLC≥96% | |  |
|  | A0760 | |  | | Standard | |  |
|  | A1222 | | Notoginsenoside Fd | | HPLC≥97% | |  |
| Alisma plantag-oaquatica L. var. orientale Samuels. | A0865 | | Alisol A | | HPLC≥98% | |  |
|  | A0882 | | Alisol B | | HPLC≥98% | |  |
|  | A0861 | | Alisol A 24-acetate | | HPLC≥98% | |  |
|  | A0885 | | 25-Methoxyalisol A | | HPLC≥98% | |  |
|  | A0446 | | Alisol B 23-acetate | | HPLC≥98% | |  |
|  | A0873 | | 23-Acetyl alisol C | | HPLC≥98% | |  |
|  | A0880 | | Alisol F | | HPLC≥98% | |  |
|  | A0881 | | Alisol G | | HPLC≥98% | |  |
|  | A0883 | | Alisol F 24-acetate | | HPLC≥97% | |  |
|  | A0480 | | Alismoxide | | HPLC≥98% | |  |
|  | A1164 | | 11-Deoxyalisol B | | HPLC≥98% | |  |
| Pulsatilla chinensis (Bunge) Regel. | A0742 | | α-Hederin | | HPLC≥98% | |  |
|  | A0825 | | Pulchinenoside A3 | | HPLC≥98% | |  |
|  | A0492 | | Anemoside B4 | | HPLC≥98% | |  |
|  | A0833 | | Hederacoside C | | HPLC≥98% | |  |
|  | A0838 | | Pulchinenoside B | | HPLC≥98% | |  |
|  | A0839 | | 3-O-D-glucopyranosyl( 1→4)- [ L-rhamnopyranosyl(1→2)]-L-arabinopyranosyl 23-hydroxyl lup-20(29)-en-28-oic acid – 28-O-rhamnopyranosyl(1→4)glucopyranosyl(1→6)glucopyranoside | | HPLC≥98% | |  |
|  | A0853 | | Hederagenin - 3-O-β-D- Galactosyl( 1→3)-β-D-glucosyl( 1→3)-α-L-rhamnosyl(1→2)-α-L-arabinoside | | HPLC≥98% | |  |
|  | A0854 | | Oleanolic acid - 3-O-β-D- Galactosyl( 1→3)-β-D-glucosyl( 1→3)-α-L-rhamnosyl(1→2)-α-L-arabinoside | | HPLC≥98% | |  |
|  | A0855 | | 3-O-β-D- Galactosyl( 1→3)-β-D-glucosyl( 1→3)-α-L-rhamnosyl(1→2)-α-L-arabinosyl-Oleanolic acid - 28-O-α-L-rhamnosyl(1→4)β-D-glucosyl( 1→6)-β-D-glucoside | | HPLC≥98% | |  |
|  | A0966 | | Oleanolic acid - 3-O-α-L-rhamnosyl(1→6)β-D- Galactosyl( 1→3)-β-D-glucosyl( 1→3)-α-L-rhamnosyl(1→2)-α-L-arabinoside | | HPLC≥98% | |  |
|  | A0829 | | Kalopanaxsaponin H | | HPLC≥98% | |  |
|  | A0828 | | Hederacolchiside A1 | | HPLC≥98% | |  |
|  | A0834 | | Hederacolchiside E | | HPLC≥98% | |  |
|  | A0832 | | Cussosaponin C | | HPLC≥98% | |  |
|  | A0827 | | Oleanolic acid 3-O-β-D-glucosyl-( 1→3)-α-L-ramnosyl(1→2)-α-L-arabinoside | | HPLC≥98% | |  |
|  | A0830 | | Lup-20(29)-en-28-oic acid, 3-[ D-glucopyranosyl(1→4)[ L-rhamnopyranosyl) (1→2)-L-arabinopyranosyl]oxy], (3,4)-) | | HPLC≥98% | |  |
|  | A0831 | | Hederagenin 3-O-α-L-rhamnopyranosyl(1→2)-(β-D-glucopyranosyl(1→4))-α-L-arabinopyranoside | | HPLC≥98% | |  |
|  | A0835 | | 3-O-D-glucopyranosyl( 1→3)-L-rhamnopyranosyl(1→2)-L-arabinopyranosyl Oleanolic acid – 28-O-rhamnopyranosyl(1→4)glucopyranosyl(1→6)glucopyranoside | | HPLC≥98% | |  |
|  | A0836 | | 3-O-D-glucopyranosyl( 1→3)-L-rhamnopyranosyl(1→2)-L-arabinopyranosyl lupinic acid– 28-O-rhamnopyranosyl(1→4)glucopyranosyl(1→6)glucopyranoside | | HPLC≥98% | |  |
|  | A0837 | | 3-O-D-glucopyranosyl( 1→4)- [ L-rhamnopyranosyl(1→2)]-L-arabinopyranosyl 23-hydroxyl lup-20(29)-en-28-oic acid – 28-O-rhamnopyranosyl(1→4)glucopyranosyl(1→6)glucopyranoside | | HPLC≥98% | |  |
|  | A0826 | | Scabioside C | | HPLC≥98% | |  |
| Zingiber officinale Roscoe | A0218 | | 6-gingerol | | HPLC≥98% | |  |
|  | A0505 | | 8-Gingerol | | HPLC≥98% | |  |
|  | A0506 | | 10-Gingerol | | HPLC≥98% | |  |
|  | A0541 | | 6-Shogaol | | HPLC≥98% | |  |
|  | A0874 | | 8-Shogaol | | HPLC≥97% | |  |
|  | A0587 | | Zingerone | | HPLC≥98% | |  |
|  | A1445 | | Diacetoxy-6-gingerdiol | | HPLC≥98% | |  |
|  | A1446 | | Paradol | | HPLC≥98% | |  |
| (Ophiopogon japonicus(L•f•)Ker-Gawl•) | A0852 | | Methylophiopogonanone A | | HPLC≥98% | |  |
|  | A1180 | | Methylophiopogonanone B | | HPLC≥98% | |  |
|  | A0404 | | Liriopesides B | | HPLC≥98% | |  |
|  | A0684 | | Liriope muscari baily saponins C | | HPLC≥98% | |  |
|  | A0540 | | Ruscogenin | | HPLC≥98% | |  |
|  | A0343 | | Ophiopogonin D | | HPLC≥98% | |  |
|  | A0863 | | Ophiopojaponin C | | HPLC≥98% | |  |
|  | A0866 | | Saponin V；  Ophiogenin-3-O-α-L-rhamnosyl-(1→2)-β-D-glucoside | | HPLC≥98% | |  |
|  | A0867 | | Diosgenin-3-O-β-D-xylosyl-(1→3)-β-glucoside | | HPLC≥98% | |  |
|  | A0868 | | Deacetyl ophiopojaponin A | | HPLC≥98% | |  |
|  | A0869 | | 14α-hydroxy Sprengerinin C;  Saponin U | | HPLC≥98% | |  |
|  | A0871 | | Methylophiopogonone A | | HPLC≥98% | |  |
|  | A0870 | | Glycoside O-4 | | HPLC≥98% | |  |
|  | A0864 | | Ophiopogonin D' | | HPLC≥96% | |  |
|  | A1189 | | Ophiopogonanone C | | HPLC≥98% | |  |
|  | A1190 | | Ophiopogonanone D | | HPLC≥98% | |  |
|  | A1191 | | 8-formyl ophiopogonanone B | | HPLC≥98% | |  |
|  | A1192 | | 6-aldehydo-isoophiopogonone A | | HPLC≥98% | |  |
|  | A1195 | | 8-formyl ophiopogonone B | | HPLC≥98% | |  |
|  | A1199 | | ophiopogonone C | | HPLC≥98% | |  |
|  | A1206 | | Liriopesides C | | HPLC≥98% | |  |
|  | A1207 | | Dracaenoside F | | HPLC≥98% | |  |
|  | A1384 | | D-Mannose | | HPLC≥98% | |  |
|  | A1392 | | Triglochinic acid | | HPLC≥98% | |  |
| (Savia miltiorrhiza) | A0055 | | Salvianolic acid A | | HPLC≥98% | |  |
|  | A0056 | | Salvianolic acid B | | HPLC≥98% | |  |
|  | A0892 | | Isosalvianolic acid B | | HPLC≥98% | |  |
|  | A0623 | | Salvianolic acid C | | HPLC≥98% | |  |
|  | A0624 | | Salvianolic acid D | | HPLC≥96%） | |  |
|  | A0057 | | Tanshinone IIA | | HPLC≥98% | |  |
|  | A0058 | | Tanshinone IIA-sulfonic sodium | | HPLC≥92% | |  |
|  | A0059 | | Tanshinone I | | HPLC≥98% | |  |
|  | A0060 | | Dihydrotanshinone I | | HPLC≥98% | |  |
|  | A0061 | | Cryptotanshinone | | HPLC≥98% | |  |
|  | A0062 | | Sodium Danshensu | | HPLC≥98% | |  |
|  | A0551 | | Danshensu | | HPLC≥98% | |  |
|  | A0887 | | Dimethyl lithospermate B | | HPLC≥90% | |  |
|  | A0888 | | 9''-methyl lithospermate B | | HPLC≥98% | |  |
|  | A0889 | | \|  \| 9’’’-Methyllithospermate B \| \| --- \| --- \| | | HPLC≥98% | |  |
| Glycinemax(L.)merr | A0005 | | Glycitin | | HPLC≥98% | |  |
|  | A0006 | | Glycitein | | HPLC≥98% | |  |
|  | A0007 | | Daidzin | | HPLC≥98% | |  |
|  | A0008 | | Daidzein | | HPLC≥98% | |  |
|  | A0009 | | Genistein | | HPLC≥98% | |  |
|  | A0010 | | Genistin | | HPLC≥98% | |  |
|  | A0890 | | Soyosaponin Aa | | HPLC≥98% | |  |
|  | A0886 | | Soyosaponin Ab | | HPLC≥98% | |  |
|  | A0905 | | Soyosaponin Ac | | HPLC≥98% | |  |
|  | A1323 | | Soyasaponin Ae | | HPLC≥95% | |  |
|  | A1324 | | Soyasaponin Af | | HPLC≥98% | |  |
|  | A0715 | | Soyasaponin Ba | | HPLC≥98% | |  |
|  | A0714 | | Soyasaponin Bb | | HPLC≥98% | |  |
|  | A0948 | | Soyasaponin Bd | | HPLC≥98% | |  |
|  | A0949 | | Soyasaponin Be | | HPLC≥98% | |  |
|  | A0950 | | Soyasaponin Be methyl ester | | HPLC≥98% | |  |
| Brassica campestris L. | A0884 | | Campesterol | | HPLC≥98% | |  |
|  | A0510 | | Stigmasterol | | HPLC≥95% | |  |
|  | A0197 | | β-Sitosterol | | HPLC≥98% | |  |
| Szechwan Chinaberry fruit | A0187 | | Toosendanin | | HPLC≥98% | |  |
|  | A0976 | | Trichilinin D | | HPLC≥98% | |  |
|  | A0977 | | 1-Cinnamoyltrichilinin | | HPLC≥98% | |  |
|  | A0978 | | Trichilinin | | HPLC≥98% | |  |
|  | A0979 | | 1-Acetyltrichilinin | | HPLC≥98% | |  |
|  | A0980 | | 1-Tigloyltrichilinin | | HPLC≥98% | |  |
|  | A0981 | | Volkensin | | HPLC≥98% | |  |
|  | A0982 | | 1-Deacetylnimbolinin B | | HPLC≥98% | |  |
|  | A0983 | | 12-Ethoxynimbolinin C | | HPLC≥98% | |  |
|  | A0984 | | 12-Ethoxynimbolinin B | | HPLC≥98% | |  |
|  | A0985 | | Salannal | | HPLC≥98% | |  |
|  | A0986 | | 12-Acetyltrichilin B | | HPLC≥98% | |  |
|  | A0987 | | 7,14-Epoxymeliatoxin A1 | | HPLC≥98% | |  |
|  | A0988 | | 12-O-Acetylazedarachin A | | HPLC≥98% | |  |
|  | A0989 | | Spirosendan | | HPLC≥98% | |  |
| Lonicera Japonica | A0022 | | Chlorogenic acid | | HPLC≥98% | |  |
|  | A0023 | | Neochlorogenic acid | | HPLC≥98% | |  |
|  | A0024 | | Cryptochlorogenic acid | | HPLC≥99% | |  |
|  | A0025 | | Isochlorogenic acid A | | HPLC≥98% | |  |
|  | A0026 | | Isochlorogenic acid B | | HPLC≥98% | |  |
|  | A0027 | | Isochlorogenic acid C | | HPLC≥98% | |  |
|  | A0029 | | Cynarin | | HPLC≥98% | |  |
|  | A0030 | | 1,5-Dicaffeoylquinic acid | | HPLC≥98% | |  |
|  | A0109 | | Cynaroside | | HPLC≥98% | |  |
| Polygala tenuifolia Willd. | A0013 | | Polygalacic acid | | HPLC≥99% | |  |
|  | A0014 | | Senegenin | | HPLC≥99% | |  |
|  | A0345 | | Tenuifolin | | HPLC≥98% | |  |
|  | A0776 | | 3,6′-Disinapoyl sucrose | | HPLC≥98% | |  |
|  | A0821 | | Onjisaponin B | | HPLC≥98% | |  |
|  | A0622 | | Polygalaxanthone III | | HPLC≥98% | |  |
|  | A1071 | | Sibiricaxanthone B | | HPLC≥98% | |  |
|  | A0968 | | Sibiricose A5 | | HPLC≥93% | |  |
|  | A0969 | | Sibiricose A6 | | HPLC≥96% | |  |
|  | A1209 | | Tenuifoliside A | | HPLC≥98% | |  |
|  | A1210 | | Tenuifoliside B | | HPLC≥97% | |  |
|  | A0973 | | Tenuifoliside C | | HPLC≥98% | |  |
|  | A1061 | | 2'-cinnamoyl-3'-benzoyl-(2-O-α-glucosyl)-Sucrose | | HPLC≥98% | |  |
|  | A1080 | | 7-O-methyl mangiferin | | HPLC≥98% | |  |
|  | A1212 | | Arillanin A | | HPLC≥98% | |  |
|  | A1213 | | Glomeratose A | | HPLC≥97% | |  |
|  | A1368 | | 1,5-Anhydro-D-sorbitol | | HPLC≥98% | |  |
|  | A1372 | | Sinapic acid | | HPLC≥98% | |  |
|  | A1405 | | Tenuifoliose F | | HPLC≥98% | |  |
| Tripterygium wilfordii Hook. f. | A0104 | | Triptolide | | HPLC≥98% | |  |
|  | A0105 | | Tripdiolide | | HPLC≥98% | |  |
|  | A0106 | | Celastrol | | HPLC≥98% | |  |
|  | A0107 | | Wilforlide A | | HPLC≥98% | |  |
|  | A0508 | | Triptonide | | HPLC≥98% | |  |
|  | A0652 | | Demethylzeylasteral | | HPLC≥98% | |  |
|  | A0819 | | Triptophenolide | | HPLC≥98% | |  |
|  | A1398 | | Wilforine | | HPLC≥98% | |  |
|  | A1399 | | Wilforgine | | HPLC≥98% | |  |
|  | A1400 | | Tripterifordin | | HPLC≥98% | |  |
|  | A1441 | | Orthosphenic acid | | HPLC≥98% | |  |
| Epimedium brevicornum Maxim. | A0145 | | Icariin | | HPLC≥98% | |  |
|  | A0228 | | Epimedin A | | HPLC≥98% | |  |
|  | A0229 | | Epimedin B | | HPLC≥98% | |  |
|  | A0230 | | Epimedin C | | HPLC≥98% | |  |
|  | A0518 | | Epimedin A1 | | HPLC≥98% | |  |
|  | A0637 | | Baohuoside I | | HPLC≥98% | |  |
|  | A0951 | | Epimedoside A | | HPLC≥94% | |  |
|  | A0995 | | Icariside I | | HPLC≥98% | |  |
|  | A1261 | | Epimedin I | | HPLC≥98% | |  |
|  | A1265 | | hydroxyl icariin | | HPLC≥98% | |  |
|  | A1271 | | Epimedoside | | HPLC≥98% | |  |
|  | A1391 | | Icaritin | | HPLC≥95% | |  |
| Tea | A0158 | | (+)-Catechin Hydrate; | | HPLC≥98% | |  |
|  | A0771 | | EC;  Epicatechin | | HPLC≥98% | |  |
|  | A0159 | | EGCG | | HPLC≥98% | |  |
|  | A0160 | | ECG | | HPLC≥98% | |  |
|  | A0161 | | EGC | | HPLC≥98% | |  |
|  | A0162 | | GCG | | HPLC≥98% | |  |
|  | A0891 | | CG | | HPLC≥98% | |  |
|  | A0471 | | Theophylline | | HPLC≥98% | |  |
|  | A0512 | | GC;  (−)-Gallocatechin | | HPLC≥98% | |  |
|  | A0954 | | Theaflavin | | HPLC≥98% | |  |
|  | A0789 | | L-Theanine | | HPLC≥98% | |  |
|  | A0470 | | Caffeine | | HPLC≥98% | |  |
|  | A1374 | | Catechol | | HPLC≥98% | |  |
|  | A1421 | | Theaflavin-3-gallate | | HPLC≥98% | |  |
|  | A1422 | | Theaflavin-3'-gallate | | HPLC≥98% | |  |
|  | A1423 | | Theaflavin digallate | | HPLC≥98% | |  |
| Anemarrhena asphadeloides Bge | A0727 | | Isomangiferin | | HPLC≥98% | |  |
|  | A0338 | | Timosaponin BII | | HPLC≥98% | |  |
|  | A0339 | | Timosaponin A3 | | HPLC≥98% | |  |
|  | A0544 | | Anemarsaponin BIII | | HPLC≥96% | |  |
|  | A0545 | | Anemarsaponin E | | HPLC≥98% | |  |
|  | A0936 | | Timosaponin C | | HPLC≥97% | |  |
|  | A0895 | | Officinalisinin I | | HPLC≥98% | |  |
|  | A1104 | | Anemarrhenasaponin I | | HPLC≥98% | |  |
|  | A1105 | | Anemarrhenasaponin Ia | | HPLC≥98% | |  |
|  | A1106 | | Anemarrhenasaponin III | | HPLC≥98% | |  |
|  | A1107 | | Anemarrhenasaponin A2 | | HPLC≥98% | |  |
|  | A1132 | | Timosaponin A1 | | HPLC≥98% | |  |
| Stevia rebaudiana | A0137 | | Stevioside | | HPLC≥98% | |  |
|  | A0015 | | Rebaudioside A | | HPLC≥98% | |  |
|  | A1419 | | Rebaudioside B | | HPLC≥98% | |  |
|  | A0645 | | Rebaudioside C | | HPLC≥98% | |  |
|  | A0646 | | Rebaudioside D | | HPLC≥98% | |  |
|  | A1282 | | Rebaudioside G | | HPLC≥97% | |  |
|  | A1283 | | Rebaudioside F | | HPLC≥98% | |  |
|  | A1284 | | Sterebin E | | HPLC≥95% | |  |
|  | A1385 | | D-Galactose | | HPLC≥98% | |  |
|  | A1420 | | Steviolbioside | | HPLC≥98% | |  |
| Nelumbo Nucifera Gaertn | A0418 | | Nuciferine | | HPLC≥98% | |  |
|  | A0561 | | Liensinine Perchlorate | | HPLC≥98% | |  |
|  | A0562 | | Liensinine | | HPLC≥98% | |  |
|  | A0572 | | Neferine | | HPLC≥98% | |  |
|  | A0557 | | Isoliensinine | | HPLC≥98% | |  |
|  | A1002 | | Armepavine | | HPLC≥98% | |  |
|  | A1112 | | O-demethyl nuciferine | | HPLC≥98% | |  |
|  | A1113 | | Quercetin-3-O-β-D-ribosyl-(1→2)-β-D-glucoside | | For identification purposes only | |  |
|  | A1114 | | 7-Megastigmene-3,5,6,9-tetrol | | HPLC≥98% | |  |
|  | A1424 | | N-Nornuciferine | | HPLC≥98% | |  |
| Ginkgo biloba L. | A0163 | | Ginkgolide A | | HPLC≥98% | |  |
|  | A0164 | | Ginkgolide B | | HPLC≥98% | |  |
|  | A0165 | | Ginkgolide C | | HPLC≥98% | |  |
|  | A0166 | | Bilobalide | | HPLC≥98% | |  |
|  | A0524 | | Ginkgolide J | | HPLC≥98% | |  |
|  | A0499 | | Kaempferol-3-O-rutinoside | | HPLC≥98% | |  |
|  | A0476 | | Narcissoside; Isorhamnetin-3-O-β-D-rutinoside | | HPLC≥98% | |  |
|  | A0465 | | Apigenin-7-glucoside | | HPLC≥98% | |  |
|  | A0679 | | Kaempferol-7-O-β-D-glucopyranoside | | HPLC≥96% | |  |
|  | A0928 | | 3-O-{2-O-[6-O-(p-hydroxyl-E-coumaroyl)-glucosyl]-(1-2)rhamnosyl kaempferol | | HPLC≥98% | |  |
|  | A0929 | | Quercetin-3-O-D-glucosyl]-(1-2)-L-rhamnoside | | HPLC≥98% | |  |
|  | A0930 | | Quercetin-3-O-[2-O-(6-O-p-hydroxyl-E-coumaroyl)-D-glucosyl]-(1-2)-L-rhamnoside | | HPLC≥98% | |  |
|  | A0933 | | Clitorin | | HPLC≥98% | |  |
|  | A1367 | | Kaempferol-3-O-β-D-glucosyl(1-2)rhamnoside | | HPLC≥98% | |  |
|  | A1370 | | Quercetin 3-O-rhamnosyl(1→2)[rhamnosyl-[(1→6)]glucoside | | HPLC≥98% | |  |
| Lithosperraum erythrorhizon Sieb. et Zucc | A0186 | | Shikonin | | HPLC≥98% | |  |
|  | A0848 | | Shikonofuran A | | HPLC≥98% | |  |
|  | A0486 | | Lithospermoside | | HPLC≥98% | |  |
|  | A0690 | | Lithospermic acid | | HPLC≥95% | |  |
|  | A1085 | | β,β-Dimethylacrylshikonin | | HPLC≥98% | |  |
|  | A1200 | | β-acetoxy isovaleryl alkannin | | HPLC≥98% | |  |
|  | A1201 | | isobutyryl alkannin | | HPLC≥98% | |  |
|  | A1202 | | acetyl alkannin | | HPLC≥98% | |  |
| Schisandra chinensis (Turcz.)Baill. | A0203 | | Schizandrin A | | HPLC≥98% | |  |
|  | A0204 | | Schizandrin B | | HPLC≥98% | |  |
|  | A0205 | | Schisandrol A | | HPLC≥98% | |  |
|  | A0206 | | Schisantherin B | | HPLC≥98% | |  |
|  | A0207 | | Schisantherin A | | HPLC≥98% | |  |
|  | A0208 | | Schisandrol B | | HPLC≥98% | |  |
|  | A0593 | | Schisandrin C | | HPLC≥98% | |  |
|  | A0823 | | Schisanhenol | | HPLC≥98% | |  |
|  | A0958 | | Schisanhenol B | | HPLC≥98% | |  |
|  | A0959 | | (-)Gomisin L1 | | HPLC≥98% | |  |
|  | A0960 | | (+)Gomisin M2 | | HPLC≥98% | |  |
|  | A0926 | | Gomisin N | | HPLC≥98% | |  |
|  | A0941 | | Gomisin J | | HPLC≥98% | |  |
|  | A0944 | | Gomisin G | | HPLC≥98% | |  |
|  | A0998 | | Gomisin D | | HPLC≥98% | |  |
|  | A0945 | | β-chamigrenic acid | | HPLC≥98% | |  |
|  | A0946 | | Chamigrenal | |  | |  |
|  | A0953 | | Angeloyl gomisin H | | HPLC≥98% | |  |
|  | A1087 | | Anwuligan | | HPLC≥98% | |  |
| Fritillaria | A0262 | | Tubeimoside I | | HPLC≥98% | |  |
|  | A0270 | | Peimine | | HPLC≥99% | |  |
|  | A0271 | | Peiminine | | HPLC≥99% | |  |
|  | A0331 | | Peimisine | | HPLC≥98% | |  |
|  | A0963 | | Hapepunine | | HPLC≥98% | |  |
|  | A0410 | | Hupehenine | | HPLC≥98% | |  |
|  | A0974 | | Yubeinine | | HPLC≥98% | |  |
|  | A0975 | | Ebeiedinone | | HPLC≥98% | |  |
|  | A0993 | | Frititorine A | | HPLC≥98% | |  |
|  | A1153 | | Zhebeirine | | HPLC≥98% | |  |
|  | A0964 | | Delavinone;  Sinpeimine | | HPLC≥98% | |  |
|  | A0598 | | Sipeimine | | HPLC≥98% | |  |
|  | A0691 | | Imperialine-D-glucoside | | HPLC≥98% | |  |
|  | A1184 | | Adenosine | | HPLC≥98% | |  |
| Olea europaea | A0001 | | Oleuropein | | HPLC≥98% | |  |
|  | A0312 | | Tyrosol | | HPLC≥98% | |  |
|  | A0693 | | Hydroxytyrosol | | HPLC≥98% | |  |
| Uncaria rhynchopylla (Miq.) Jacks. | A0318 | | Rhynchophylline | | HPLC≥98% | |  |
|  | A0319 | | Isorhychophylline | | HPLC≥98% | |  |
|  | A0341 | | Corynoxeine | | HPLC≥98% | |  |
|  | A0342 | | Isocorynoxeine | | HPLC≥98% | |  |
|  | A1060 | | Geissoschizine methyl ether | | HPLC≥98% | |  |
|  | A1067 | | Hirsutine | | HPLC≥98% | |  |
|  | A1068 | | Hirsuteine | | HPLC≥98% | |  |
| Crotalaria mucronata | A0322 | | Crotaline | | HPLC≥99% | |  |
|  | A0847 | | Usaramine | | HPLC≥98% | |  |
| Scrophularia ningpoensis Hemsl. | A0334 | | Harpagoside | | HPLC≥98% | |  |
|  | A0335 | | harpgide | | HPLC≥98% | |  |
|  | A1026 | | Sibirioside A | | HPLC≥98% | |  |
|  | A1036 | | 6’-O-cinnamoyl harpagide | | HPLC≥98% | |  |
|  |  |  |  |  |  |  |  |
|  |  |  |  |  |  |  |  |
|  | A0336 | | Angoroside C | | HPLC≥98% | |  |
|  | A1208 | | Picfeltarraenin IA | | HPLC≥98% | |  |
|  | A0370 | | Picfeltarraenin IB | | HPLC≥98% | |  |
| Atractylodes macrocephala Koidz | A0372 | | Atractylenolide I | | HPLC≥98% | |  |
|  | A0373 | | Atractylenolide II | | HPLC≥98% | |  |
|  | A0374 | | Atractylenolide III | | HPLC≥98% | |  |
| Atractylodes lancea( Thunb.)DC. | A1127 | | Taraxeryl acetate | | HPLC≥98% | |  |
|  | A1126 | | Tractylodinol acetate | | HPLC≥98% | |  |
|  | A1143 | | (4E,6E,12E)-tetradecatriene -8,10-diyne-1,3-diol diacetate | | HPLC≥98% | |  |
|  | A0547 | | Atractylodin | | HPLC≥98% | |  |
|  | A1136 | | Atractylone | |  | |  |
| Thalictrum dasycarpum Fisch.et Hall. | A0564 | | Magnoflorine | | HPLC≥98% | |  |
|  | A0764 | | Magnoflorine chloride | | HPLC≥98% | |  |
|  | A0607 | | Magnoflorine iodide | | HPLC≥98% | |  |
| Marsdenia tenacissima (Roxb.) Wight et Arn | A0568 | | Tenacissoside X | | HPLC≥98% | |  |
|  | A0692 | | Tenacissoside H | | HPLC≥98% | |  |
|  | A0749 | | Tenacissoside I | | HPLC≥98% | |  |
|  | A0750 | | Tenacissoside G | | HPLC≥98% | |  |
| Wolfiporias | A0575 | | Pachymic acid | | HPLC≥98% | |  |
|  | A1172 | | Dehydrotrametenolic acid | | HPLC≥98% | |  |
|  | A1389 | | Tsugaric acid A | | HPLC≥98% | |  |
|  | A1390 | | Trametenolic acid | | HPLC≥98% | |  |
|  | A1438 | | Dehydropachymic acid | | HPLC≥98% | |  |
| leaf of Broadleaf Holly | A1023 | | Kudinoside D | | HPLC≥98% | |  |
| S.aviculare Forst. | A0620 | | Solasonine | | HPLC≥98% | |  |
|  | A0621 | | Solamargine | | HPLC≥98% | |  |
|  | A0758 | | Solasodine | | HPLC≥98% | |  |
|  | A0790 | | Khasianine | | HPLC≥98% | |  |
| Ganoderma lucidum | A0630 | | Ergosterol | | HPLC≥98% | |  |
|  | A0842 | | Ganoderic acid A | | HPLC≥98% | |  |
|  | A0906 | | Ganoderic acid D | | HPLC≥98% | |  |
|  | A0907 | | Ganoderenic acid D | | HPLC≥98% | |  |
|  | A0908 | | Ganoderic acid F | | HPLC≥95% | |  |
|  | A0909 | | Ganoderic acid G | | HPLC≥98% | |  |
|  | A0910 | | Lucidenic acid LM1;  Lucidenic acid N | | HPLC≥98% | |  |
|  | A0911 | | Lucidenic acid C | | HPLC≥98% | |  |
|  | A0913 | | Lucidenic acid E | | HPLC≥98% | |  |
|  | A0914 | | Lucidenic acid L | |  | |  |
|  | A0915 | | Lucidenic acid A | | HPLC≥98% | |  |
|  | A0916 | | Lucidenic acid B | | HPLC≥98% | |  |
|  | A0917 | | Deacetyl ganoderic acid F | | HPLC≥98% | |  |
|  | A0920 | | Ganoderenic acid E | | HPLC≥98% | |  |
|  | A0921 | | Lucidenic acid D | | HPLC≥98% | |  |
|  | A0922 | | 23S-hydroxyl-11,15-dioxo-ganoderic acid DM | | HPLC≥98% | |  |
| Gelsemium elegans (Gardn. et Champ.) Benth. | A0696 | | Koumine | | HPLC≥98% | |  |
|  | A0697 | | Gelsemine | | HPLC≥98% | |  |
|  | A0955 | | Gelsenicine | | HPLC≥98% | |  |
|  | A1234 | | Sempervirine | | HPLC≥98% | |  |
|  | A1240 | | N-Methoxyanhydrovobasinediol | | HPLC≥98% | |  |
|  | A1241 | | rankinidine | | HPLC≥98% | |  |
|  | A1242 | | humantenirine | | HPLC≥98% | |  |
|  | A1243 | | Gelsevirine | | HPLC≥98% | |  |
|  | A1253 | | Humantenine | | HPLC≥98% | |  |
|  | A1254 | | 19-(Z)-Akuammidine | | HPLC≥95% | |  |
|  | A1255 | | Koumidine | | HPLC≥98% | |  |
| Rheum palmatum L | A0043 | | Rhein | | HPLC≥98% | |  |
|  | A0044 | | Emodin | | HPLC≥98% | |  |
|  | A0045 | | Physcion | | HPLC≥98% | |  |
|  | A0046 | | Chrysophanol | | HPLC≥98% | |  |
|  | A0047 | | Aloeemodin | | HPLC≥98% | |  |
|  | A0110 | | Gallic acid | | HPLC≥98% | |  |
|  | A0577 | | Rhein-8-O-β-D-glucopyranoside | | HPLC≥98% | |  |
|  | A0555 | | Emodin-8-glucoside | | HPLC≥98% | |  |
|  | A0762 | | Chrysophanol-8-O-β-D-glucopyranoside | | HPLC≥98% | |  |
| Eupatorium lindleyanum | A0767 | | Eupalinilide B | | HPLC≥96% | |  |
|  | A0768 | | Eupalinilide C | | HPLC≥98% | |  |
|  | A0769 | | Eupalinilide D | | HPLC≥98% | |  |
| Paederia scandense | A0858 | | Paederoside | | HPLC≥98% | |  |
|  | A0859 | | Paederosidic acid | | HPLC≥98% | |  |
|  | A0860 | | Paederosidic acid methyl ester | | HPLC≥98% | |  |
| Akebiaquinata | A0872 | | Calceolarioside B | | HPLC≥95% | |  |
| Rhododendron molle G. Don | A0565 | | Rhodojaponin II | | HPLC≥98% | |  |
|  | A0566 | | Rhodojaponin III | | HPLC≥98% | |  |
|  | A0567 | | Rhodojaponin V | | HPLC≥98% | |  |
| Puffer | A0224 | | Tetrodotoxin | | HPLC≥99% | |  |
| Bufonid | A0739 | | Resibufogenin | | HPLC≥98% | |  |
|  | A0375 | | Bufalin | | HPLC≥98% | |  |
|  | A0784 | | Cinobufagin | | HPLC≥95% | |  |
| Desmodiumstyracifolium（Osh.）Merr | A0792 | | Schaftoside | | HPLC≥98% | |  |
|  | A0856 | | Isoshaftoside | | HPLC≥98% | |  |
| Lamiophlomis rotata | A0495 | | Sesamoside | | HPLC≥98% | |  |
|  | A0528 | | Shanzhiside methylester | | HPLC≥98% | |  |
|  | A0531 | | Barlerin | | HPLC≥98% | |  |
|  | A1245 | | Phlorigidoside C | | HPLC≥97% | |  |
|  | A1246 | | Phloyoside I | | HPLC≥98% | |  |
|  | A1262 | | 5, 9-epi-Phlomiol | | HPLC≥98% | |  |
|  | A1263 | | 9-epi-Phlomiol | | HPLC≥98% | |  |
|  | A1264 | | 6β-hydroxyl-7-epi-loganin | | HPLC≥98% | |  |
|  | A1266 | | decaffeoyl acteoside | | HPLC≥98% | |  |
|  | A1277 | | Lamalbide | | HPLC≥97% | |  |
|  | A1278 | | 5-deoxypulchelloside I | | HPLC≥97% | |  |
|  | A1279 | | 6'-O-β-D-Glucopyranosyl phlorigidoside C | | HPLC≥98% | |  |
| Catharanthus roseus | A0461 | | Vindoline | | HPLC≥98% | |  |
|  | A0462 | | Catharanthine | | HPLC≥98% | |  |
|  | A0666 | | Vinorelbine | | HPLC≥98% | |  |
|  | A0667 | | Vinorelbine Tartrate | | HPLC≥98% | |  |
|  | A0668 | | Vinblastine | | HPLC≥98% | |  |
|  | A0669 | | Vinblastine Sulfate | | HPLC≥98% | |  |
|  | A0670 | | Anhydrovinblastine | | HPLC≥95% | |  |
|  | A0671 | | Catharanthine hemitartrate | | HPLC≥98% | |  |
|  | A0672 | | Catharanthine Sulfate | | HPLC≥98% | |  |
|  | A0673 | | Vincristine sulfate | | HPLC≥98% | |  |
|  | A0674 | | Vincristine | | HPLC≥94% | |  |
| Myristica fragrans Houtt | A0443 | | Dehydrodiisoeugenol | | HPLC≥98% | |  |
|  | A0444 | | (-)-Licarin B | | HPLC≥98% | |  |
|  | A0445 | | Myrislignan | | HPLC≥98% | |  |
| Echinacea Angustifolia | A0409 | | Cichoric Acid | | HPLC≥98% | |  |
|  | A0710 | |  | |  | |  |
| Alpinia katsumadai Hayata | A1051 | | Alpinetin | | HPLC≥98% | |  |
|  | A1052 | | Cardamonin | | HPLC≥98% | |  |
| Garcinia mangostana. L. | A0406 | | α-Mangostin | | HPLC≥98% | |  |
|  | A0407 | | β-Mangostin | | HPLC≥98% | |  |
|  | A0408 | | 3-Isomangostin | | HPLC≥98% | |  |
| Cynanchum auriculatumRoyle exW ight | A0396 | | Caudatin | | HPLC≥98% | |  |
|  | A0397 | | Qingyangshengenin A | | HPLC≥98% | |  |
|  | A0398 | | Qingyangshengenin B | | HPLC≥96% | |  |
| Paris yunnanensis Franch | A0386 | | Polyphyllin I | | HPLC≥98% | |  |
|  | A0387 | | Paris saponin II | | HPLC≥98% | |  |
|  | A0389 | | Polyphyllin VI | | HPLC≥98% | |  |
|  | A0390 | | Polyphyllin VII | | HPLC≥98% | |  |
|  | A0628 | | Gracillin | | HPLC≥98% | |  |
|  | A1314 | | Paris saponin V | | HPLC≥98% | |  |
|  | A1325 | | Diosgenin-3-O-rhamnosyl(1-2)[glucosyl(1-6)]glucoside | | HPLC≥98% | |  |
|  | A1336 | | Paris saponin V palmitate | | HPLC≥98% | |  |
|  | A1337 | | 17-hydroxygracillin | | HPLC≥98% | |  |
|  | A1338 | | Pennogenin -3-O-chacotrioside | | HPLC≥98% | |  |
| Dioscorea nipponica Makino | A0125 | | Dioscin | | HPLC≥98% | |  |
| D.Zingiberensis C. H. Wright | A0124 | | Diosgenin | | HPLC≥98% | |  |
|  | A0126 | | Protodioscin | | HPLC≥98% | |  |
|  | A0894 | | Pseudoprotodioscin | | HPLC≥98% | |  |
| Dioscorea opposite Thunb | A0661 | | Allantoin | | HPLC≥98% | |  |
| Cnidium monnieric(L.)Cuss | A0127 | | Osthole | | HPLC≥98% | |  |
|  | A0426 | | 5-Methoxypsoralen | | HPLC≥98% | |  |
|  | A0655 | | Xanthotoxol | | HPLC≥98% | |  |
| Kaempferia galanga L | A0129 | | Kaempferol | | HPLC≥98% | |  |
|  | A0478 | | Kaempferide | | HPLC≥98% | |  |
| Sophora flavescens Ait. | A0094 | | Matrine | | HPLC≥98% | |  |
|  | A0095 | | Oxymatrine | | HPLC≥98% | |  |
|  | A0441 | | Trifolirhizin;  (-)-Maackiain-3-O-glucoside | | HPLC≥98% | |  |
|  | A0481 | | N－Methylcytisine | | HPLC≥98% | |  |
|  | A0520 | | Oxysophocarpine | | HPLC≥98% | |  |
| Fiveleaf Gynostemma Herb | A0092 | | Stevenleaf | | UV≥98% | |  |
|  | A0605 | | Gypenoside XLIX | | HPLC≥98% | |  |
|  | A0934 | | Gypenoside A | | HPLC≥98% | |  |
|  | A0638 | | Gypenoside XVII | | HPLC≥98% | |  |
| Forsythia suspense（Thunb.）Vahl | A0090 | | Hyperoside | | HPLC≥98% | |  |
|  | A0091 | | Hypericin | | HPLC≥98% | |  |
|  | A0272 | | Forsythin | | HPLC≥98% | |  |
|  | A0590 | | Forsythoside A | | HPLC≥98% | |  |
|  | A0603 | | Forsythoside B | | HPLC≥96% | |  |
|  | A1193 | | Forsythoside E | | HPLC≥98% | |  |
|  | A1181 | | Forsythoside I | | HPLC≥98% | |  |
|  | A1144 | | Phillygenin | | HPLC≥98% | |  |
|  | A0862 | | (+)-pinoresinol-β-D-glucoside;(+)-pinoresinol-4-O-beta-D-glucopyranoside | | HPLC≥98% | |  |
|  | A1182 | | epipinoresinol-4-O-glucopyranoside | | HPLC≥98% | |  |
|  | A1183 | | epipinoresinol-4'-O-glucopyranoside | | HPLC≥98% | |  |
|  | A1187 | | Matairesinoside | | HPLC≥96% | |  |
|  | A1188 | | (+)-Pinoresinol monomethyl ether 4-O-β-D-glucoside | | HPLC≥98% | |  |
| C.longa L. | A0086 | | Curcumin | | HPLC≥98% | |  |
|  | A0087 | | Demethoxycurcumin | | HPLC≥98% | |  |
|  | A0088 | | Bisdemethoxycurcumin | | HPLC≥98% | |  |
| Curcuma zedoaria (Christm.) Rosc | A0089 | | Curcumol | | HPLC≥98% | |  |
|  | A0519 | | Curdione | | HPLC≥98% | |  |
|  | A1069 | | Curcumenol | | HPLC≥98% | |  |
|  | A1345 | | isogermafurenolide | | HPLC≥98% | |  |
| Artemisia apiacea | A0114 | | Artemisinine | | UV≥98% | |  |
|  | A0114H | | Artemisinine | | HPLC≥96% | |  |
|  | A0085 | | Artemether | | HPLC≥98% | |  |
|  | A0115 | | Dihydroartemisinin | | UV≥98% | |  |
|  | A0879 | | Artemisinic acid | | HPLC≥98% | |  |
|  | A1442 | | Artesunate | | HPLC≥98% | |  |
| Gossypiumherbaceum L | A0084 | | Quercitrin | | HPLC≥98% | |  |
|  | A0439 | | Isoquercitrin;  Isoquercetin;  Isoquercitroside | | HPLC≥98% | |  |
|  | A0743 | | Gossypol | | HPLC≥98% | |  |
|  | A0744 | | Acetate gossypol | | HPLC≥96% | |  |
| Rosmarinus officinalis L． | A0003 | | Carnosic acid | | HPLC≥98% | |  |
|  | A0004 | | Rosmarinic acid | | HPLC≥98% | |  |
|  | A0388 | | Carnosol | | HPLC≥98% | |  |
|  | A1293 | | 12-O-Methylcarnosic acid | | HPLC≥98% | |  |
|  | A1294 | | Rosmanol | | HPLC≥97% | |  |
|  | A1296 | | Eugenitin | | HPLC≥98% | |  |
|  | A1366 | | hydroxygenkwanin | | HPLC≥98% | |  |
|  | A1416 | | Salviaflaside | | HPLC≥98% | |  |
| Flacourtiajangomas(Lour.)Raeusch | A0011 | | Imperatorin | | HPLC≥98% | |  |
|  | A0012 | | Isoimperatorin | | HPLC≥98% | |  |
| Scutellaria baicalensis Georgi | A0016 | | Baicalin | | HPLC≥98% | |  |
|  | A0017 | | Scutellarin | | HPLC≥98% | |  |
|  | A0912 | | Scutellarin methylester | | HPLC≥97% | |  |
|  | A0018 | | Baicalein | | HPLC≥98% | |  |
|  | A0502 | | Wogonin | | HPLC≥98% | |  |
|  | A0595 | | Wogonoside | | HPLC≥98% | |  |
|  | A0990 | | Chrysin-7-O-glucoronide | | HPLC≥98% | |  |
| Fraxinus brngeana DC. | A0019 | | Fraxetin | | HPLC≥98% | |  |
|  | A0435 | | Fraxin;Fraxoside; Paviin | | HPLC≥98% | |  |
|  | A0479 | | Dimethylfraxetin | | HPLC≥98% | |  |
| Sophora flavescens Ai | A0081 | | Sophocarpine | | HPLC≥98% | |  |
|  | A0083 | | Quercetin | | HPLC≥98% | |  |
|  | A0103 | | Rutin | | UV≥98% | |  |
|  | A0103 | | Rutin | | HPLC≥98% | |  |
|  | A0382 | |  | | HPLC≥98% | |  |
|  | A1137 | | Sophoricoside | | HPLC≥98% | |  |
|  | A0474 | | Quercetin-7-O-β-D-glucopyranoside | | HPLC≥98% | |  |
| Citrus paradisi Macfadyen | A0132 | | Narirutin | | HPLC≥98% | |  |
|  | A0146 | | Naringin | | HPLC≥98% | |  |
|  | A0147 | | Naringenin | | HPLC≥98% | |  |
| Coptis chinensis | A0151 | | Berberine hydrochloride | | HPLC≥98% | |  |
|  | A0935 | | Columbamine | | HPLC≥98% | |  |
|  | A0627 | | Epiberberine | | HPLC≥98% | |  |
|  | A0947 | | Groenlandicine;  Tetradehydrocheilanthifoline | | HPLC≥98% | |  |
|  | A0153 | | Coptisine | | HPLC≥98% | |  |
|  | A0154 | | Coptisine chloride | | HPLC≥98% | |  |
|  | A0618 | | Coptisine Sulfate | | HPLC≥98% | |  |
| Cortex Phellodendri Chinensis | A0152 | | Berberine | | HPLC≥98% | |  |
|  | A0265 | | Obacunone | | HPLC≥98% | |  |
|  | A0517 | | Phellodendrine | | HPLC≥98% | |  |
|  | A0786 | | Demethyleneberberine | | HPLC≥98% | |  |
| Rhizoma picrorhizae | A0155 | | Picroside I | | HPLC≥98% | |  |
|  | A0156 | | Picroside II | | HPLC≥98% | |  |
|  | A0157 | | Picroside III | | HPLC≥98% | |  |
|  | A1194 | | picroside IV | | HPLC≥98% | |  |
| Gentiana manshurica Kitag. | A0170 | | Gentiopicrin | | HPLC≥98% | |  |
| SesamumindicumDC | A0174 | | Sesamin | | HPLC≥98% | |  |
|  | A0169 | | Phytic acid | |  | |  |
|  | A0175 | | Sesamolin | | HPLC≥98% | |  |
| Mucuna cochinchine-sis (Lour)Tang et Wang | A0176 | | Levodopa | | UV≥99% | |  |
| Taxaceae | A0177 | | Paclitaxel | | HPLC≥98% | |  |
|  | A0337 | | 10-Deacetyl baccatin | | HPLC≥98% | |  |
|  | A0753 | | Cephalomannine | | HPLC≥98% | |  |
|  | A0754 | | Docetaxel | | HPLC≥96% | |  |
|  | A0755 | | Deacetyltaxol | | HPLC≥95% | |  |
|  | A0756 | | 7-Epitaxol | | HPLC≥94% | |  |
|  | A1082 | | 10-Deacetyl-7-xylosyl paclitaxel | | HPLC≥82% | |  |
|  | A1083 | | Deacetyltaxol | | HPLC≥92% | |  |
|  | A1084 | | Baccatin III | | HPLC≥98% | |  |
| Gardenia jasminoides Ellis | A0178 | | Geniposide | | HPLC≥98% | |  |
|  | A0526 | | Geniposidic acid | | HPLC≥98% | |  |
|  | A0516 | | Genipin | | HPLC≥98% | |  |
|  | A1079 | | 6α-hydroxygeniposide | | HPLC≥97.5% | |  |
|  | A1412 | | Asperulosidic acid | | HPLC≥98% | |  |
|  | A1413 | | Asperuloside | | HPLC≥98% | |  |
| Magnolia officinalis Rehd. et Wils. | A0180 | | Magnolol | | HPLC≥98% | |  |
|  | A0181 | | Honokiol | | HPLC≥98% | |  |
|  | A1035 | | Liriodenine | | HPLC≥98% | |  |
|  | A1380 | | Magnoloside A | | HPLC≥98% | |  |
| Cassia angutifolia | A0182 | | Sennoside A | | HPLC≥97% | |  |
|  | A0183 | | Sennoside B | | HPLC≥98% | |  |
|  | A0184 | | Sennoside C | | HPLC≥98% | |  |
|  | A0185 | | Sennoside D | | HPLC≥98% | |  |
|  | A0582 | | Sennidin A | | HPLC≥98% | |  |
|  | A0583 | | Sennidin B | | HPLC≥98% | |  |
| Ligusticum chuanxiong Hort. | A0188 | | 2,3,5,6-Tetramethylpyrazine | | HPLC≥98% | |  |
|  | A0189 | | Ligustrazine Hydrochloride | | HPLC≥98% | |  |
|  | A0469 | | Ethyl ferulate | | HPLC≥98% | |  |
|  | A0571 | | 3-Butylidenephthalide | | HPLC≥98% | |  |
|  | A0580 | | Senkyunolide H | | HPLC≥98% | |  |
|  | A0579 | | Senkyunolide A | | HPLC≥98% | |  |
|  | A0581 | | Senkyunolide I | | HPLC≥98% | |  |
|  | A0592 | | Levistilide A | | HPLC≥98% | |  |
|  | A0219 | | Z-Ligustilide | | HPLC≥98% | |  |
|  | A0761 | | 3-n-Butylphathlide;3-Butylphthalide | | HPLC≥98% | |  |
| Angelica sinensis(Oliv.)Diels | A0491 | | Columbianadin | | HPLC≥98% | |  |
| Inula helenium L. | A0221 | | Alantolactone | | HPLC≥98% | |  |
|  | A0284 | | Isoalantolactone | | HPLC≥98% | |  |
| Evodia rutaecarpa (Juss.) Benth. | A0223 | | Nomilin | | HPLC≥98% | |  |
|  | A0266 | | Evodiamine | | HPLC≥98% | |  |
|  | A0267 | | Rutaecarpine | | HPLC≥98% | |  |
| Acanthopanax senticosus Harms | A0252 | | Ciwujianoside-B | | HPLC≥96% | |  |
|  | A0254 | | Eleutheroside E | | HPLC≥98% | |  |
|  | A0256 | | Hedera saponin B | | HPLC≥98% | |  |
|  | A1252 | | Eleutheroside E1 | | HPLC≥98% | |  |
|  | A1275 | | Eugenol rutinoside | | HPLC≥95% | |  |
|  | A1276 | | Salvadoraside | | HPLC≥95% | |  |
| Ziziphus psinosa Hu | A0274 | | Jujuboside A | | HPLC≥98% | |  |
|  | A0275 | | Jujuboside B | | HPLC≥98% | |  |
|  | A0585 | | Spinosin | | HPLC≥98% | |  |
| Caulis Polygoni Multiflori | A0269 | | 2,3,5,4-tetrahydroxyl diphenylethylene -2-o-glucoside | | HPLC≥98% | |  |
| Cimicifnga foetida L. | A0278 | | Prim-o-glucosylcimifugin | | HPLC≥98% | |  |
|  | A0279 | | cimifugin | | HPLC≥98% | |  |
|  | A0509 | | 3-Hydroxy-4-methoxycinnamic acid | | HPLC≥98% | |  |
|  | A0800 | | Acetylcimigenol-3-O-α-L-arabinopyranside | | HPLC≥98% | |  |
|  | A0601 | | Cimigenol-3- O-α-L -arabinoside | | HPLC≥98% | |  |
| Cistanche deserticola Y.C. Ma | A0280 | | acteoside | | HPLC≥98% | |  |
|  | A0281 | | Isoacteoside | | HPLC≥98% | |  |
|  | A0282 | | Echinacoside | | HPLC≥98% | |  |
|  | A0942 | | Tubuloside A | | HPLC≥98% | |  |
|  | A0943 | | 2-acetylacteoside | | HPLC≥98% | |  |
|  | A1019 | | Cistanoside A | | HPLC≥95% | |  |
|  | A1037 | | Cistanoside F | | HPLC≥98% | |  |
| Saposhnikovia divaricata (Turcz.) Schischk | A0283 | | 4’-O-glucopyranosyl-5-O-methylvisamminol | | HPLC≥98% | |  |
|  | A0431 | | Sec-O-Glucosylhamaudol | | HPLC≥97% | |  |
| Berberis julianae Schneid | A0285 | | Jatrorrhizine Hydrochloride | | HPLC≥94% | |  |
|  | A0619 | | Berbamine hydrochloride | | HPLC≥98% | |  |
|  | A0748 | | Tetrahydroberberine,THB | | HPLC≥98% | |  |
| Oroxylum indicum(L.)Vent. | A0292 | | Chrysin | | HPLC≥98% | |  |
|  | A0596 | | Oroxyloside | | HPLC≥98% | |  |
|  | A0967 | | Oroxylin A | | HPLC≥98% | |  |
|  | A0599 | | Oroxin A | | HPLC≥98% | |  |
|  | A0600 | | Oroxin B | | HPLC≥98% | |  |
|  | A1142 | | Chrysin 7-O-beta-gentiobioside | | HPLC≥98% | |  |
|  | A1157 | | Guaijaverin | | HPLC≥98% | |  |
| Camptotheca acuminata | A0294 | | Camptothecine | | HPLC≥98% | |  |
|  | A0414 | | 9-methoxycamptothecine | | HPLC≥98% | |  |
|  | A0420 | | 10-hydroxycamptothecin | | HPLC≥98% | |  |
|  | A1426 | | 7-Ethylcamptothecin | | HPLC≥98% | |  |
|  | A0745 | | 7-Ethyl-10-hydroxycamptothecin | | HPLC≥98% | |  |
|  | A0746 | | Irinotecan | | HPLC≥98% | |  |
| Euphorbiae Lathyridis L. | A0295 | | Euhorbiasteroid | | HPLC≥98% | |  |
|  | A0296 | | Lathyrol | | HPLC≥98% | |  |
|  | A0591 | | Euphorbia factor L3;  5,15-Diacetyl-3-benzoyllathyrol;  Diacetyl Benzoyl Lathyrol | | HPLC≥98% | |  |
|  | A1205 | | Euphorbiasteroid | | HPLC≥98% | |  |
| Cinnamon | A0297 | | Borneol | | HPLC≥98% | |  |
|  | A0305 | | Cinnamic acid | | HPLC≥98% | |  |
|  | A0306 | | Cinnamyl alcohol | | HPLC≥98% | |  |
|  | A0307 | | Cinnamaldehyde | | HPLC≥98% | |  |
| Cornus officinalis Sieb. et Zucc | A0298 | | 5-hydroxymethyl-2-furaldehyde | | HPLC≥98% | |  |
|  | A0317 | | Loganin | | HPLC≥98% | |  |
|  | A0348 | | cornin | | HPLC≥98% | |  |
|  | A0349 | | Morroniside | | HPLC≥98% | |  |
|  | A0733 | | Cornuside I | | HPLC≥98% | |  |
|  | A0734 | | 7-O-ethyl-morroniside | | HPLC≥98% | |  |
|  | A1020 | | Loganetin | | HPLC≥98% | |  |
| Auckiandialappa Decne | A0301 | | Dehydrocostus Lactone | | HPLC≥98% | |  |
|  | A0302 | | Costunlide | | HPLC≥98% | |  |
| Psoralea corylifolia L | A0303 | | Psoralen | | HPLC≥98% | |  |
|  | A0304 | | Isopsoralen | | HPLC≥98% | |  |
|  | A0399 | | Bakuchiol | | HPLC≥98% | |  |
|  | A1092 | | Psoralidin | | HPLC≥98% | |  |
|  | A1097 | | Bavachinin A | | HPLC≥98% | |  |
|  | A1099 | | Isobavachalcone | | HPLC≥98% | |  |
|  | A1100 | | 4'-O-Methylbroussochalcone B | | HPLC≥98% | |  |
|  | A1101 | | Bavachin | | HPLC≥98% | |  |
|  | A1108 | | Corylifol A | | HPLC≥98% | |  |
|  | A1109 | | Neobavaisoflavone | | HPLC≥98% | |  |
|  | A1116 | | Isobavachromene | | HPLC≥98% | |  |
|  | A1117 | | 3-hydroxybakuchiol | | HPLC≥98% | |  |
|  | A1118 | | Bavachromene | | HPLC≥98% | |  |
|  | A1119 | | 8-Prenyldaidzein | | HPLC≥98% | |  |
|  | A1120 | | Coumestrol | | HPLC≥98% | |  |
|  | A1121 | | 8-Geranyl daidzein | | HPLC≥98% | |  |
|  | A1123 | | 3'-methoxycoumestrol | | HPLC≥98% | |  |
|  | A1124 | | Corylifol C | | HPLC≥98% | |  |
|  | A1125 | | Paratocarpin K | | HPLC≥98% | |  |
|  | A1130 | | Delta3,2-Hydroxylbakuchiol | | HPLC≥98% | |  |
| Mangifera indica L | A0309 | | Mangiferin | | HPLC≥98% | |  |
|  | A0310 | | Neomangiferin | | HPLC≥98% | |  |
| Platycodon grandiforus | A0321 | | Platycodin D | | HPLC≥98% | |  |
|  | A0613 | | Deapio platycodin D | | HPLC≥98% | |  |
|  | A1388 | | Deapi-platycoside D2 | | HPLC≥98% | |  |
|  | A1395 | | Deapi-platycodin D3 | | HPLC≥98% | |  |
|  | A1404 | | Platycodin D2 | | HPLC≥98% | |  |
|  | A1386 | | Platycodin D3 | | HPLC≥98% | |  |
|  | A1383 | | DL-Arabinose | | HPLC≥98% | |  |
| Peucedanum praeruptorum Dunn | A0329 | | (-)-pareruptorin A | | HPLC≥98% | |  |
|  | A0330 | | Praeruptorin B | | HPLC≥98% | |  |
|  | A0425 | | Peucedanol | | HPLC≥98% | |  |
|  | A0751 | | Praeruptorin C | | HPLC≥98% | |  |
|  | A0290 | | Pteryxin | | HPLC≥98% | |  |
| Caesalpinia sappan L. | A0344 | | Brazilin | | HPLC≥98% | |  |
|  | A0365 | | Protosappanin B | | HPLC≥98% | |  |
| Hemsleya amabilis Diels | A0361 | | Curcurbitacin IIa | | HPLC≥84% | |  |
|  | A0362 | | Curcurbitacin Iib | | HPLC≥98% | |  |
| Foeniculum vulgare | A0363 | | vitexicarpin | | HPLC≥98% | |  |
|  | A0777 | | Trans-Anethol | | HPLC≥98% | |  |
| Lindera aggregata (Sims) Kosterm. | A0367 | | Linderane | | HPLC≥98% | |  |
|  | A0458 | | Isolinderalactone | | HPLC≥98% | |  |
|  | A0538 | | Norisoboldine | | HPLC≥98% | |  |
|  | A1319 | | Lindenenyl acetate | | HPLC≥98% | |  |
|  | A1320 | | Lindenenol | | HPLC≥98% | |  |
|  | A1321 | | Linderalactone | | HPLC≥98% | |  |
|  | A1326 | | Linderanine C | | HPLC≥98% | |  |
|  | A1339 | | Hydroxylinderstrenolide | | HPLC≥98% | |  |
| Carthamus tinctorius L | A0380 | | hydroxysafflor yellow A | | HPLC≥98% | |  |
|  | A1351 | | Crocetin | | HPLC≥98% | |  |
|  | A0210 | | Crocin | |  | |  |
|  | A0211 | | Crocin I | | HPLC≥98% | |  |
|  | A0212 | | Crocin II | | HPLC≥98% | |  |
|  | A1041 | | Kaempferol-3-O-sophoroside | | HPLC≥98% | |  |
|  | A1042 | | 6-hydroxyapigenin-6-O-β-D-glucoside-7-O-β-D-glucuronide | | HPLC≥98% | |  |
|  | A1043 | | 6-Hydroxykaempferol 3,6-diglucoside | | HPLC≥98% | |  |
|  | A1044 | | 6-Hydroxykaempferol-3,6,7-triglucoside | | HPLC≥98% | |  |
|  | A1045 | | 6-Hydroxykaempferol 3-Rutinoside -6-glucoside | | HPLC≥98% | |  |
|  | A1046 | | 6-hydroxyl kaempherol-3,6-O-diglucosyl-7-O-Glucuronic acid | | HPLC≥98% | |  |
|  | A1235 | | 3,4’,5,7-Tetrahydroxy-6-methoxyflaone 3-O-β-D-rutinoside | | HPLC≥98% | |  |
|  | A1236 | | Anhydrosafflor yellow B | |  | |  |
|  | A1439 | | 6-hydroxylapigenin-6,7-diglucoside | | HPLC≥98% | |  |
| Morus alba L. | A0213 | | Morin hydrate | | HPLC≥98% | |  |
|  | A0220 | | 1-DNJ ;  1-Deoxynojirimycin | | HPLC≥98% | |  |
|  | A0569 | | Mulberroside A | | HPLC≥98% | |  |
|  | A0570 | | Mulberroside C | | HPLC≥98% | |  |
|  | A0578 | | Sanggenone C | | HPLC≥98% | |  |
|  | A0589 | | Sanggenone D | | HPLC≥98% | |  |
|  | A0701 | | Morusin | | HPLC≥98% | |  |
|  | A0923 | | Scopolin | | HPLC≥98% | |  |
|  | A1316 | | Kuwanon A | | HPLC≥98% | |  |
|  | A1317 | | Kuwanon T | | HPLC≥98% | |  |
|  | A1349 | | Mulberrofuran A | | HPLC≥98% | |  |
|  | A1342 | | Mulberrofuran B | | HPLC≥98% | |  |
|  | A1350 | | Cyclomorusin | | HPLC≥98% | |  |
| Centella asiatica (L. ) Urban | A0192 | | Asiaticoside | | HPLC≥98% | |  |
|  | A0193 | | Madecassoside | | HPLC≥98% | |  |
|  | A0194 | | Asiatic acid | | HPLC≥98% | |  |
|  | A0195 | | Madecassic acid | | HPLC≥98% | |  |
|  | A1122 | | Asiaticoside B | | HPLC≥98% | |  |
|  | A1133 | | 2α,6β,23-trihydroxyl oleanolic acid；  Terminolic acid | | HPLC≥98% | |  |
|  | A1154 | | Scheffoleoside A | | HPLC≥95% | |  |
|  | A1274 | | Isoasiaticoside | | HPLC≥94% | |  |
| Rhizoma Belamcandae | A0201 | | Tectorigenin | | HPLC≥98% | |  |
|  | A0202 | | Tectoridin | | HPLC≥98% | |  |
|  | A1280 | | Iridin | | HPLC≥98% | |  |
|  | A1297 | | Irigenin | | HPLC≥98% | |  |
|  | A0198 | | Capsaicin | | HPLC≥98% | |  |
| CapsicumannuumL. | A1056 | | Capsaicin | | HPLC≥98% | |  |
|  | A0199 | | Dihydrocapsaicin | | HPLC≥98% | |  |
|  | A0065 | | Tetrandrine | | HPLC≥98% | |  |
| Stephania tetrandra S. Moore | A0066 | | Fangchinoline | | HPLC≥98% | |  |
| SinomeniumAcutumRehderettWilson | A0150 | | Sinomenine Hydrochloride | | HPLC≥98% | |  |
|  | A0148 | | Proanthocyanidins | | UV≥95% | |  |
| Grape | A0447 | | Procyanidin B2 | | HPLC≥98% | |  |
|  | A0698 | | Procyanidin B1 | | HPLC≥95% | |  |
|  | A1022 | | Procyanidin B4 | | HPLC≥98% | |  |
| Corydalis yanhusuo W.T. Wang | A0144 | | Tetrahydropalmatine | | HPLC≥98% | |  |
|  | A0781 | | (+)- Corydaline | | HPLC≥98% | |  |
|  | A0782 | | Dehydrocorydaline | | HPLC≥98% | |  |
| Ligustrum lucidum Ait. | A0139 | | Ursolic acid | | HPLC≥98% | |  |
|  | A0525 | | Specnuezhenide | | HPLC≥98% | |  |
|  | A0791 | | Ligustroflavone | | HPLC≥98% | |  |
| Paeonia albiflora Pall | A0133 | | Paeoniflorin | | HPLC≥98% | |  |
|  | A0477 | | Albiflorin | | HPLC≥98% | |  |
|  | A0548 | | Benzoylpaeoniflorin | | HPLC≥98% | |  |
|  | A0574 | | Oxypaeoniflorin | | HPLC≥98% | |  |
|  | A0077 | | Rosarin | | HPLC≥98% | |  |
| Rhodiola | A0078 | | Rosavin | | HPLC≥98% | |  |
|  | A0079 | | Rosin | | HPLC≥98% | |  |
|  | A0080 | | Rosiridin | | HPLC≥98% | |  |
|  | A0076 | | Salidroside | | HPLC≥98% | |  |
|  | A0452 | | Rhodionin | | HPLC≥98% | |  |
|  | A0068 | | Puerarin | | HPLC≥98% | |  |
| Menispermum dauricum | A0116 | | Sinomenine | | HPLC≥98% | |  |
|  | A0315 | | Dauricine | | HPLC≥98% | |  |
|  | A0996 | | Daurisoline | | HPLC≥98% | |  |
|  | A0048 | | Myricetin | | HPLC≥98% | |  |
| Myrica rubra （Lour.）Sieb. et Zucc | A0521 | | Myricetrin | | HPLC≥98% | |  |
|  | A0049 | | Dihydromyricetin | | HPLC≥98% | |  |
|  | A0051 | | Resveratrol | | HPLC≥98% | |  |
| Polygonum cuspidatum Sieb. et Zucc. | A0052 | | Polydatin | | HPLC≥98% | |  |
| Polygala japonicaHoutt | A0688 | | Polygalasaponin V | | HPLC≥98% | |  |
| Labiatae | A0779 | | Scutellarein | | HPLC≥98% | |  |
| Andrographis paniculata(Burm.f.)Nees | A0036 | | Andrographolide | | HPLC≥98% | |  |
|  | A0473 | | 14-Dehydroandrographolide | | HPLC≥98% | |  |
|  | A0573 | | Neoandrographolide | | HPLC≥98% | |  |
|  | A1394 | | Dehydroandrographolide | | HPLC≥98% | |  |
|  | A0340 | | Byakangelicin | | HPLC≥98% | |  |
| Ruta graveolens L. | A0504 | | Umbelliferone | | HPLC≥98% | |  |
|  | A0333 | | Typhaneoside | | HPLC≥98% | |  |
| Typha angustifolia L. | A0432 | | Isorhamnetin-3-O-neohespeidoside | | HPLC≥98% | |  |
|  | A0354 | | Loganic acid | | HPLC≥98% | |  |
| Gentiana macrophylla Pall | A0639 | | Roburic acid | | HPLC≥98% | |  |
|  | A0358 | | Vitexin | | HPLC≥98% | |  |
| Crateagus pinnatifida | A0513 | | Glucosyl-vitexin | | HPLC≥98% | |  |
|  | A0925 | | Maslinic acid | | HPLC≥98% | |  |
|  | A0383 | | Trigonelline Hydrochloride | | HPLC≥98% | |  |
| Trigonellafoenum-graecumL. | A0654 | | Trigonelline | | HPLC≥98% | |  |
|  | A0395 | | Nobiletin | | HPLC≥98% | |  |
| Citrus nobilis Lour. | A0795 | | Tangeretin | | HPLC≥98% | |  |
|  | A0428 | | cyanidin-3-O-glucoside | | HPLC≥90% | |  |
| Glycinemax (L.) meri | A0428 | | cyanidin-3-O-glucoside | | HPLC≥98% | |  |
|  | A0429 | | Cyanidin Chloride | | HPLC≥98% | |  |
|  | A0488 | | Fraxinellone | | HPLC≥98% | |  |
| Dictamnus dasycarpus Turcz | A0489 | | Dictamine | | HPLC≥98% | |  |
|  | A1009 | | 8-Hydroxy dictanmnine | | HPLC≥98% | |  |
|  | A1017 | | γ-Fagarine | | HPLC≥98% | |  |
|  | A0625 | | Ziyuglycoside I | | HPLC≥98% | |  |
| I. pubescens Hook. et Arn. | A0626 | | Ziyuglycoside II | | HPLC≥98% | |  |
|  | A0616 | | 3,4-Dihydroxybenzaldehyde | | HPLC≥98% | |  |
| Stenoloma Chusanum(L.)Ching | A0617 | | 3,4-Dihydroxybenzoic acid | | HPLC≥98% | |  |
|  | A0054 | | Paeonol | | HPLC≥99% | |  |
| Paeonia moutan Sim | A0369 | | Paeonolide | | HPLC≥98% | |  |
| Mylabris cichorii L.de | A0678 | | Sodium Demethylcantharidate | | HPLC≥98% | |  |
|  | A0677 | |  | | HPLC≥98% | |  |
|  | A0167 | | Cantharidin | | HPLC≥98% | |  |
|  | A0168 | | Norcantharidin | | HPLC≥98% | |  |
|  | A0173 | | Deoxycholic acid | | HPLC≥98% | |  |
|  | A0172 | | Hyodeoxycholic acid | | UV≥98% | |  |
|  | A0171 | | Cholic acid | | HPLC≥98% | |  |
|  | A1425 | | Ursodeoxycholic acid | | HPLC≥98% | |  |
| Codonopsis pilosula | A0002 | | Lobetyolin | | HPLC≥98% | |  |
|  | A1396 | | Lobetyol | | HPLC≥98% | |  |
|  | A1406 | | Pinoresinol | | HPLC≥98% | |  |
|  | A1407 | | Lariciresinol | | HPLC≥98% | |  |
|  | A1408 | | Isolariciresinol | | HPLC≥98% | |  |
|  | A1409 | | secoisolariciresinol | | HPLC≥98% | |  |
|  | A1410 | | (7S，8R)-didydrodehydrodiconiferyl alcohol | | HPLC≥98% | |  |
|  | A1428 | | lobetyolinin | | HPLC≥98% | |  |
|  | A0482 | | (-)-Sparteine sulfate pentahydrate | | HPLC≥98% | |  |
| Genista tinctoria Linn | A0675 | |  | | HPLC≥98% | |  |
| Aesculushippocastanumlinn | A0020 | | Esculin | | HPLC≥98% | |  |
|  | A0021 | | 6,7-Dihydroxycoumarin | | HPLC≥98% | |  |
|  | A0722 | | Escin IA | | HPLC≥98% | |  |
|  | A0723 | | Escin IB | | HPLC≥98% | |  |
|  | A0724 | | Aescin IIA | | HPLC≥98% | |  |
|  | A0725 | | AescinIIB | | HPLC≥98% | |  |
|  | A0031 | | Palmatine hydrochloride | | HPLC≥98% | |  |
| Herba fibraureae recisae | A0893 | | Palmatine | | HPLC≥98% | |  |
| Tangerine Peel | A0032 | | Hesperidin | | HPLC≥98% | |  |
|  | A0033 | | Neohesperidin | | HPLC≥98% | |  |
| Poncirus trifoliata（L.）Raf. | A0222 | | Limonin | | HPLC≥98% | |  |
|  | A0035 | | Hesperitin | | HPLC≥98% | |  |
| Citrus limon | A0927 | | Diosmetin | | HPLC≥98% | |  |
|  | A1295 | | citrin | | HPLC≥98% | |  |
| Eclipta prostrasta L | A0747 | | Wedelolactone | | HPLC≥98% | |  |
| Ferula asafoetida | A0050 | | Ferulic acid | | HPLC≥98% | |  |
| Vaccaria segetalis(Neck.)Garcke | A0053 | | Vaccarin | | HPLC≥98% | |  |
|  | A0971 | | Segetalin A | | HPLC≥98% | |  |
| the seed of cowherd | A0972 | | Segetalin B | | HPLC≥98% | |  |
| Rabdosia rubescens | A0063 | | Oridonin | | HPLC≥98% | |  |
| PodophyUum emodi Wal1 | A0067 | | Podophyllotoxin | | HPLC≥98% | |  |
| the rhizome of Dysosma versipellis(Hance.)M.Cheng | A0970 | | Picropodophyllotoxin | | HPLC≥98% | |  |
| Syzygium aromaticum | A0074 | | Eugenol | | HPLC≥99% | |  |
|  | A1393 | | Methyl eugenol | | HPLC≥98% | |  |
|  | A1417 | | Isoeugenol | | HPLC≥98% | |  |
| Syringa vulgaris L | A0253 | | Syringin | | HPLC≥98% | |  |
| Lobular boxwood | A0075 | | Cyclovirobuxine | | Titration≥99% | |  |
|  | A0552 | | Dihydrocurcumin | | HPLC≥98% | |  |
| Curcuma aromatica Salisb | A0320 | | Germacrone | | HPLC≥99% | |  |
| Korea huai | A0434 | | (±)-Maackiain; l- Maackiain | | HPLC≥98% | |  |
| Robinia pseudoacacia L | A0763 | | Acacetin | | HPLC≥98% | |  |
| Douglas fir | A0082 | | Taxifolin | | HPLC≥98% | |  |
| The northeast bitter apricot | A0093 | | Amygdalin | | HPLC≥98% | |  |
|  | A0096 | | Caffeic acid | | HPLC≥98% | |  |
| Crataegus pinnatifida Bge.var. major N.E.Br | A0357 | | vitexin-2″-o-rhamnoside | | HPLC≥98% | |  |
|  | A0098 | | Alphalipoic acid | | HPLC≥98% | |  |
| Carrot-root | A0299 | | β-Carotene | |  | |  |
| Rotundine | A0101 | | Rotundine | | HPLC≥98% | |  |
| Astilbe chinensis | A0102 | | Astilbin | | HPLC≥98% | |  |
| Reseda odorata | A0108 | | Luteolin | | HPLC≥98% | |  |
| Chrysanthemum indicum L | A0111 | | Buddleoside | | HPLC≥98% | |  |
| Anise | A0112 | | Shikimic acid | | HPLC≥97% | |  |
| Apium graveolens L. var. dulce DC | A0113 | | Apigenin | | HPLC≥98% | |  |
| olea europaea l. | A0117 | | Oleanolic acid | | HPLC≥98% | |  |
| Aesculus wilsonii | A0118 | | Sodium Aescinate | | ≥80% | |  |
|  | A0119 | | Xylitol | | HPLC≥98% | |  |
| Sugarcane | A1371 | | sucrose | | HPLC≥98% | |  |
| Corynante Yohimbe | A0120 | | Yohimbine Hydrochloride | | HPLC≥98% | |  |
| Nicotiana sanderae | A0121 | | Nicotine | | HPLC≥98% | |  |
| ThermoPsis lanceolate | A0122 | | Cytisine | | HPLC≥98% | |  |
| Colchicum autumnale | A0123 | | Colchicine | | HPLC≥98% | |  |
| Silybum marianum | A0131 | | Silymarin | | HPLC≥98% | |  |
|  | A0702 | | Isosilybin | | HPLC≥98% | |  |
|  | A0703 | | Silydianin | | HPLC≥98% | |  |
|  | A0704 | | Silicristin | | HPLC≥97% | |  |
|  | A0134 | | Rhamnose | | HPLC≥98% | |  |
| Rhus toxicodendron | A0689 | | Rhoifolin | | HPLC≥98% | |  |
| Evolvulus alsinoides | A0135 | | Betaine | | HPLC≥98% | |  |
| Lycium barbarum L. | A0136 | | Betaine hydrochloride | | HPLC≥98% | |  |
| Gastrodia elata | A0138 | | Gastrodin | | HPLC≥98% | |  |
| Bearberry | A0140 | | Arbutin | | HPLC≥98% | |  |
| Bitter orange | A0141 | | Synephrine | | HPLC≥98% | |  |
|  | A0735 | | 4-hydroxyephedrine hydrochloride | |  | |  |
| Tonka beans | A0142 | | Coumarin | | HPLC≥98% | |  |
| Fagopyrum cymosum Meisn | A0143 | | *p*-Coumaric acid | | HPLC≥98% | |  |
| Bergenia purpurascens | A0149 | | Bergenin | | HPLC≥98% | |  |
| Hippophae fhamnoides L. | A0190 | | Isorhamnetin | | HPLC≥98% | |  |
| Maize | A0191 | | D-(+)-Xylose | | HPLC≥99% | |  |
| Piper nigrum L. | A0200 | | Piperine | | HPLC≥98% | |  |
| Oat | A0209 | | Nicotinamide | | HPLC≥99.5% | |  |
| Cinchona | A0214 | | D-(-)-Quinic acid | | HPLC≥98% | |  |
| RemhnnaaigiutniosaLibosch | A0215 | | Catalpol | | HPLC≥98% | |  |
|  | A0694 | | Aucubin | | HPLC≥98% | |  |
|  | A1129 | | Rehmannioside D | | HPLC≥98% | |  |
|  | A1128 | | Rehmannioside A | |  | |  |
|  | A1411 | | Melittoside | | HPLC≥98% | |  |
|  | A0216 | | Phloridzin | | HPLC≥99% | |  |
| Apple | A0376 | | Phloretin | | HPLC≥98% | |  |
| Cyanotis,arachnoidea,C,B,Clarke | A0217 | | Ecdysterone | | HPLC≥98% | |  |
| Swertia mussotii | A0227 | | Swertiamarin | | HPLC≥98% | |  |
| Anemone raddeana Regel | A0255 | | Raddeanin A | | HPLC≥98% | |  |
|  | A0801 | | Raddeanoside R8(CAS: 124961-61-1) | | HPLC≥98% | |  |
|  | A0814 | | Oleanolic acid-3-O-β-D-glucopyranosyl (1→2)-α-L-arabinopyranoside(CAS：60213-69-6) | | HPLC≥98% | |  |
|  | A0263 | | Arctiin | | HPLC≥98% | |  |
| Fructus Arctii | A0546 | | Arctigenin | | HPLC≥98% | |  |
|  | A0277 | | Betulinic acid | | HPLC≥98% | |  |
| Betula alba L. | A0455 | | Betulin | | HPLC≥98% | |  |
|  | A0456 | | Betulinaldehyde | | HPLC≥98% | |  |
|  | A0323 | | Orientin | | HPLC≥98% | |  |
| Flos Trollii Chinensis | A0728 | | 2"-O-beta-L-galactopyranosylorientin | |  | |  |
|  | A0649 | | Cucurbitacin B | | HPLC≥98% | |  |
| Muskmelon | A1341 | | Cucurbitacin D | | HPLC≥98% | |  |
|  | A0695 | | Cucurbitacin E | | HPLC≥98% | |  |
|  | A1353 | | Isocucurbitacin B | | HPLC≥98% | |  |
|  | A1354 | | 3-epi-isocucurbitacin B | | HPLC≥98% | |  |
|  | A0659 | | Eupalinolide A | | HPLC≥98% | |  |
| Wheel Ye Zelan | A0660 | | Eupalinolide B | | HPLC≥96% | |  |
|  | A1156 | | Eupalinolide C | | HPLC≥98% | |  |
|  | A0664 | | Liquidambaric lactone | | HPLC≥98% | |  |
| Liquidambar formosana Hance | A0788 | | Liquidambaric acid | | HPLC≥95% | |  |
|  | A0705 | | Garcinone C | | HPLC≥98% | |  |
| Garcinia mangostana | A0706 | | Garcinone D | | HPLC≥98% | |  |
|  | A0811 | | 3β-acetoxy-eupha- 7,25-dien-24(R)-ol | | HPLC≥98% | |  |
| Broussonetia papyrifera | A0813 | | Broussonetine A | | HPLC≥98% | |  |
| Anemone hupehensis | A0802 | | Huzhangoside D | | HPLC≥98% | |  |
|  | A0804 | | Prosapogenin CP6 | | HPLC≥98% | |  |
|  | A0815 | | Hederagenin-3-O-α-L-rhamnopyranosyl (1→2)-α-L-arabinopyranoside | | HPLC≥98% | |  |
|  | A0816 | | Presapogenin CP4 | | HPLC≥98% | |  |
|  | A0798 | | Xanthiazone | | HPLC≥98% | |  |
| Xanthium sibiricum | A0799 | | Xanthiside | | HPLC≥98% | |  |
|  | A0809 | | Pungiolide A | | HPLC≥98% | |  |
|  | A0808 | | Xanthatin | | HPLC≥98% | |  |
|  | A0918 | | Atractyloside | | HPLC≥98% | |  |
|  | A0919 | | Carboxyatractyloside | | HPLC≥98% | |  |
|  | A0924 | | 1,3,5-tricaffeoylquinic acid | | HPLC≥98% | |  |
|  | A0730 | | Desacetyl asperulosidic acid | | HPLC≥98% | |  |
| Morinda officinalis How. | A1091 | | Nystose Trihydrate;  Fungitetraose Trihydrate | | HPLC≥98% | |  |
|  | A1305 | | 1,3-dihydroxy-2-methoxy-anthraquinone | | HPLC≥98% | |  |
|  | A1306 | | Rubiadin-1-methyl ether | | HPLC≥98% | |  |
|  | A1307 | | 2-hydroxy-1-methoxy-anthraquinone | | HPLC≥98% | |  |
|  | A1308 | | 3-hydroxy-1,2-dimethoxy-anthraquinone | | HPLC≥98% | |  |
|  | A1309 | | 2,5-dihydroxy-1-methoxy-anthraquinone | | HPLC≥98% | |  |
|  | A1310 | | 2-methyl anthraquinone | | HPLC≥98% | |  |
|  | A1311 | | 2-hydroxy-3-methyl antraquinone | | HPLC≥98% | |  |
|  | A1312 | | Rubiadin | | HPLC≥98% | |  |
|  | A1318 | | 2-hydroxymethyl3-hydroanthraquinone | | HPLC≥98% | |  |
|  | A1322 | | anthraquinone-2-carboxylic acid | | HPLC≥98% | |  |
|  | A0731 | | Jaceosidin | | HPLC≥98% | |  |
|  | A1415 | | 1F-fructofuranosylnystose | | HPLC≥98% | |  |
|  | A0717 | |  | | HPLC≥98% | |  |
| pseudolarix amabilis ( Nelsn ) Regd. | A0718 | | Pseudolaric acid A-O-β-D-glucopyranoside | |  | |  |
|  | A0719 | | Pseudolaric acid B-O-β-D-glucopyranoside | |  | |  |
|  | A0720 | | Pseudolaric Acid C | |  | |  |
| Dryopteris crassirhizoma Nakai. | A0542 | | Albaspidin AA | | HPLC≥98% | |  |
|  | A0543 | | Albaspidin AP | | HPLC≥98% | |  |
|  | A0553 | | Dryocrassin ABBA | | HPLC≥98% | |  |
|  | A0556 | | Filixic acid ABA | | HPLC≥98% | |  |
| Croton tiglium | A0534 | | Crotonoside | | HPLC≥98% | |  |
|  | A0818 | | 12-O-tetradecanoyl phorbol-13-acetate | | HPLC≥95% | |  |
|  | A0817 | | 12-O-Tiglylphorbol-13 –isobutyrate | | HPLC≥98% | |  |
|  | A0665 | | Glycerine trioleate | |  | |  |
|  | A0805 | | Crovatin | | HPLC≥98% | |  |
|  | A0806 | | Croverin | | HPLC≥98% | |  |
|  | A0807 | | Levatin | | HPLC≥98% | |  |
| Aralia elate(Miq.)Seem. | A0466 | | Araloside V | | HPLC≥98% | |  |
|  | A0467 | | Araloside X | | HPLC≥98% | |  |
|  | A0468 | | Araloside VII | | HPLC≥98% | |  |
| Heracleum nepalense D.Don | A0352 | | Byakangelicol | | HPLC≥98% | |  |
| oranges and tangerines | A0353 | | Neosperidin dihydrochalcone | | HPLC≥98% | |  |
|  | A0401 | | Eriocitrin | | HPLC≥98% | |  |
|  | A0584 | | Sinensetin | | HPLC≥98% | |  |
| Indigo | A0264 | | Indigo | | HPLC≥98% | |  |
| Eucommia ulmoides | A0268 | | Pinoresinol diglucoside | | HPLC≥98% | |  |
| N. chinensis Batal | A0276 | | Nardosinone | | HPLC≥98% | |  |
| Toddalia asiatica (L.)Lam | A0286 | | Chelerythrine Chloride | | HPLC≥98% | |  |
| Macleaya cordata | A0287 | | Sanguinarine | | HPLC≥98% | |  |
|  | A0288 | | Nitidine Chloride | | HPLC≥98% | |  |
| Zanthoxylum nitidum | A1226 | | Toddalolactone | | HPLC≥98% | |  |
|  | A1369 | | Dihydrochelerythrine | | HPLC≥98% | |  |
| Cephalotaxus fortunei | A0289 | | Homoharringtonine | | HPLC≥98% | |  |
| Devilpepper Root | A0291 | | Reserpine | | HPLC≥98% | |  |
| Pogostemon cablin | A0300 | | Patchouli alcohol | | GC≥98% | |  |
|  | A1360 | | Pogostone | | HPLC≥98% | |  |
| Smilax aristolochiaefolia Miller | A0485 | | Sarsasapogenin | | HPLC≥98% | |  |
|  | A0311 | | Menthol | | GC≥98% | |  |
| Herba Menthae Haplocalycis | A1086 | | Pulegone | | HPLC≥98% | |  |
|  | A1375 | | DL-Camphor | | HPLC≥98% | |  |
| A.scoparia Waldst.e Kit | A0314 | | Scoparone | | HPLC≥98% | |  |
|  | A0316 | | Brucine | | HPLC≥98% | |  |
| Strychnos nux vomica | A1053 | | Strychnine | | HPLC≥98% | |  |
| Bamboo leaf | A0324 | | Homoori entin | | HPLC≥98% | |  |
| Thesium chinense | A0325 | | astragalin | | HPLC≥98% | |  |
| Stemona tuberosa L. | A0326 | | tuberostemonine | | HPLC≥96% | |  |
| Curculigo glabrescens (Ridl.) Merr. | A0327 | | Curculigoside | | HPLC≥98% | |  |
| Curculigo orchioides Gaertn. | A0683 | | Orcinol glucosid | | HPLC≥98% | |  |
| Datura metm L | A0346 | | Scopolamine butylbromide | | HPLC≥98% | |  |
|  | A0347 | | 8-O-Acetylharpagide | | HPLC≥98% | |  |
| Ajuga decumbens Thunb | A1333 | | Ajugamacrin | | HPLC≥95% | |  |
|  | A1334 | | Ajuforrestin A | | HPLC≥98% | |  |
|  | A1335 | | Ajuforrestin B | | HPLC≥98% | |  |
| Phytolacca acinosa Roxb | A0351 | | Esculentoside A | | HPLC≥98% | |  |
|  | A0355 | | Agrimol B | | TLC | |  |
| Herba Agrimoniae | A1244 | | Agrimoniin | | HPLC≥98% | |  |
| Beesia calthifolia | A0356 | | Cimigenol-3-O-β-D-xylpyranoside | | HPLC≥98% | |  |
| Dracaena cochinchinensis | A0364 | | LOUREIRIN B | | HPLC≥98% | |  |
|  | A0366 | | Demethoxyaschantin | | HPLC≥98% | |  |
| Magnolia biondii Pamp | A1059 | | Magnolin | | HPLC≥98% | |  |
| Rhus succedanea L | A0368 | | Fisetin | | HPLC≥98% | |  |
| Aloe vera L | A0371 | | barbaloin | | HPLC≥98% | |  |
|  | A0378 | | Rubimaillin | | HPLC≥98% | |  |
| Madder | A1078 | | Purpurin | | HPLC≥98% | |  |
| Clinopod ium chinense ( Benth) O. Kuntze | A0379 | | Clinodiside A | | HPLC≥98% | |  |
| Pyrola incarnata | A0532 | | Monotropein | | HPLC≥98% | |  |
| Salvia SclareL. | A0381 | | Sclareol | | HPLC≥98% | |  |
| Daphne Korean Nakai | A0384 | | Daphnetin | | HPLC≥98% | |  |
| Tsoongiodendronodorum Chun | A0385 | | parthenolide | | HPLC≥98% | |  |
| Diospyros ebenum | A0391 | | Robustaside B | | HPLC≥98% | |  |
| Swertia cincta Burk | A0392 | | Sweroside | | HPLC≥98% | |  |
| HeliciaerratieaHook. | A0393 | | Helicid | | HPLC≥98% | |  |
| Huperzinaserrata(Thumb.)Trev | A0394 | | Huperzine-A | | HPLC≥98% | |  |
|  | A0400 | | Obtusifolin | | HPLC≥98% | |  |
| Cassia tora | A0417 | | aurantio-obtusin | | HPLC≥98% | |  |
|  | A1159 | | Chrysoobtusin | | HPLC≥98% | |  |
|  | A1160 | | Obtusin | | HPLC≥98% | |  |
|  | A1018 | | 8-Methyl Chrysophanol | | HPLC≥98% | |  |
|  | A1148 | | Chrysophanol triglucoside | | HPLC≥95% | |  |
|  | A1149 | | Chrysophanol-1-O-β-gentiobioside | | HPLC≥96% | |  |
|  | A1150 | | Isorubrofusarin-6-O-β-gentiobioside | | HPLC≥98% | |  |
|  | A1151 | | Rubrofusarin triglucoside | | HPLC≥98% | |  |
|  | A1152 | | Rubrofusarin-6-O-β-gentiobioside | | HPLC≥98% | |  |
|  | A1162 | | 1-Methyl Emodin | | HPLC≥98% | |  |
|  | A1163 | | 2-hydroxyl emodin-1-methyl ether | | HPLC≥98% | |  |
| Eriodictyon sp | A0402 | | Eriodictyol | | HPLC≥98% | |  |
| Linum usitatissimum L. | A0405 | | SDG ;  Secoisolariciresinol Diglucoside | | HPLC≥98% | |  |
|  | A0411 | | Momordin Ic | | HPLC≥98% | |  |
| Kochia scoparia(L.)Schrad | A1247 | | Oleanolic acid -3-O-glucosyl(1-2)xylyl(1-3)glucosiduronic acid | | HPLC≥98% | |  |
|  | A1248 | | 3-O-xylyl(1-3)glucosiduronic acid-oleanolic acid -28-O-glucoside | | HPLC≥98% | |  |
| Cirsium japonicum DC. | A0412 | | Pectolinarin | | HPLC≥98% | |  |
| Wintergreen | A0413 | | 2"-O-galloylhyperin | | HPLC≥98% | |  |
|  | A0415 | | Lycorine chloride | | HPLC≥98% | |  |
| Lycoris radiata | A1131 | | Galantamine Hydrobromide Lycoremine | | HPLC≥98% | |  |
| Cicer arietinum L. | A0419 | | Biochanin A | | HPLC≥99% | |  |
| Cocoa | A0421 | | Theobromine | | HPLC≥99% | |  |
| Vicia faba Linn | A0422 | | D-Pinitol | | HPLC≥95% | |  |
| HederanepalensisK.Kochvar.sinensis（Tobl.）Rehd | A0423 | | Hederagenin | | HPLC≥98% | |  |
|  | A1093 | | Hederacoside D | | HPLC≥98% | |  |
| Fagava zanthoxyloides Lam | A0424 | | 8-Methoxypsoralen | | HPLC≥98% | |  |
| Griffonia simplicifolia | A0430 | | 5-HTP | | HPLC≥98% | |  |
| corydalis sheareri S. Moore | A0433 | | (+)-Corynoline | | HPLC≥98% | |  |
| Siegesbeckia orientalis L. | A0436 | | Kirenol | | HPLC≥98% | |  |
| Alpinia officinarum Hance | A0437 | | Galangin;3,5,7-Trihydroxyflavone | | HPLC≥98% | |  |
| Lathy flaky crystal | A0438 | | (+)-Bicuculline | | HPLC≥98% | |  |
| Corydalis bungeana | A0440 | | Acetylcorynoline | | HPLC≥98% | |  |
| Filifolium sibiricum (L.) Kitam | A0451 | | Isorhamnetin-3-O-β-D-Glucoside | | HPLC≥98% | |  |
| Lagerstroemia speciosa | A0453 | | Corosolic acid | | HPLC≥98% | |  |
| Lupinus polyphyllus | A0454 | | Lupeol | | HPLC≥98% | |  |
| Dioscorea Nipponica | A0457 | | Diosgenin glucoside | | HPLC≥98% | |  |
| Dipsacus asperoides C. Y. Cheng et T .M. Ai. | A0464 | | Asperosaponin Ⅵ | | HPLC≥98% | |  |
|  | A0475 | | Diosmetin-7-O-β-D-glucopyranoside | | HPLC≥97% | |  |
| Chrysanthemum | A1344 | | Spiraeoside | | HPLC≥98% | |  |
| Thesium chinense Turcz | A0483 | | Kaempferol-3-O-glucorhamnoside | | HPLC≥98% | |  |
| Equisetum hiemale L. | A0484 | | Herbacetin; 3,4',5,7,8-pentahydroxyflavone | | HPLC≥98% | |  |
| Muskroot-like Semiaquilegia Root | A0487 | | Griffonilide | | HPLC≥96% | |  |
| Aster tataricus L. f. | A0490 | | Shionone | | HPLC≥98% | |  |
| Ocimum basilicumL. | A0493 | | Lysionotin | | HPLC≥98% | |  |
| Rhizoma Belamcandae | A0494 | | Irisflorentin | | HPLC≥98% | |  |
| Euonymus alatus | A0497 | | Epifriedelanol | | HPLC≥98% | |  |
| lstonia scholaris ( L . ) R. Bro. | A0498 | | Picrinine | | HPLC≥98% | |  |
| Helianthemumordosicum | A0496 | | Isoforskolin | | HPLC≥94% | |  |
| Saururus chinensis(Lour.) Baill. | A0501 | | Sauchinone | | HPLC≥98% | |  |
| Sunglo | A0503 | | Usnic acid | | HPLC≥98% | |  |
| Dendrobe | A0507 | | Dendrobine | | HPLC≥98% | |  |
|  | A1021 | | Erianin | | HPLC≥98% | |  |
| Lonicera macranthoides Hand.-Mazz. | A0523 | | Macranthoidin B | | HPLC≥98% | |  |
| Lonicara confusa DC | A0527 | | Dipsacoside B | | HPLC≥98% | |  |
|  | A1196 | | Macranthoidin A | | HPLC≥98% | |  |
|  | A1429 | | Ethyl caffeate | | HPLC≥98% | |  |
| I. indigotica Fort | A0529 | | Epigoitrin | | HPLC≥98% | |  |
| Plantago asiatica L | A0530 | | Plantamajoside | | HPLC≥98% | |  |
|  | A1158 | | 6-hydroxyl luteolin-7-O-glucoside | | HPLC≥95% | |  |
|  | A1161 | | Plantainoside D | | HPLC≥98% | |  |
|  | A1165 | | 10-hydroxy majoroside | | HPLC≥98% | |  |
|  | A1211 | | scutellarein-7-O-glucoside | | HPLC≥98% | |  |
|  | A1440 | | Plantagoside | | HPLC≥98% | |  |
| Engelhardtia roxburghiana Wall | A0533 | | Engeletin | | HPLC≥98% | |  |
| Cunninghamia lanceolata | A0536 | | Amentoflavone | | HPLC≥98% | |  |
| Pseudolarix kaempferi Gord | A0537 | | Pseudolaric Acid B | | HPLC≥98% | |  |
| L.heterophyllus Sweet | A0539 | | Leonurine hydrochloride | | HPLC≥98% | |  |
|  | A1024 | | Stachydrine | | HPLC≥98% | |  |
| Eucalyptus | A0549 | | β-Eudesmol | | HPLC≥98% | |  |
| Cinnamomum camphora (L.) Presl | A0563 | | Linalool | | HPLC≥93% | |  |
|  | A1355 | | vanillic acid | | HPLC≥98% | |  |
| Hypericum monogynum | A0576 | | Pseudohypericin | | HPLC≥98% | |  |
| Ilex rotunda Thunb | A0586 | | Pedunculoside | | HPLC≥98% | |  |
| Cyperus rotundus L | A0588 | | α-Cyperone | | HPLC≥98% | |  |
| Physochlaina infundibularis Kuang | A0594 | | Scopolamine hydrobromide | | HPLC≥98% | |  |
| Rhizoma Dioscoreae Bulbiferae | A0597 | | Diosbulbin B | | HPLC≥95% | |  |
| Callicarpa bodinieri | A0602 | | Poliumoside | | HPLC≥98% | |  |
| Palm | A0604 | | Palmitic acid | | HPLC≥98% | |  |
| PhellodendronchineseSchneid | A0606 | | Phellodendrine chloride | | HPLC≥98% | |  |
| Syringa oblata | A0612 | | (-)-Syringaresnol-4-O-β-D-apiofuranosyl-(1→2)-β-D-glucopyranoside | | HPLC≥98% | |  |
| Corydalis yanhusuo W.T.Wang | A0614 | | Protopine | | HPLC≥98% | |  |
| Corydalis decumbens (Thunb.) Pers | A0615 | | (R)-(+)-Corypalmine | | HPLC≥98% | |  |
| Peucedanumdecursivum(Miq.)Maxim | A0629 | | Nodakenin | | HPLC≥98% | |  |
|  | A1048 | | Decurson | | HPLC≥95% | |  |
|  | A1292 | | Oxypeucedanin hydrate | | HPLC≥98% | |  |
| Centipeda minima | A0636 | | Geraniin | | HPLC≥98% | |  |
| Celosia argentea L. | A0640 | | Daucosterol | |  | |  |
| Notopterygium forbesii Boiss | A0644 | | Notopterol | | HPLC≥98% | |  |
| Phyllan lhus mat shilllirae | A0647 | | Corilagin | | HPLC≥98% | |  |
| Kalimeris indica Sch-Bip | A0650 | | Syringic acid | | HPLC≥98% | |  |
| Sophora alopecuroides L. | A0651 | | Sophoridine | | HPLC≥96.50% | |  |
| Stephania japonica （Thunb.） Miers | A0653 | | Cepharanthine | | HPLC≥98% | |  |
|  | A1443 | | Cepharanthine hydrochloride | | HPLC≥98% | |  |
| Peganum Harmala Genus | A0656 | | Harmine hydrochloride | | HPLC≥98% | |  |
| Boswellia sacra | A0658 | | α-Boswellic acid | | HPLC≥98% | |  |
| Torikabuto | A0662 | | Jolkinolide B | | HPLC≥98% | |  |
| Walnut | A0663 | | 28-demethyl -β-amyrone | | HPLC≥98% | |  |
|  | A1376 | | Juglone | | HPLC≥98% | |  |
| Papaya | A0680 | | Lycopene | |  | |  |
| Ficus microcarpa L.f. | A0681 | | Isovitexin | | HPLC≥98% | |  |
| Ardisia crispa | A0686 | | Ardisiacrispin A | | HPLC≥98% | |  |
| Eupatorium odoratumL | A0687 | | Pectolinarigenin | | HPLC≥98% | |  |
| Cyathula officinalis Kuan | A0699 | | Cyasterone | | HPLC≥98% | |  |
| Albizzia julibrissin Durazz | A0700 | | Echinocystic acid | | HPLC≥98% | |  |
| Arachis hypogaea L. | A0707 | |  | | HPLC≥98% | |  |
|  | A0709 | |  | |  | |  |
| Trigonella foenum-graecum L. | A0721 | | 4-Hydroxyisoleucine | | HPLC≥98% | |  |
| Rubus suavissimus S. Lee | A0726 | | Rubusoside | | HPLC≥98% | |  |
| Apium graveolens L. | A0729 | | Apioside | | HPLC≥98% | |  |
| Artemisia argyi Levl. et Vant. | A0732 | | Eupatilin | | HPLC≥98% | |  |
| Berberis thunbergii DC. | A0736 | | Berbamine | |  | |  |
| Paeonia lactiflora Pall. | A0737 | | 1,2,3,4,6-pentagalloylglucose | | HPLC≥98% | |  |
|  | A1232 | | Lactiflorin | | HPLC≥98% | |  |
|  | A1233 | | Benzoylalbiflorin | | HPLC≥98% | |  |
|  | A1237 | | Galloylpaeoniflorin | | HPLC≥95% | |  |
|  | A1238 | | 4-O-galloylalbiflorin | | HPLC≥98% | |  |
|  | A1239 | | 6'-O-galloylalbiflorin | | HPLC≥95% | |  |
|  | A1298 | | Paeoniflorin sulfite | | HPLC≥98% | |  |
| Crotalaria ferruginea Grah | A0738 | | Octacosanol | |  | |  |
| Edgew orthia chrysantha Lindl. | A0740 | | Tiliroside | | HPLC≥98% | |  |
| Euphorbia soongarica Boiss | A0741 | | Ellagic acid | | HPLC≥98% | |  |
|  | A0952 | | Ingenol | | HPLC≥98% | |  |
| Pterocarpus indicus | A0752 | | Pterostilbene | | HPLC≥98% | |  |
| Polygonum aviculare L. | A0757 | | Avicularin | | HPLC≥98% | |  |
| Asarum sieboldii Miq. | A0759 | | L-Asarinin/ (-)-asarinin | | HPLC≥98% | |  |
|  | A1140 | | α-Asarone | | HPLC≥98% | |  |
|  | A1141 | | β-Asarone | | HPLC≥98% | |  |
| Pharbitis nil (Linn.)Choisy | A0765 | | 15-hydroxy-dehydroabietic acid | | HPLC≥95% | |  |
| Apocynaceae | A0766 | | taraxasteryl acetate | | HPLC≥80% | |  |
| Nerium oleander L. | A0785 | | Oleandrin | | HPLC≥98% | |  |
|  | A0965 | | Adynerin | | HPLC≥98% | |  |
| Clematis terniflora DC. | A0770 | | aurantiamide acetate | | HPLC≥98% | |  |
| Pinus cerebra | A0778 | | Pinocembrin | | HPLC≥98% | |  |
| Daphne genkwa Sieb. et Zucc. | A0780 | | Genkwanin | | HPLC≥98% | |  |
| Thalictrum polygamun Muhl. | A0783 | | Berberrubine | | HPLC≥98% | |  |
| Berberis phanera Schneid | A0794 | | Dihydroberberine | | HPLC≥98% | |  |
| Berberisthunbergiicv.atropurpurea | A0796 | | Berberine Sulfate | | HPLC≥98% | |  |
| Spinacia oleracea | A0797 | | Folic acid | | HPLC≥98% | |  |
| Jatropha curcas L. | A0810 | | Jatropholone B | | HPLC≥98% | |  |
| Berneuxia thibetica | A0812 | | 11-hydroxy-sugiol | | HPLC≥98% | |  |
| Garcinia hanburyi Hook. f. | A0820 | | Gambogic acid | | HPLC≥98% | |  |
| composition | A0787 | | Olaquindox | | HPLC≥98% | |  |
|  | A0903 | | Pregnenolone | | HPLC≥98% | |  |
|  | A0902 | | Apo-12’-lycopenal | | HPLC≥95% | |  |
|  | A1074 | | Fumaric acid；Lichenic acid；(E)-2-Butenedioic acid | | HPLC≥98% | |  |
|  | A1075 | | Tartaric acid | | HPLC≥98% | |  |
|  | A1076 | | 2,5-dihydroxybenzoic acid | | HPLC≥98% | |  |
|  | A1077 | | Citric acid hydrate | | HPLC≥98% | |  |
|  | A1088 | | p-hydroxyl phenylpropanol | | HPLC≥98% | |  |
|  | A1089 | | 3-(3-hydroxyl phenyl)propanol | | HPLC≥98% | |  |
|  | A1098 | | Etoposide | | HPLC≥98% | |  |
|  | A1145 | | Methyl 3,4-dihydroxybenzoate；  Protocatechuic acid methyl ester | | HPLC≥98% | |  |
|  | A1146 | | 4-Methylcatechol；  3,4-dihydroxyl toluene | | GC≥98% | |  |
|  | A1352 | | Methyl gallate | | HPLC≥98% | |  |
|  | A1168 | | Ethyl gallate | | HPLC≥98% | |  |
|  | A1169 | |  | | ≥98% | |  |
|  | A1348 | | Benzoic acid | | HPLC≥98% | |  |
|  | A1373 | | Salicylic acid | | HPLC≥98% | |  |
|  | A1382 | | 4-Hydroxybenzyl Alcohol | | HPLC≥98% | |  |
|  | A1387 | | Pyrogallic Acid | | HPLC≥98% | |  |
| Ramification | A0685 | |  | | HPLC≥98% | |  |
| chrysophoron | A0657 | | Succinic acid | | HPLC≥99% | |  |
| Cordyceps militaris | A0682 | | Cordycepin | | HPLC≥98% | |  |
| Ramification | A0500 | | 4'-Demethylepipodophyllotoxin | | HPLC≥98% | |  |
| hydrolysate | A0472 | | D(+)-Glucose | | HPLC≥98% | |  |
| composition | A0459 | | Homovanillic acid | | TLC≥99% | |  |
| cow bezoar | A0377 | | Bilirubin | | HPLC≥94% | |  |
| composition | A0332 | | Diosimin | | HPLC≥98% | |  |
|  | A0308 | | Fructose | | HPLC≥96% | |  |
| Honey | A0427 | | L-Carnitine inner salt | | 98% | |  |
|  | A1414 | | 1-Kestose | | HPLC≥98% | |  |
| The root of germinating barley seed | A0179 | | Hordenine | | HPLC≥98% | |  |
| composition | A0231 | | Lovastatin | | HPLC≥98% | |  |
| composition | A1224 | | Guanosine | | HPLC≥98% | |  |
| Musk | A0128 | | Muscone | | GC≥98% | |  |
| Chellotin | A0100 | | Glucosamine Hydrochloride | | HPLC≥98% | |  |
| composition | A0099 | | Glucosamine sulfate | | HPLC≥98% | |  |
| Shark bone | A0097 | | Chondroitine sulfate | | HPLC≥98% | |  |
| composition | A0064 | | Coenzyme Q10 | | HPLC≥98% | |  |
| Aristolochia debilis | A0849 | | Aristolochic acid A | | HPLC≥98% | |  |
| Alsophila spinulosa | A0850 | | Piceatannol | | HPLC≥98% | |  |
| moleplant seed | A0901 | | Euphorbia factor L1 | | HPLC≥97% | |  |
| oriental wormwood | A0931 | | Scopoletin | | HPLC≥98% | |  |
| Arte-mida sacrorum | A0932 | | Isoscopoletin | | HPLC≥98% | |  |
| Boswellia carterii Birdw | A0940 | | β-demonic acid;  β-elemonic acid | | HPLC≥98% | |  |
| Coleus forskohlii | A0937 | | Forskolin | | HPLC≥97% | |  |
| flos farfarae | A0956 | | Tussilagone | | HPLC≥98% | |  |
| The seeds of cruciferae radish | A0961 | | Sinapine thiocyanate | | HPLC≥98% | |  |
| Artemisia annua linn | A0962 | | Artemisetin | | HPLC≥98% | |  |
| Cortex Periplocae Radicis | A1012 | | Periplocoside;  Periplocin | | HPLC≥98% | |  |
|  | A1013 | | Periplogenin | | HPLC≥98% | |  |
|  | A1014 | | Periplocymarin | | HPLC≥98% | |  |
|  | A1029 | | Periplogenin 3-O-β -glucopyranosyl- (1-4)-β- sarmentopyranoside | | HPLC≥95% | |  |
|  | A1030 | | Perisesaccharide B | | HPLC≥98% | |  |
|  | A1031 | | Perisesaccharide C | | HPLC≥98% | |  |
|  | A1032 | | Acetyl Perisesaccharide C | | HPLC≥98% | |  |
|  | A1033 | | Periplocoside M | | HPLC≥98% | |  |
|  | A1034 | | Periplocoside N | | HPLC≥98% | |  |
| Acanthopanar gracilistμlusW.W.Smith | A1103 | | Kaurenoic acid | | HPLC≥98% | |  |
| Hydrangea paniculata Sieb | A1010 | | Skimmin | | HPLC≥98% | |  |
| Amomum kravanh Pierre ex Gagnep. | A1072 | | Alnustone | | HPLC≥98% | |  |
| Adiantum capillus-veneris L. | A1073 | | Quercetin 3-O-β-D-glucuronide | | HPLC≥98% | |  |
| Eclipta prostrata | A1028 | | Ecliptasaponin A | | HPLC≥98% | |  |
| Rabdosia amethystoides (Benth.) Hara | A1057 | | Maoecrystal A | | HPLC≥95% | |  |
| Tribulus terrestris L | A1058 | | Hecogenin | | HPLC≥95% | |  |
|  | A1256 | | Quercetin 3-O-β-D-glucose-7-O-β-D-gentiobioside | | HPLC≥98% | |  |
|  | A1437 | | (25R)-Spirost-4-en-3,12-dion | | HPLC≥98% | |  |
| Rumex madaio MakinoR. daiwoo Makino | A1047 | | Rhapontin | | HPLC≥98% | |  |
| Aquilaria agallocha | A1039 | | Agarotetrol | | HPLC≥98% | |  |
| Clerodendranthus spicatus (Thunb.) C. Y. Wu | A1049 | | Isosinensetin | | HPLC≥98% | |  |
| Sarcandra glabra (Thunb.) Nakai | A1070 | | Isofraxidin | | HPLC≥98% | |  |
| FewflowerLysionotus | A1050 | | Lysionotin | | HPLC≥98% | |  |
| Willow | A0130 | | D-(−)-Salicin | | HPLC≥98% | |  |
| Trichosanthes kirilowii Maxim | A1115 | | 3,29-Dibenzoyl karounitriol | | HPLC≥98% | |  |
| Trachelospermum jasminoides (Lindl.) Lem. | A1134 | | Tracheloside | | HPLC≥98% | |  |
| Panax pseudo-ginseng | A1135 | | Chikusetsusaponin IVa | | HPLC≥98% | |  |
|  | A1221 | | Araloside A | | HPLC≥98% | |  |
|  | A1223 | | L-Fucose | | HPLC≥98% | |  |
| Isatidis Folium | A1147 | | Indirubin | | HPLC≥97% | |  |
|  | A1331 | | Isoscoparin | | HPLC≥97% | |  |
|  | A1332 | | Isoscoparin-2''-O-glucoside | | HPLC≥97% | |  |
| Semen Impatientis balsamina L. | A1138 | | Hosenkoside A | | HPLC≥98% | |  |
|  | A1139 | | Hosenkoside K | | HPLC≥98% | |  |
| Areca catechu L | A1095 | | Arecoline | | HPLC≥98% | |  |
|  | A1096 | | Arecoline hydrobromide | | HPLC≥98% | |  |
| Salvia plebeia | A1000 | | Hispidulin | | HPLC≥98% | |  |
|  | A1001 | | Homoplantaginin | | HPLC≥98% | |  |
| Veratrum | A0938 | | Jervine | | HPLC≥98% | |  |
|  | A0939 | | Veratramine | | HPLC≥98% | |  |
| Brassica oleracea L.var.italic Planch. | A1170 | | Glucoraphanin | | HPLC≥98% | |  |
|  | A1171 | | Sulforaphane | | HPLC≥97% | |  |
| Aristolochia debilis Sieb. et Zucc. | A1225 | | Aristolone | | HPLC≥98% | |  |
| Coreopsis tinctoria | A1219 | | Marein | | HPLC≥98% | |  |
|  | A1220 | | Okanin | | HPLC≥98% | |  |
|  | A1313 | | Coreoside B | | HPLC≥95% | |  |
|  | A1300 | | isookanin | | HPLC≥98% | |  |
|  | A1303 | | Quercetagitrin | | HPLC≥98% | |  |
|  | A1315 | | Flavanomarein | | HPLC≥95% | |  |
|  | A1418 | | Coreopsis tinctoria polysacharide | | HPLC≥98% | |  |
| Coix lacryma-jobi L.var.mayuen(Roman.)Stap | A1185 | | Coix Seed oil | |  | |  |
| Amomum villosum Lour. | A1186 | | bornyl acetate | | GC≥97% | |  |
| Tamarindus indica | A1249 | | 7, 4’-dihydroxyl flavone | | HPLC≥96% | |  |
|  | A1250 | | Dihydrokaempferol | | HPLC≥98% | |  |
|  | A1251 | | chrysoeriol 7-O-glucoside | | HPLC≥98% | |  |
| Bletilla striata(Thunb.) Reichb.f. | A1257 | | Gymnoside IX | | HPLC≥98% | |  |
|  | A1258 | | Gymnoside VII | | HPLC≥98% | |  |
|  | A1259 | | 4,7-dihydroxy-2-methoxy-9,10-dihydrophenanthrene | | HPLC≥98% | |  |
|  | A1260 | | Batatasin III | | HPLC≥98% | |  |
|  | A1267 | | Blestriarene A | | HPLC≥98% | |  |
|  | A1299 | | Blestriarene B | | HPLC≥96% | |  |
|  | A1268 | | 1-(4-hydroxybenzyl)-4-methoxy-9,10-dihydropenanthrene-2,7-diol | | HPLC≥98% | |  |
|  | A1269 | | 3’,5-dihydroxy-2-(4-hydroxybenzyl)3-methoxybibenzyl | | HPLC≥98% | |  |
|  | A1270 | | 2,7-dihydroxy-3,4-dimethoxyphenanthrene | | HPLC≥98% | |  |
|  | A1302 | | Gymconopin C | | HPLC≥98% | |  |
|  | A1301 | | Monbarbatain A | | HPLC≥98% | |  |
|  | A1304 | | 1-(4-hydroxybenzyl)-4-methoxypenanthrene-2,7-diol | | HPLC≥95% | |  |
|  | A1378 | | Militarine | | HPLC≥98% | |  |
|  | A1379 | | Dactylorhin A | | HPLC≥98% | |  |
| Narcissus tazetta L. var. chinensis Roem. | A1285 | | Narciclasine | | HPLC≥98% | |  |
|  | A1286 | | 7-deoxynarciclasine | | HPLC≥98% | |  |
| Cyclocarya paliurus. | A1287 | | alpha-Amyrin | | HPLC≥96% | |  |
|  | A1288 | | beta-Amyrin | | HPLC≥96% | |  |
|  | A1289 | | alpha-Amyrin acetate | | HPLC≥98% | |  |
|  | A1290 | | beta-Amyrin acetate | | HPLC≥98% | |  |
| Abrus precatorius L. | A1291 | | cirsimarin | | HPLC≥98% | |  |
| Lilium brownii var. viridulum | A1346 | | Regaloside A | | HPLC≥98% | |  |
|  | A1347 | | Regaloside B | | HPLC≥98% | |  |
| Rosa sp. | A1357 | | Casuarictin | | HPLC≥98% | |  |
|  | A1359 | | Eugenin | | HPLC≥98% | |  |
| Comanthosphace japonica (Miq) S Moore | A1358 | | Comanthoside A | | HPLC≥98% | |  |
| Humulus lupulus L. | A1377 | | Xanthohumol | | HPLC≥98% | |  |
| Rosa canina/Rosa rugosa | A1381 | | Rosamultin | | HPLC≥98% | |  |
| Angelica dahurica (Fisch. ex Hoffm.) Benth. et Hook. f. ex Franch. et Sav | A1397 | | Phellopterin | | HPLC≥98% | |  |
| Celastrus orbiculatus Thunb | A1401 | | 3-O-Acetyloleanolic acid | | HPLC≥98% | |  |
|  | A1402 | | pristimerin | | HPLC≥98% | |  |
| AraliacontinentalisKitag. | A1403 | | Continentalic acid | | HPLC≥98% | |  |
|  | A1444 | | Febrifugine | | HPLC≥98% | |  |
