# Supplementary figures and images for: Enhancing platinum-based chemotherapy efficacy and safety through combination therapy-mediated remodeling of autophagic homeostasis in gastric cancer
Source: Cell Death Dis. 2026 Apr 22;17(1):532. doi: 10.1038/s41419-026-08703-3 (PMC13234414; doi:10.1038/s41419-026-08703-3)

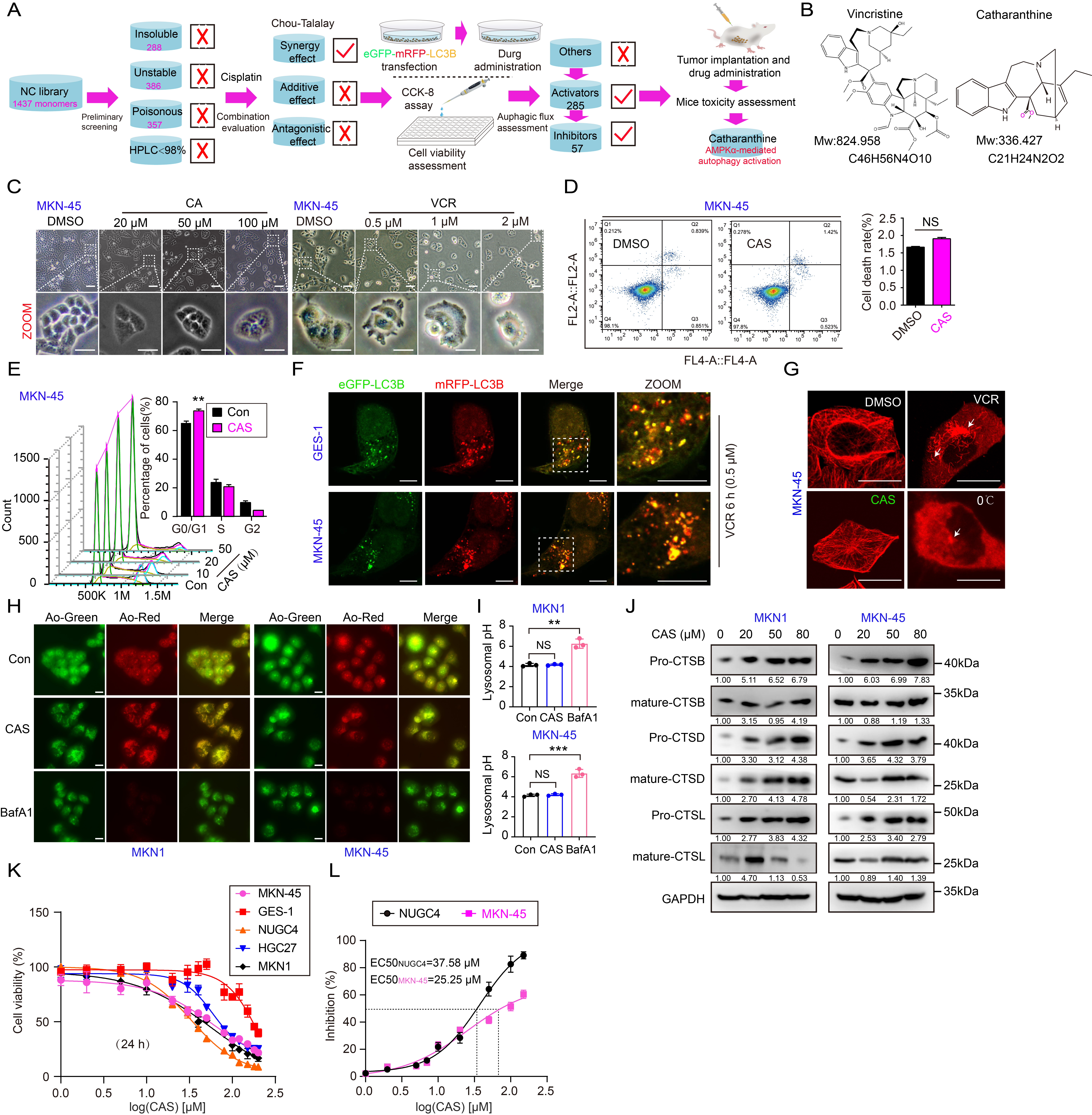

Supplement: Supplementary file 3 — Fig. S1 Discovery of CAS as a specific autophagic flux inhibitor distinct from VCR. [file 41419_2026_8703_MOESM3_ESM.jpg]

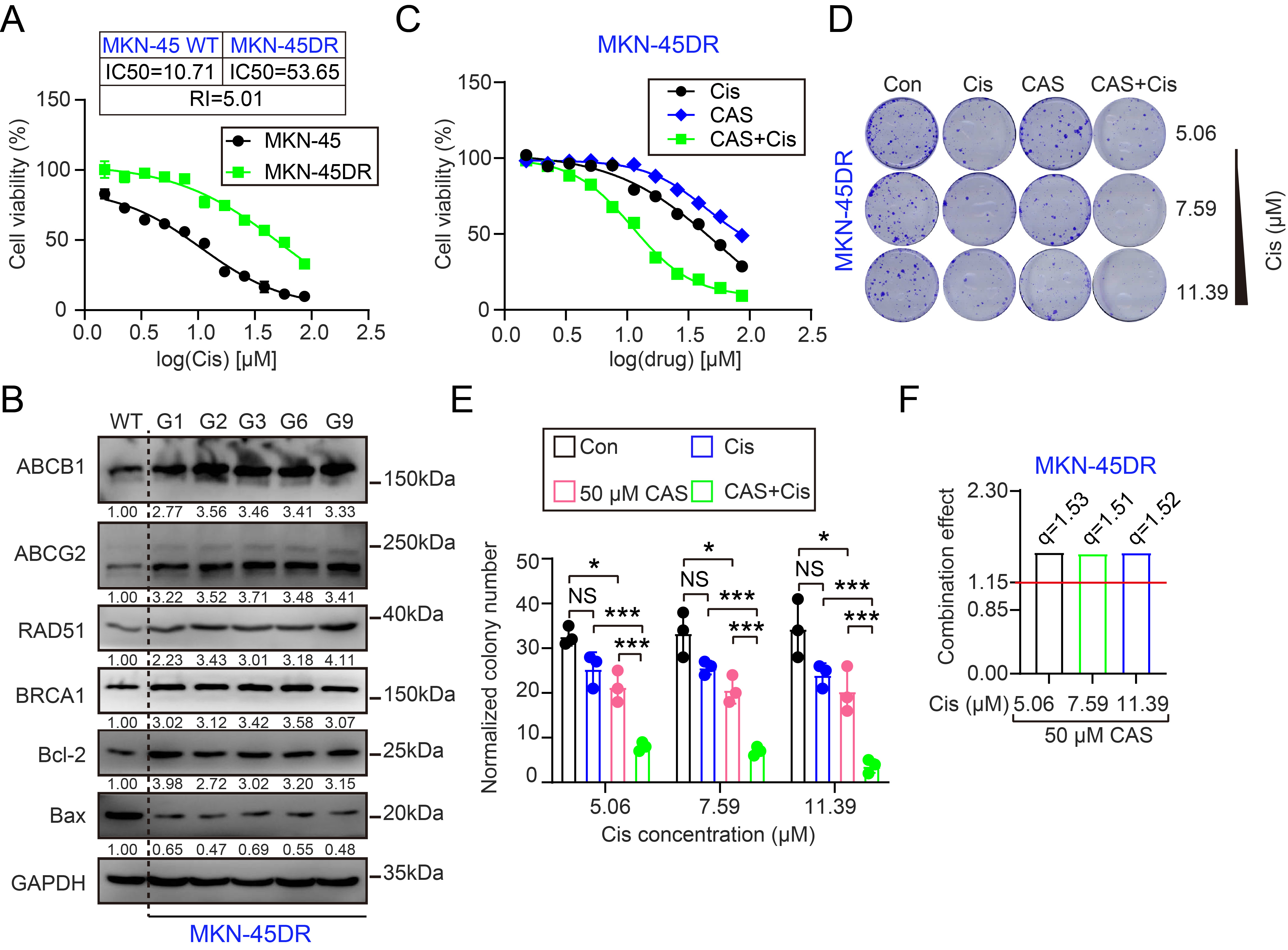

Supplement: Supplementary file 4 — Fig.S2 Cis-CAS combination acts synergistically to inhibit the proliferation of cisplatin-resistant cancer cells. [file 41419_2026_8703_MOESM4_ESM.jpg]

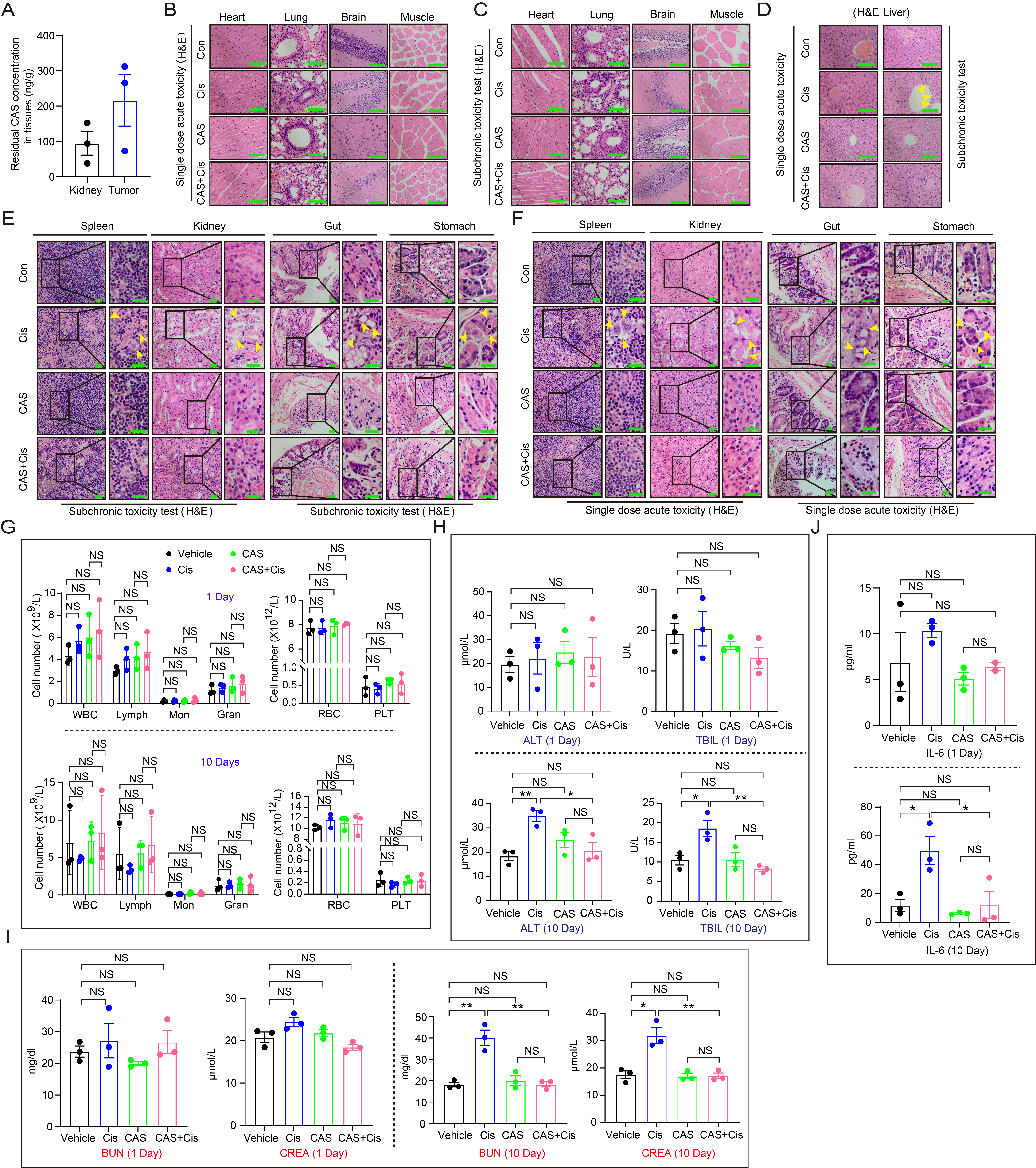

Supplement: Supplementary file 5 — Fig.S3 Cis-CAS combination alleviates the individual toxicity induced by Cis chemotherapy in mice. [file 41419_2026_8703_MOESM5_ESM.jpg]

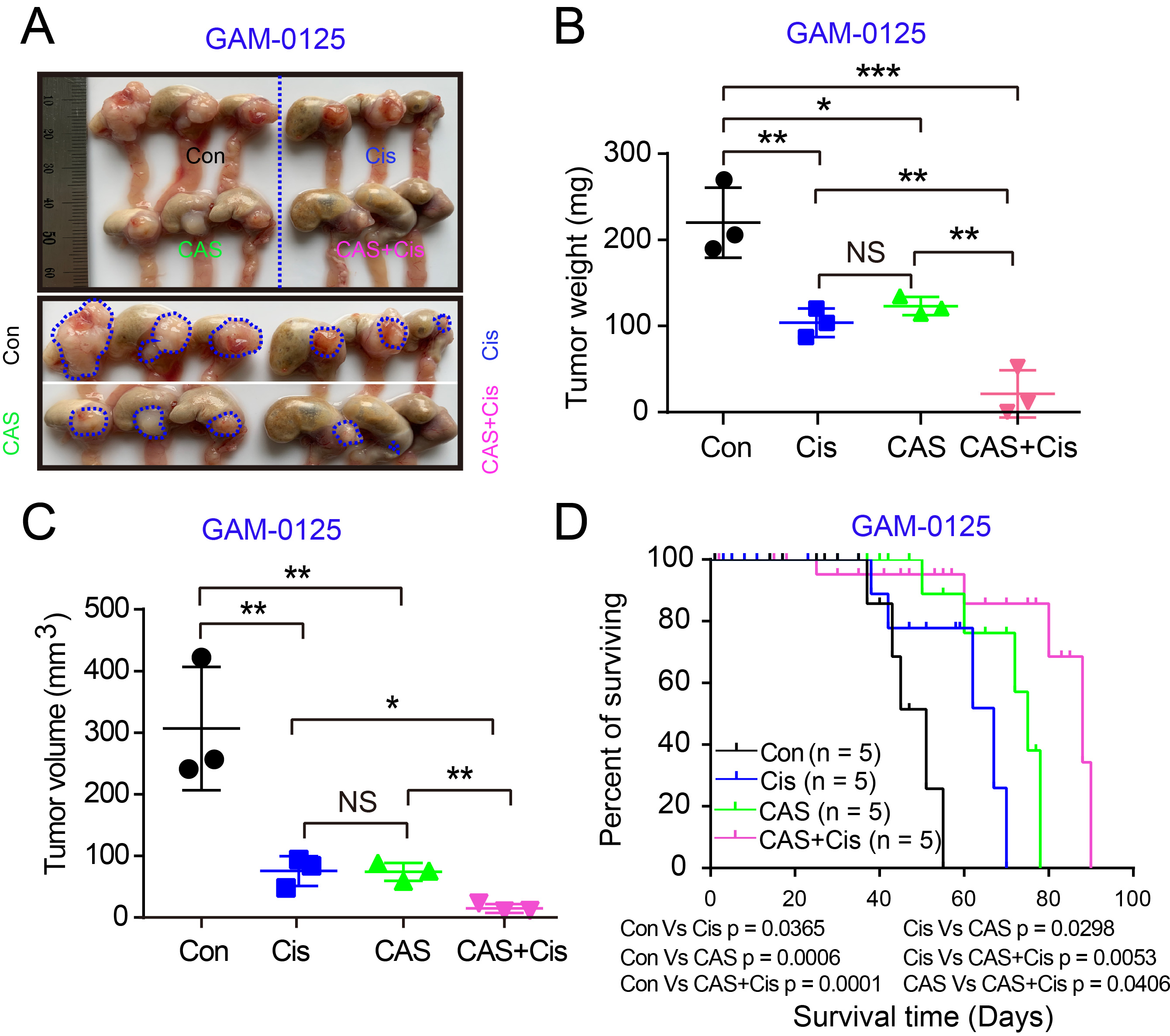

Supplement: Supplementary file 6 — Fig.S4 Combinatorial therapy prolongs tumor-bearing mice survival in PDX models. [file 41419_2026_8703_MOESM6_ESM.jpg]

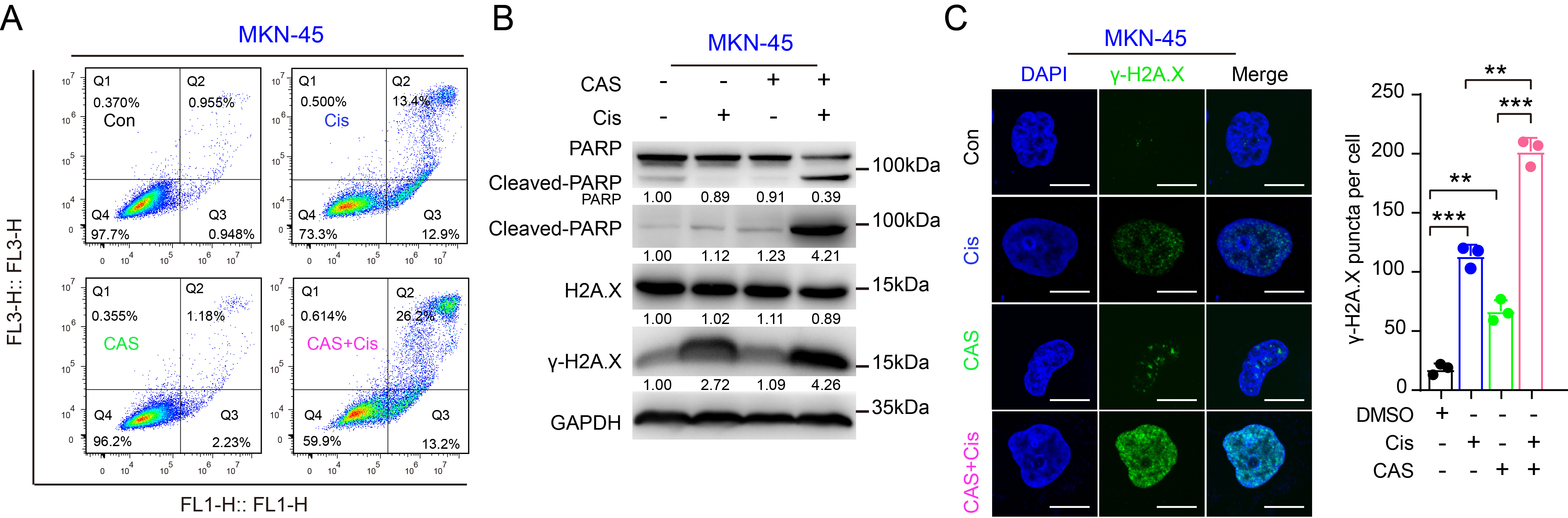

Supplement: Supplementary file 7 — Fig.S5 Cis-CAS combination suppresses tumor growth via DNA damage-mediated apoptosis. [file 41419_2026_8703_MOESM7_ESM.jpg]

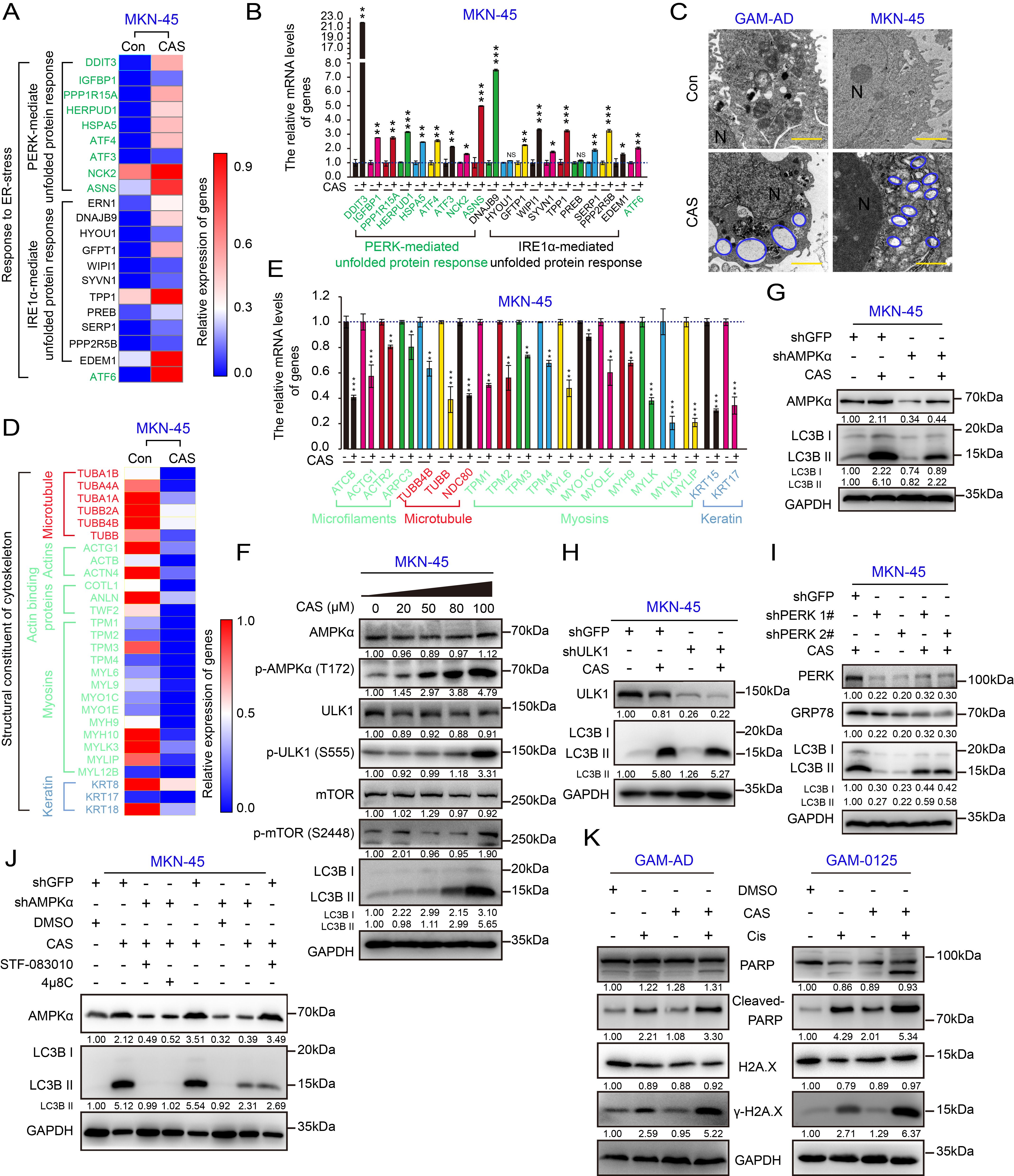

Supplement: Supplementary file 8 — Fig. S6 CAS is a newly ERS/AMPKα-dependent autophagy activator. [file 41419_2026_8703_MOESM8_ESM.jpg]

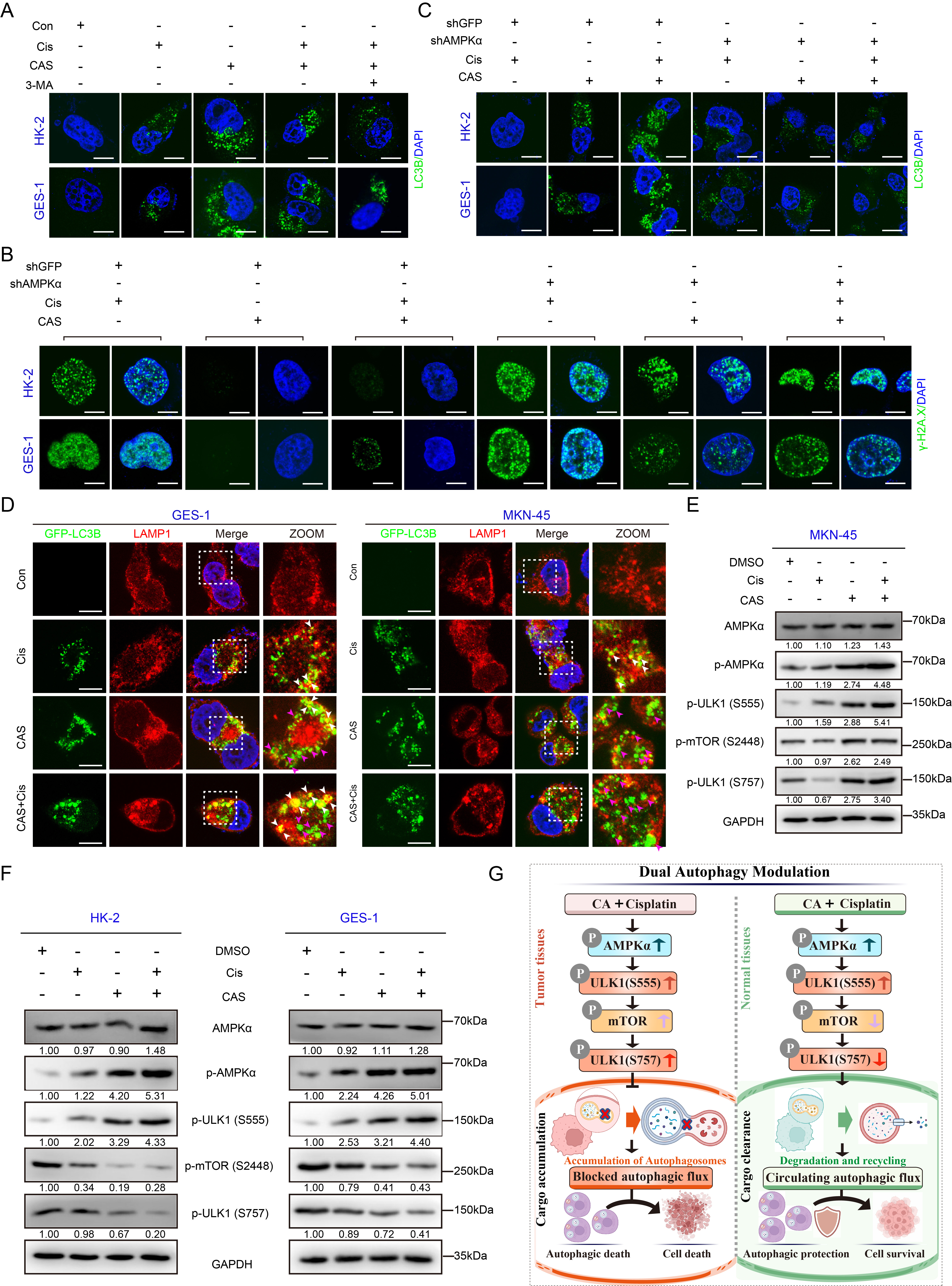

Supplement: Supplementary file 9 — Fig.S7 CAS alleviates cisplatin-induced toxicity by activating AMPKα-mediated protective autophagy. [file 41419_2026_8703_MOESM9_ESM.jpg]
