# Supplementary material for: Enhancing platinum-based chemotherapy efficacy and safety through combination therapy-mediated remodeling of autophagic homeostasis in gastric cancer
Source: Cell Death Dis. 2026 Apr 22;17(1):532. doi: 10.1038/s41419-026-08703-3 (PMC13234414; doi:10.1038/s41419-026-08703-3)
Supplement: Supplementary file 10 — Original western blots [file 41419_2026_8703_MOESM10_ESM.pdf]

Figure 5C

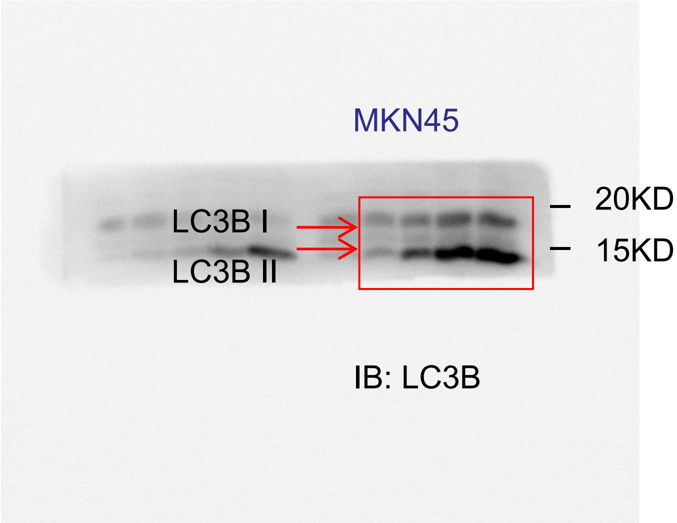
MKN45
LC3B I
LC3B II
20KD
15KD
IB: LC3B

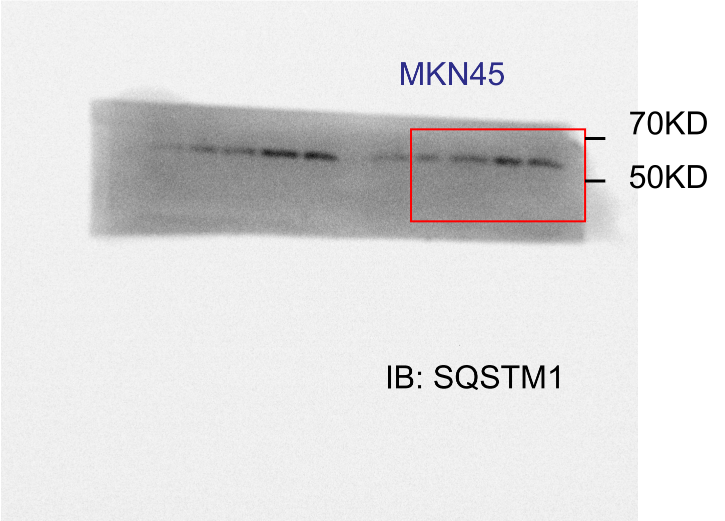
MKN45
70KD
50KD
IB: SQSTM1

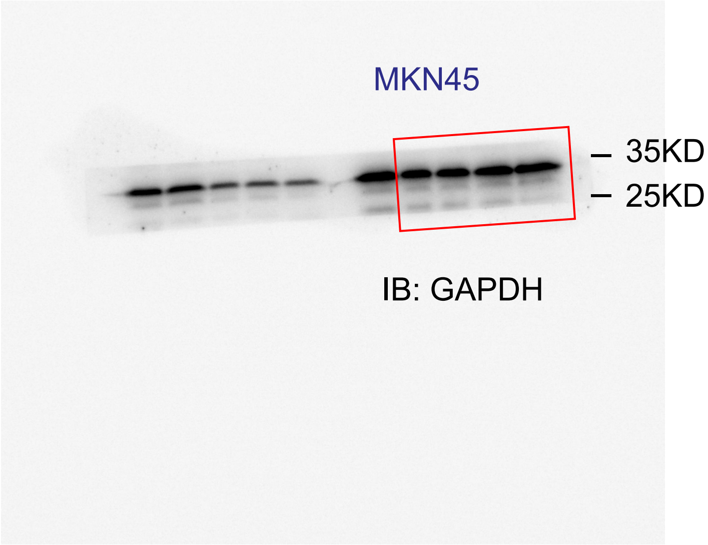
MKN45
35KD
25KD
IB: GAPDH

Figure 5J

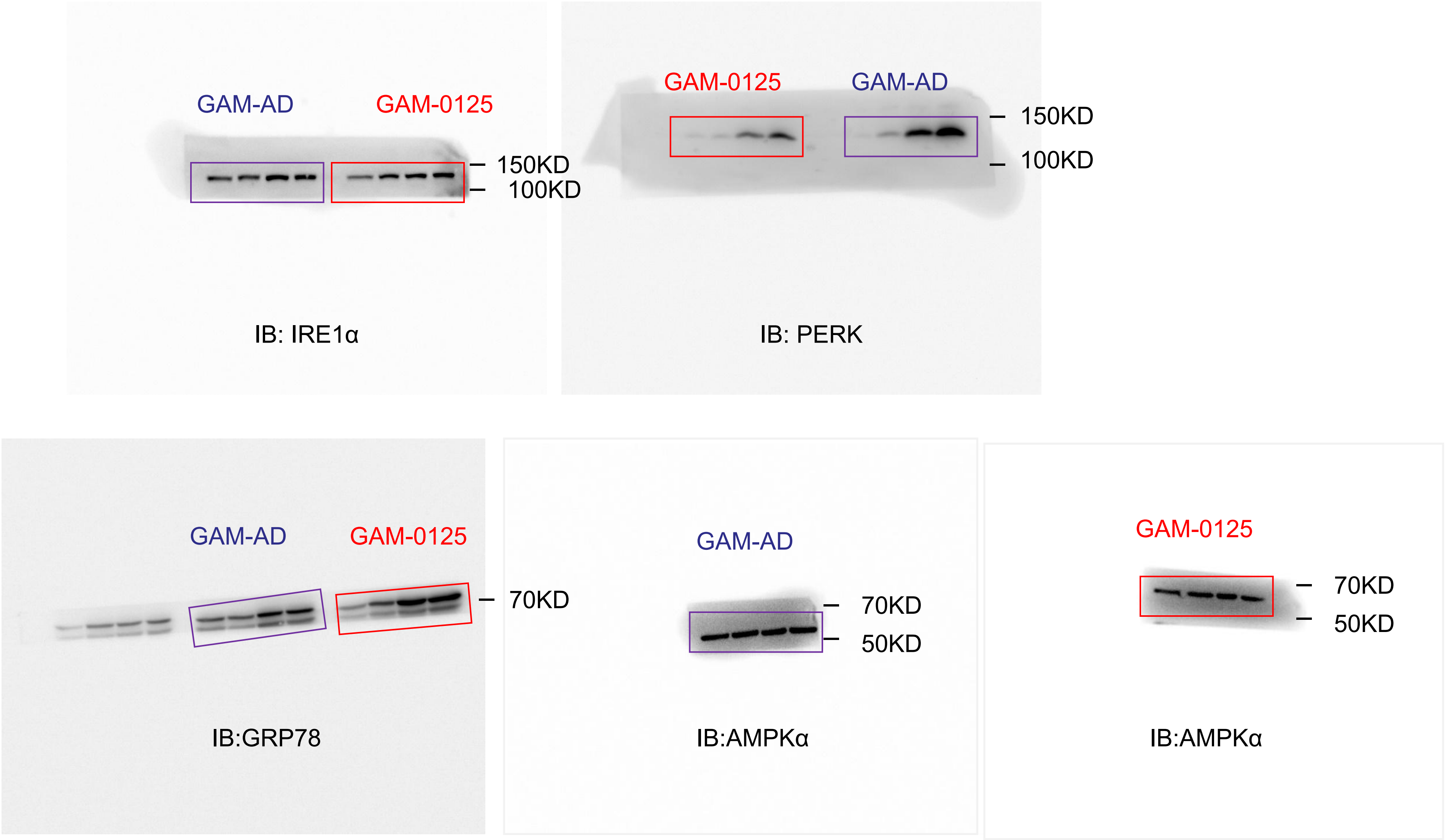

GAM-AD
GAM-0125
150KD
100KD
IB: IRE1α
GAM-0125
GAM-AD
150KD
100KD
IB: PERK
GAM-AD
GAM-0125
70KD
IB:GRP78
GAM-AD
70KD
50KD
IB:AMPKα
GAM-0125
70KD
50KD
IB:AMPKα

Figure 5J

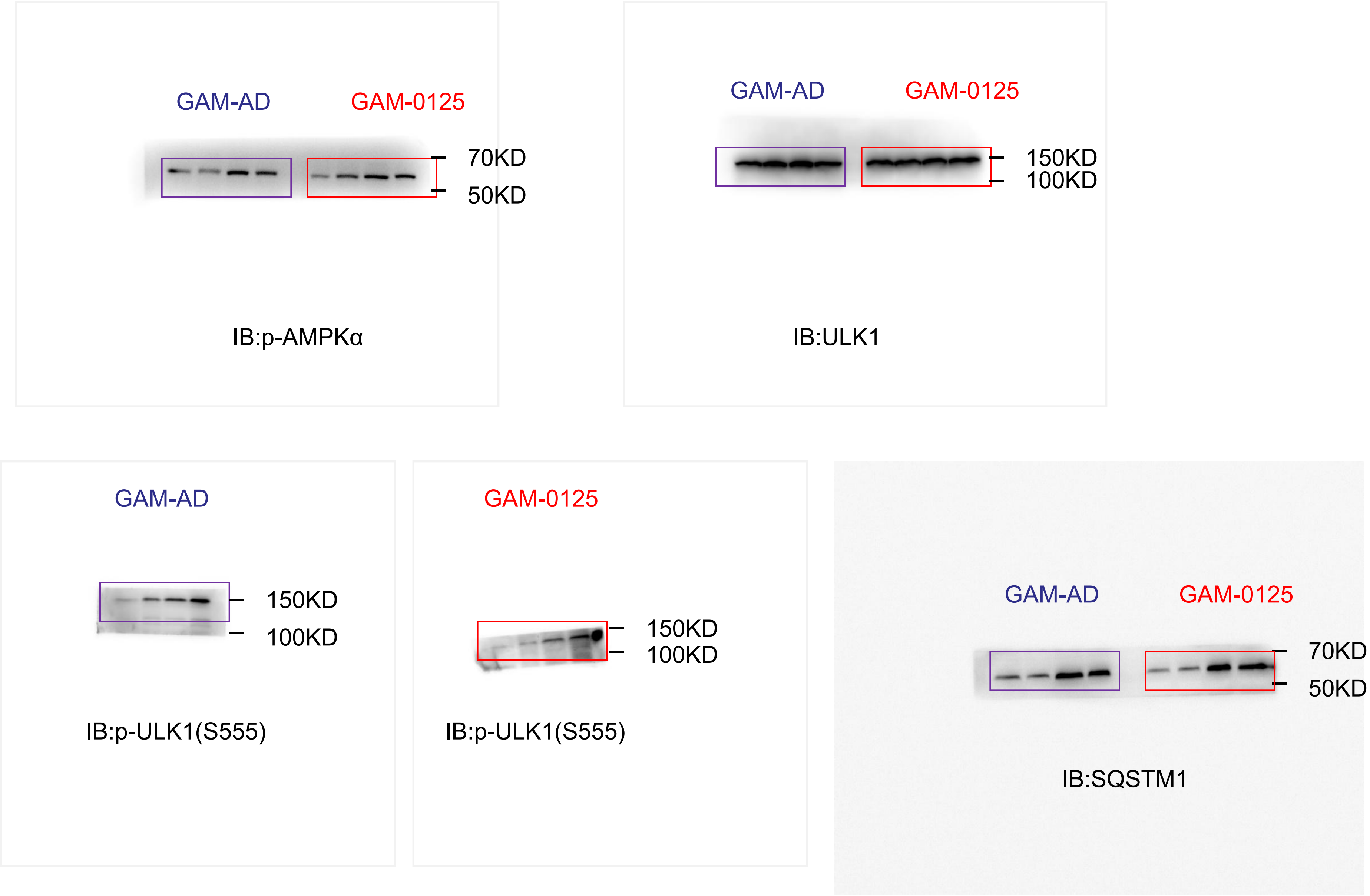
GAM-AD
GAM-0125
70KD
50KD
IB:p-AMPKα
GAM-AD
GAM-0125
150KD
100KD
IB:ULK1
GAM-AD
150KD
100KD
IB:p-ULK1(S555)
GAM-0125
150KD
100KD
IB:p-ULK1(S555)
GAM-AD
GAM-0125
70KD
50KD
IB:SQSTM1

Figure 5J

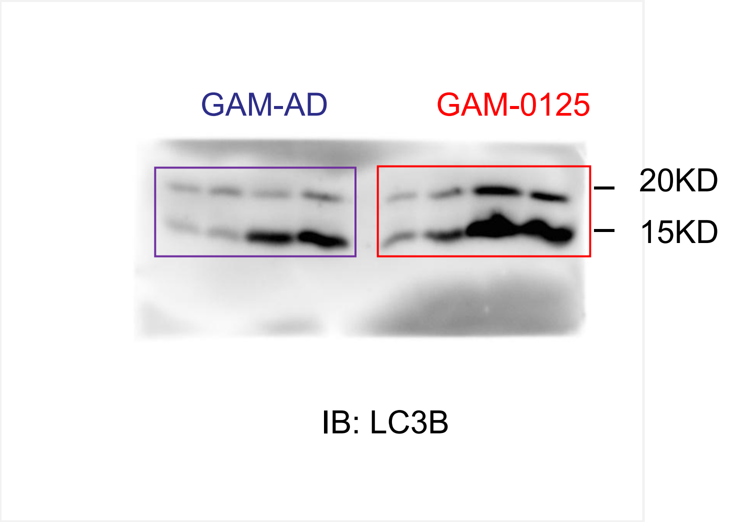
GAM-AD
GAM-0125
20KD
15KD
IB: LC3B

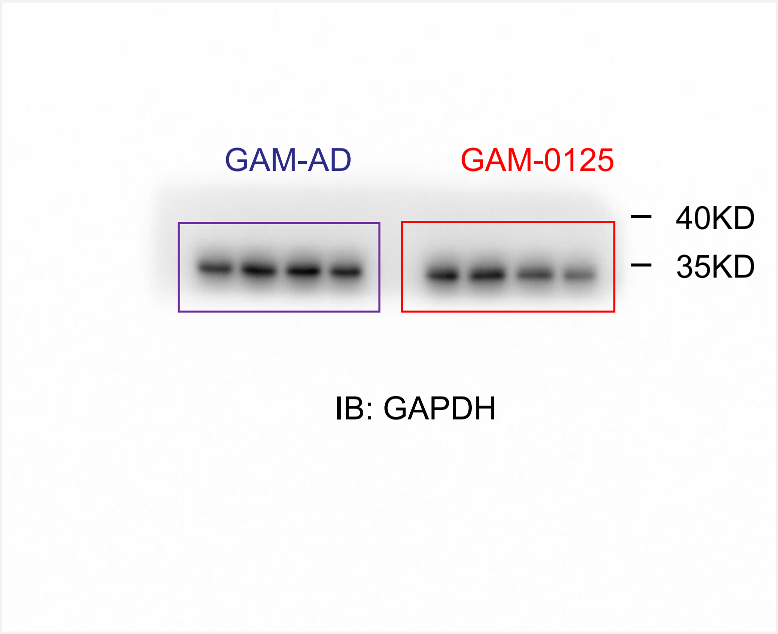
GAM-AD
GAM-0125
40KD
35KD
IB: GAPDH

Figure 6A

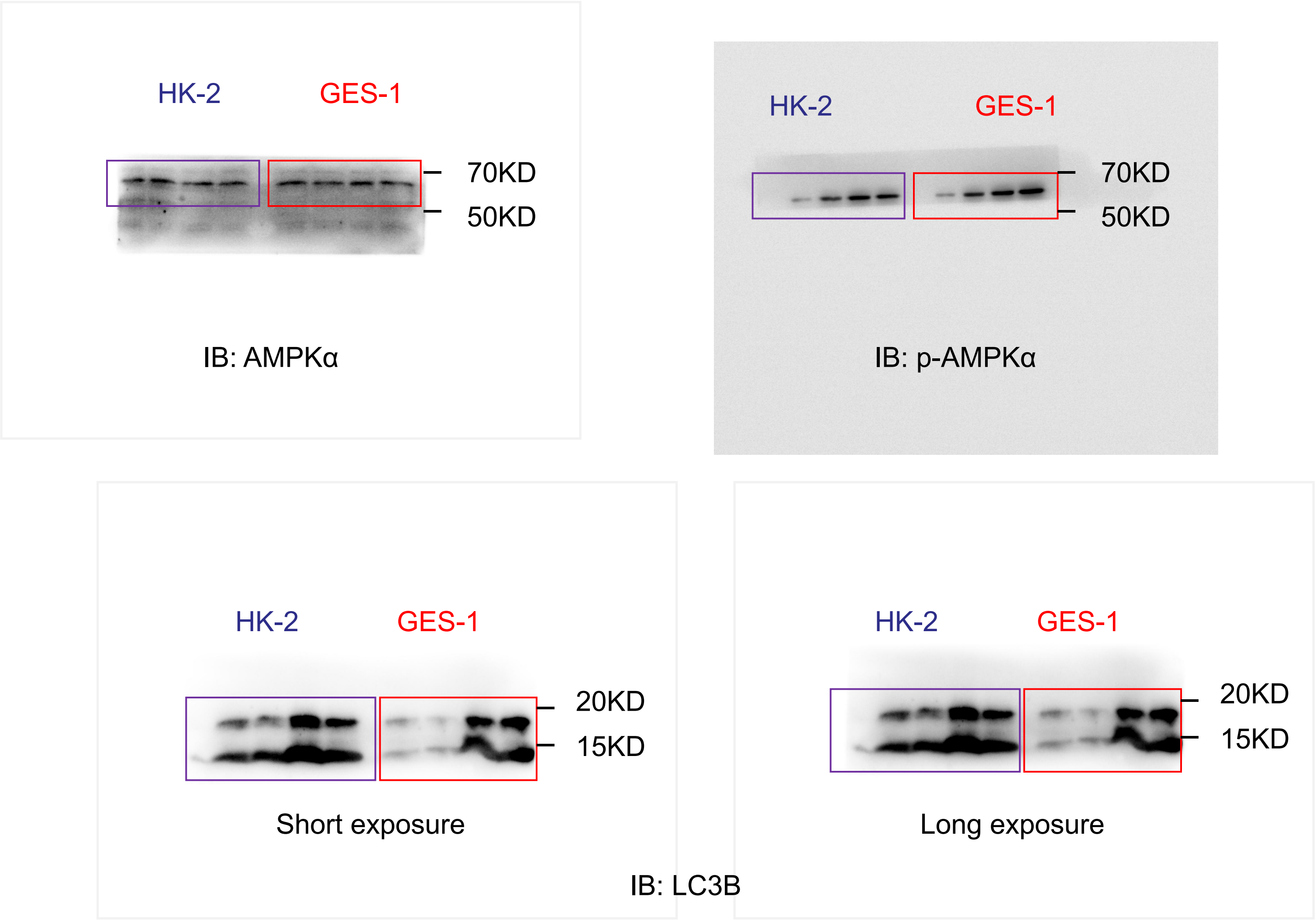
HK-2
GES-1
70KD
50KD
IB: AMPKα
HK-2
GES-1
70KD
50KD
IB: p-AMPKα
HK-2
GES-1
20KD
15KD
Short exposure
HK-2
GES-1
20KD
15KD
Long exposure
IB: LC3B

Figure 6A

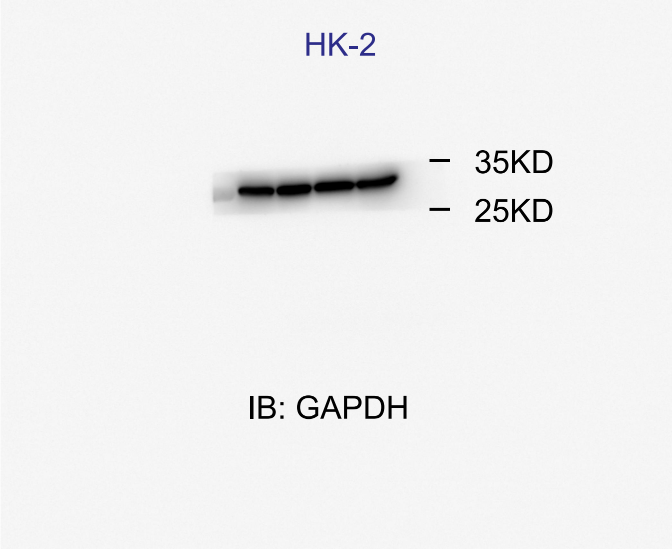

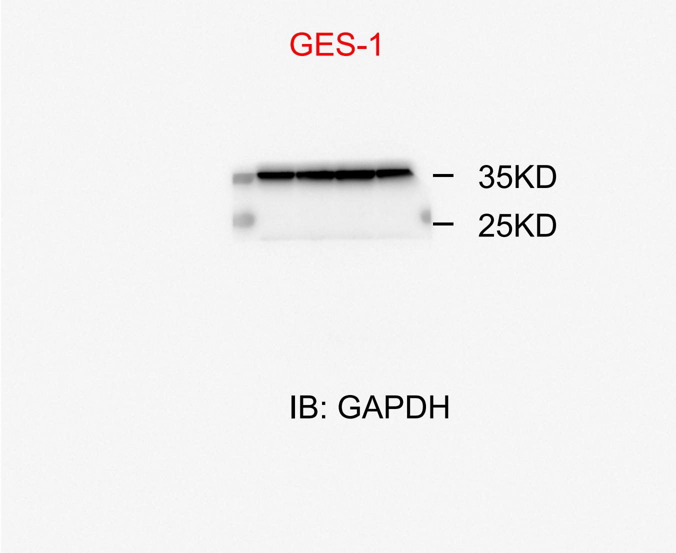

Figure 6B

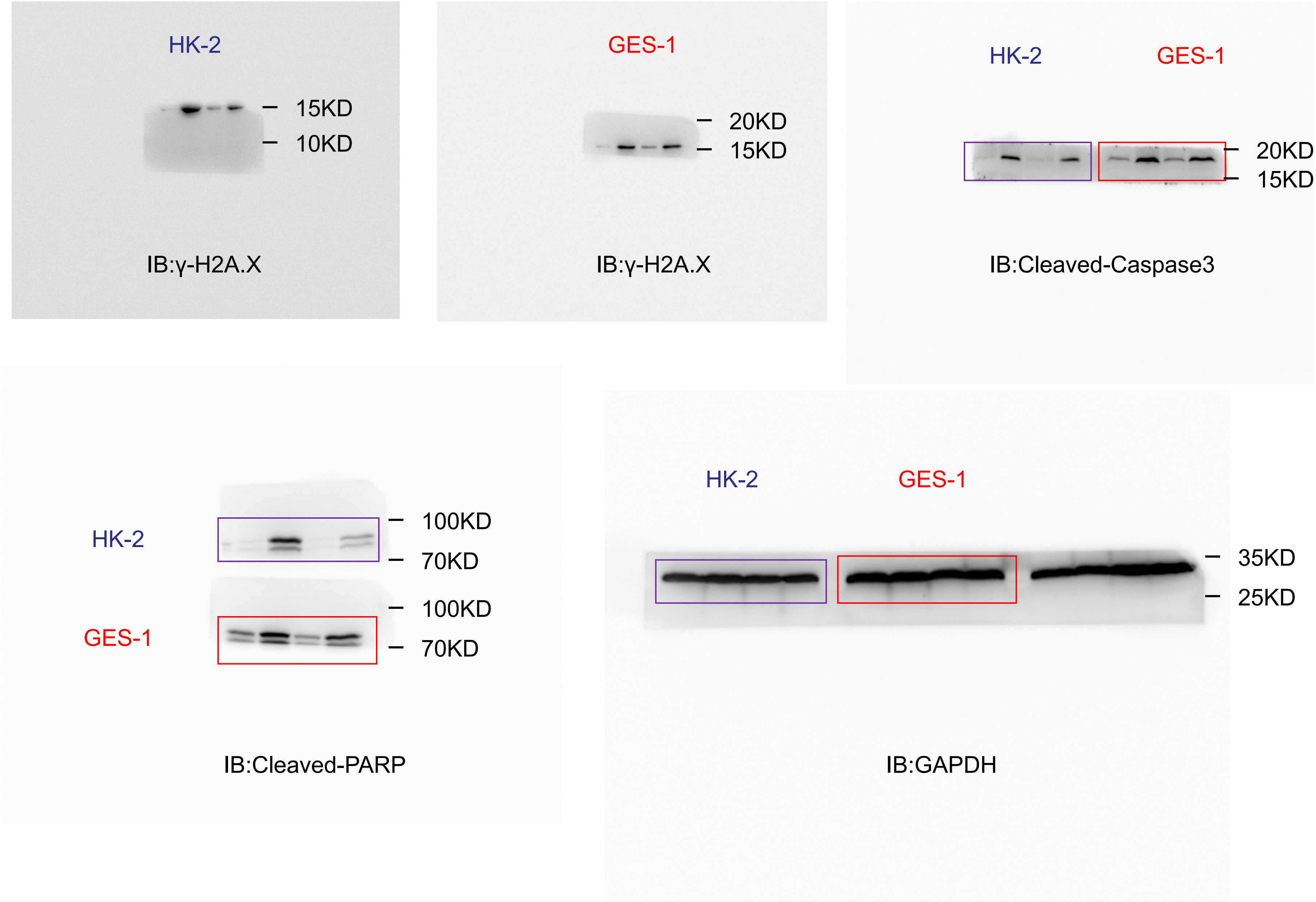

HK-2
15KD
10KD
IB:γ-H2A.X
GES-1
20KD
15KD
IB:γ-H2A.X
HK-2
GES-1
20KD
15KD
IB:Cleaved-Caspase3
HK-2
100KD
70KD
GES-1
100KD
70KD
IB:Cleaved-PARP
HK-2
GES-1
35KD
25KD
IB:GAPDH

Figure 6C

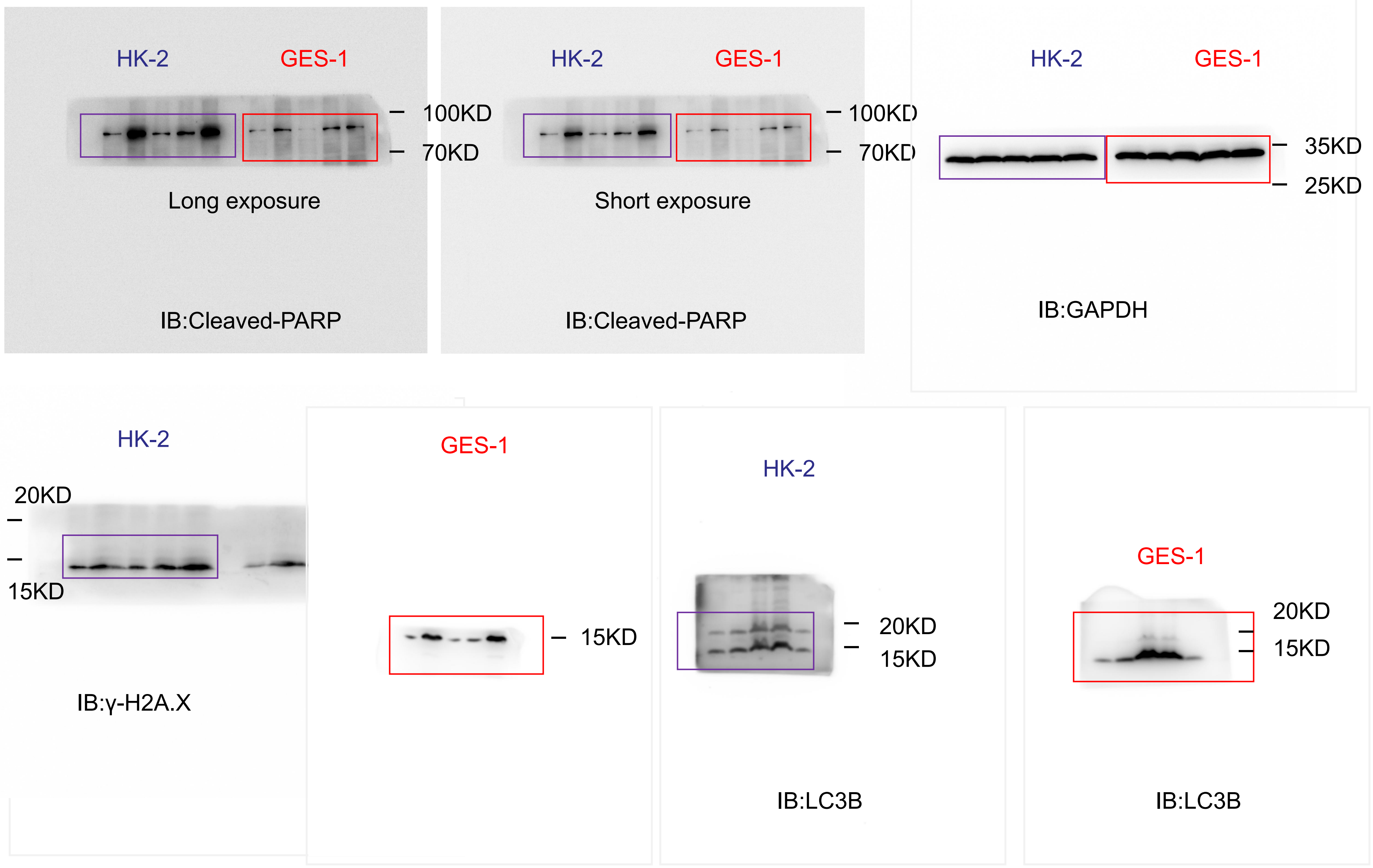
HK-2
GES-1
100KD
70KD
Long exposure
IB:Cleaved-PARP
HK-2
GES-1
100KD
70KD
Short exposure
IB:Cleaved-PARP
HK-2
GES-1
35KD
25KD
IB:GAPDH
HK-2
20KD
15KD
IB:γ-H2A.X
GES-1
15KD
HK-2
20KD
15KD
IB:LC3B
GES-1
20KD
15KD
IB:LC3B

Figure 6G

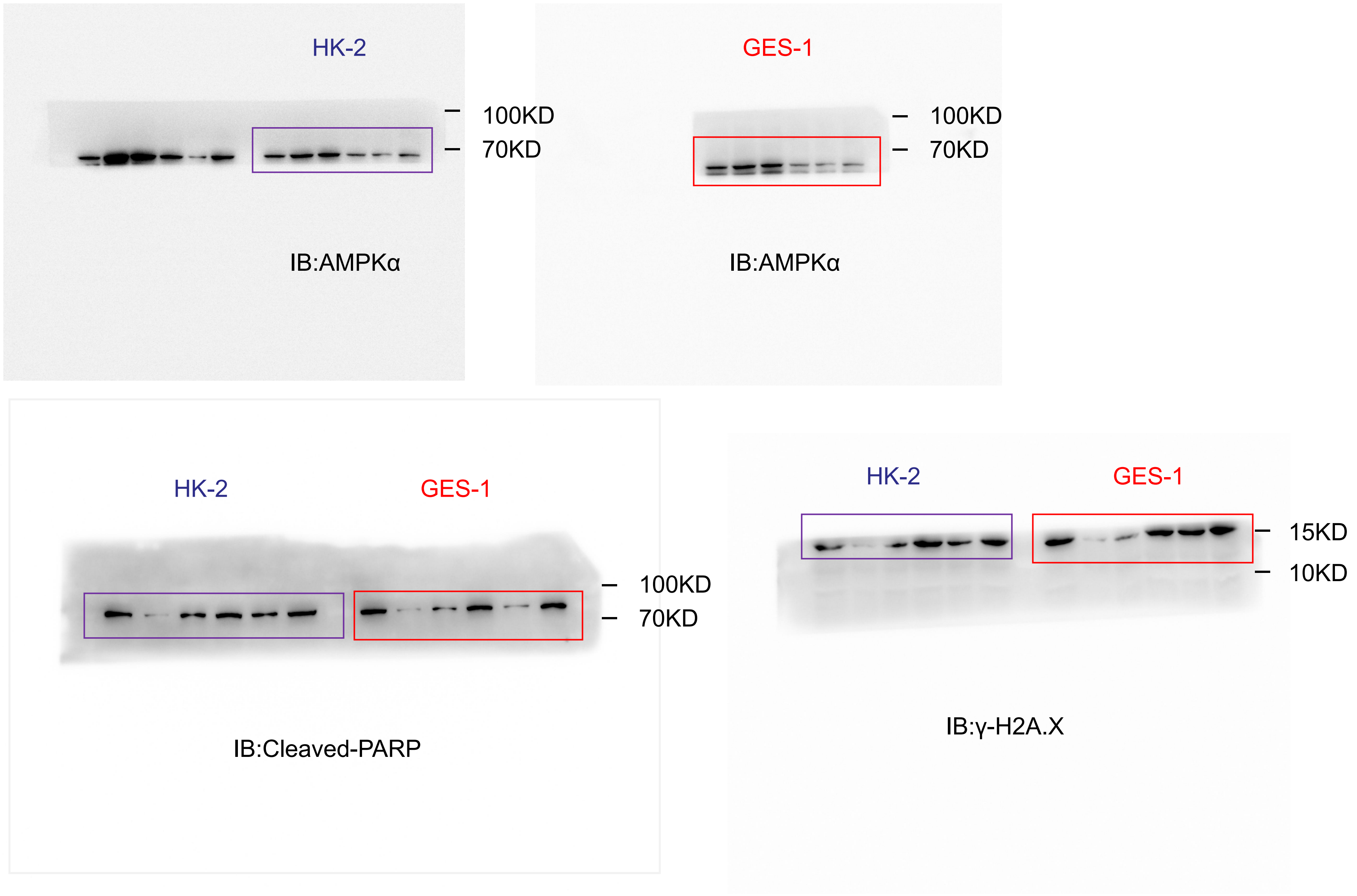
HK-2
100KD
70KD
IB:AMPKα
GES-1
100KD
70KD
IB:AMPKα
HK-2
GES-1
100KD
70KD
IB:Cleaved-PARP
HK-2
GES-1
15KD
10KD
IB:γ-H2A.X

Figure 6G

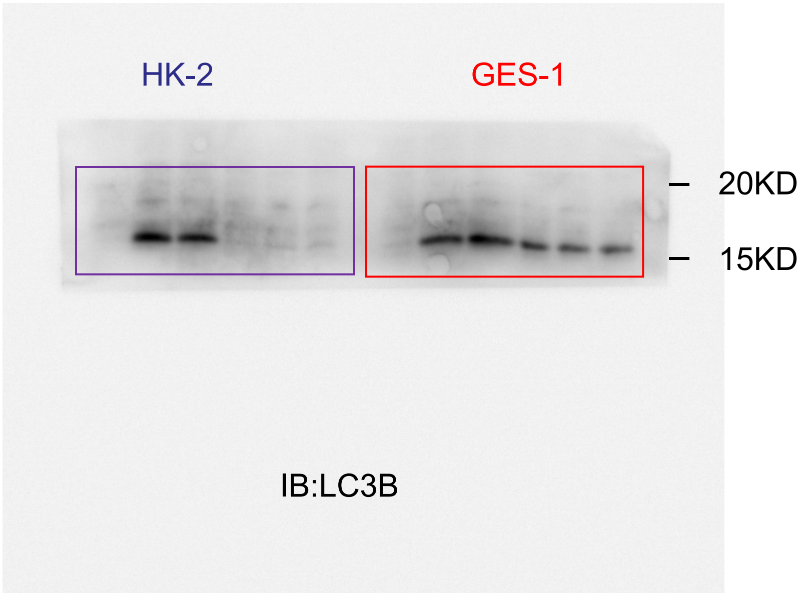

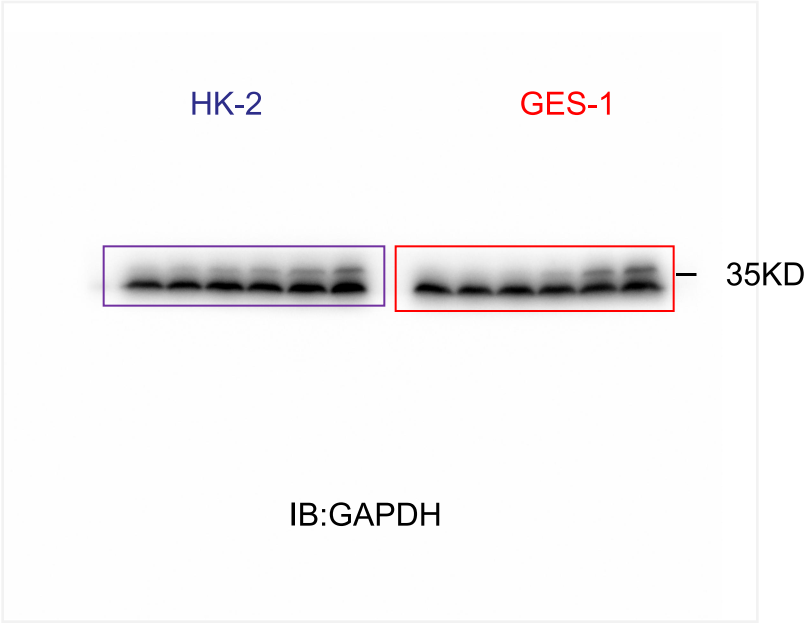

Figure S1J

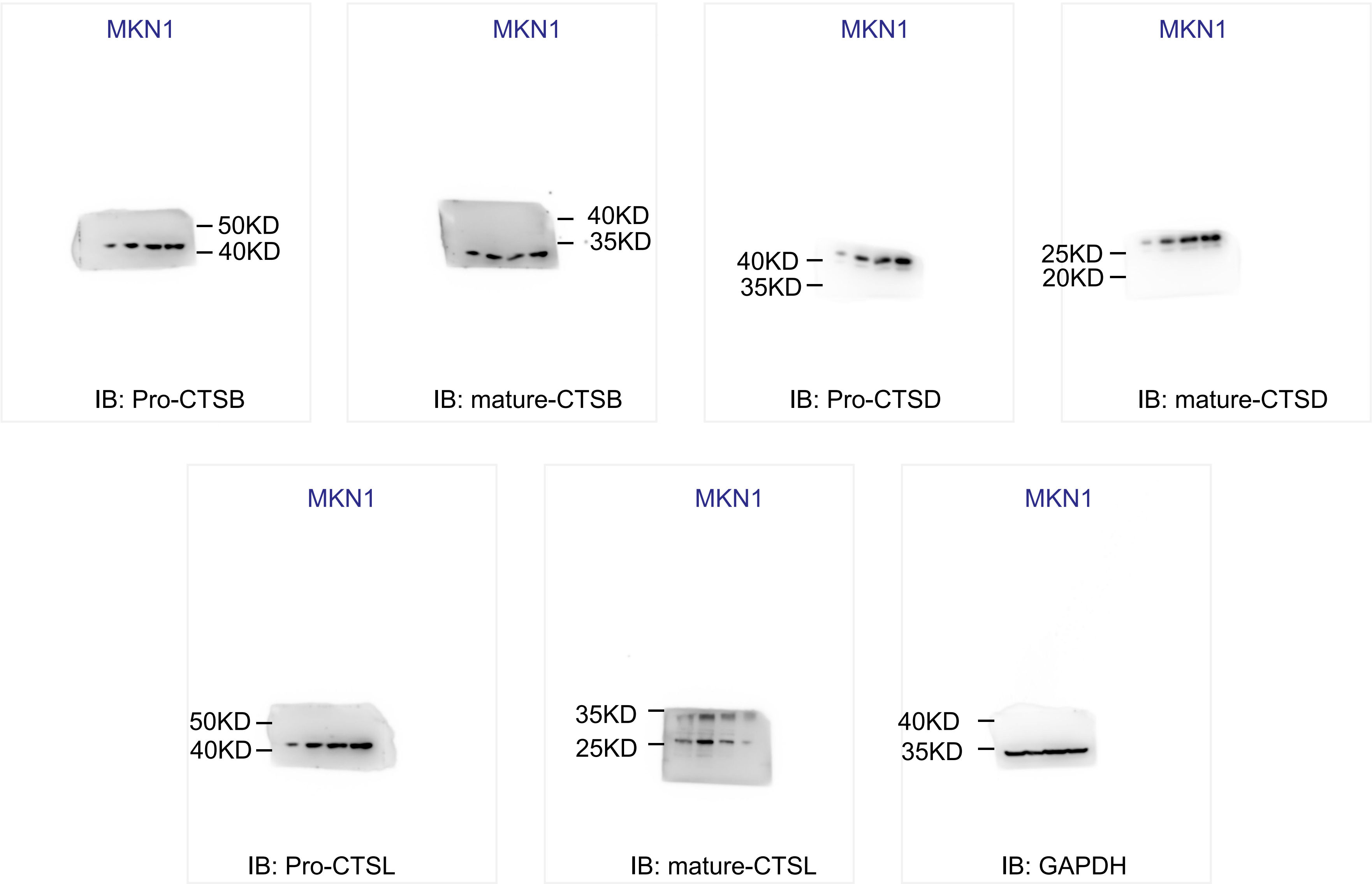
MKN1
50KD
40KD
IB: Pro-CTSB
MKN1
40KD
35KD
IB: mature-CTSB
MKN1
40KD
35KD
IB: Pro-CTSD
MKN1
25KD
20KD
IB: mature-CTSD
MKN1
50KD
40KD
IB: Pro-CTSL
MKN1
35KD
25KD
IB: mature-CTSL
MKN1
40KD
35KD
IB: GAPDH

Figure S1J

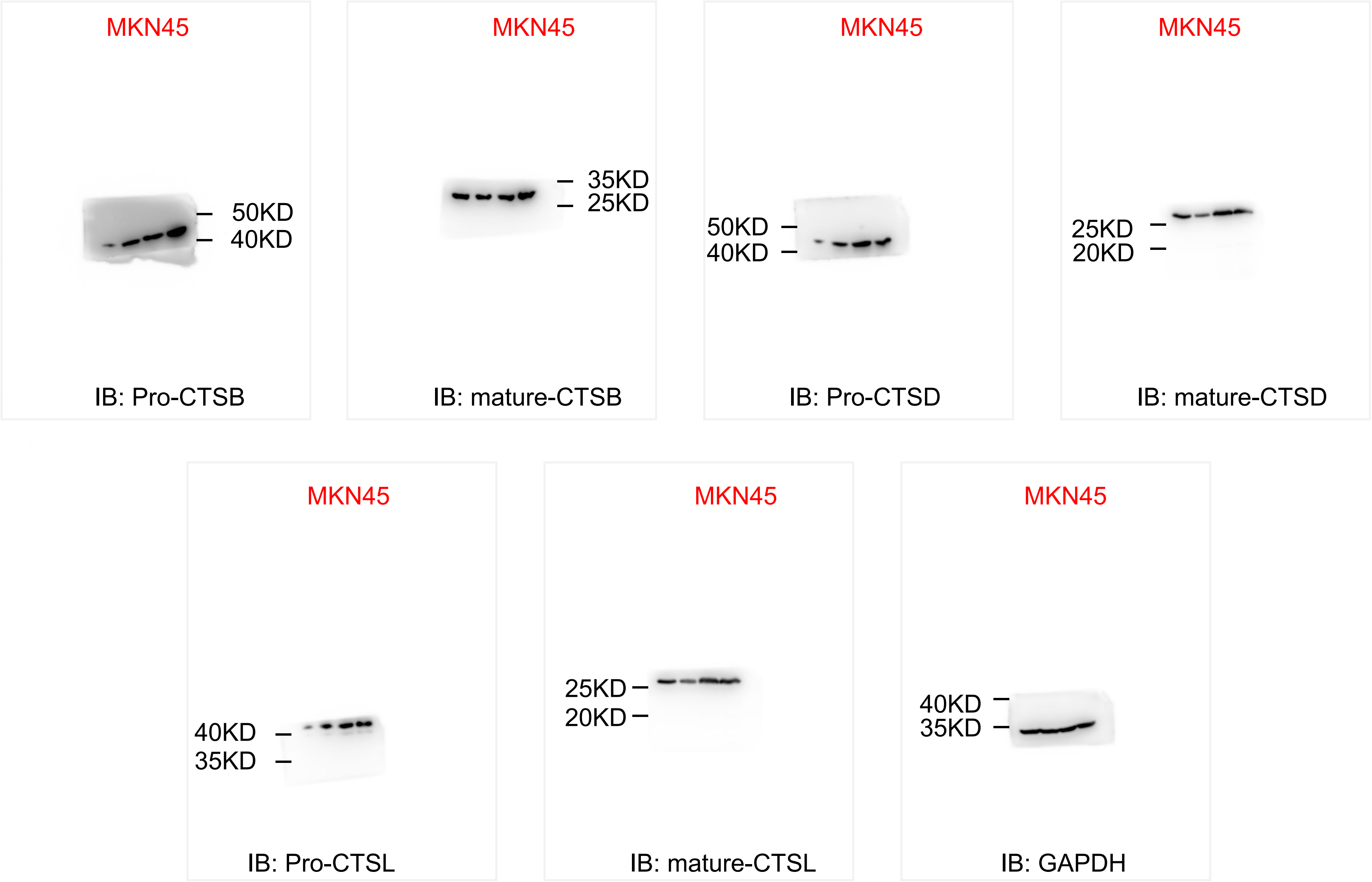
MKN45
50KD
40KD
IB: Pro-CTSB
MKN45
35KD
25KD
IB: mature-CTSB
MKN45
50KD
40KD
IB: Pro-CTSD
MKN45
25KD
20KD
IB: mature-CTSD
MKN45
40KD
35KD
IB: Pro-CTSL
MKN45
25KD
20KD
IB: mature-CTSL
MKN45
40KD
35KD
IB: GAPDH

Figure S2B

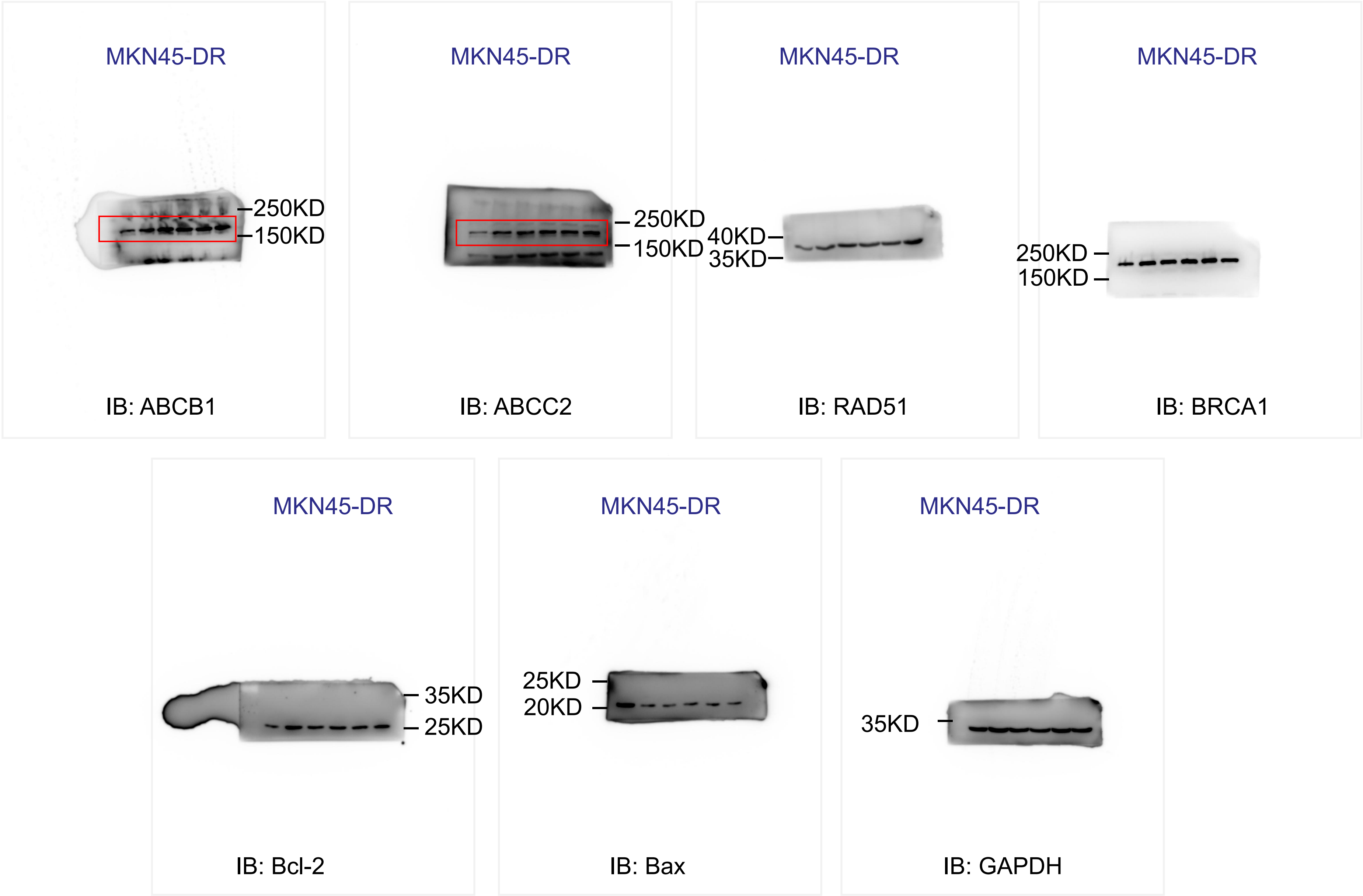
MKN45-DR
250KD
150KD
IB: ABCB1
MKN45-DR
250KD
150KD
IB: ABCC2
MKN45-DR
40KD
35KD
IB: RAD51
MKN45-DR
250KD
150KD
IB: BRCA1
MKN45-DR
35KD
25KD
IB: Bcl-2
MKN45-DR
25KD
20KD
IB: Bax
MKN45-DR
35KD
IB: GAPDH

Figure S5B

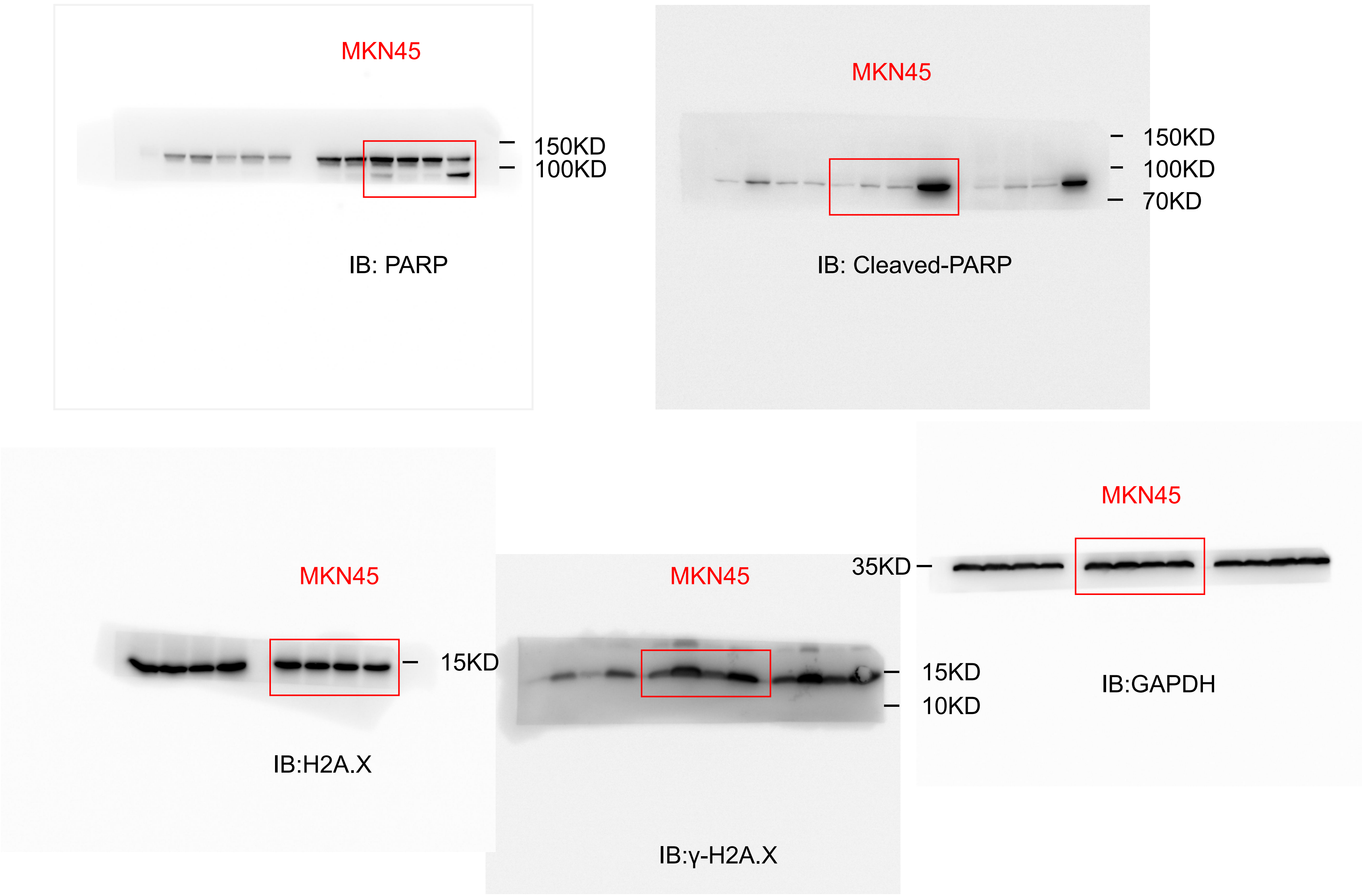
MKN45
150KD
100KD
IB: PARP
MKN45
150KD
100KD
70KD
IB: Cleaved-PARP
MKN45
15KD
IB:H2A.X
MKN45
15KD
10KD
IB:γ-H2A.X
MKN45
35KD
IB:GAPDH

Figure S6F

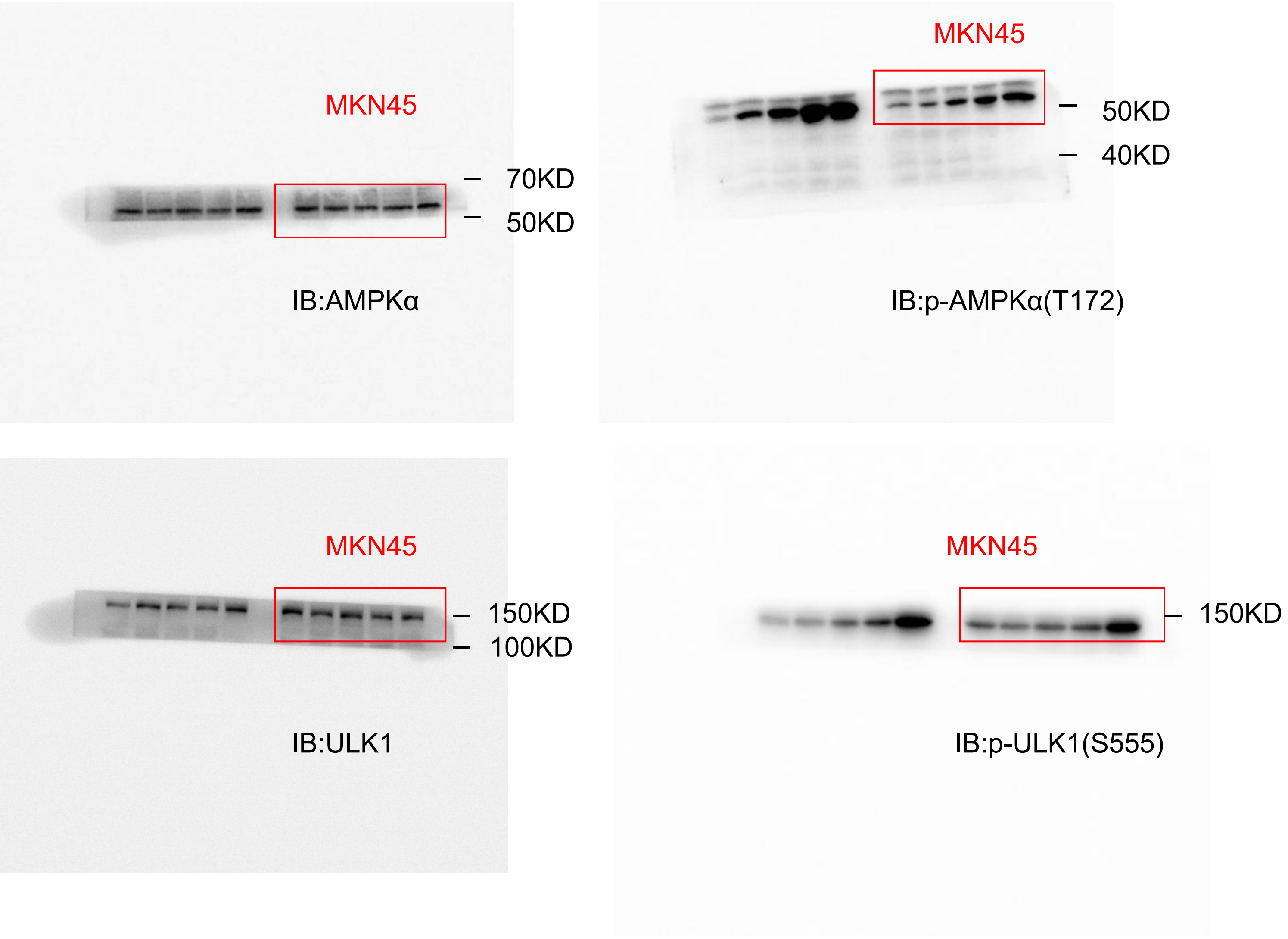
MKN45
70KD
50KD
IB:AMPKα
MKN45
50KD
40KD
IB:p-AMPKα(T172)
MKN45
150KD
100KD
IB:ULK1
MKN45
150KD
IB:p-ULK1(S555)

Figure S6F

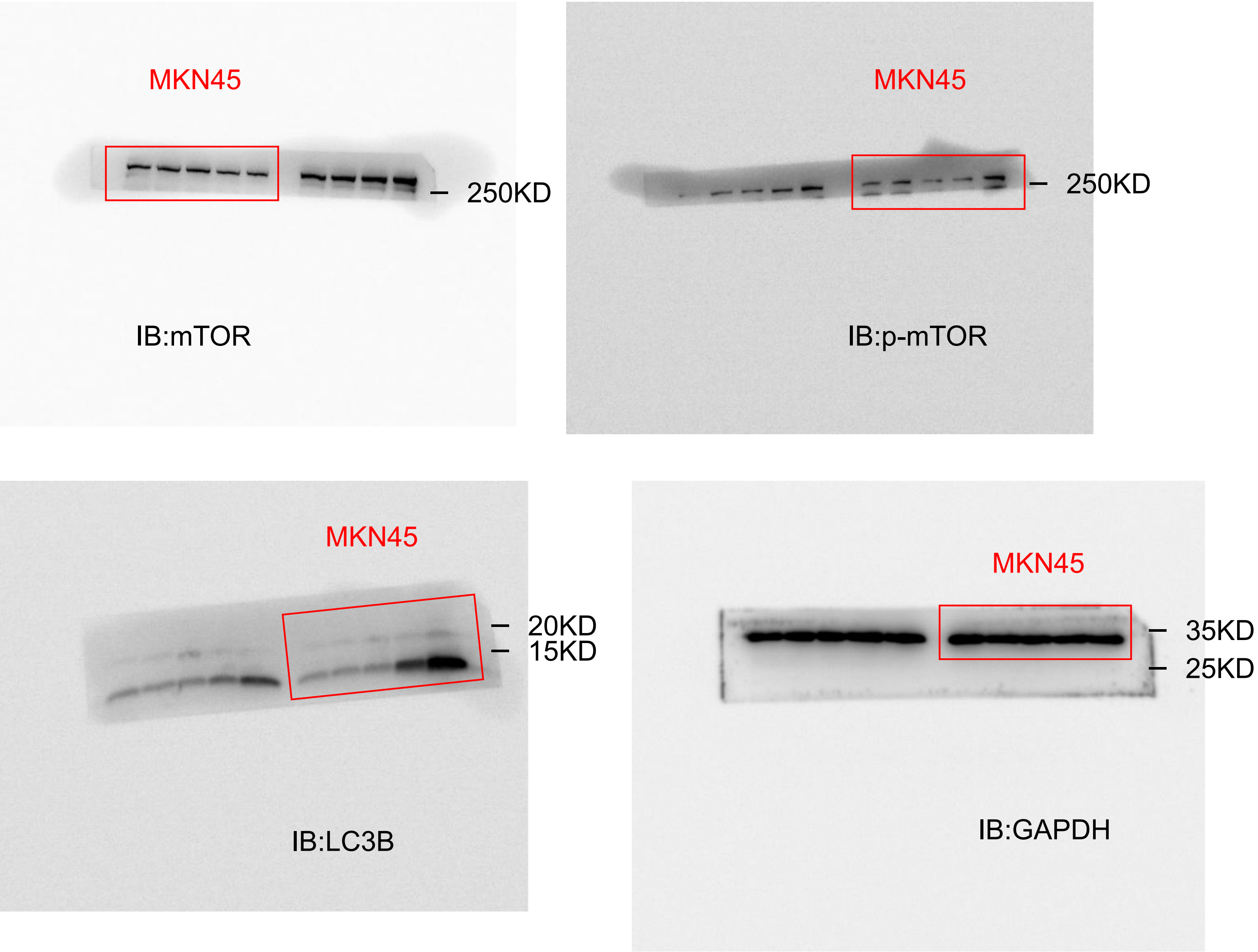
MKN45
250KD
IB:mTOR
MKN45
250KD
IB:p-mTOR
MKN45
20KD
15KD
IB:LC3B
MKN45
35KD
25KD
IB:GAPDH

Figure S6G

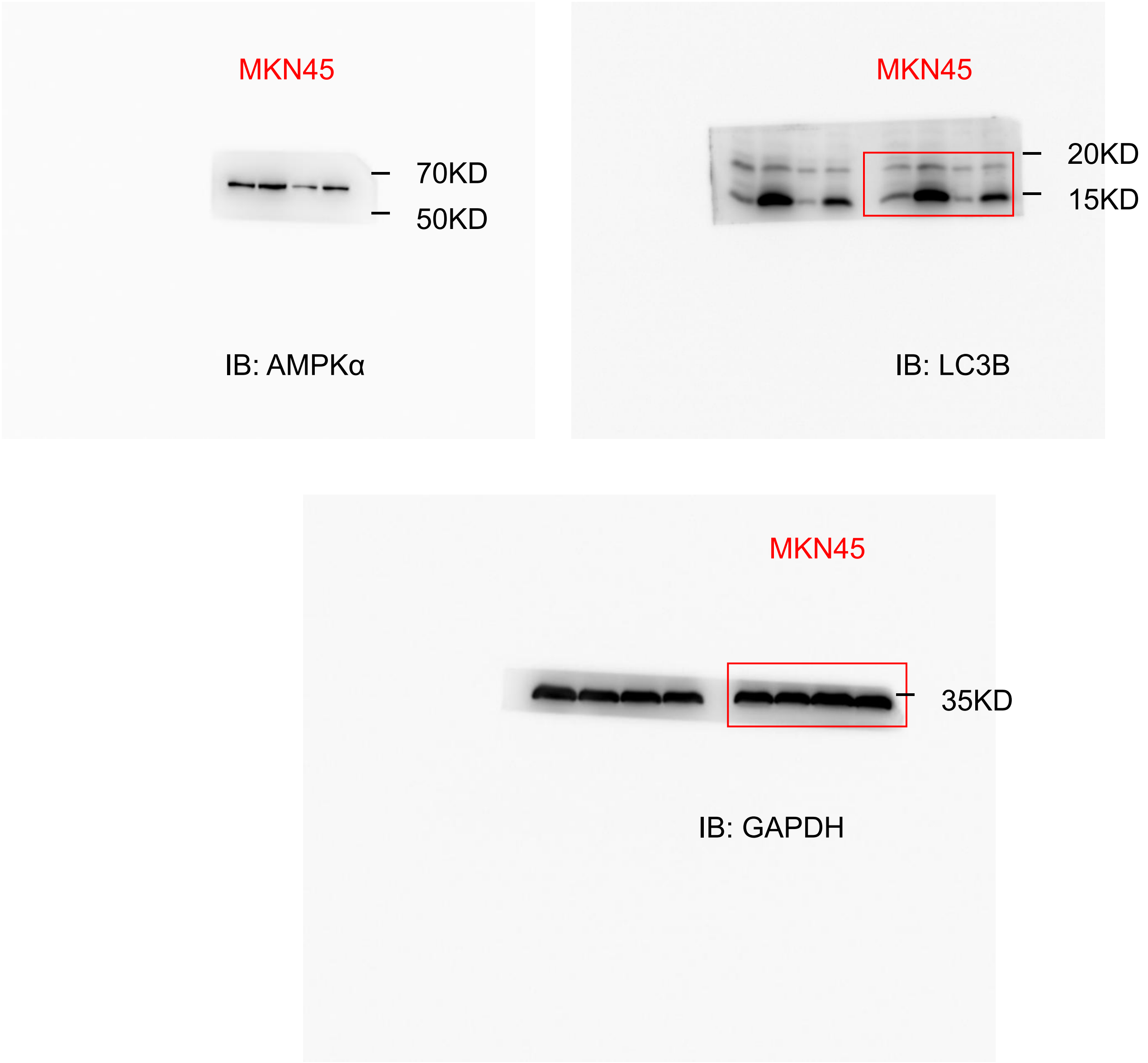
MKN45
70KD
50KD
IB: AMPKα
MKN45
20KD
15KD
IB: LC3B
MKN45
35KD
IB: GAPDH

Figure S6H

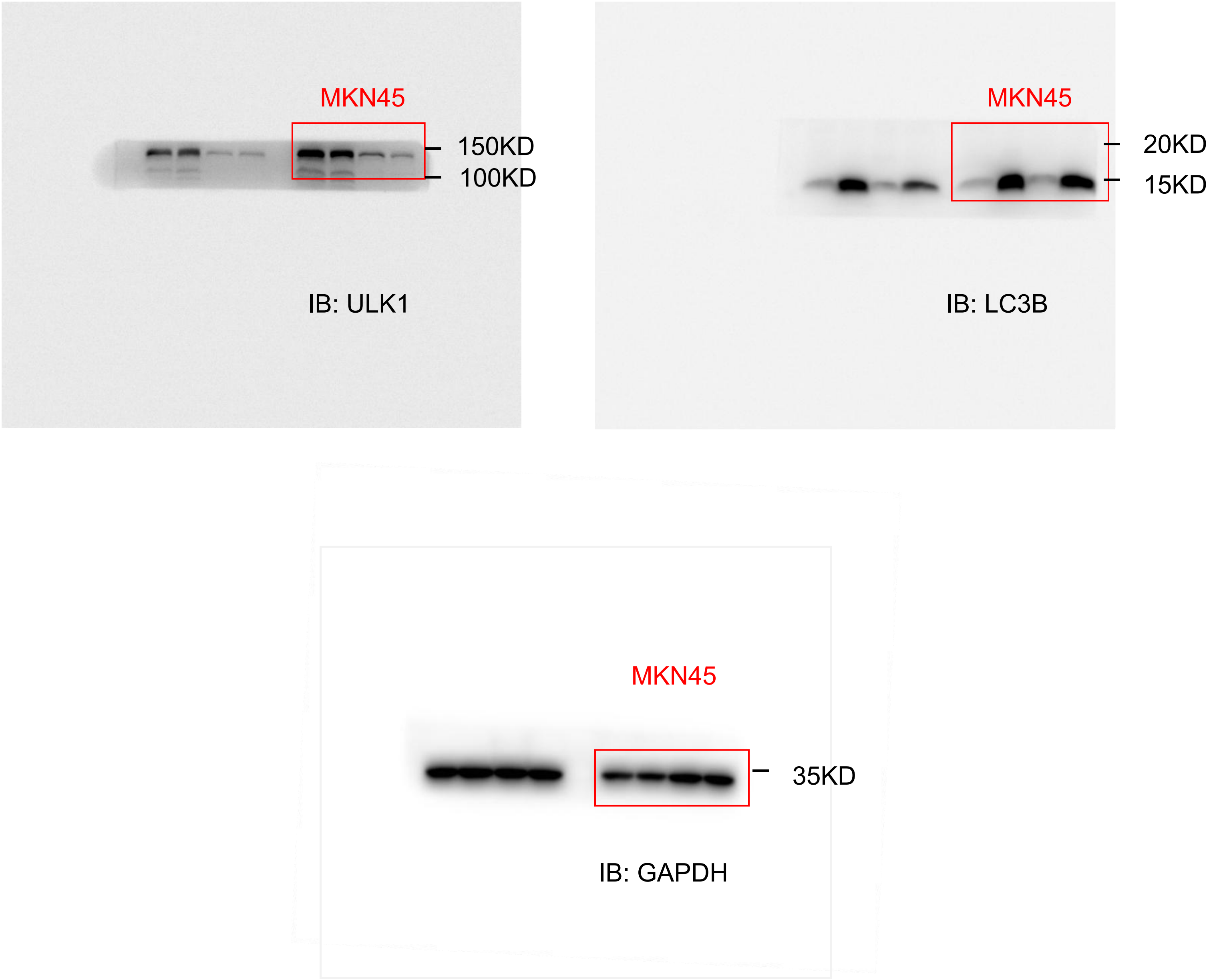
MKN45
150KD
100KD
IB: ULK1
MKN45
20KD
15KD
IB: LC3B
MKN45
35KD
IB: GAPDH

Figure S6I

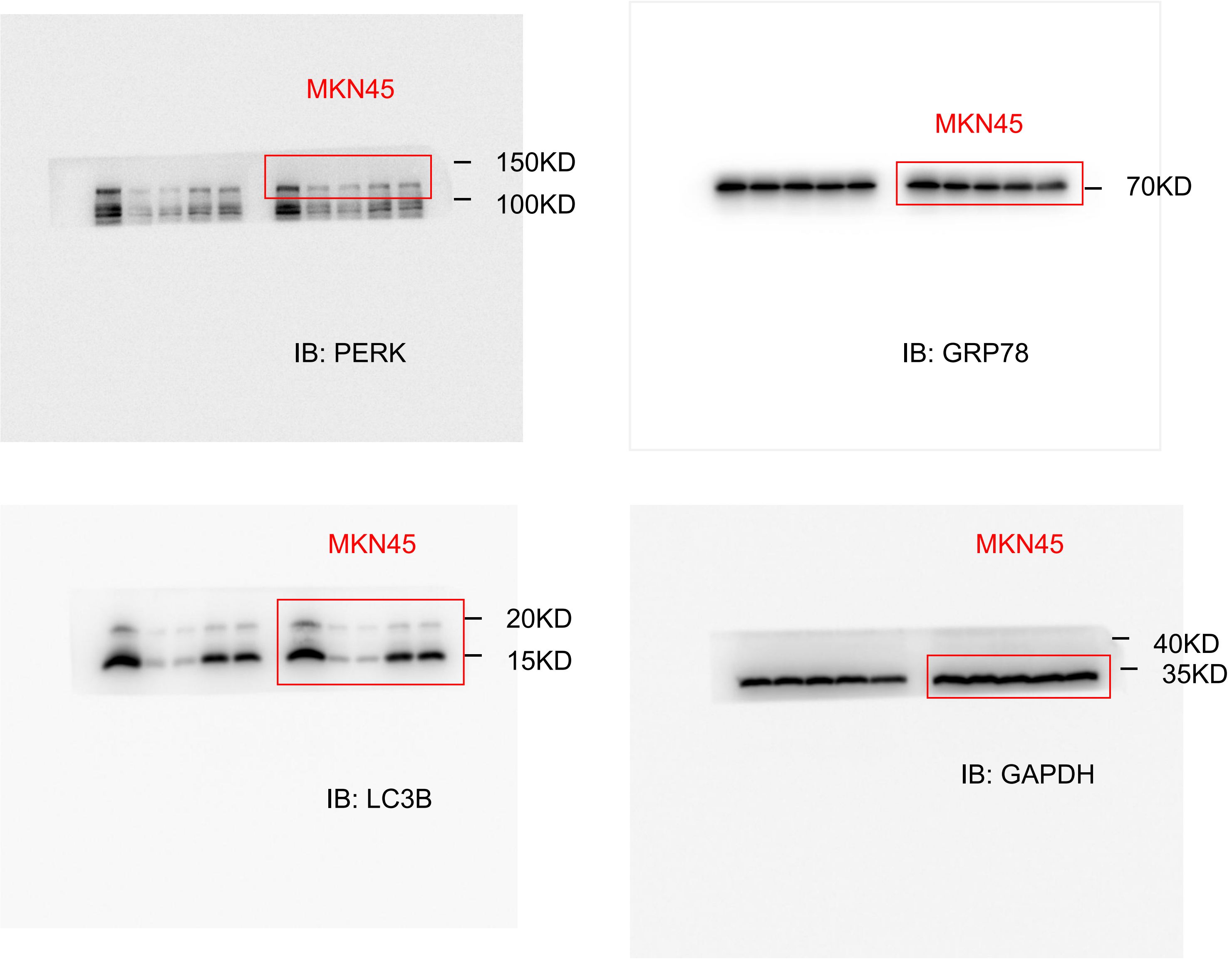
MKN45
150KD
100KD
IB: PERK
MKN45
70KD
IB: GRP78
MKN45
20KD
15KD
IB: LC3B
MKN45
40KD
35KD
IB: GAPDH

Figure S6J

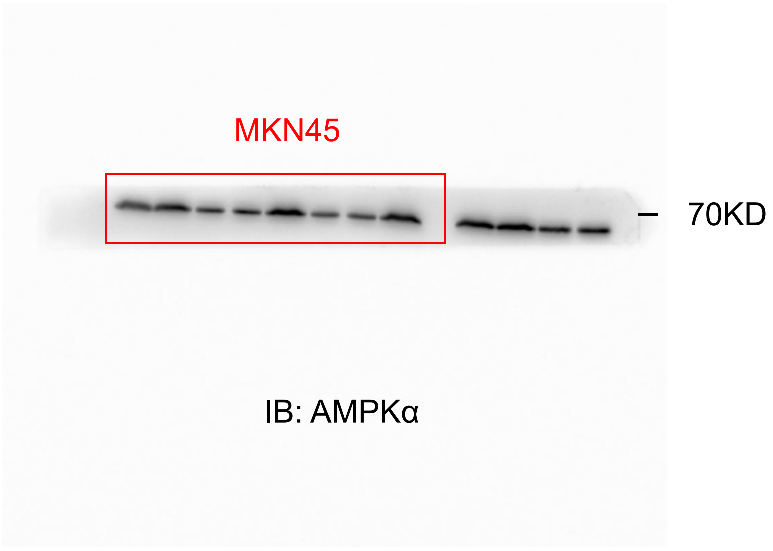
MKN45
70KD
IB: AMPKα

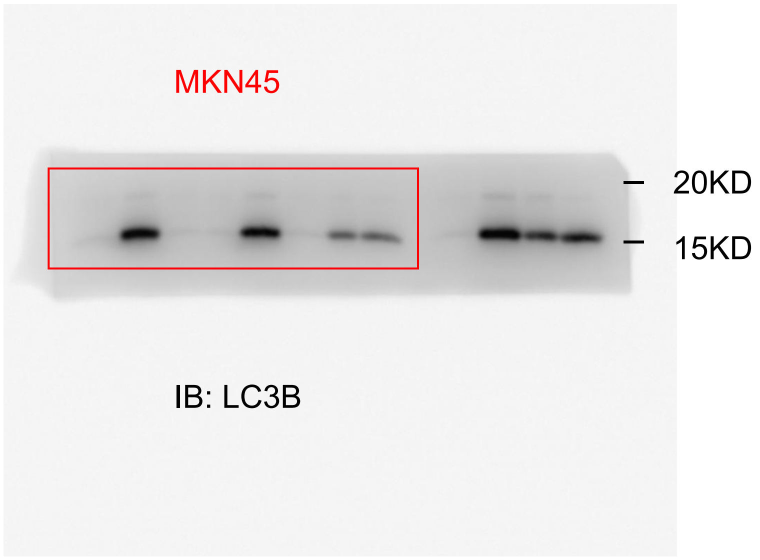
MKN45
20KD
15KD
IB: LC3B

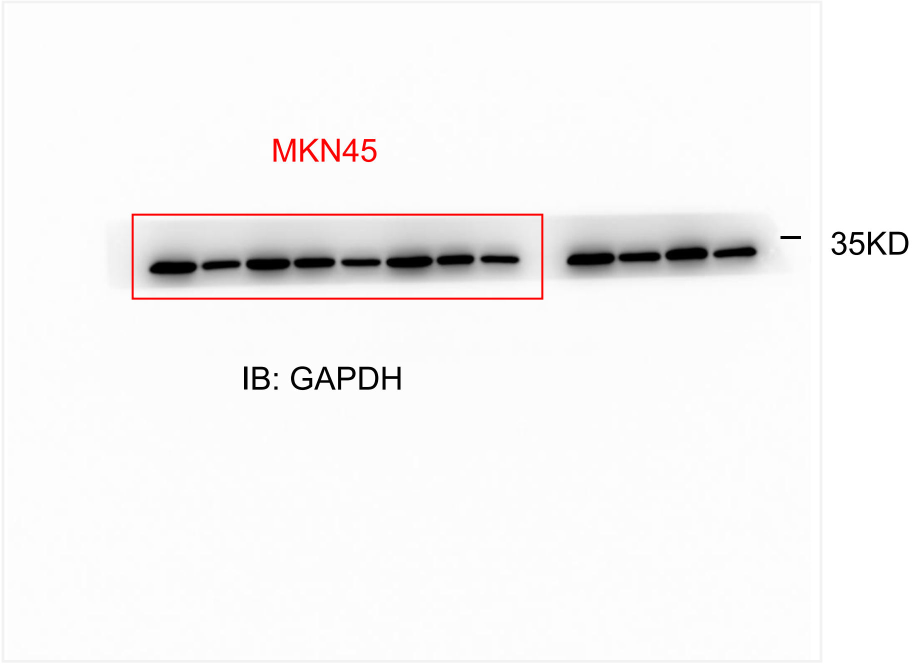
MKN45
35KD
IB: GAPDH

Figure S6K

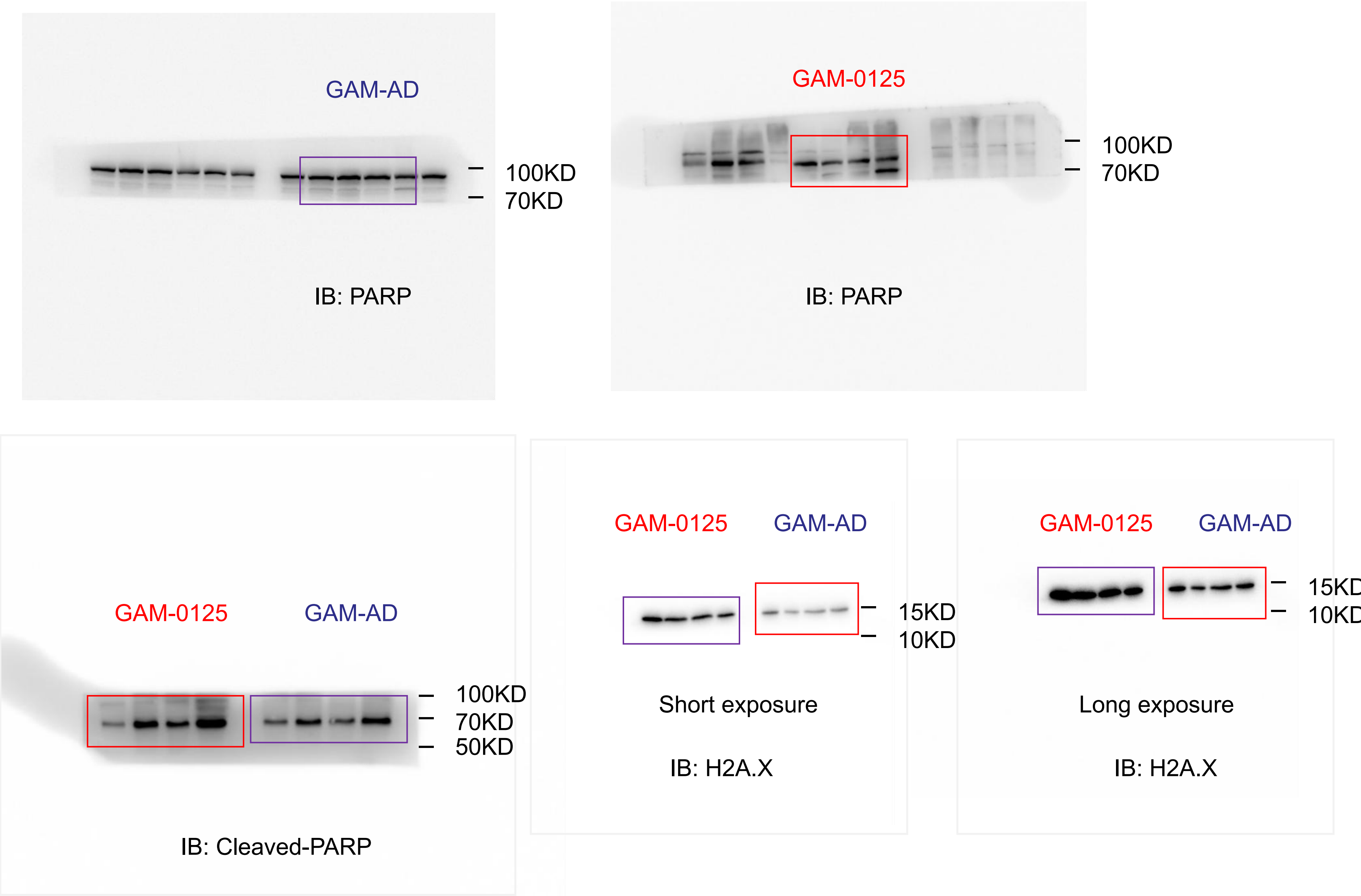
GAM-AD
100KD
70KD
IB: PARP
GAM-0125
100KD
70KD
IB: PARP
GAM-0125
GAM-AD
100KD
70KD
50KD
IB: Cleaved-PARP
GAM-0125
GAM-AD
15KD
10KD
Short exposure
IB: H2A.X
GAM-0125
GAM-AD
Long exposure
IB: H2A.X

Figure S6K

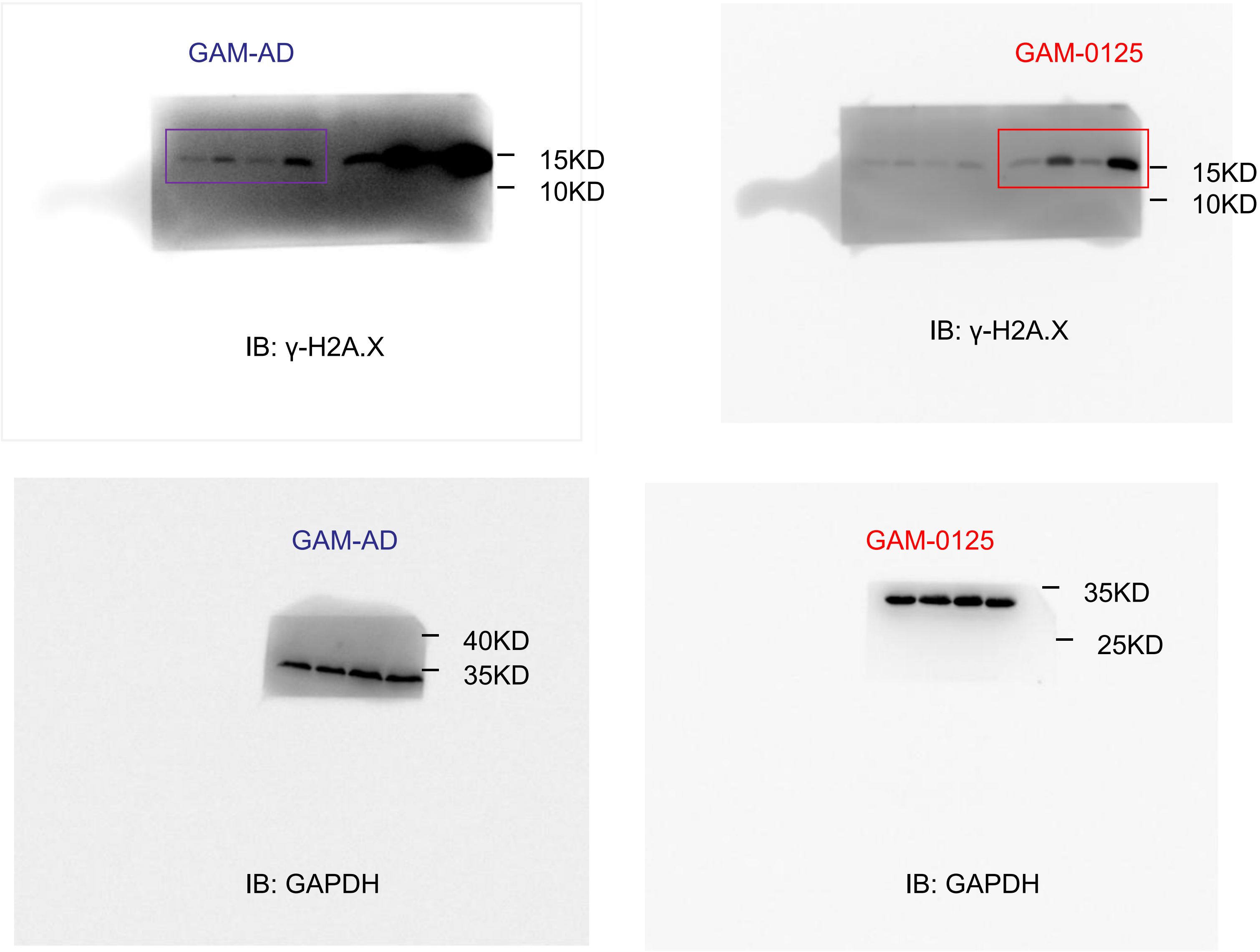

GAM-AD
15KD
10KD
IB: γ-H2A.X
GAM-0125
15KD
10KD
IB: γ-H2A.X
GAM-AD
40KD
35KD
IB: GAPDH
GAM-0125
35KD
25KD
IB: GAPDH

Figure S7E

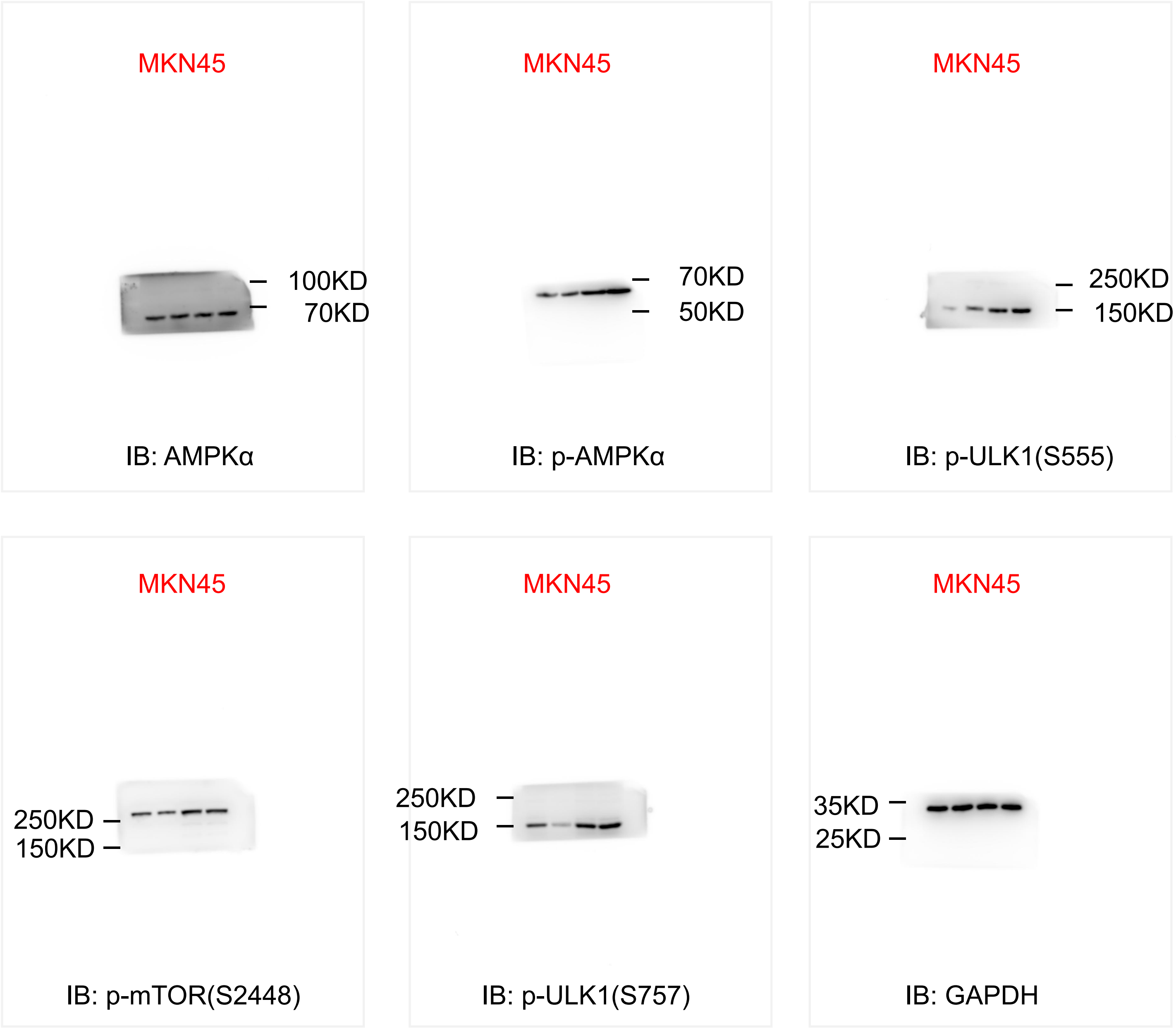
MKN45
100KD
70KD
IB: AMPKα
MKN45
70KD
50KD
IB: p-AMPKα
MKN45
250KD
150KD
IB: p-ULK1(S555)
MKN45
250KD
150KD
IB: p-mTOR(S2448)
MKN45
250KD
150KD
IB: p-ULK1(S757)
MKN45
35KD
25KD
IB: GAPDH

Figure S7F

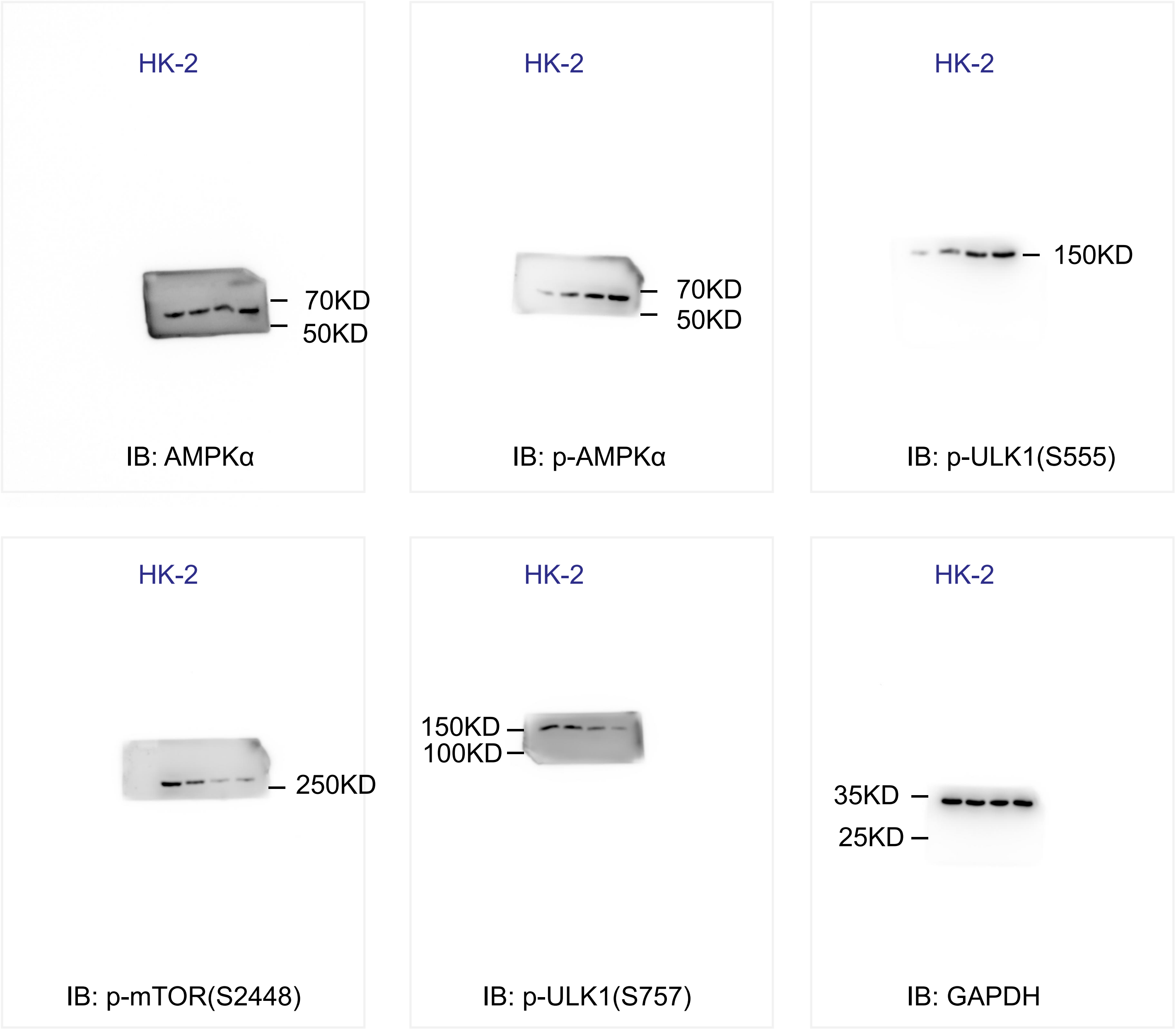
HK-2
70KD
50KD
IB: AMPKα
HK-2
70KD
50KD
IB: p-AMPKα
HK-2
150KD
IB: p-ULK1(S555)
HK-2
250KD
IB: p-mTOR(S2448)
HK-2
150KD
100KD
IB: p-ULK1(S757)
HK-2
35KD
25KD
IB: GAPDH

Figure S7F

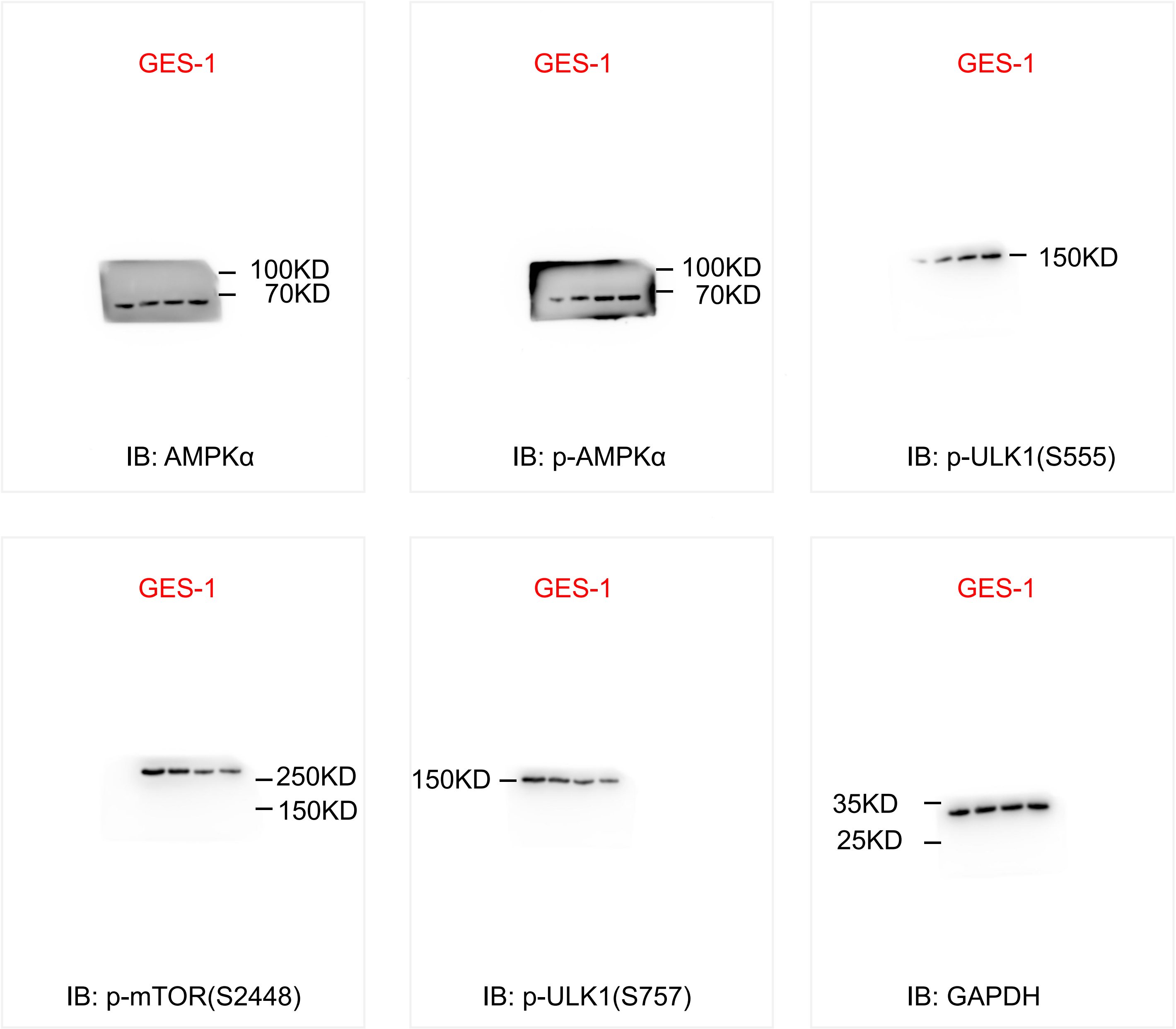
GES-1
100KD
70KD
IB: AMPKα
GES-1
100KD
70KD
IB: p-AMPKα
GES-1
150KD
IB: p-ULK1(S555)
GES-1
250KD
150KD
IB: p-mTOR(S2448)
GES-1
150KD
IB: p-ULK1(S757)
GES-1
35KD
25KD
IB: GAPDH

Figure 5C

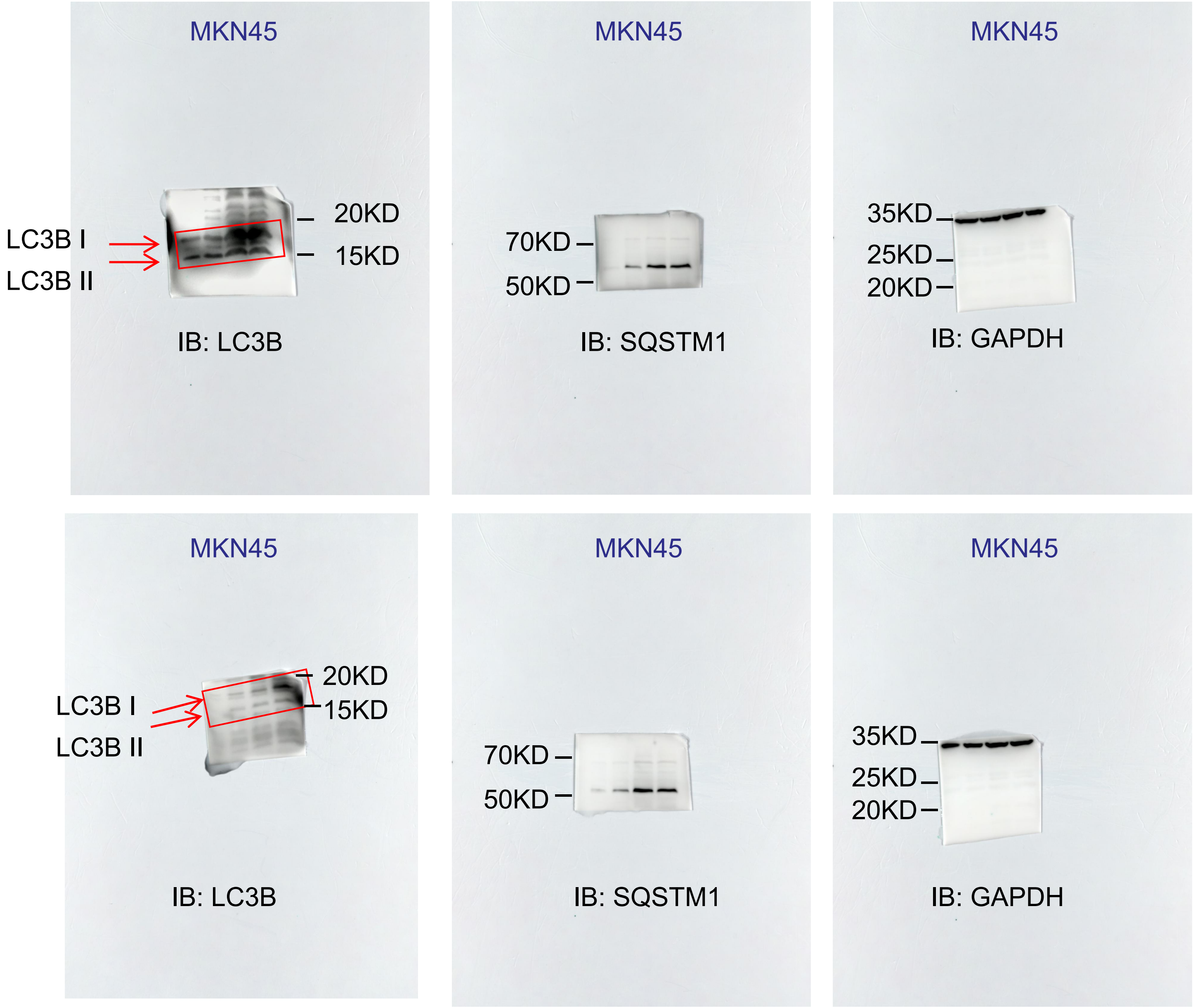
MKN45
LC3B I
LC3B II
20KD
15KD
IB: LC3B
MKN45
70KD
50KD
IB: SQSTM1
MKN45
35KD
25KD
20KD
IB: GAPDH
MKN45
LC3B I
LC3B II
20KD
15KD
IB: LC3B
MKN45
70KD
50KD
IB: SQSTM1
MKN45
35KD
25KD
20KD
IB: GAPDH

Figure 5J

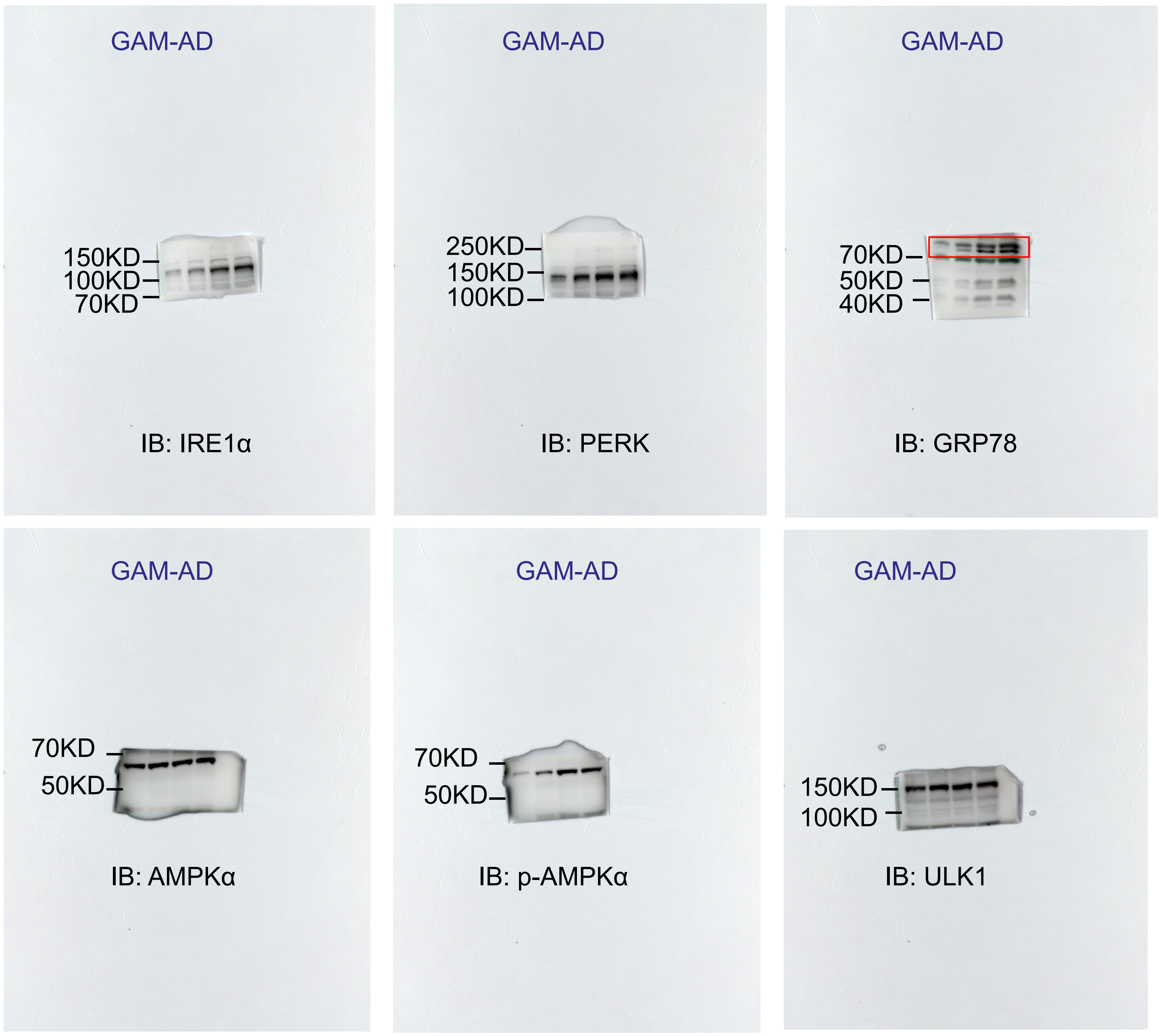
GAM-AD
150KD
100KD
70KD
IB: IRE1α
GAM-AD
250KD
150KD
100KD
IB: PERK
GAM-AD
70KD
50KD
40KD
IB: GRP78
GAM-AD
70KD
50KD
IB: AMPKα
GAM-AD
70KD
50KD
IB: p-AMPKα
GAM-AD
150KD
100KD
IB: ULK1

Figure 5J

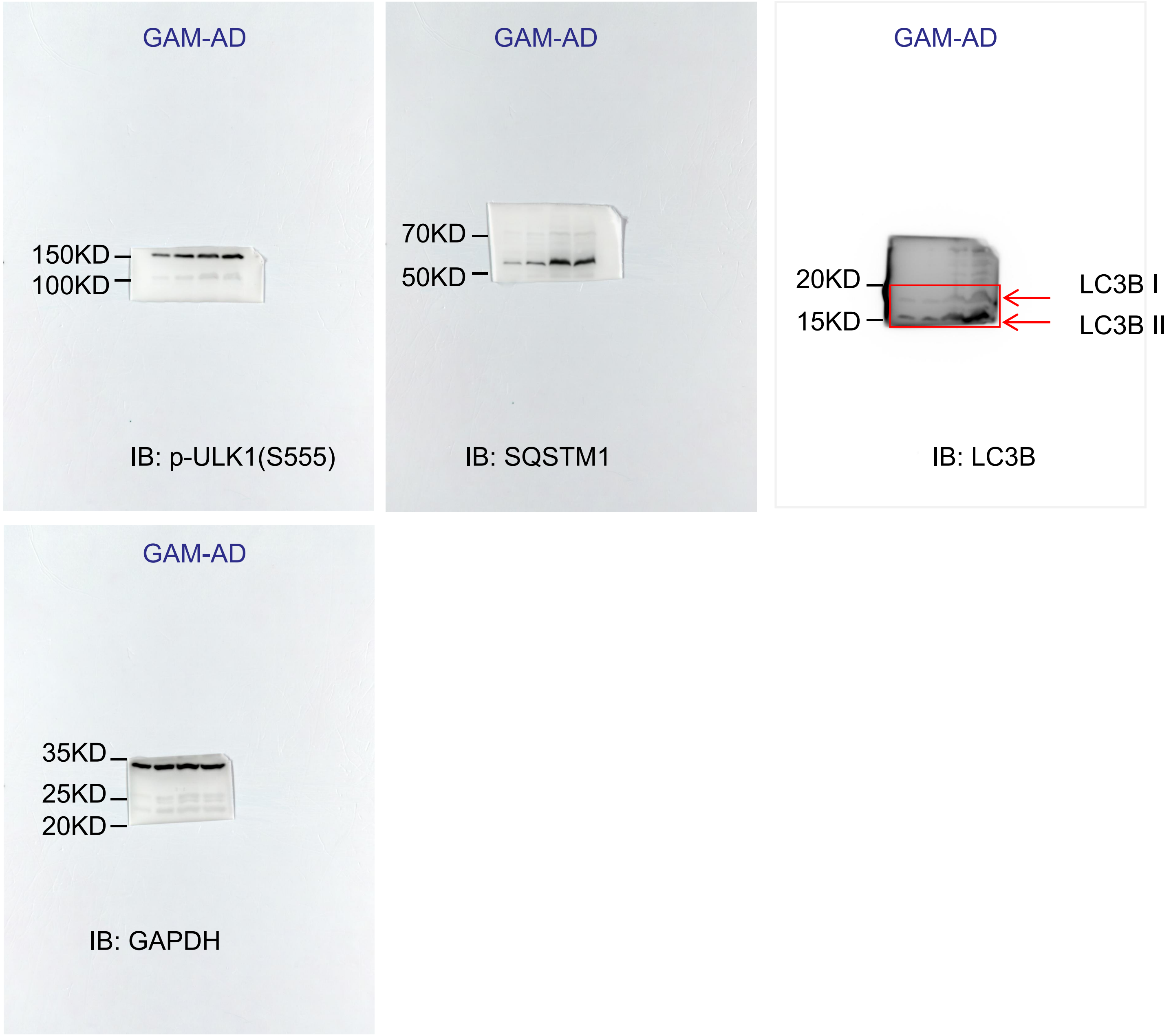
GAM-AD
150KD
100KD
IB: p-ULK1(S555)
GAM-AD
70KD
50KD
IB: SQSTM1
GAM-AD
20KD
15KD
LC3B I
LC3B II
IB: LC3B
GAM-AD
35KD
25KD
20KD
IB: GAPDH

Figure 5J

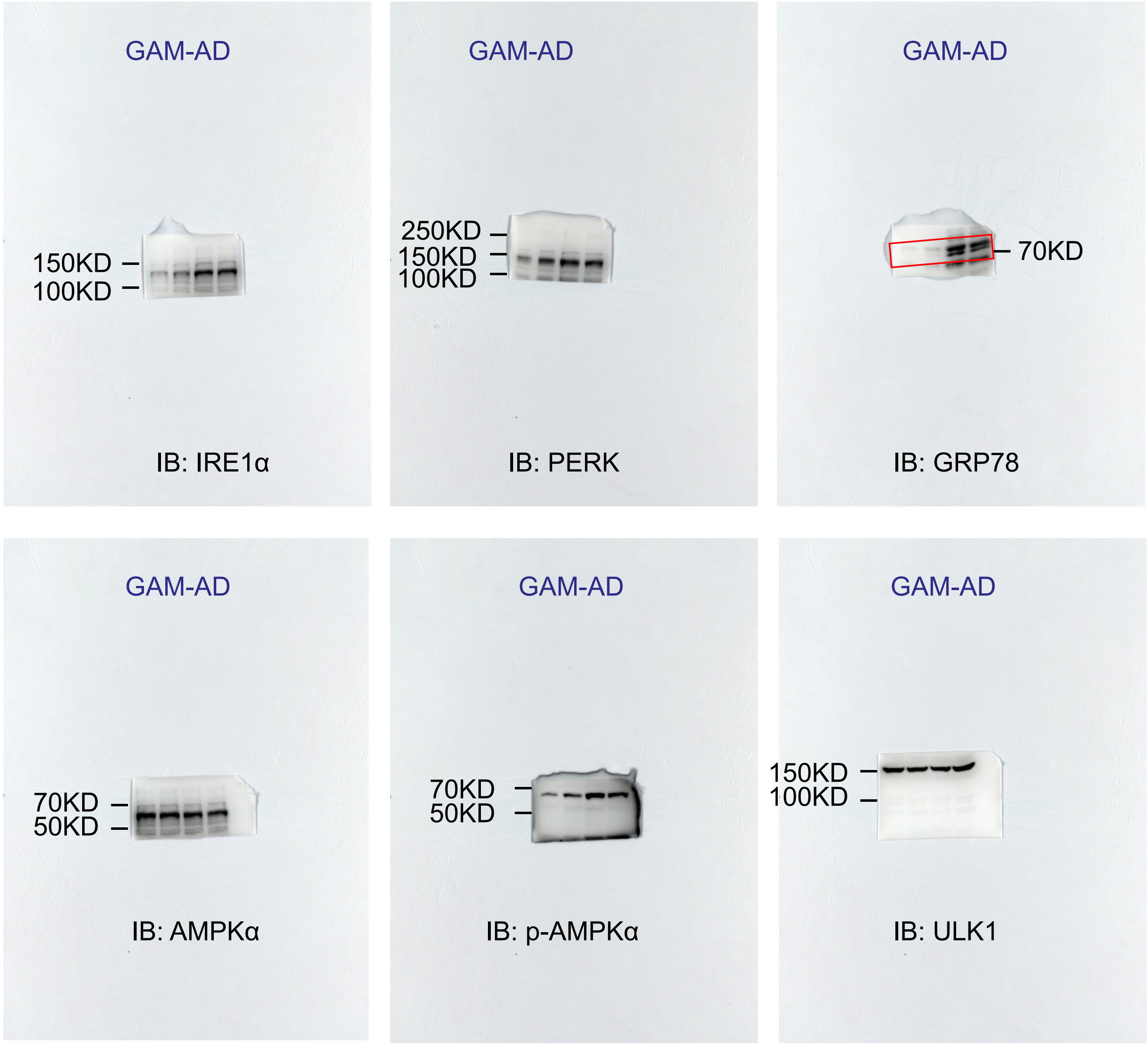
GAM-AD
150KD
100KD
IB: IRE1α
GAM-AD
250KD
150KD
100KD
IB: PERK
GAM-AD
70KD
IB: GRP78
GAM-AD
70KD
50KD
IB: AMPKα
GAM-AD
70KD
50KD
IB: p-AMPKα
GAM-AD
150KD
100KD
IB: ULK1

Figure 5J

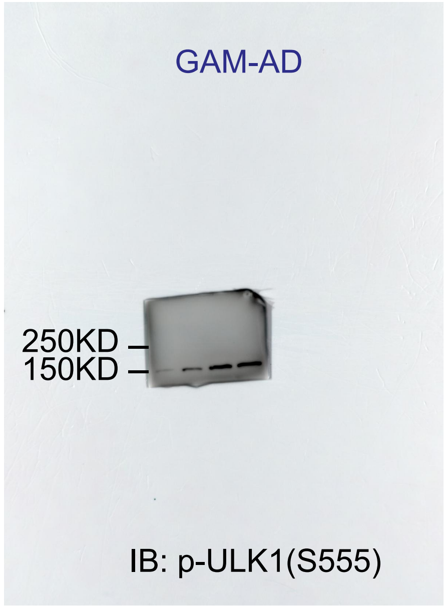

GAM-AD
250KD
150KD
IB: p-ULK1(S555)

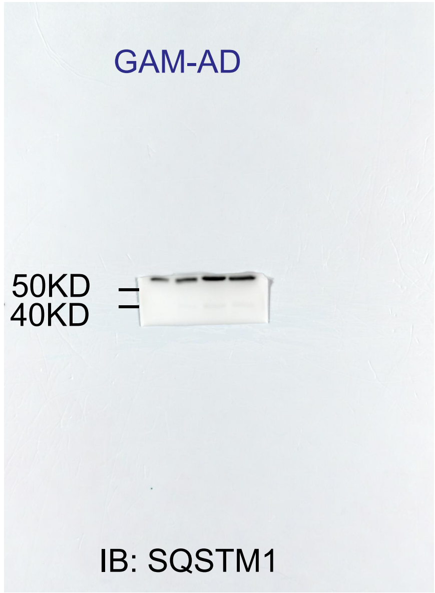

GAM-AD
50KD
40KD
IB: SQSTM1

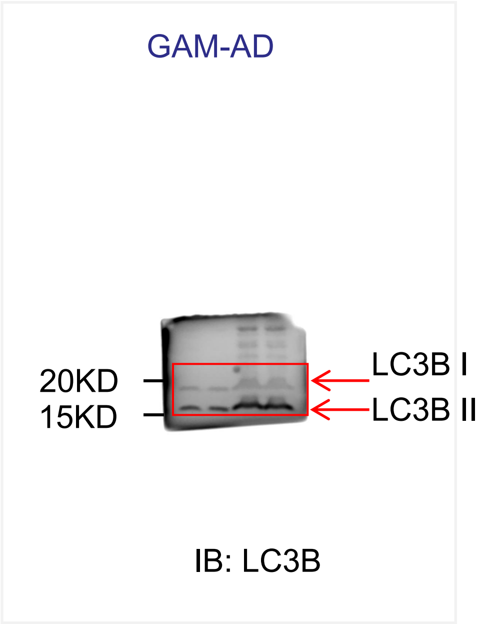

GAM-AD
20KD
15KD
LC3B I
LC3B II
IB: LC3B

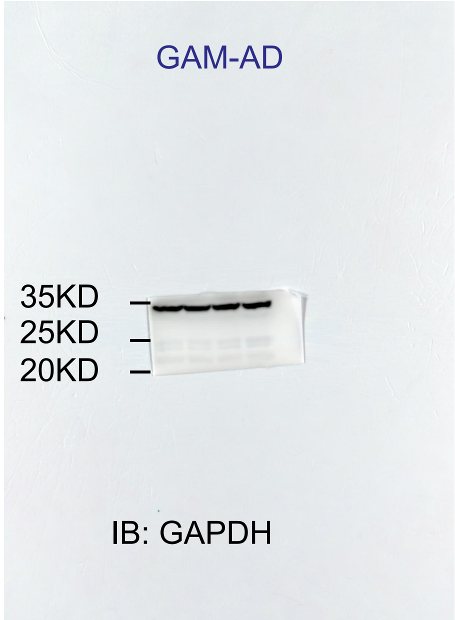

GAM-AD
35KD
25KD
20KD
IB: GAPDH

Figure 5J

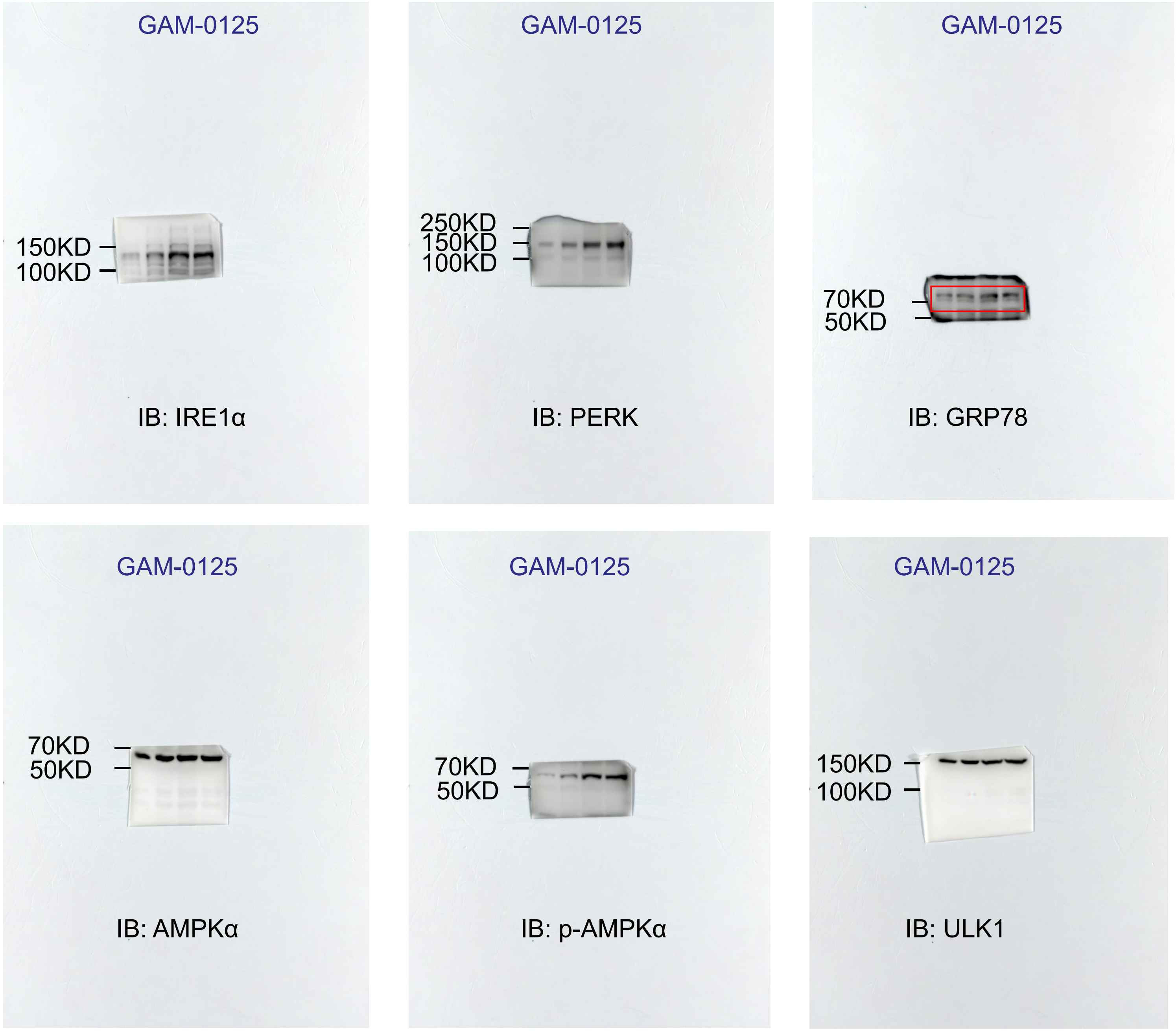
GAM-0125
150KD
100KD
IB: IRE1α
GAM-0125
250KD
150KD
100KD
IB: PERK
GAM-0125
70KD
50KD
IB: GRP78
GAM-0125
70KD
50KD
IB: AMPKα
GAM-0125
70KD
50KD
IB: p-AMPKα
GAM-0125
150KD
100KD
IB: ULK1

Figure 5J

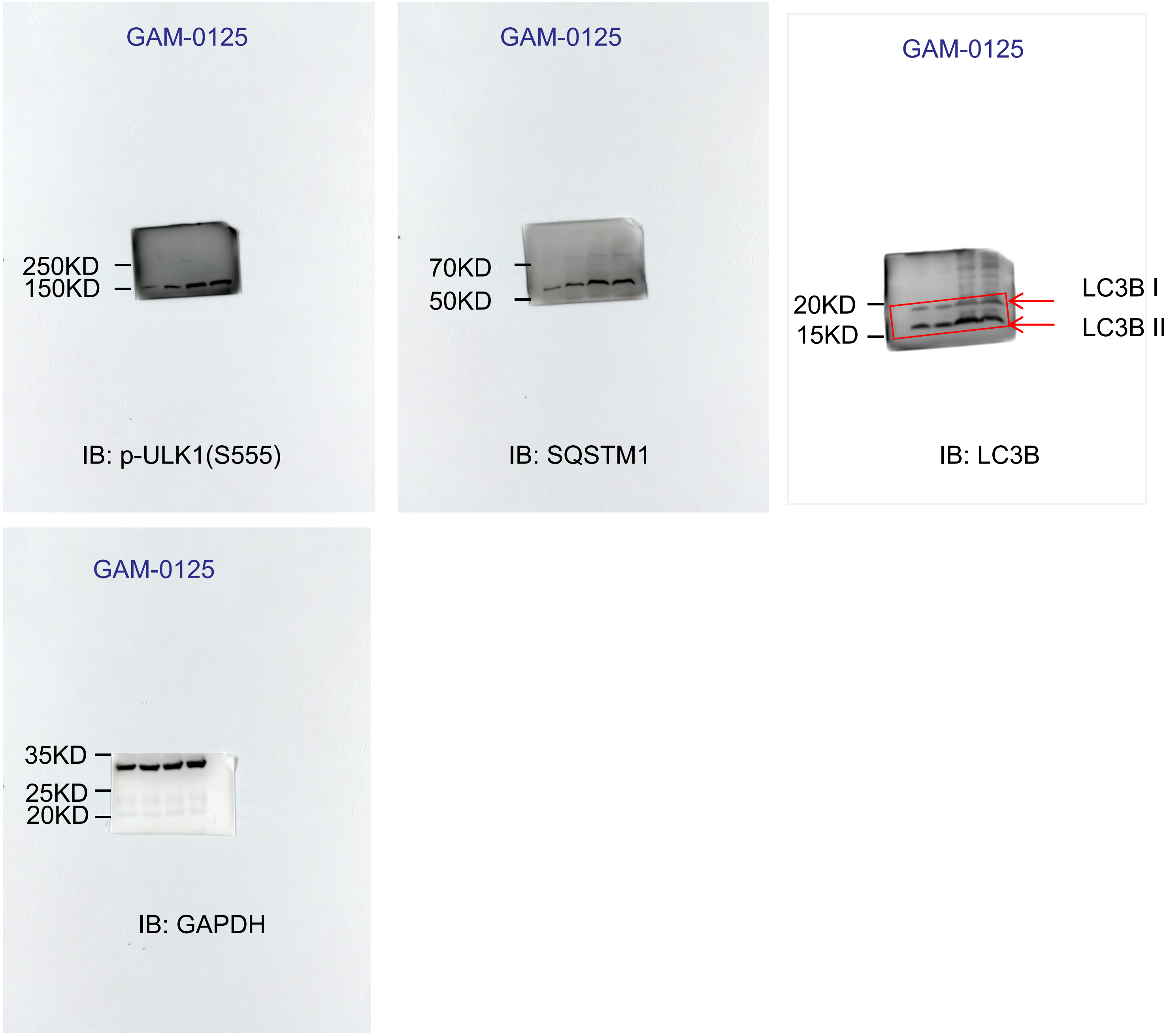
GAM-0125
250KD
150KD
IB: p-ULK1(S555)
GAM-0125
70KD
50KD
IB: SQSTM1
GAM-0125
20KD
15KD
LC3B I
LC3B II
IB: LC3B
GAM-0125
35KD
25KD
20KD
IB: GAPDH

Figure 5J

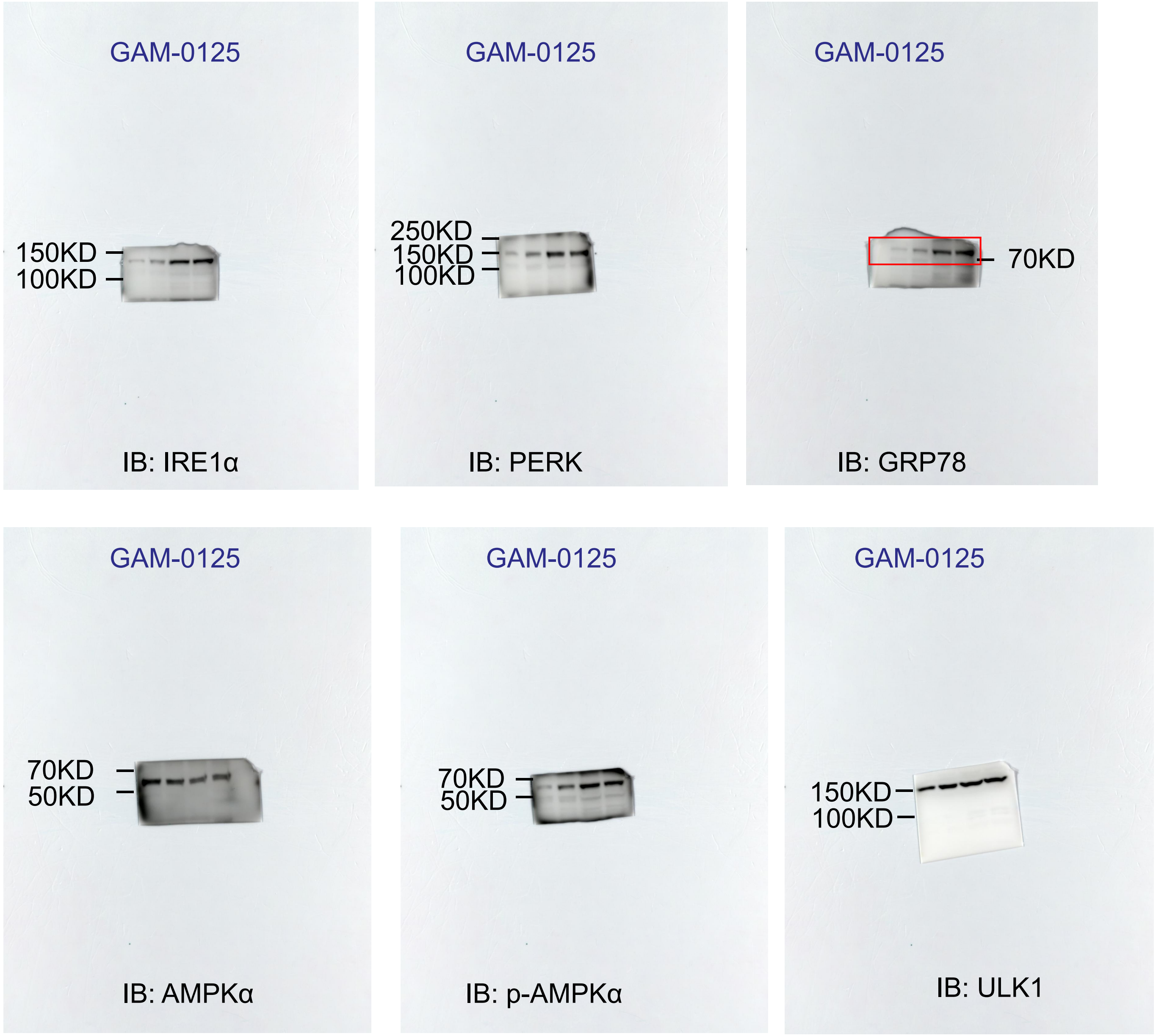
GAM-0125
150KD
100KD
IB: IRE1α
GAM-0125
250KD
150KD
100KD
IB: PERK
GAM-0125
70KD
IB: GRP78
GAM-0125
70KD
50KD
IB: AMPKα
GAM-0125
70KD
50KD
IB: p-AMPKα
GAM-0125
150KD
100KD
IB: ULK1

Figure 5J

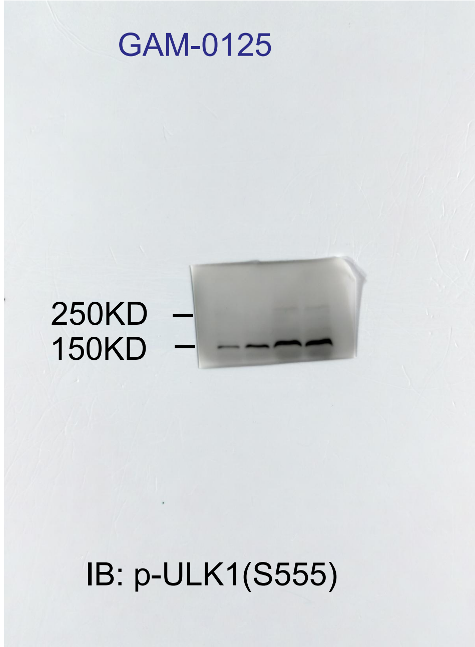
GAM-0125
250KD
150KD
IB: p-ULK1(S555)

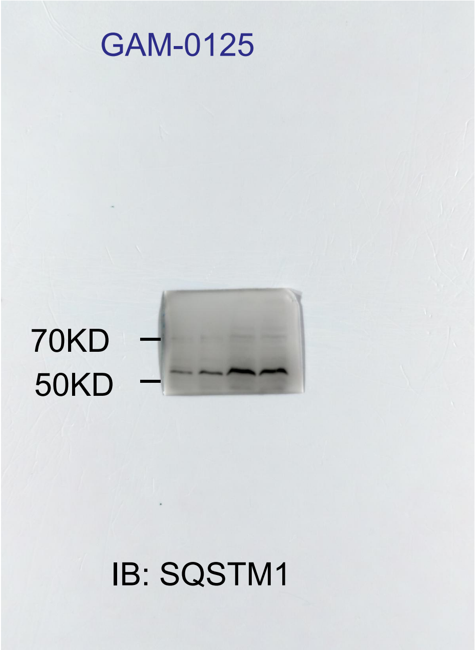
GAM-0125
70KD
50KD
IB: SQSTM1

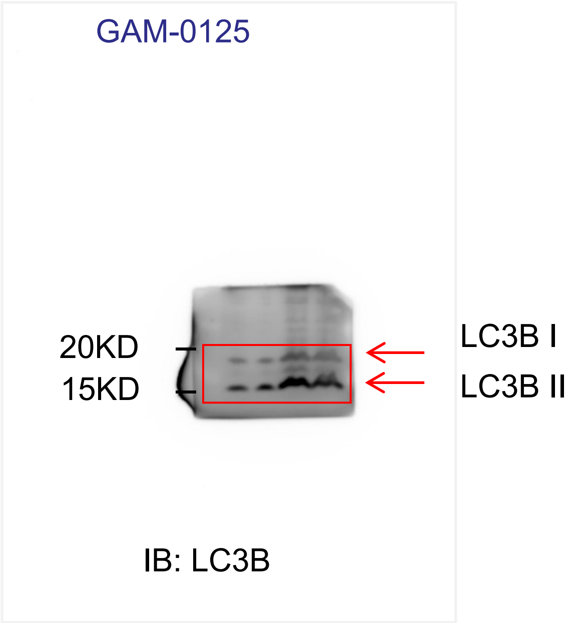
GAM-0125
20KD
15KD
LC3B I
LC3B II
IB: LC3B

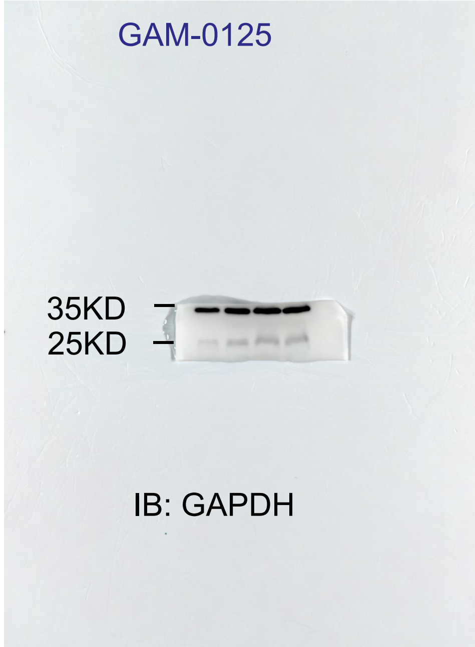
GAM-0125
35KD
25KD
IB: GAPDH

Figure 6A

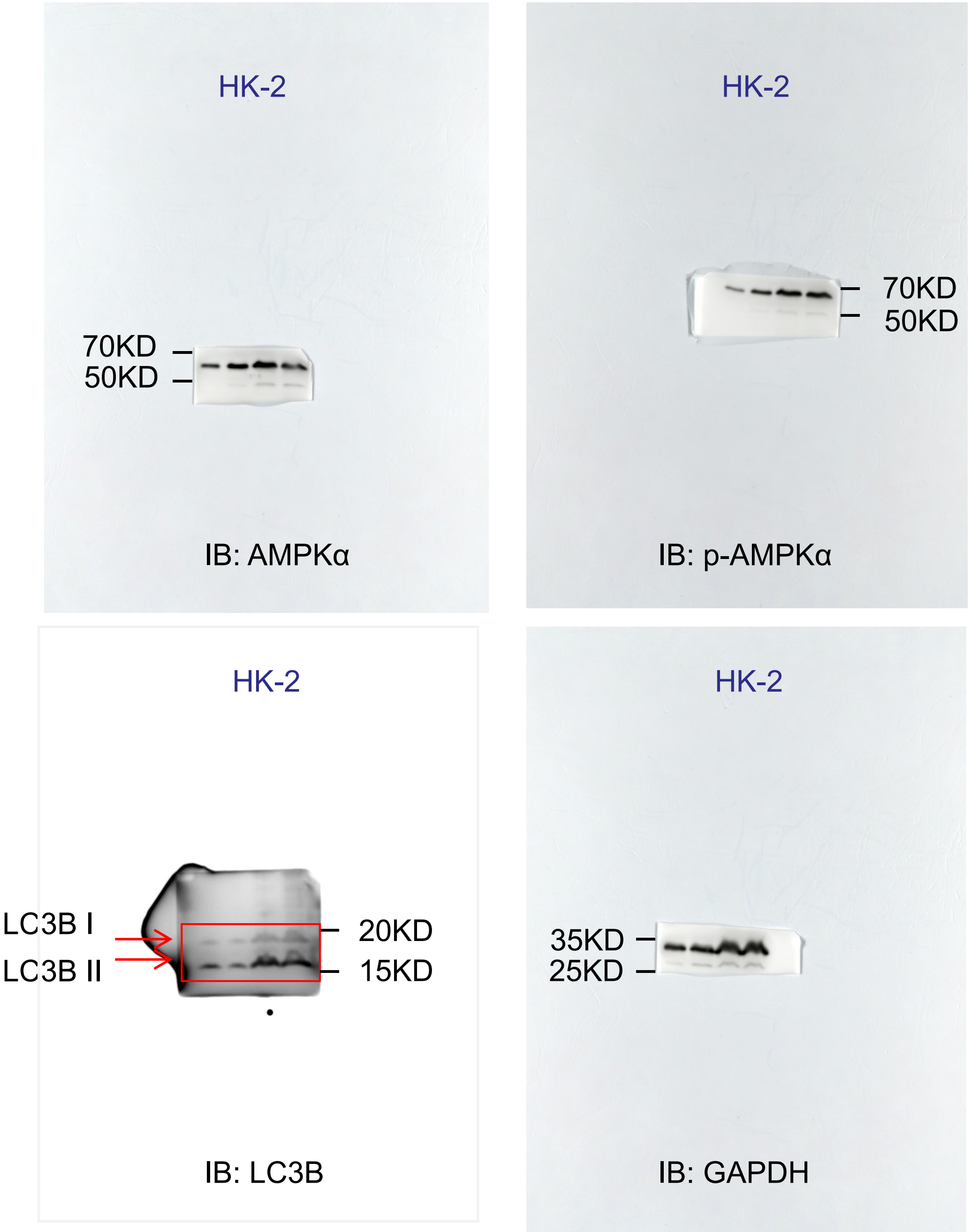

HK-2
70KD
50KD
IB: AMPKα
HK-2
70KD
50KD
IB: p-AMPKα
HK-2
LC3B I
LC3B II
20KD
15KD
IB: LC3B
HK-2
35KD
25KD
IB: GAPDH

Figure 6A

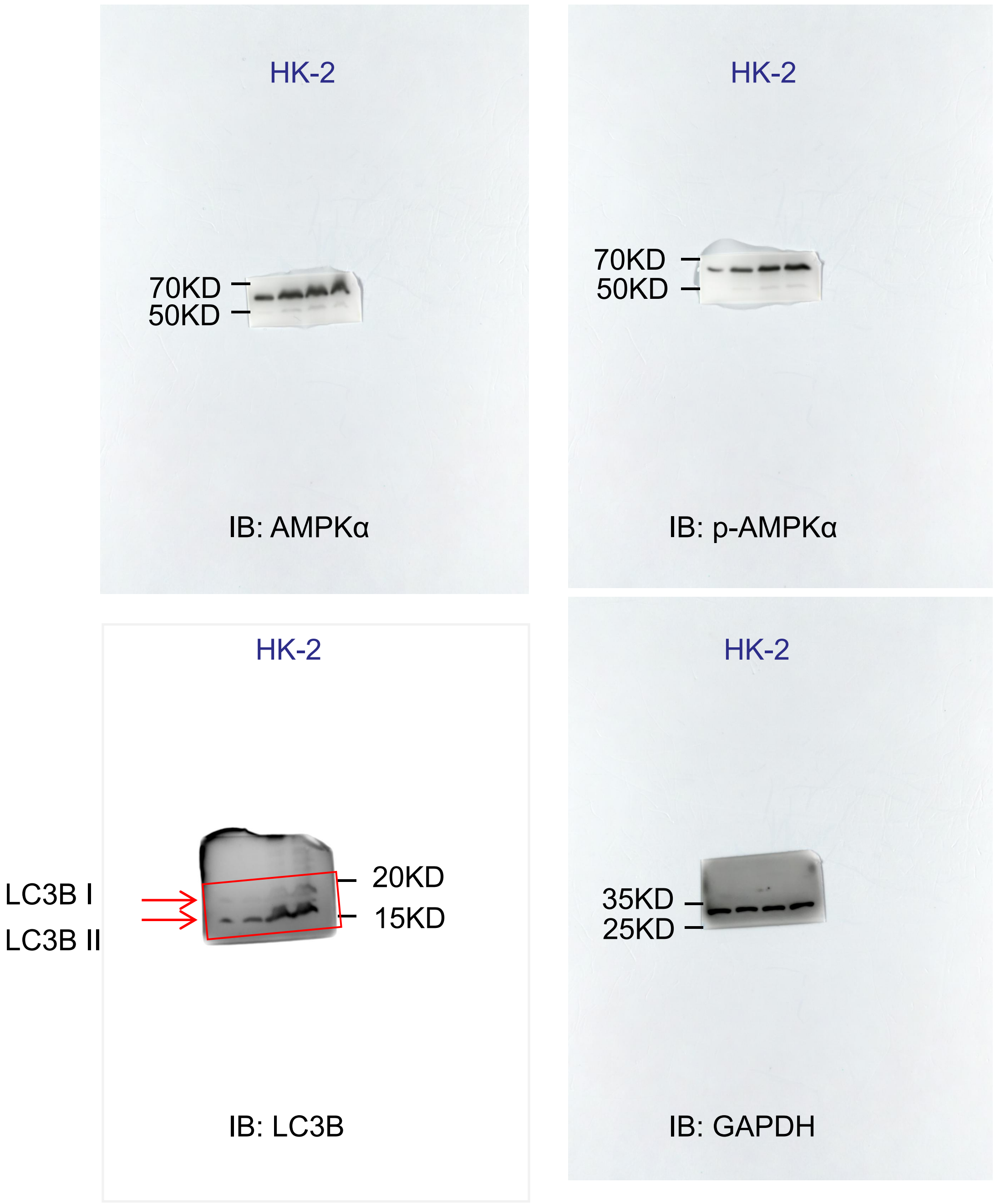

HK-2
70KD
50KD
IB: AMPKα
HK-2
70KD
50KD
IB: p-AMPKα
HK-2
LC3B I
LC3B II
20KD
15KD
IB: LC3B
HK-2
35KD
25KD
IB: GAPDH

Figure 6A

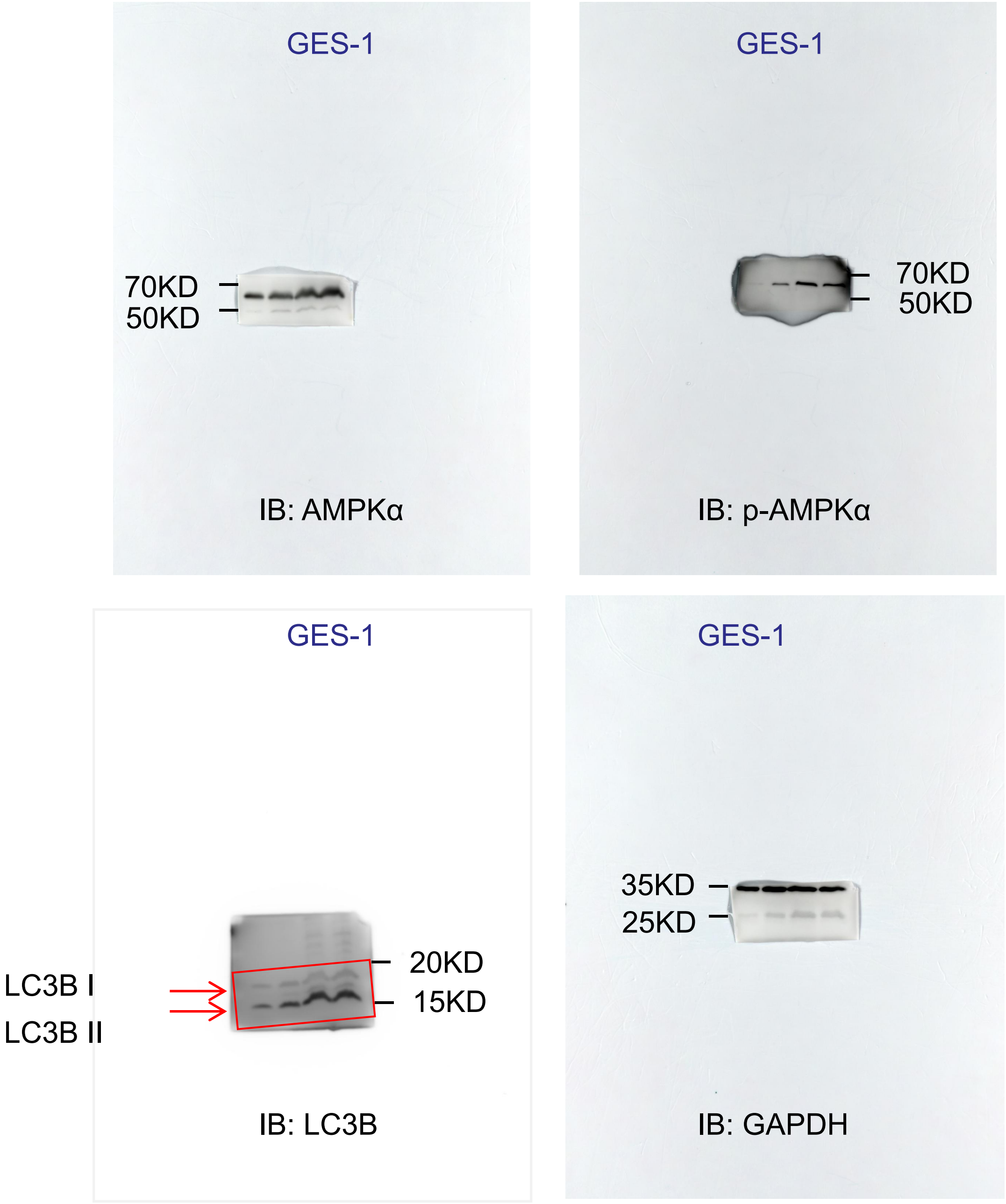
GES-1
70KD
50KD
IB: AMPKα
GES-1
70KD
50KD
IB: p-AMPKα
GES-1
LC3B I
LC3B II
20KD
15KD
IB: LC3B
GES-1
35KD
25KD
IB: GAPDH

Figure 6A

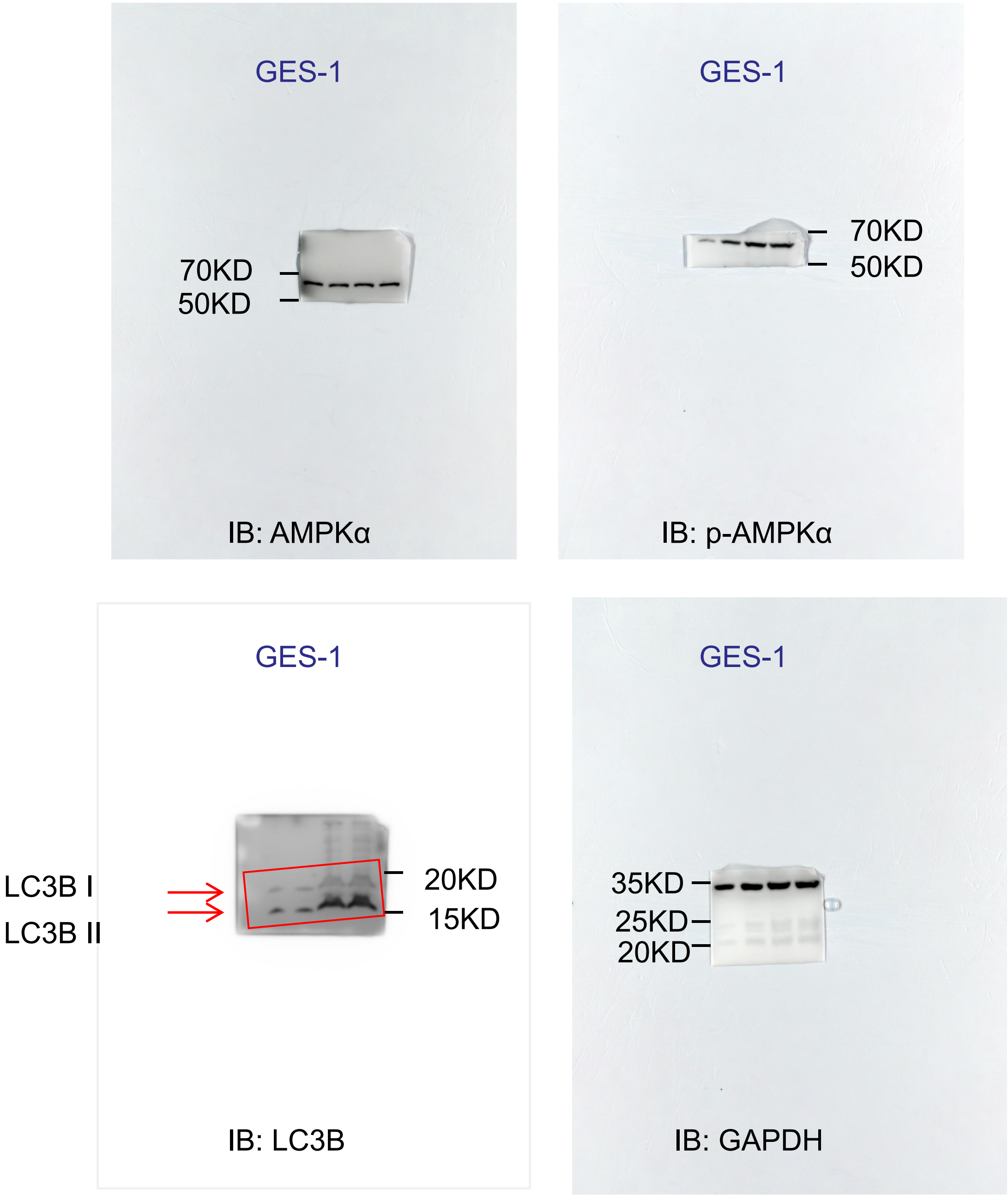
GES-1
70KD
50KD
IB: AMPKα
GES-1
70KD
50KD
IB: p-AMPKα
GES-1
LC3B I
LC3B II
20KD
15KD
IB: LC3B
GES-1
35KD
25KD
20KD
IB: GAPDH

Figure 6B

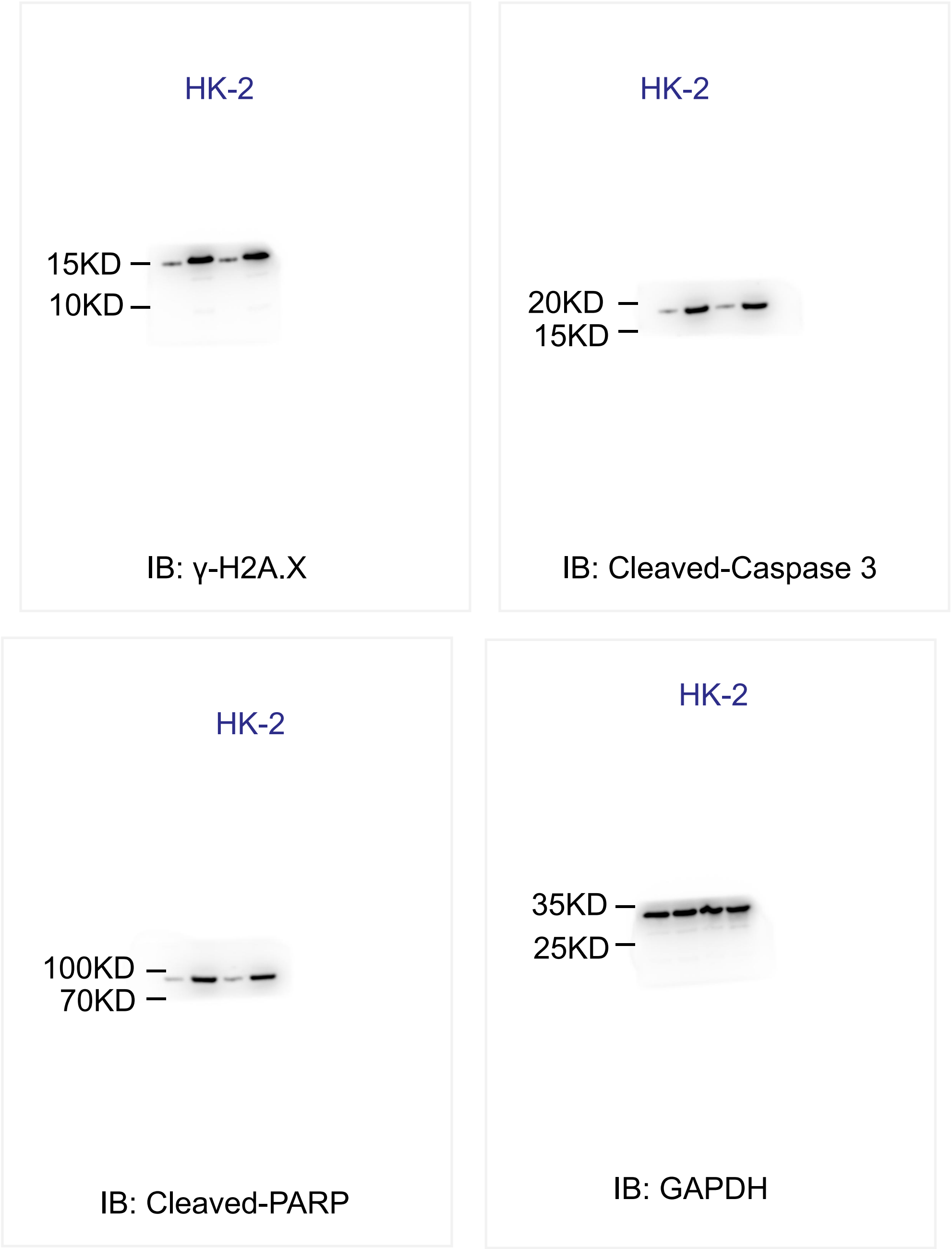
HK-2
15KD
10KD
IB: γ-H2A.X
HK-2
20KD
15KD
IB: Cleaved-Caspase 3
HK-2
100KD
70KD
IB: Cleaved-PARP
HK-2
35KD
25KD
IB: GAPDH

Figure 6B

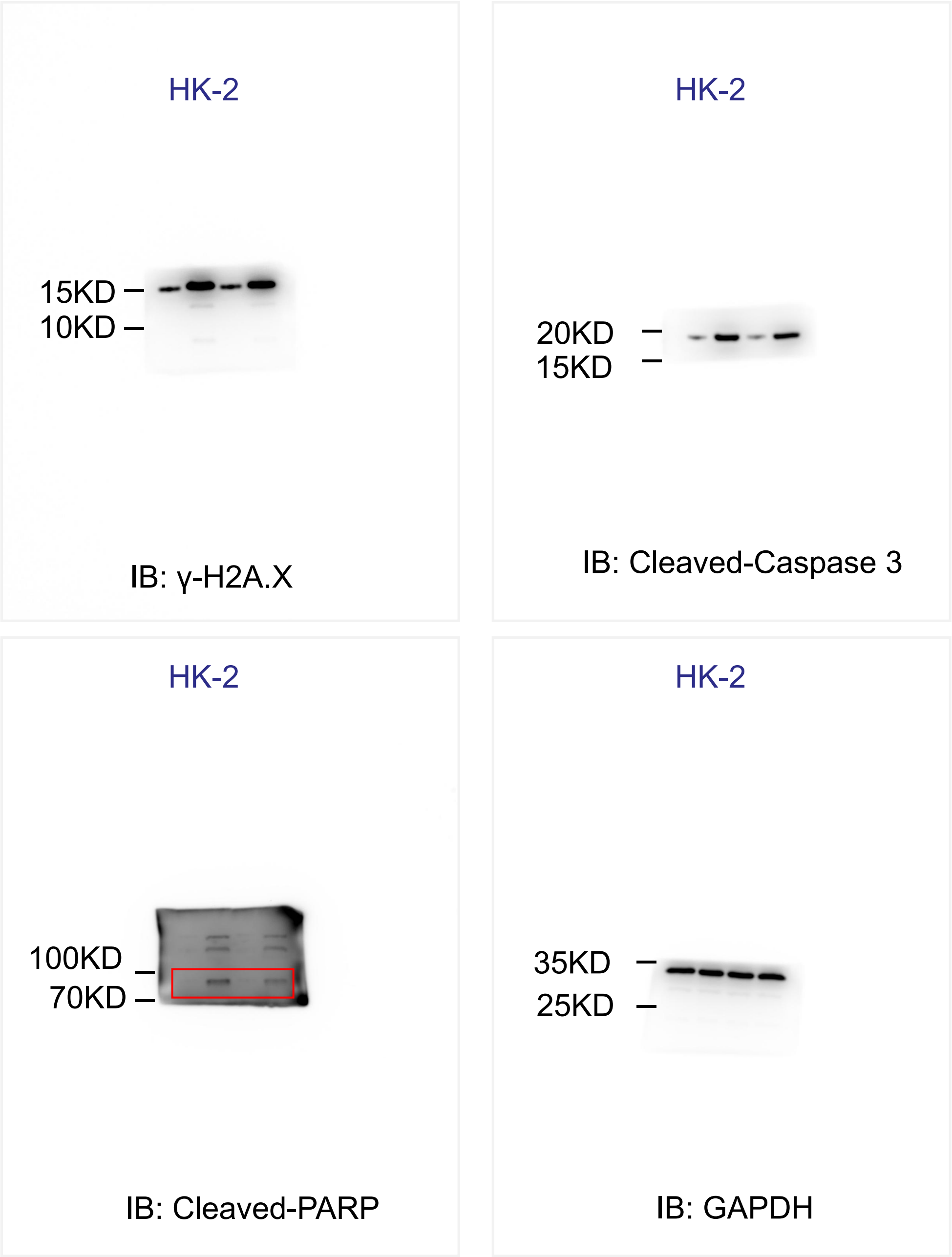
HK-2
15KD
10KD
IB: γ-H2A.X
HK-2
20KD
15KD
IB: Cleaved-Caspase 3
HK-2
100KD
70KD
IB: Cleaved-PARP
HK-2
35KD
25KD
IB: GAPDH

Figure 6B

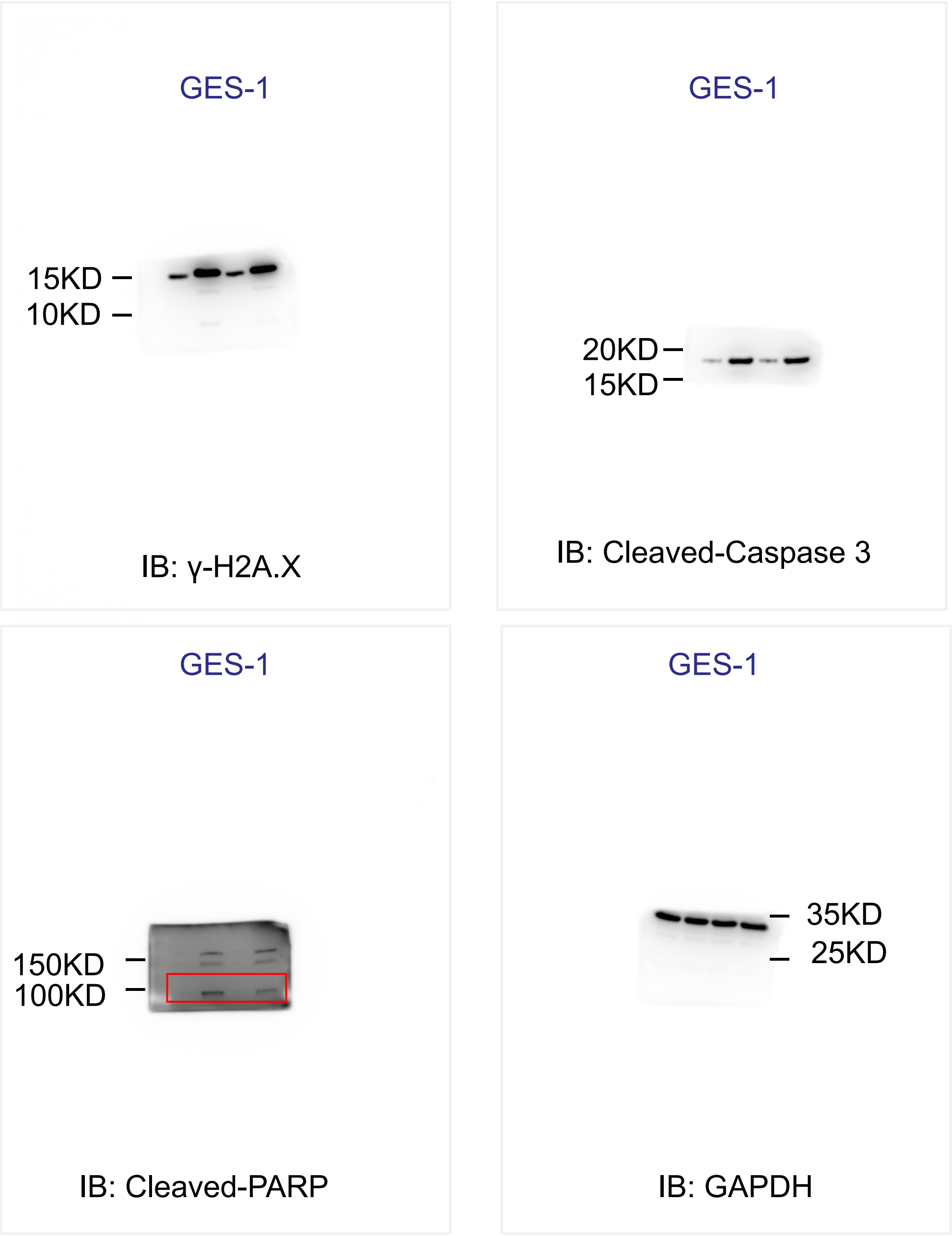
GES-1
15KD
10KD
IB: γ-H2A.X
GES-1
20KD
15KD
IB: Cleaved-Caspase 3
GES-1
150KD
100KD
IB: Cleaved-PARP
GES-1
35KD
25KD
IB: GAPDH

Figure 6B

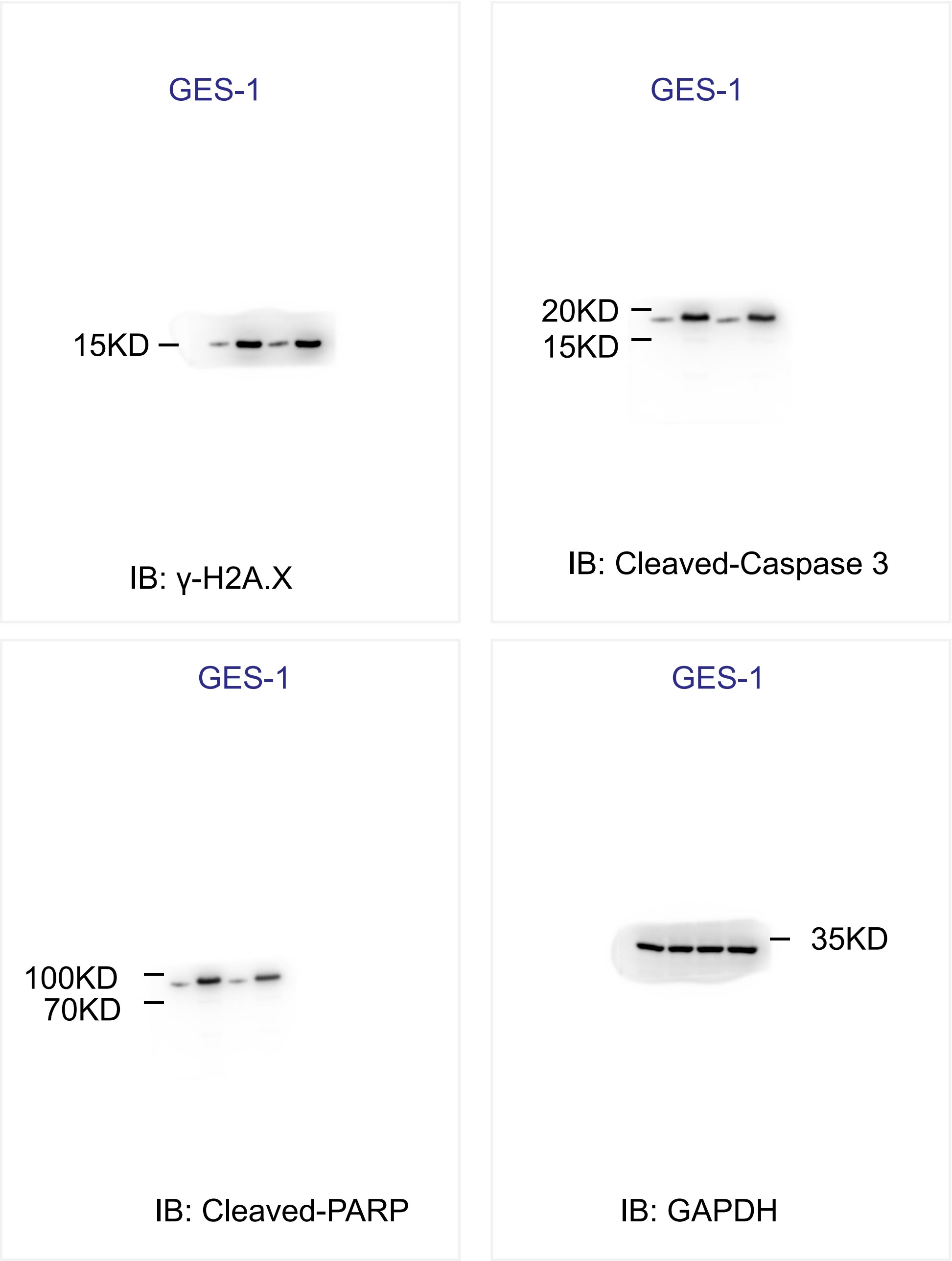
GES-1
15KD
IB: γ-H2A.X
GES-1
20KD
15KD
IB: Cleaved-Caspase 3
GES-1
100KD
70KD
IB: Cleaved-PARP
GES-1
35KD
IB: GAPDH

Figure 6C

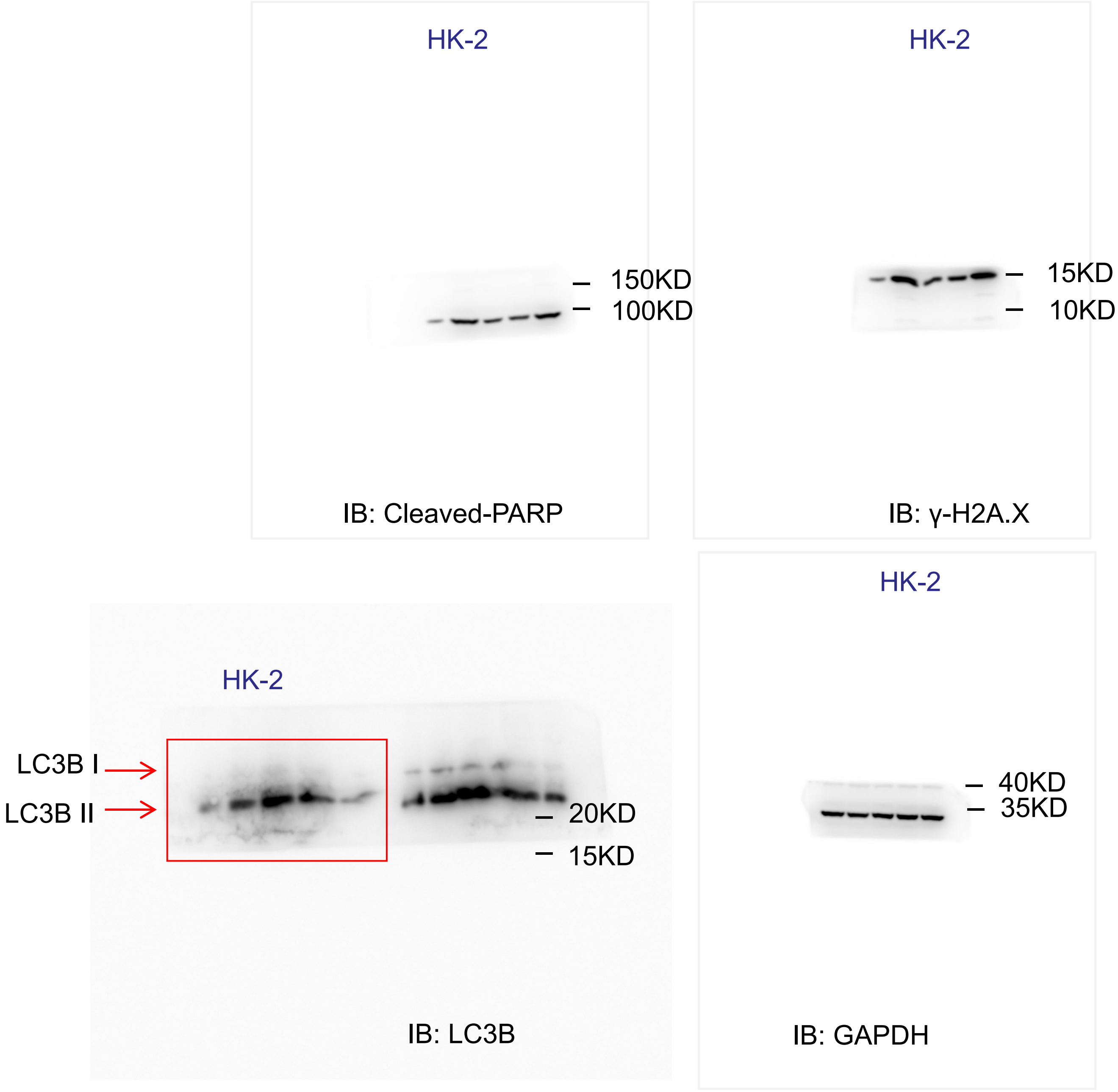
HK-2
150KD
100KD
IB: Cleaved-PARP
HK-2
15KD
10KD
IB: γ-H2A.X
HK-2
LC3B I
LC3B II
20KD
15KD
IB: LC3B
HK-2
40KD
35KD
IB: GAPDH

Figure 6C

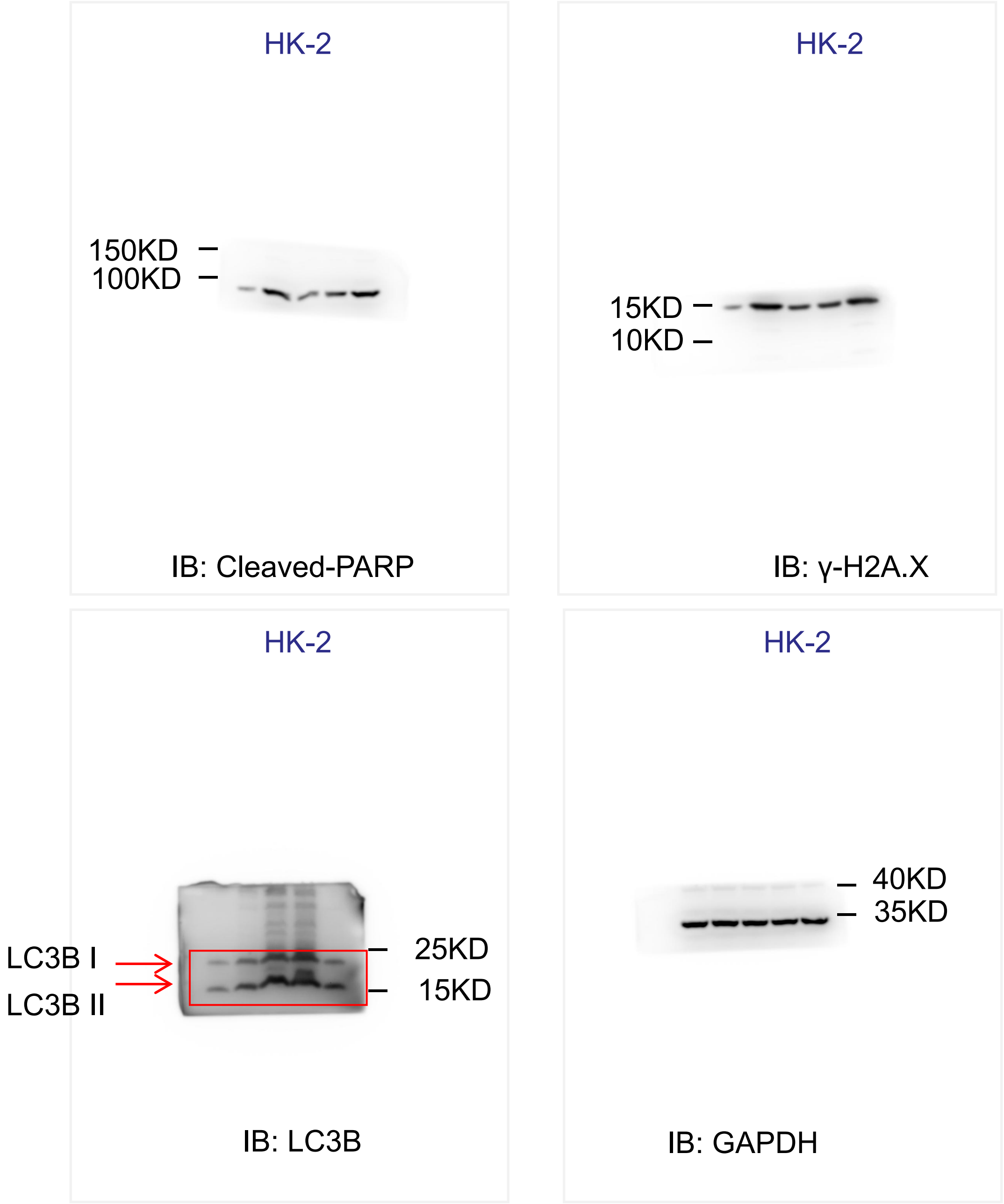
HK-2
150KD
100KD
IB: Cleaved-PARP
HK-2
15KD
10KD
IB: γ-H2A.X
HK-2
LC3B I
LC3B II
25KD
15KD
IB: LC3B
HK-2
40KD
35KD
IB: GAPDH

Figure 6C

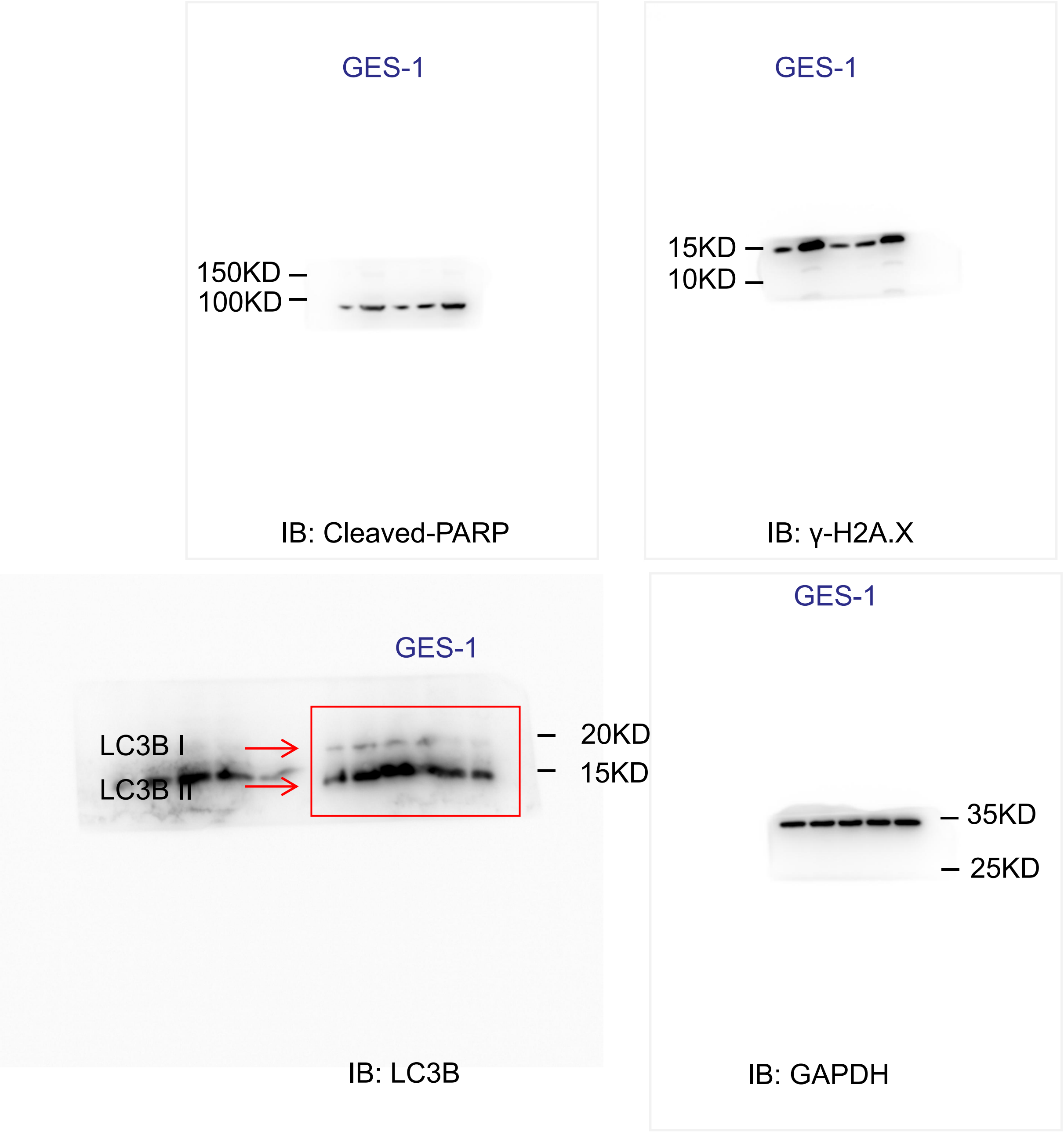
GES-1
150KD
100KD
IB: Cleaved-PARP
GES-1
15KD
10KD
IB: γ-H2A.X
GES-1
LC3B I
LC3B II
20KD
15KD
IB: LC3B
GES-1
35KD
25KD
IB: GAPDH

Figure 6C

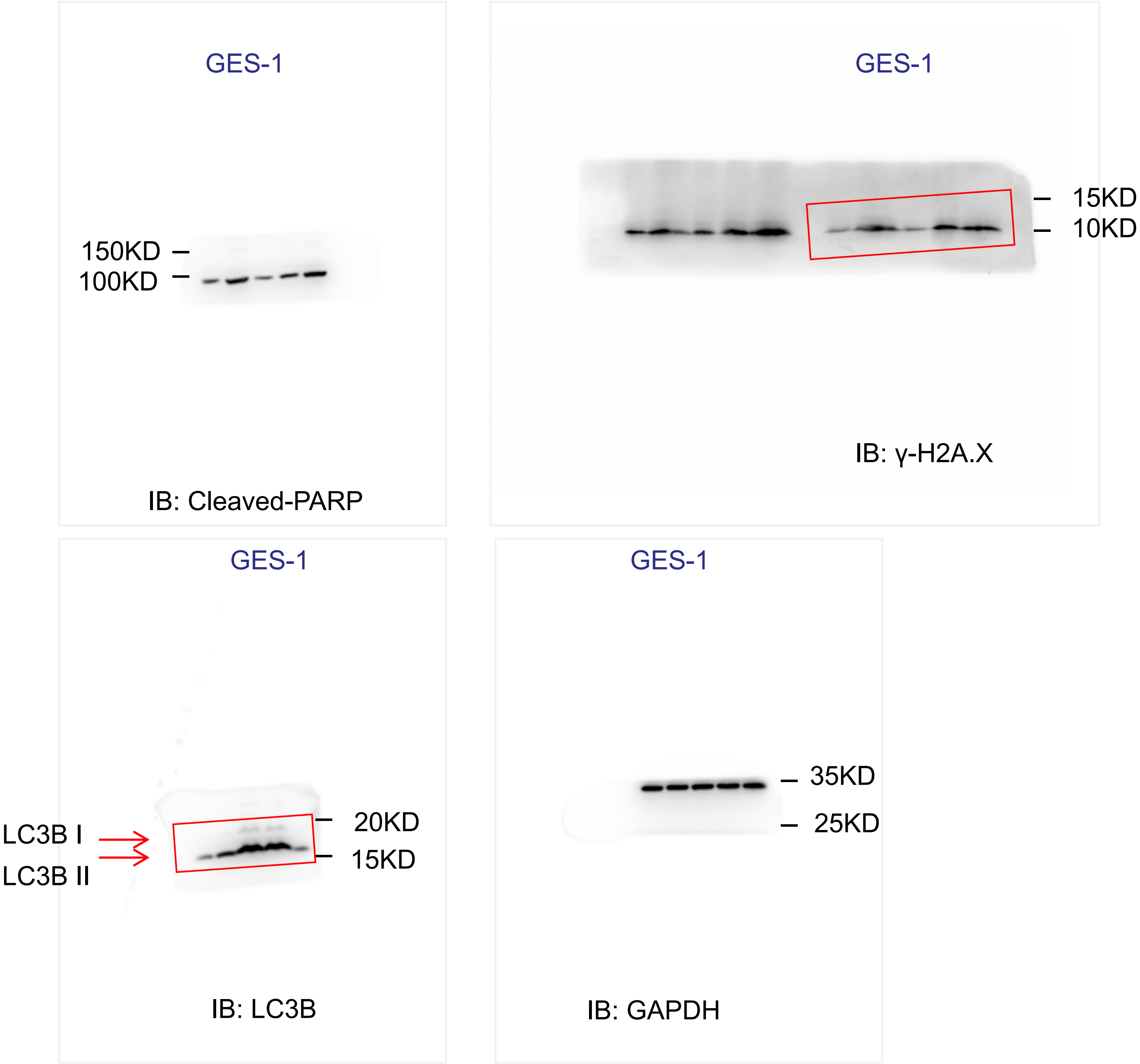
GES-1
150KD
100KD
IB: Cleaved-PARP
GES-1
15KD
10KD
IB: γ-H2A.X
GES-1
LC3B I
LC3B II
20KD
15KD
IB: LC3B
GES-1
35KD
25KD
IB: GAPDH

Figure 6G

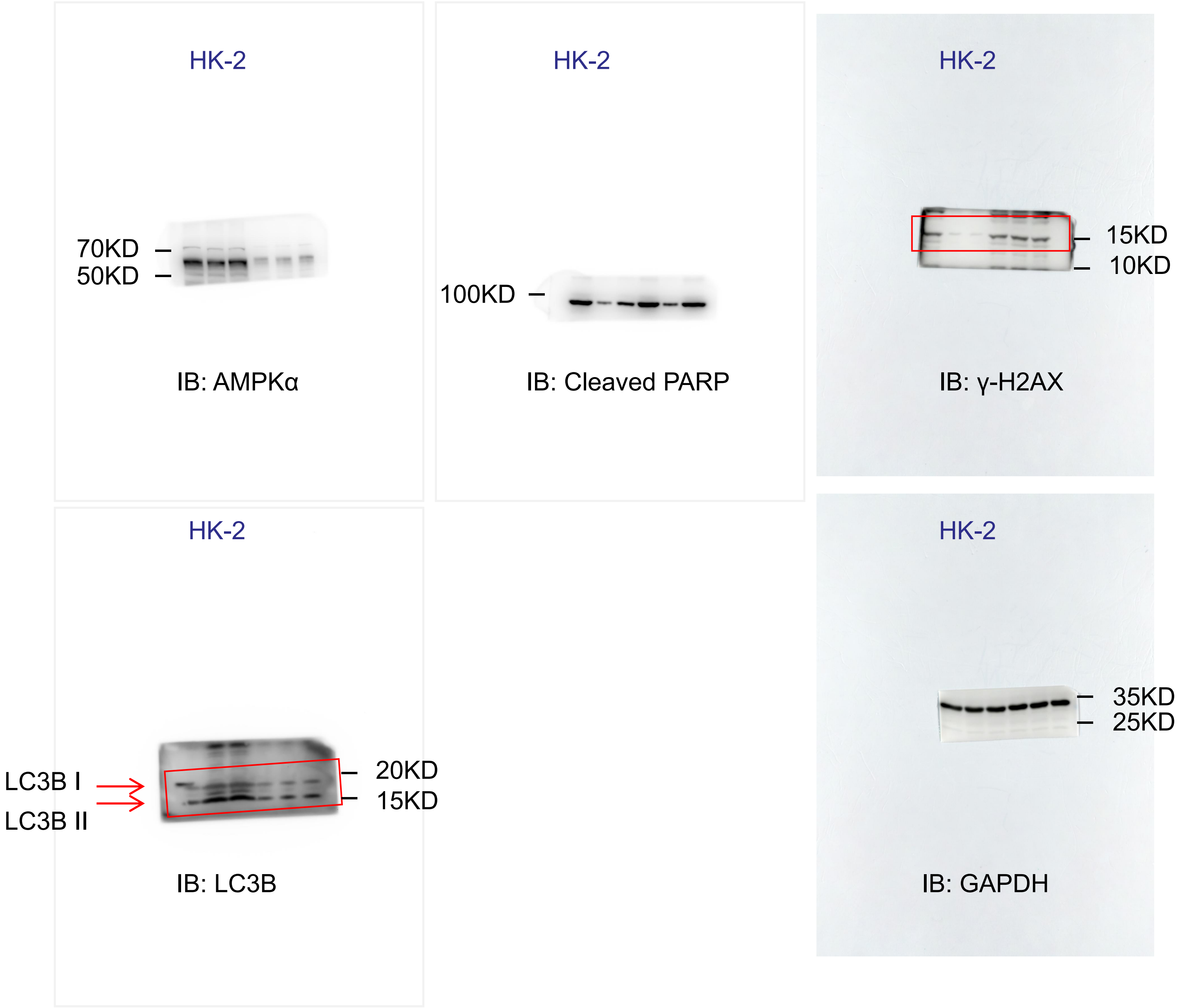
HK-2
70KD
50KD
IB: AMPKα
HK-2
100KD
IB: Cleaved PARP
HK-2
15KD
10KD
IB: γ-H2AX
HK-2
LC3B I
LC3B II
20KD
15KD
IB: LC3B
HK-2
35KD
25KD
IB: GAPDH

Figure 6G

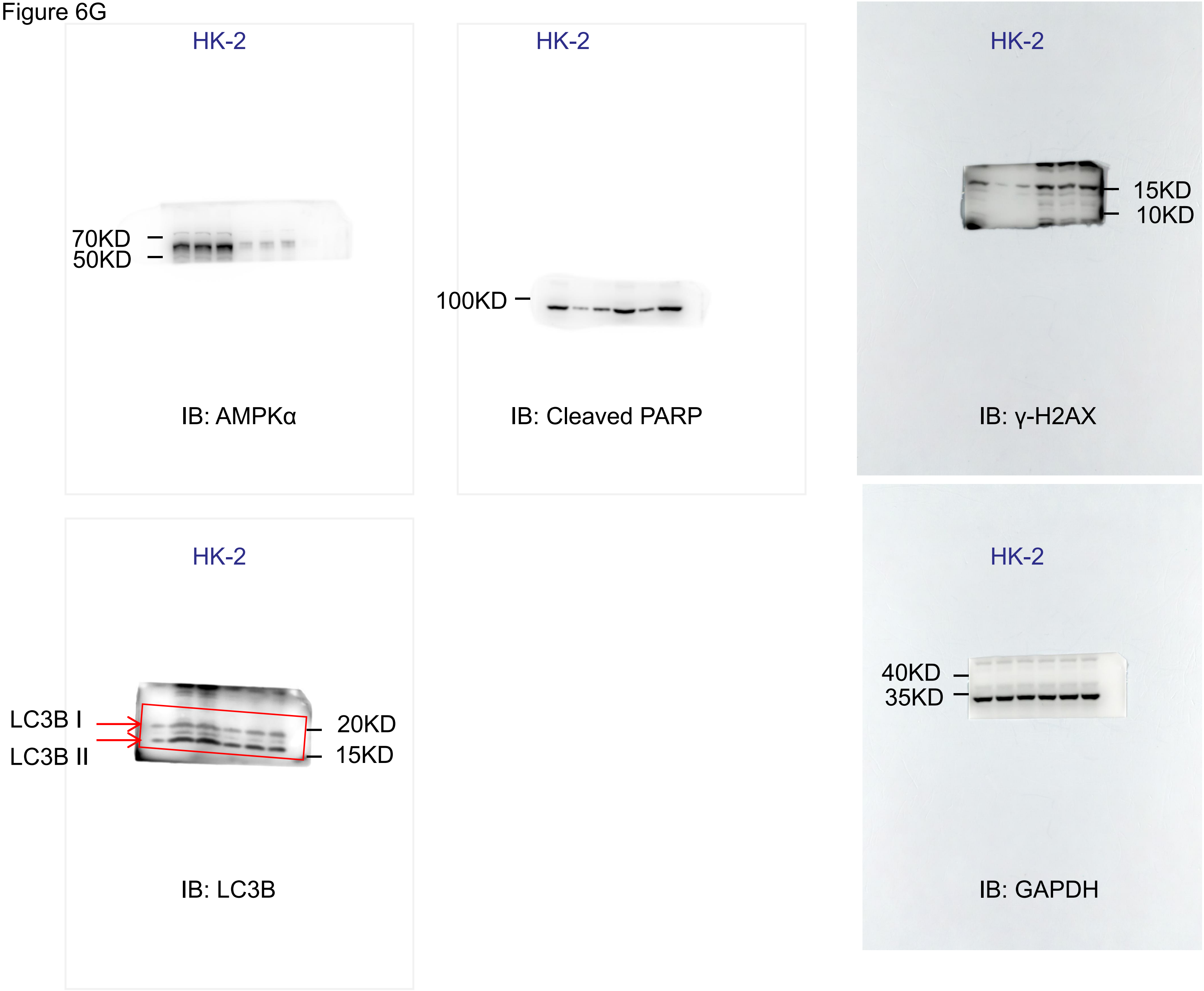
HK-2
70KD
50KD
IB: AMPKα
HK-2
100KD
IB: Cleaved PARP
HK-2
15KD
10KD
IB: γ-H2AX
HK-2
LC3B I
LC3B II
20KD
15KD
IB: LC3B
HK-2
40KD
35KD
IB: GAPDH

Figure 6G

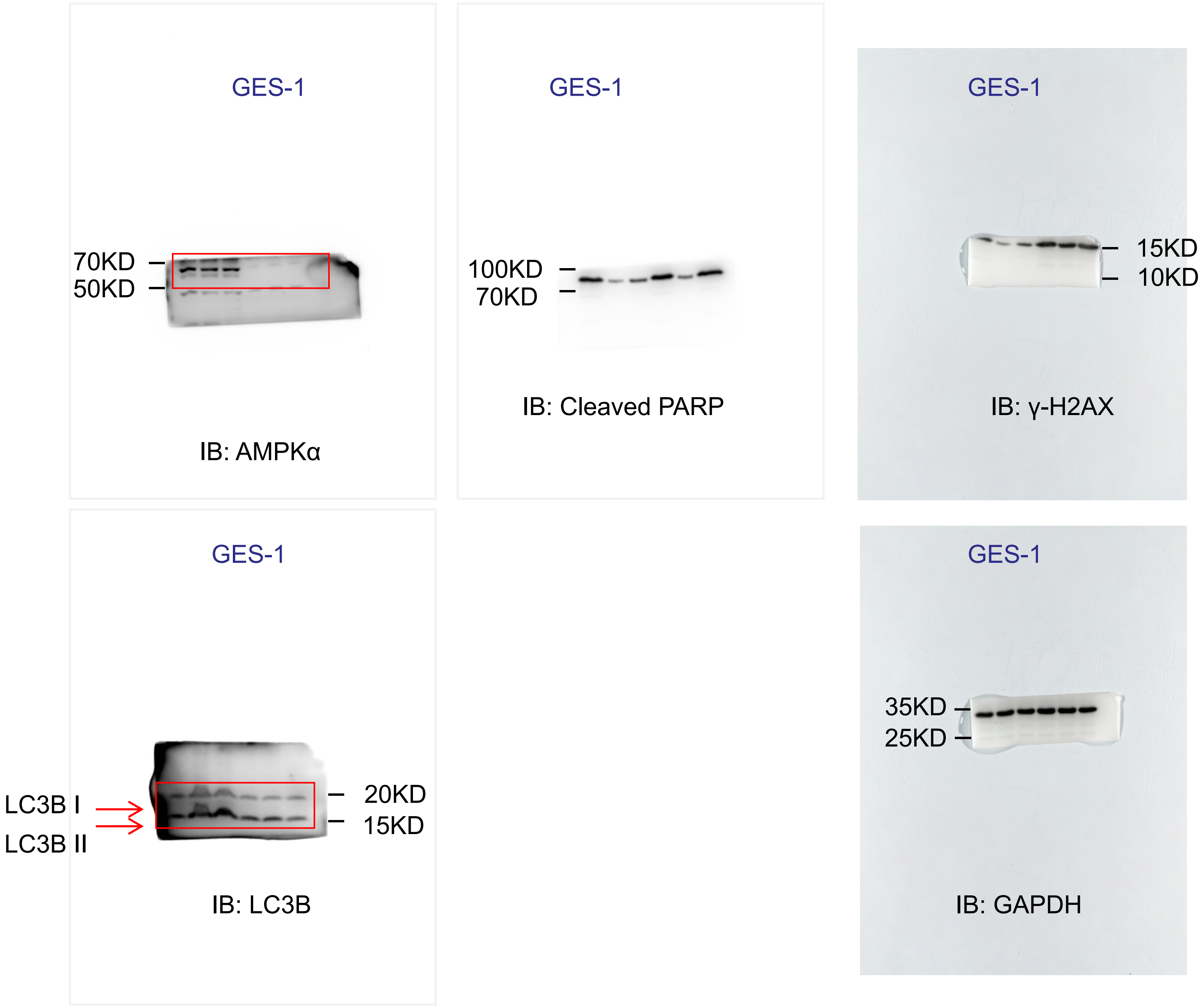
GES-1
70KD
50KD
IB: AMPKα
GES-1
100KD
70KD
IB: Cleaved PARP
GES-1
15KD
10KD
IB: γ-H2AX
GES-1
LC3B I
LC3B II
20KD
15KD
IB: LC3B
GES-1
35KD
25KD
IB: GAPDH

Figure 6G

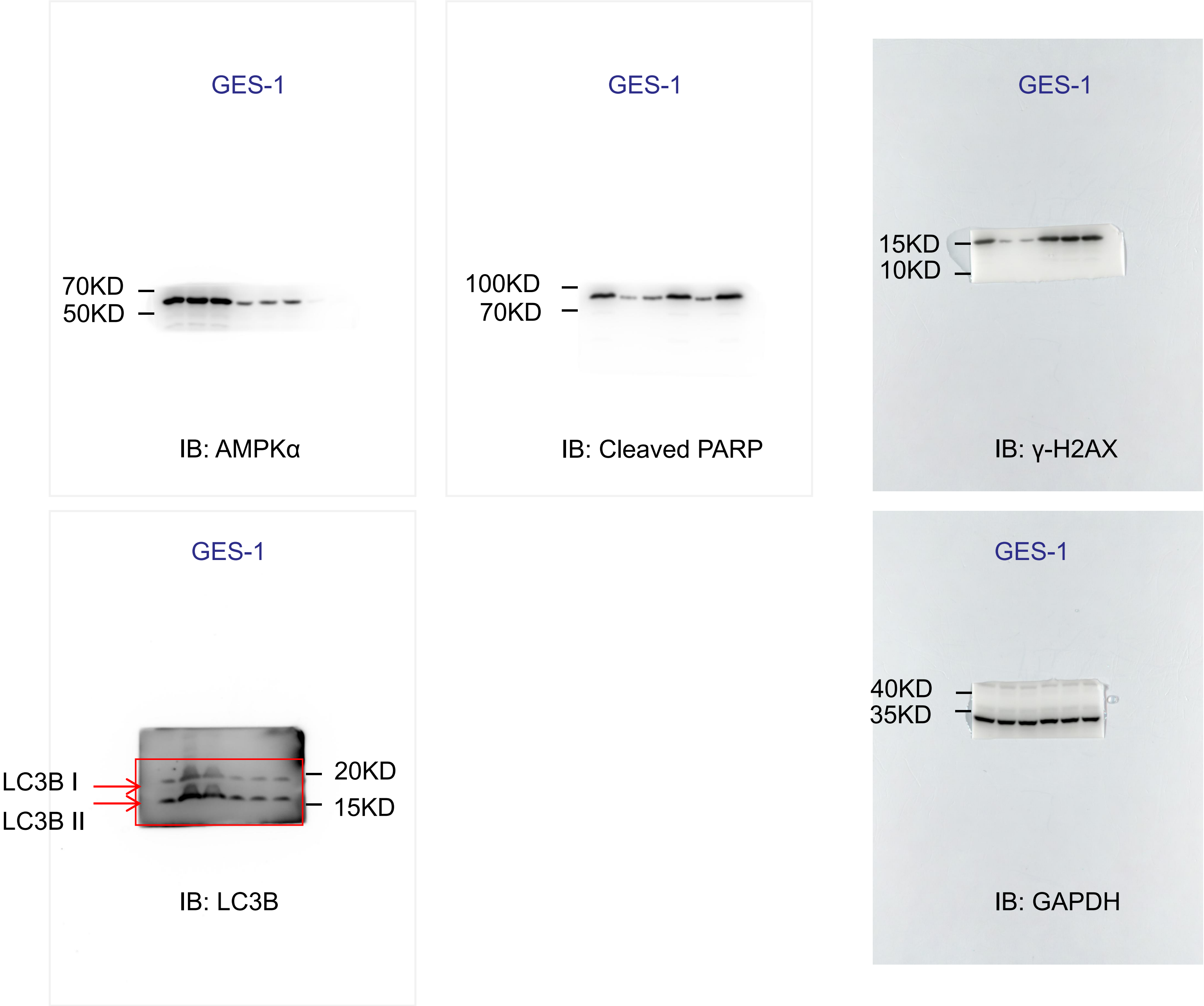
GES-1
70KD
50KD
IB: AMPKα
GES-1
100KD
70KD
IB: Cleaved PARP
GES-1
15KD
10KD
IB: γ-H2AX
GES-1
LC3B I
LC3B II
20KD
15KD
IB: LC3B
GES-1
40KD
35KD
IB: GAPDH

Figure S1J

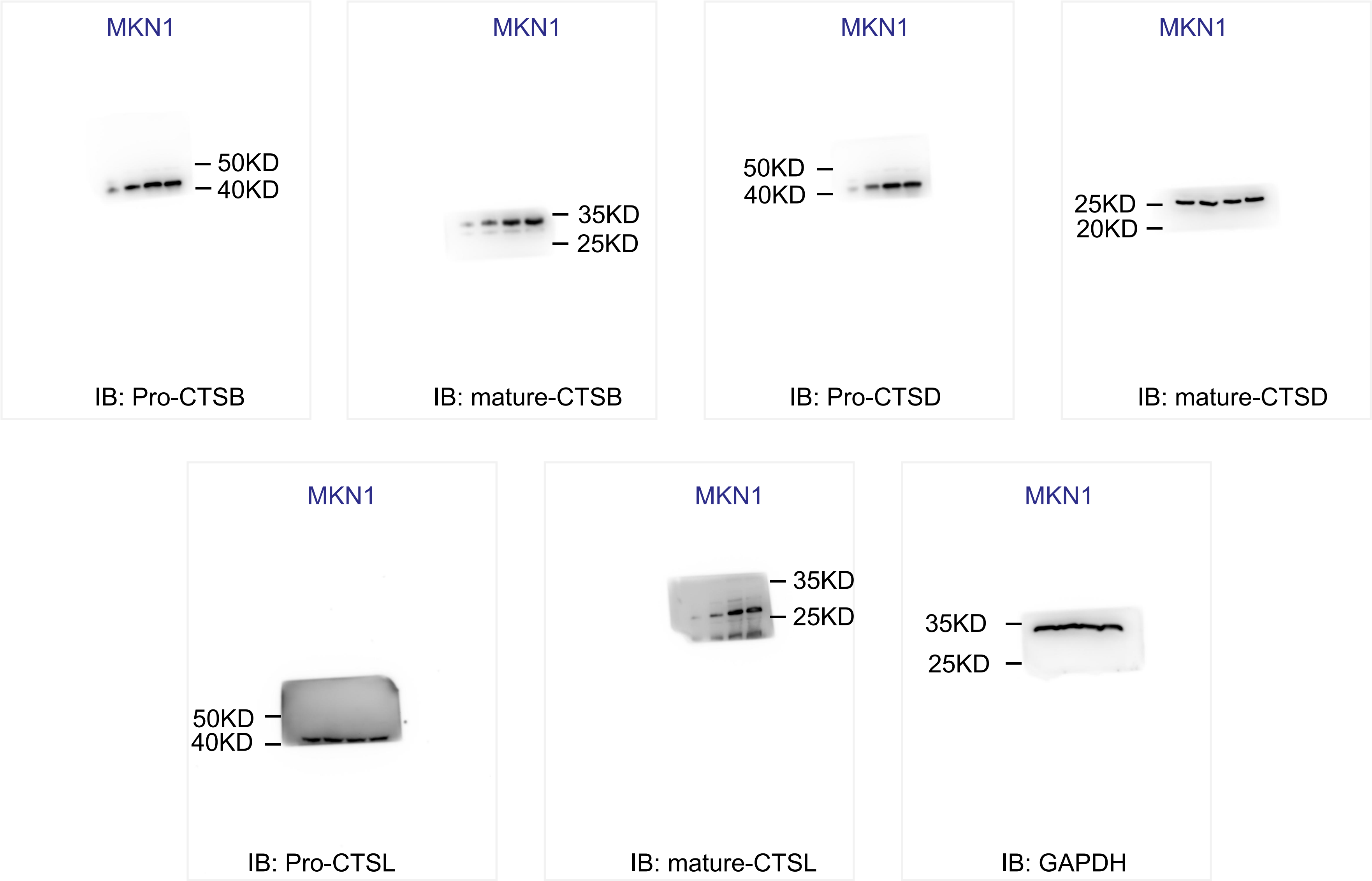
MKN1
50KD
40KD
IB: Pro-CTSB
MKN1
35KD
25KD
IB: mature-CTSB
MKN1
50KD
40KD
IB: Pro-CTSD
MKN1
25KD
20KD
IB: mature-CTSD
MKN1
50KD
40KD
IB: Pro-CTSL
MKN1
35KD
25KD
IB: mature-CTSL
MKN1
35KD
25KD
IB: GAPDH

Figure S1J

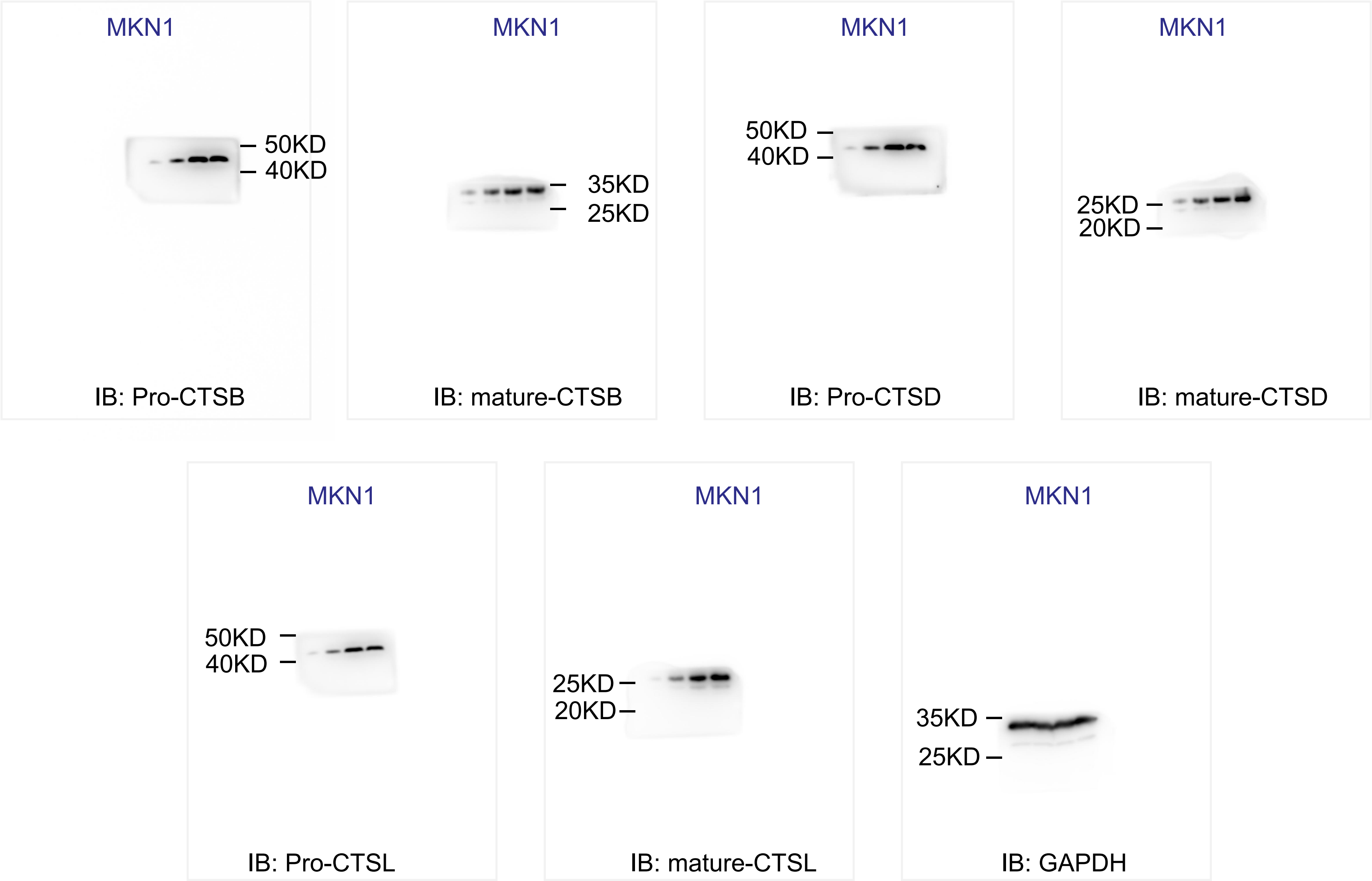
MKN1
50KD
40KD
IB: Pro-CTSB
MKN1
35KD
25KD
IB: mature-CTSB
MKN1
50KD
40KD
IB: Pro-CTSD
MKN1
25KD
20KD
IB: mature-CTSD
MKN1
50KD
40KD
IB: Pro-CTSL
MKN1
25KD
20KD
IB: mature-CTSL
MKN1
35KD
25KD
IB: GAPDH

Figure S1J

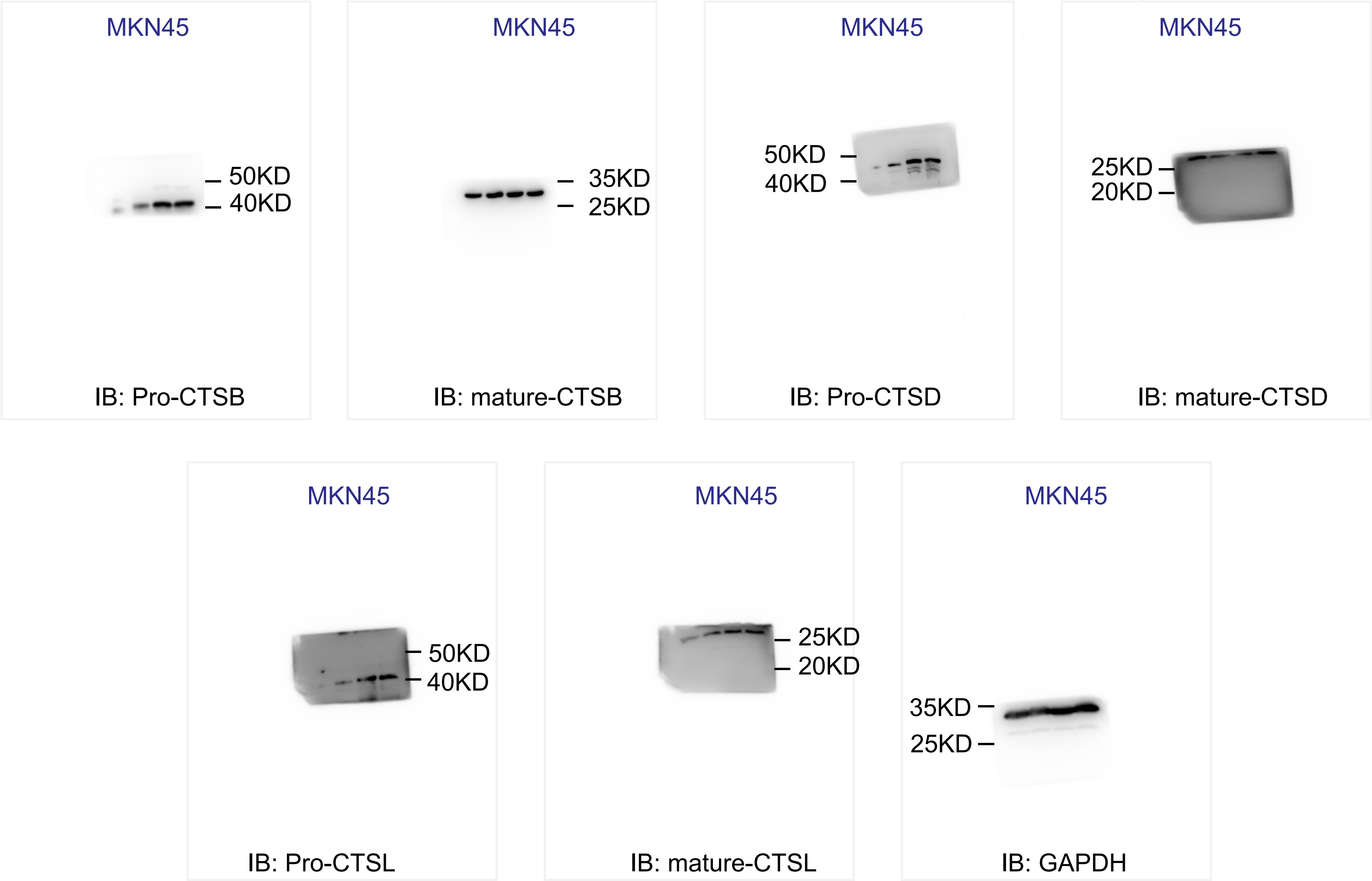
MKN45
50KD
40KD
IB: Pro-CTSB
MKN45
35KD
25KD
IB: mature-CTSB
MKN45
50KD
40KD
IB: Pro-CTSD
MKN45
25KD
20KD
IB: mature-CTSD
MKN45
50KD
40KD
IB: Pro-CTSL
MKN45
25KD
20KD
IB: mature-CTSL
MKN45
35KD
25KD
IB: GAPDH

Figure S1J

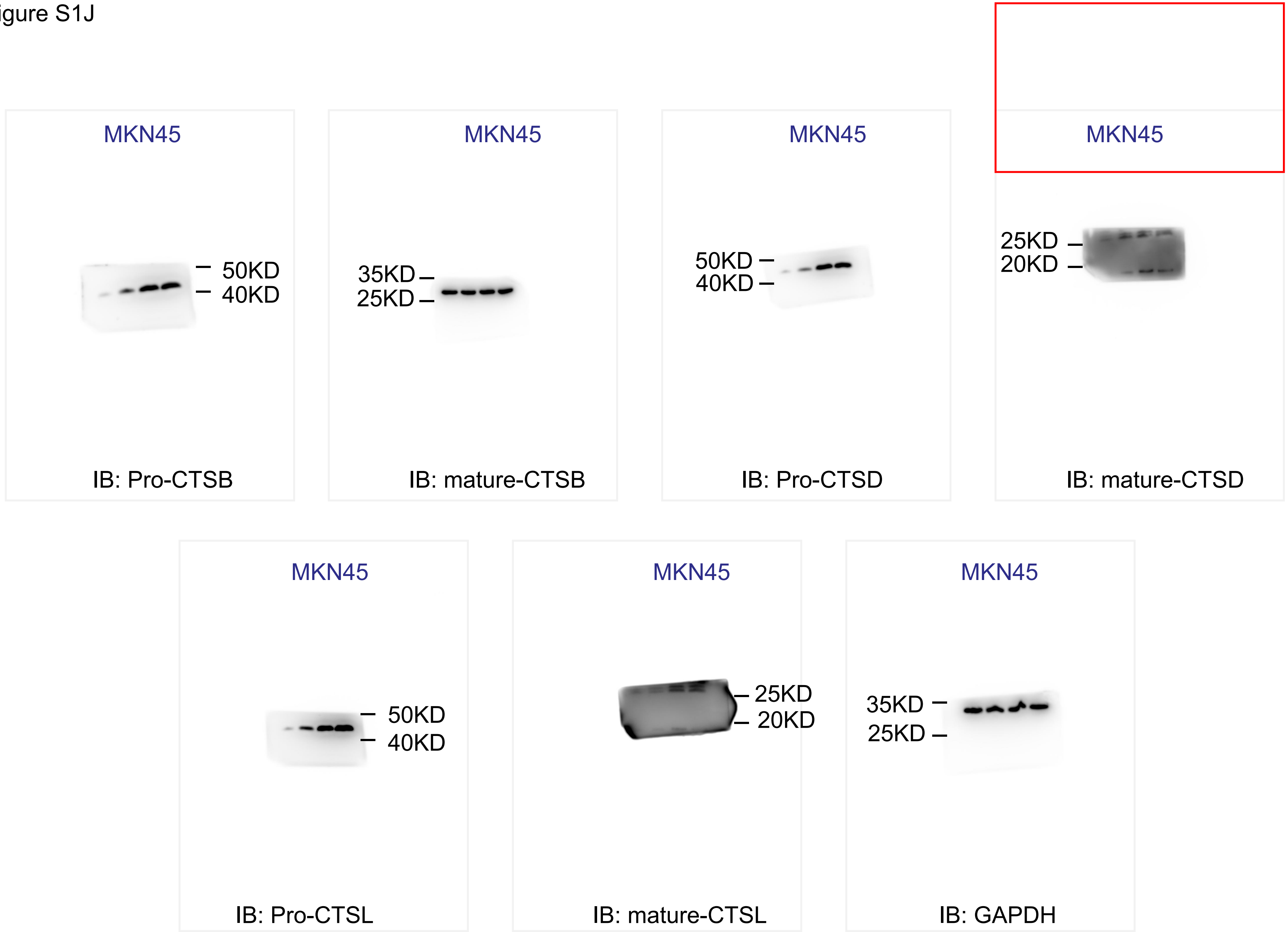
MKN45
50KD
40KD
IB: Pro-CTSB
MKN45
35KD
25KD
IB: mature-CTSB
MKN45
50KD
40KD
IB: Pro-CTSD
MKN45
25KD
20KD
IB: mature-CTSD
MKN45
50KD
40KD
IB: Pro-CTSL
MKN45
25KD
20KD
IB: mature-CTSL
MKN45
35KD
25KD
IB: GAPDH

Figure S2B

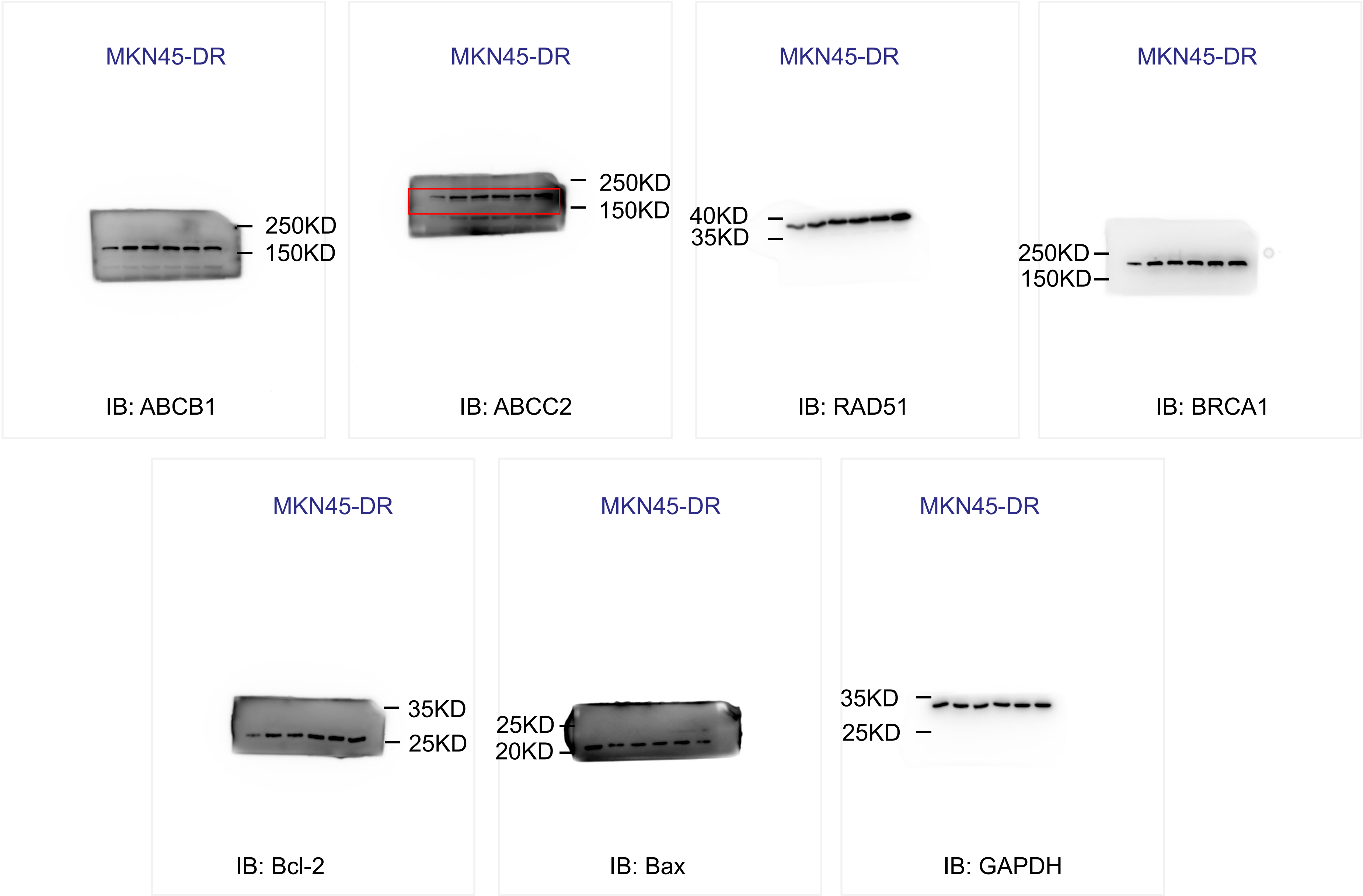
MKN45-DR
250KD
150KD
IB: ABCB1
MKN45-DR
250KD
150KD
IB: ABCC2
MKN45-DR
40KD
35KD
IB: RAD51
MKN45-DR
250KD
150KD
IB: BRCA1
MKN45-DR
35KD
25KD
IB: Bcl-2
MKN45-DR
25KD
20KD
IB: Bax
MKN45-DR
35KD
25KD
IB: GAPDH

Figure S2B

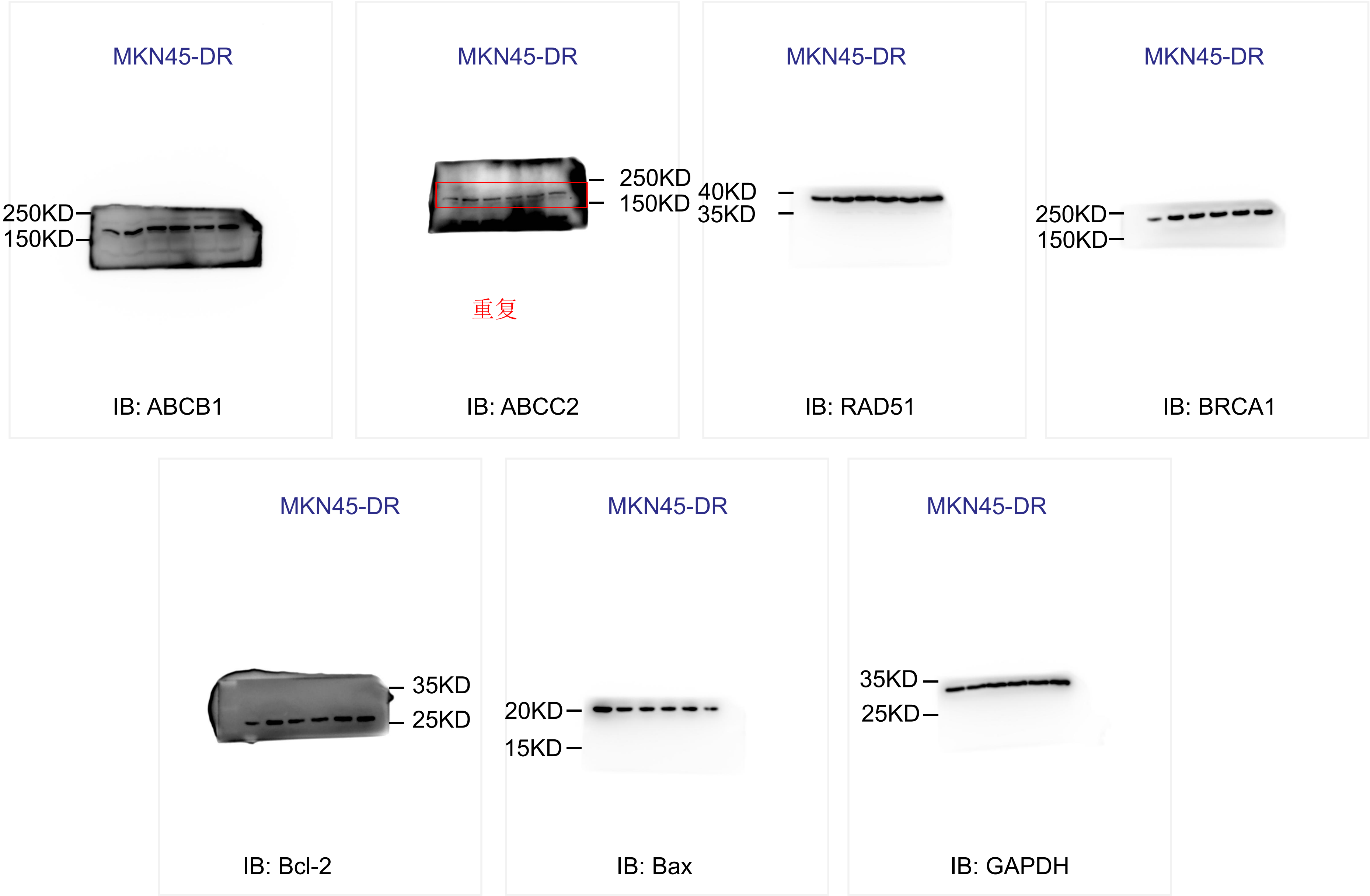
MKN45-DR
250KD
150KD
IB: ABCB1
MKN45-DR
250KD
150KD
重复
IB: ABCC2
MKN45-DR
40KD
35KD
IB: RAD51
MKN45-DR
250KD
150KD
IB: BRCA1
MKN45-DR
35KD
25KD
IB: Bcl-2
MKN45-DR
20KD
15KD
IB: Bax
MKN45-DR
35KD
25KD
IB: GAPDH

Figure S5B

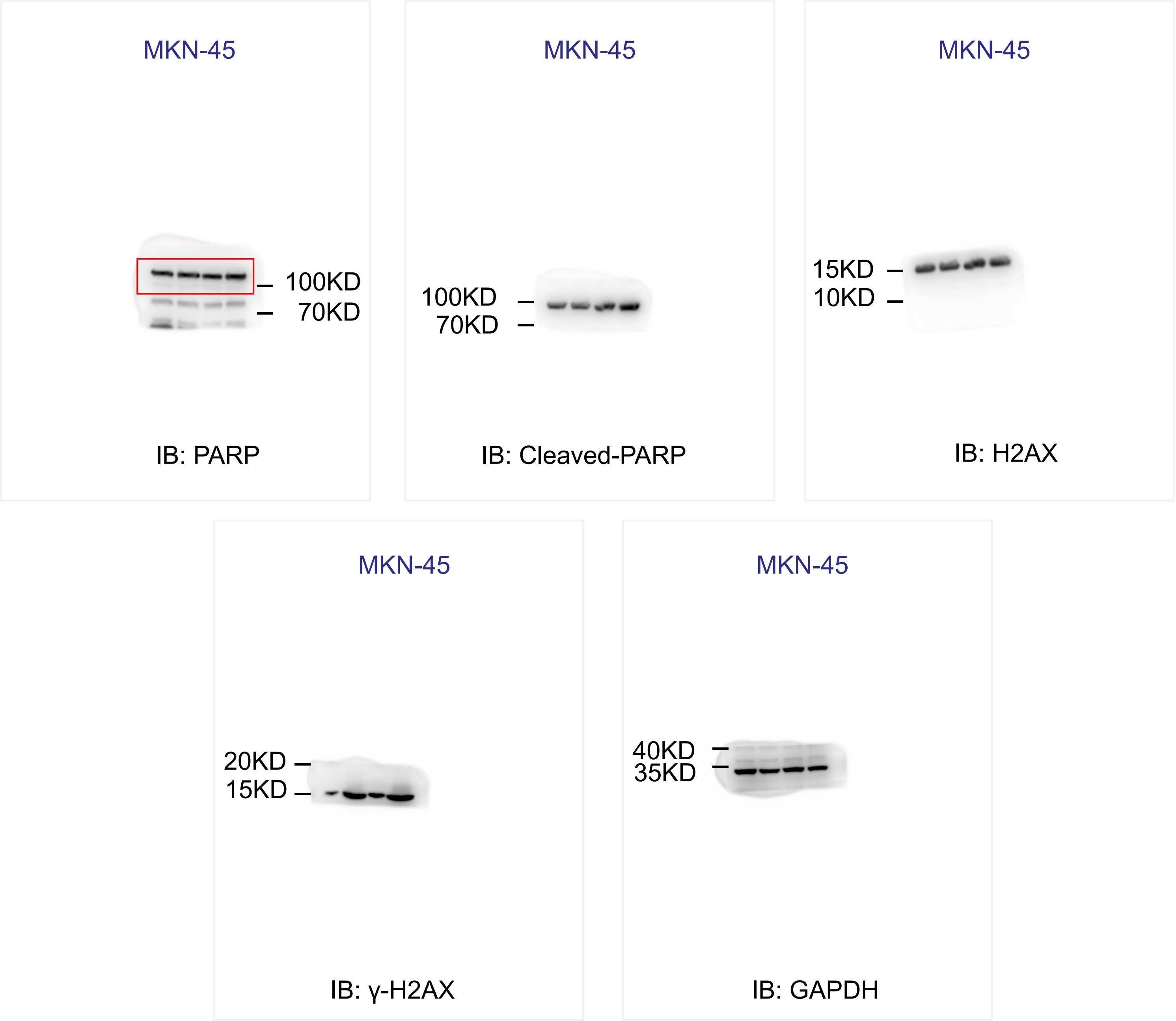

MKN-45
100KD
70KD
IB: PARP
MKN-45
100KD
70KD
IB: Cleaved-PARP
MKN-45
15KD
10KD
IB: H2AX
MKN-45
20KD
15KD
IB: γ-H2AX
MKN-45
40KD
35KD
IB: GAPDH

Figure S5B

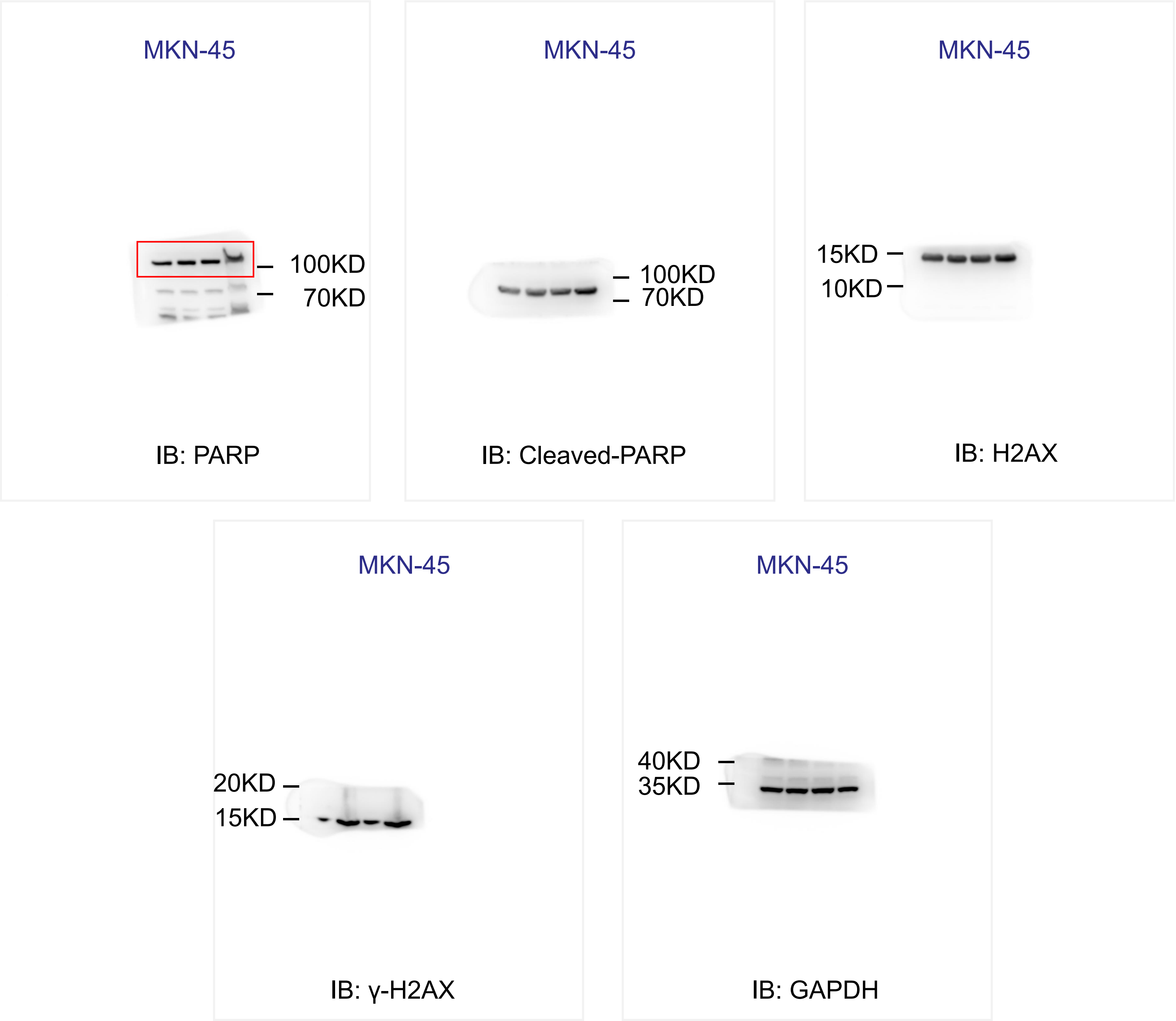
MKN-45
100KD
70KD
IB: PARP
MKN-45
100KD
70KD
IB: Cleaved-PARP
MKN-45
15KD
10KD
IB: H2AX
MKN-45
20KD
15KD
IB: γ-H2AX
MKN-45
40KD
35KD
IB: GAPDH

Figure S6F

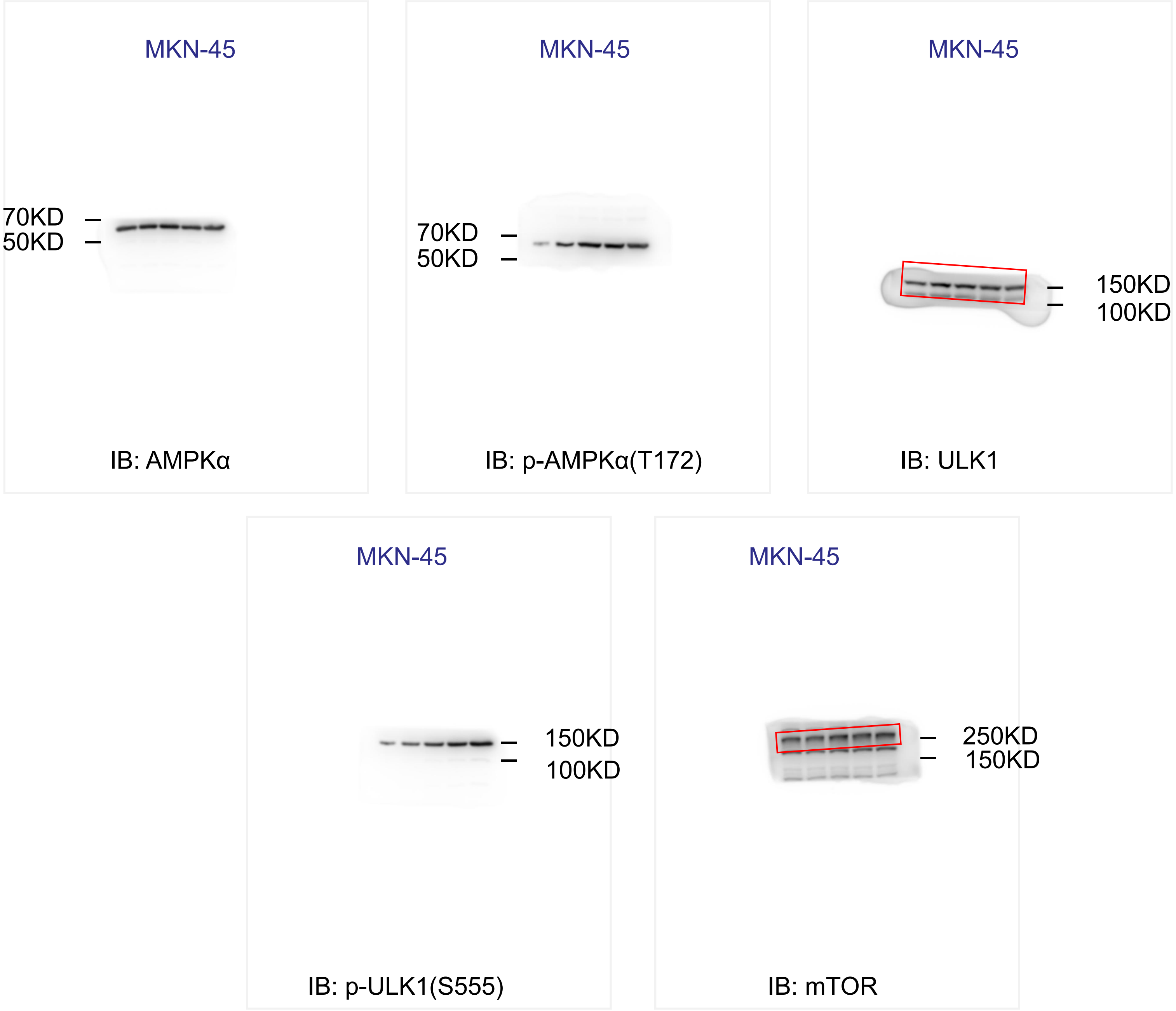
MKN-45
70KD
50KD
IB: AMPKα
MKN-45
70KD
50KD
IB: p-AMPKα(T172)
MKN-45
150KD
100KD
IB: ULK1
MKN-45
150KD
100KD
IB: p-ULK1(S555)
MKN-45
250KD
150KD
IB: mTOR

Figure S6F

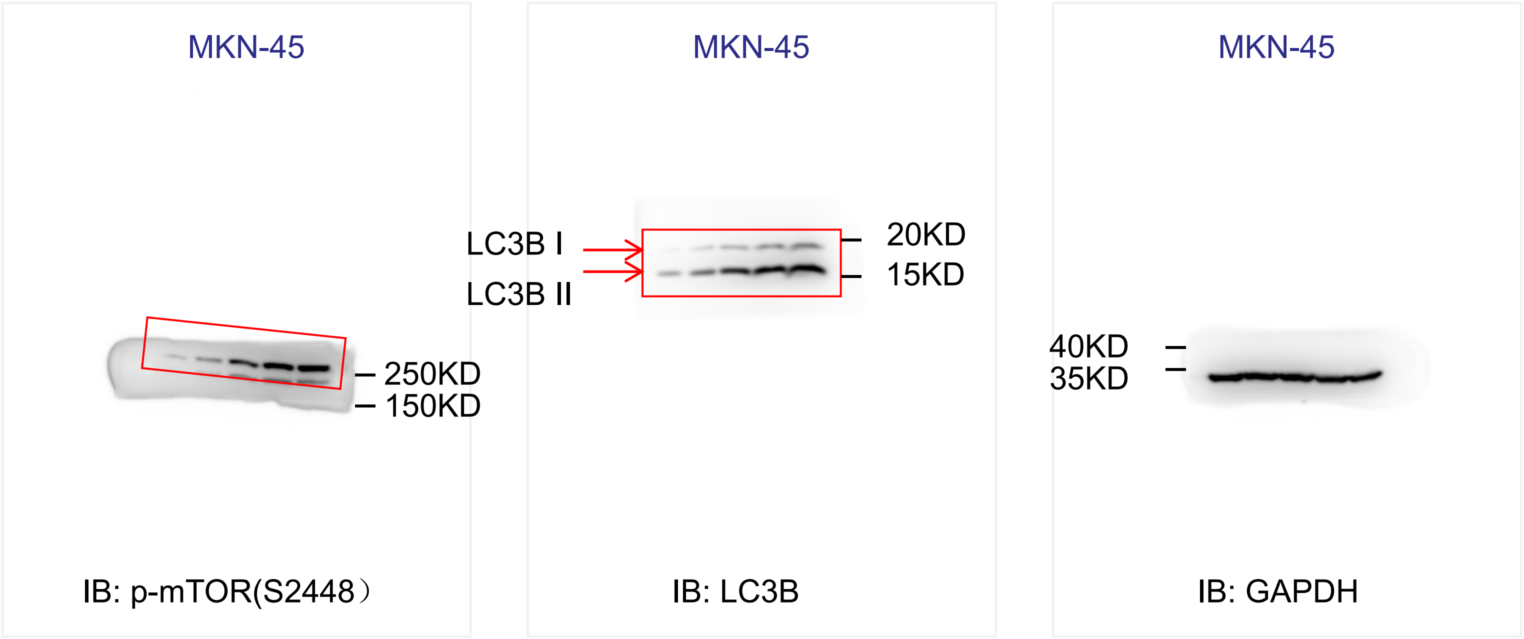
MKN-45
250KD
150KD
IB: p-mTOR(S2448）
MKN-45
LC3B I
LC3B II
20KD
15KD
IB: LC3B
MKN-45
40KD
35KD
IB: GAPDH

Figure S6F

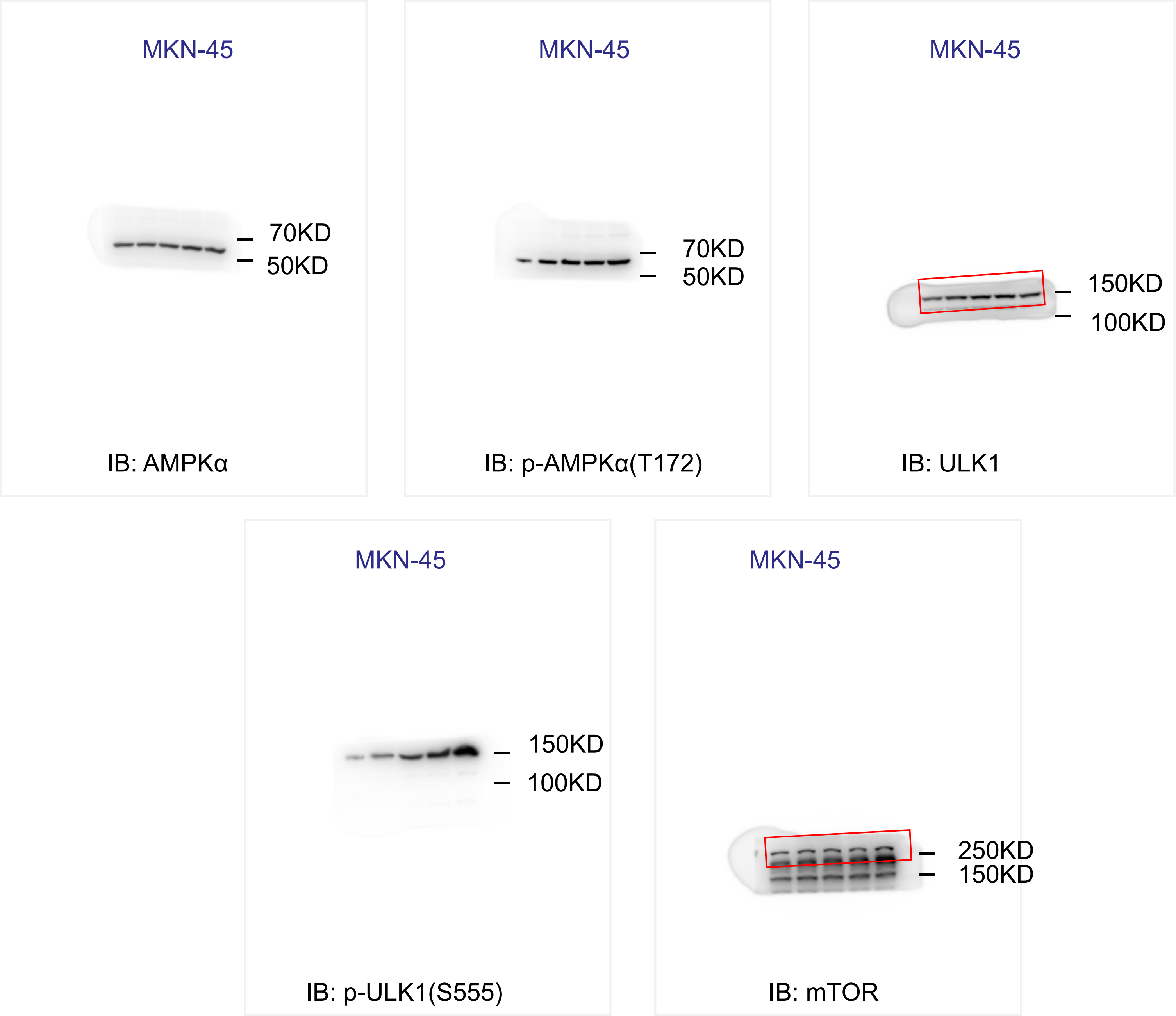
MKN-45
70KD
50KD
IB: AMPKα
MKN-45
70KD
50KD
IB: p-AMPKα(T172)
MKN-45
150KD
100KD
IB: ULK1
MKN-45
150KD
100KD
IB: p-ULK1(S555)
MKN-45
250KD
150KD
IB: mTOR

Figure S6F

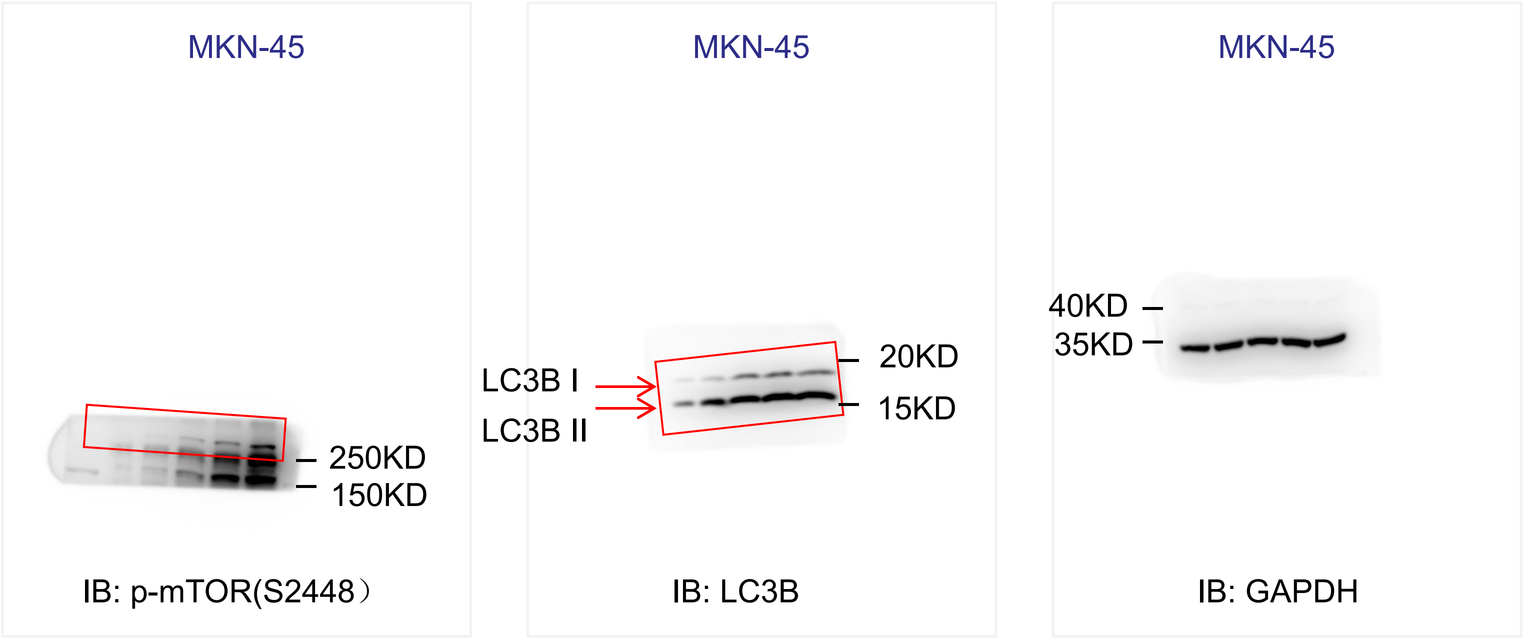
MKN-45
250KD
150KD
IB: p-mTOR(S2448）
MKN-45
LC3B I
LC3B II
20KD
15KD
IB: LC3B
MKN-45
40KD
35KD
IB: GAPDH

Figure S6G

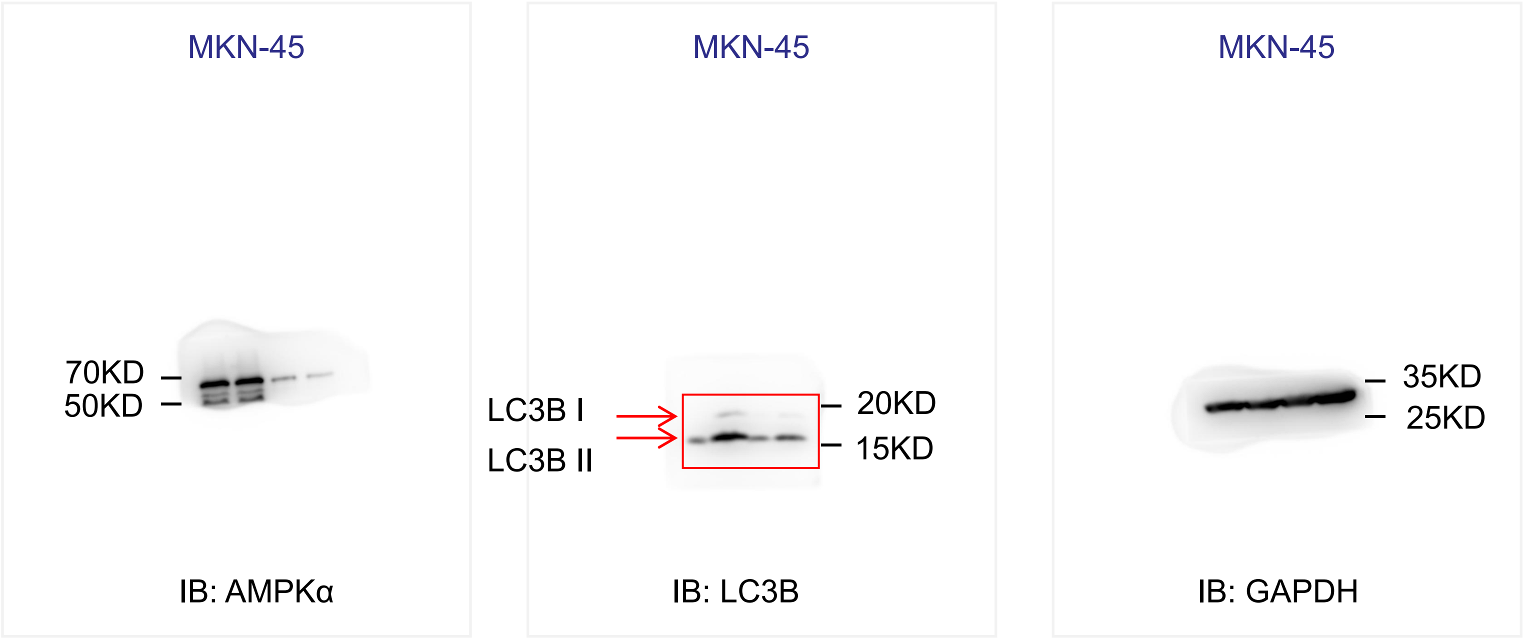
MKN-45
70KD
50KD
IB: AMPKα
MKN-45
LC3B I
LC3B II
20KD
15KD
IB: LC3B
MKN-45
35KD
25KD
IB: GAPDH

Figure S6G

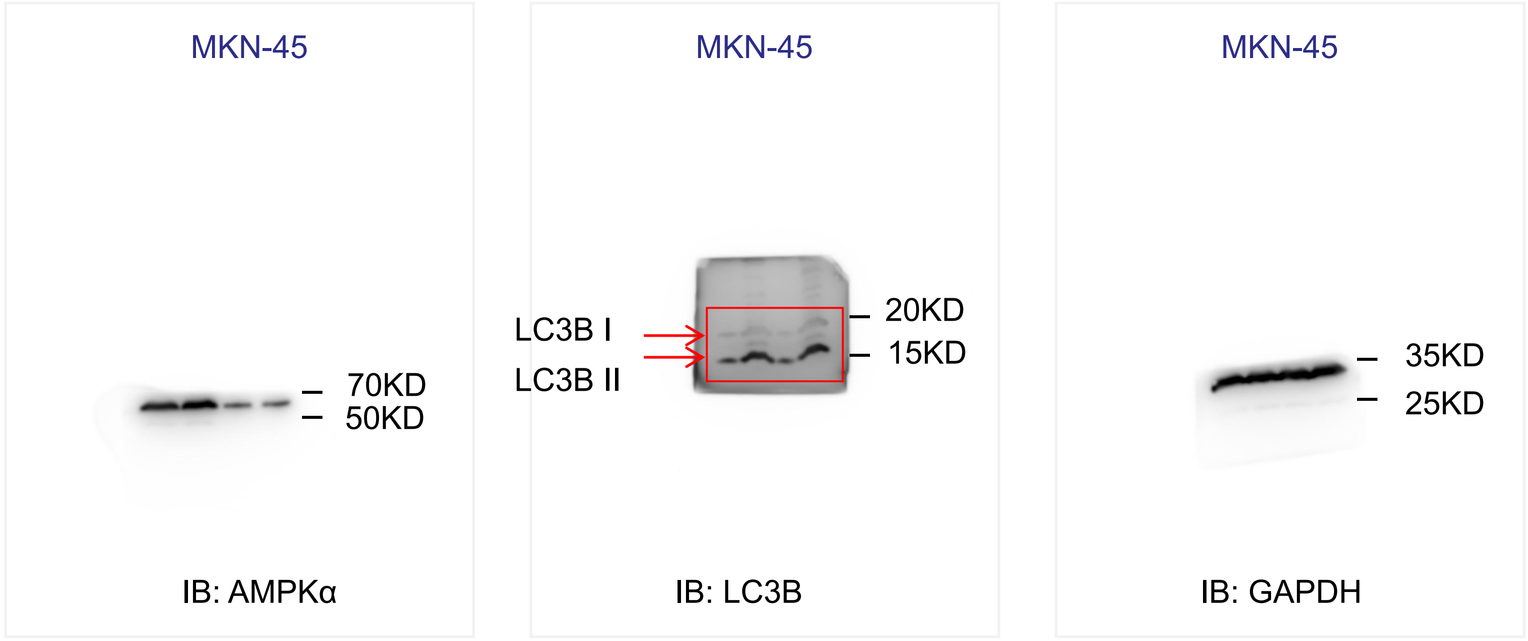
MKN-45
70KD
50KD
IB: AMPKα
MKN-45
LC3B I
LC3B II
20KD
15KD
IB: LC3B
MKN-45
35KD
25KD
IB: GAPDH

Figure S6H

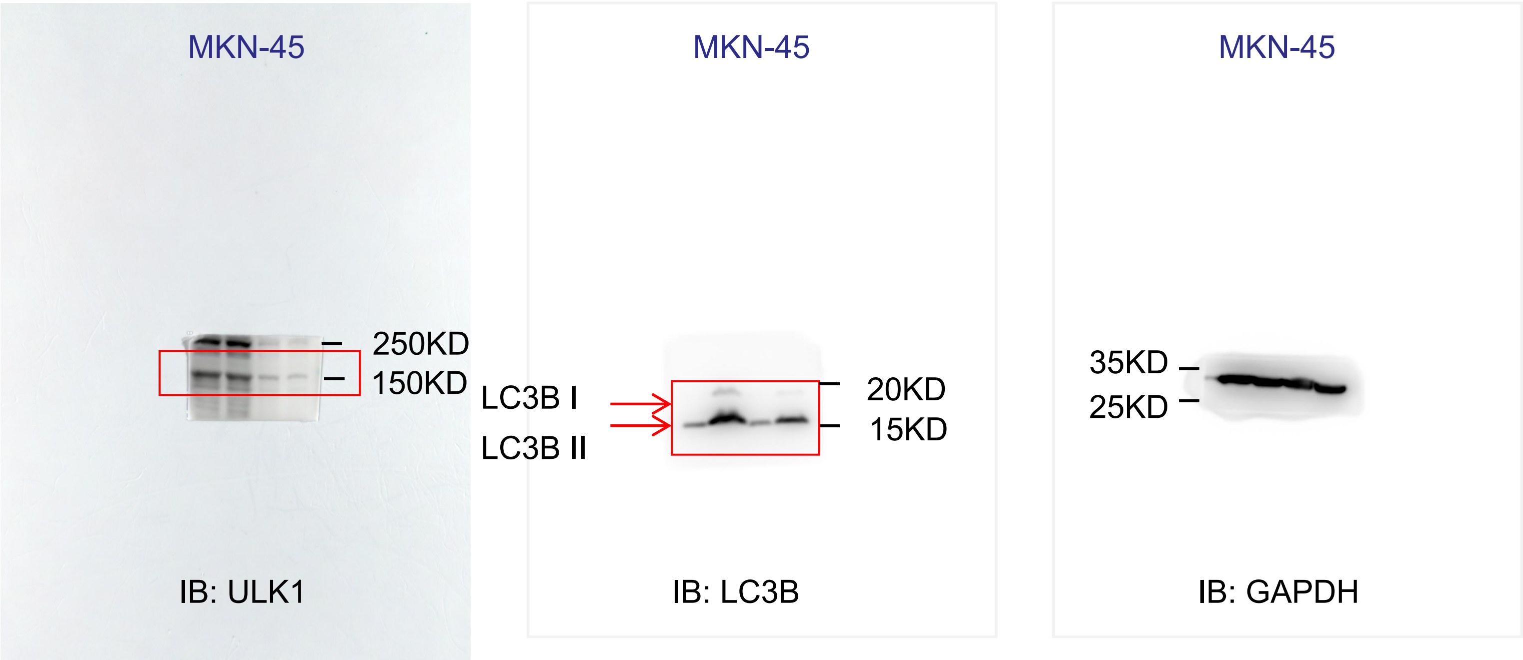
MKN-45
250KD
150KD
IB: ULK1
MKN-45
LC3B I
LC3B II
20KD
15KD
IB: LC3B
MKN-45
35KD
25KD
IB: GAPDH

Figure S6H

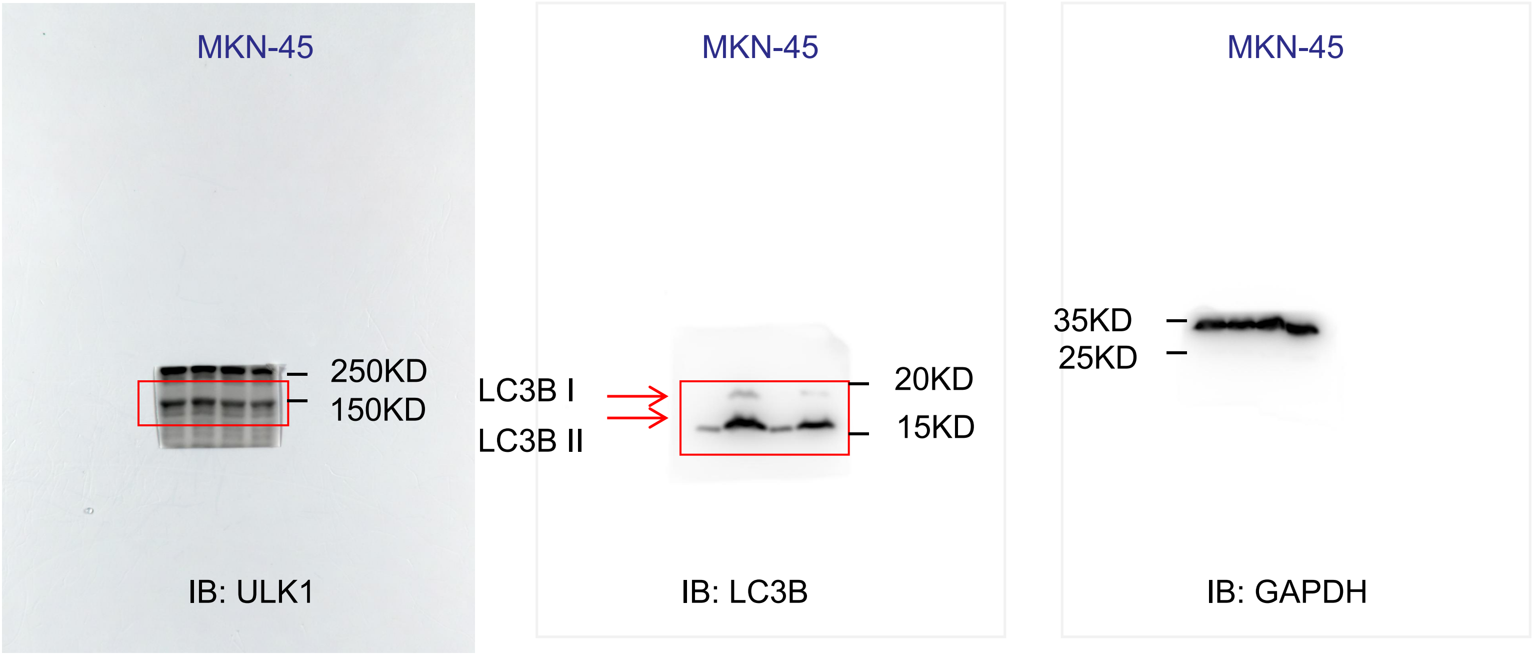
MKN-45
250KD
150KD
IB: ULK1
MKN-45
LC3B I
LC3B II
20KD
15KD
IB: LC3B
MKN-45
35KD
25KD
IB: GAPDH

Figure S6I

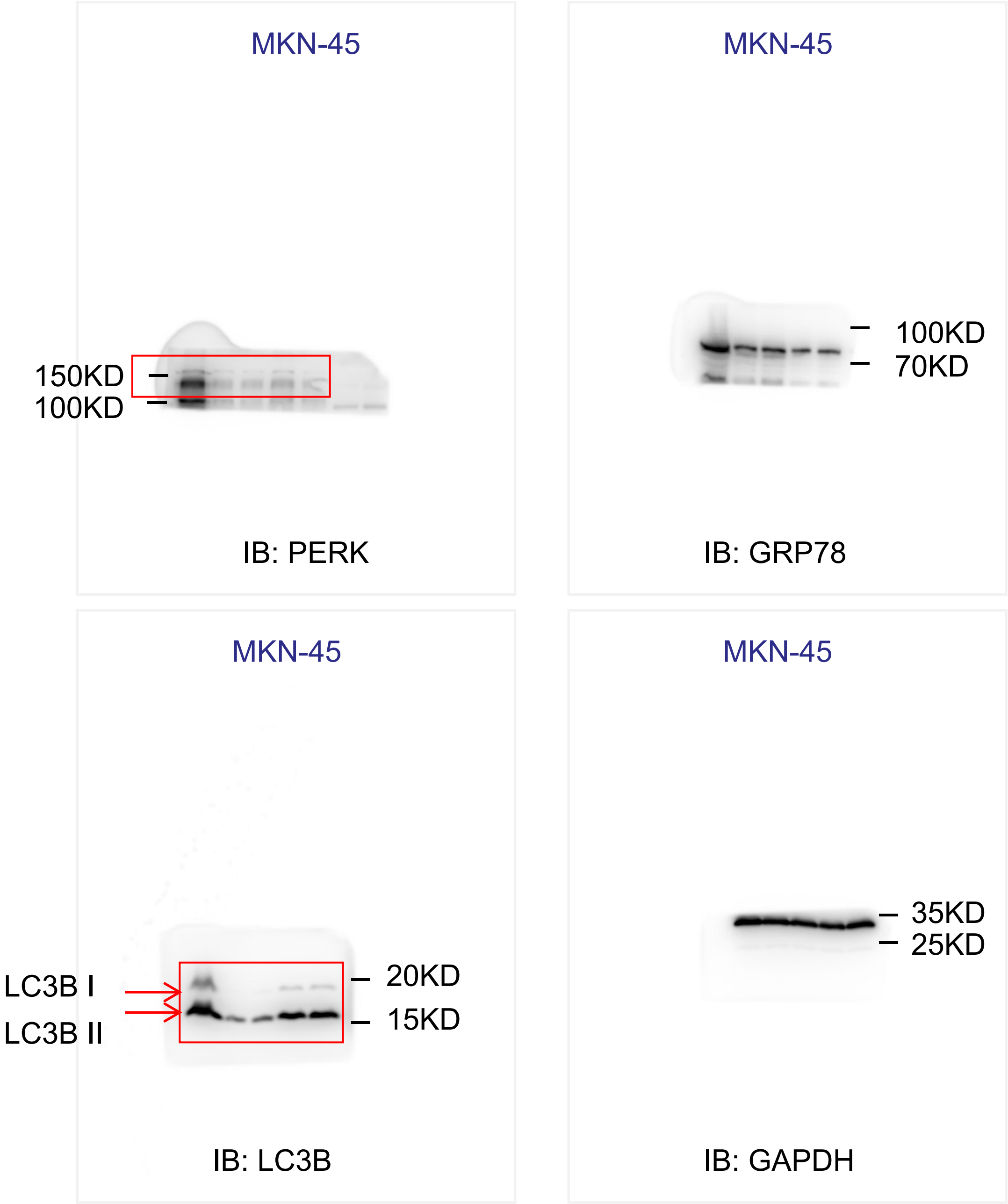
MKN-45
150KD
100KD
IB: PERK
MKN-45
100KD
70KD
IB: GRP78
MKN-45
LC3B I
LC3B II
20KD
15KD
IB: LC3B
MKN-45
35KD
25KD
IB: GAPDH

Figure S6I

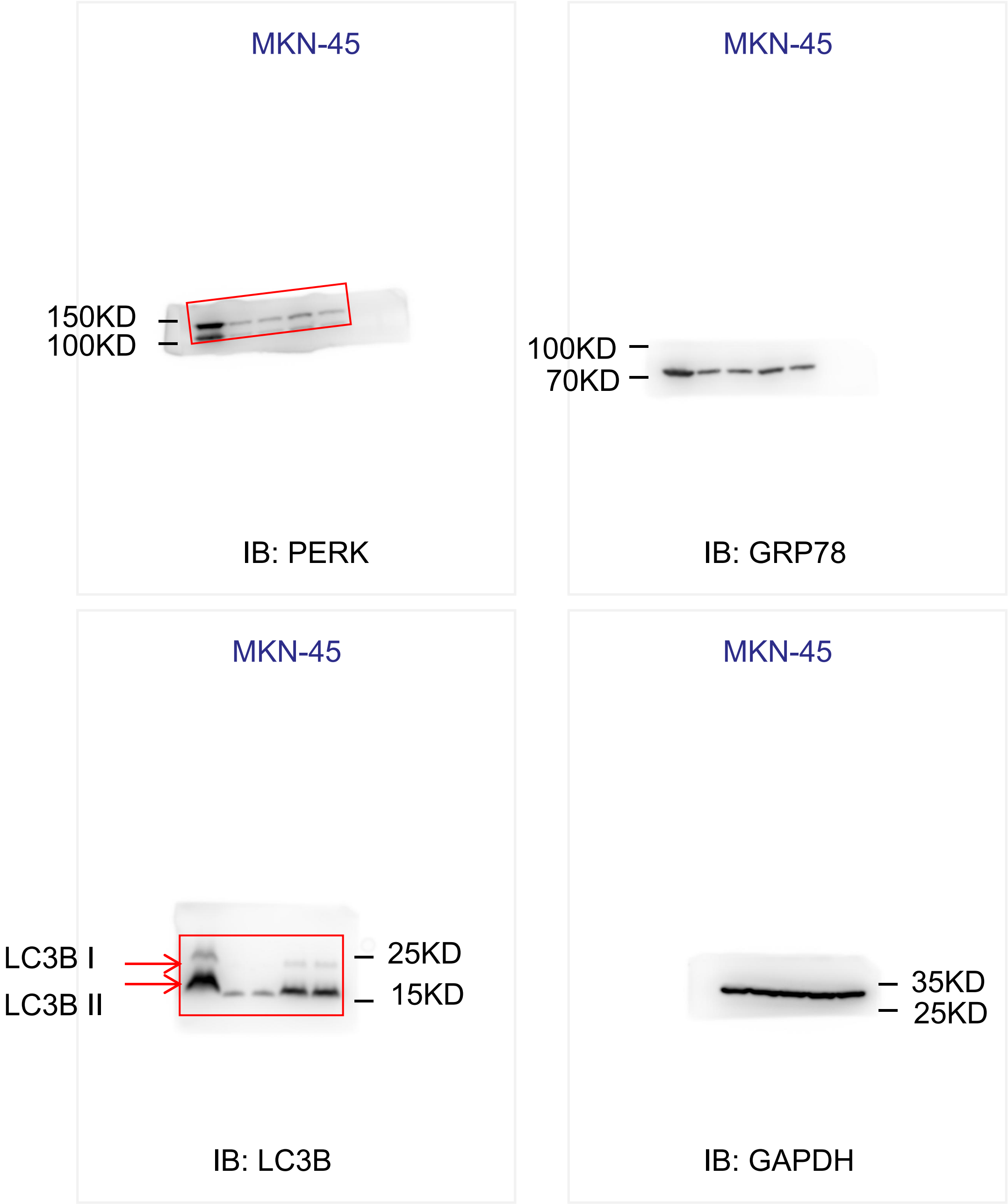
MKN-45
150KD
100KD
IB: PERK
MKN-45
100KD
70KD
IB: GRP78
MKN-45
LC3B I
LC3B II
25KD
15KD
IB: LC3B
MKN-45
35KD
25KD
IB: GAPDH

Figure S6J

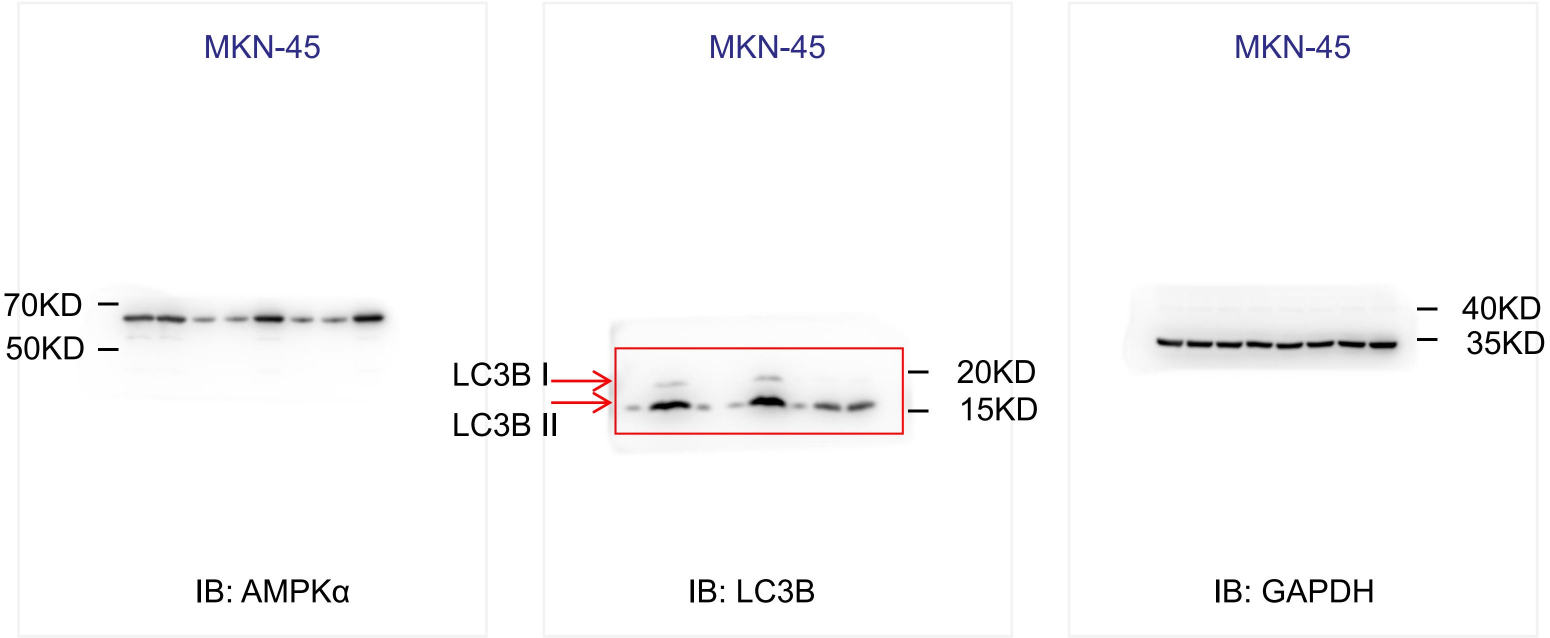
MKN-45
70KD
50KD
IB: AMPKα
MKN-45
LC3B I
LC3B II
20KD
15KD
IB: LC3B
MKN-45
40KD
35KD
IB: GAPDH

Figure S6J

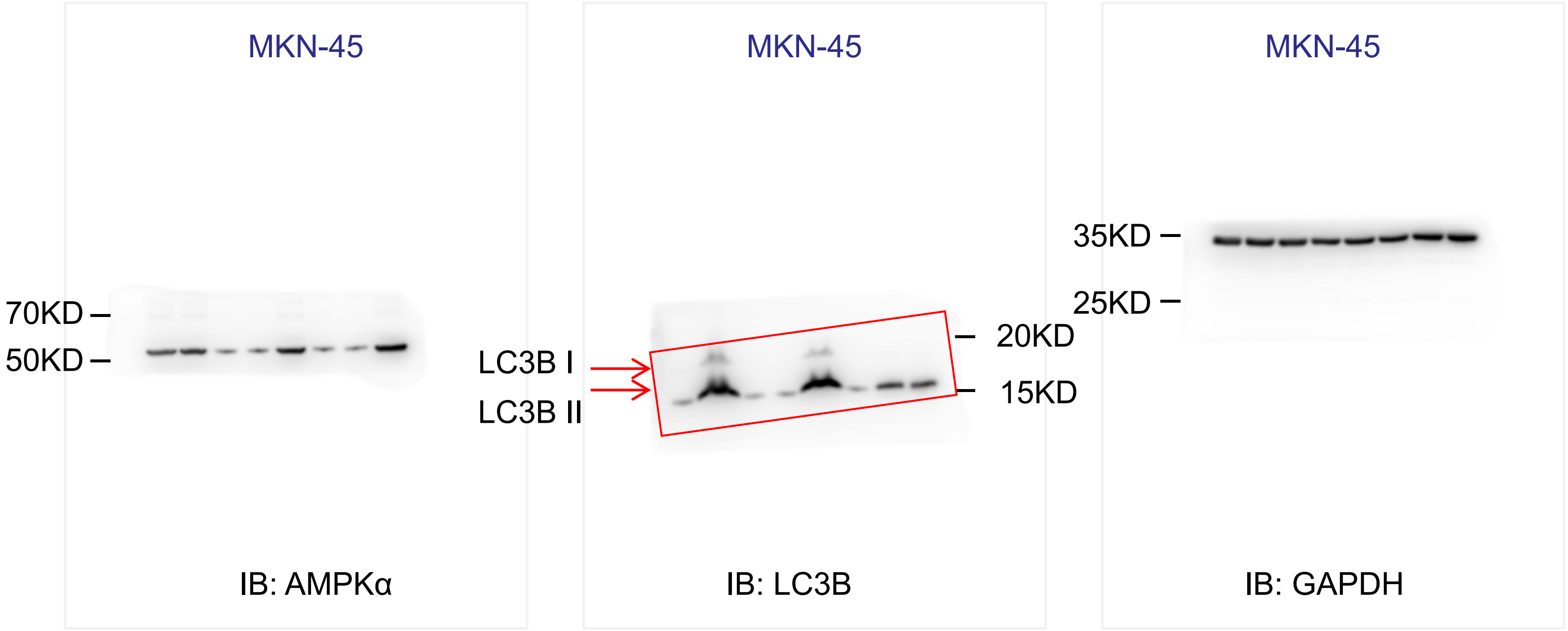
MKN-45
70KD
50KD
IB: AMPKα
MKN-45
LC3B I
LC3B II
20KD
15KD
IB: LC3B
MKN-45
35KD
25KD
IB: GAPDH

Figure S6K

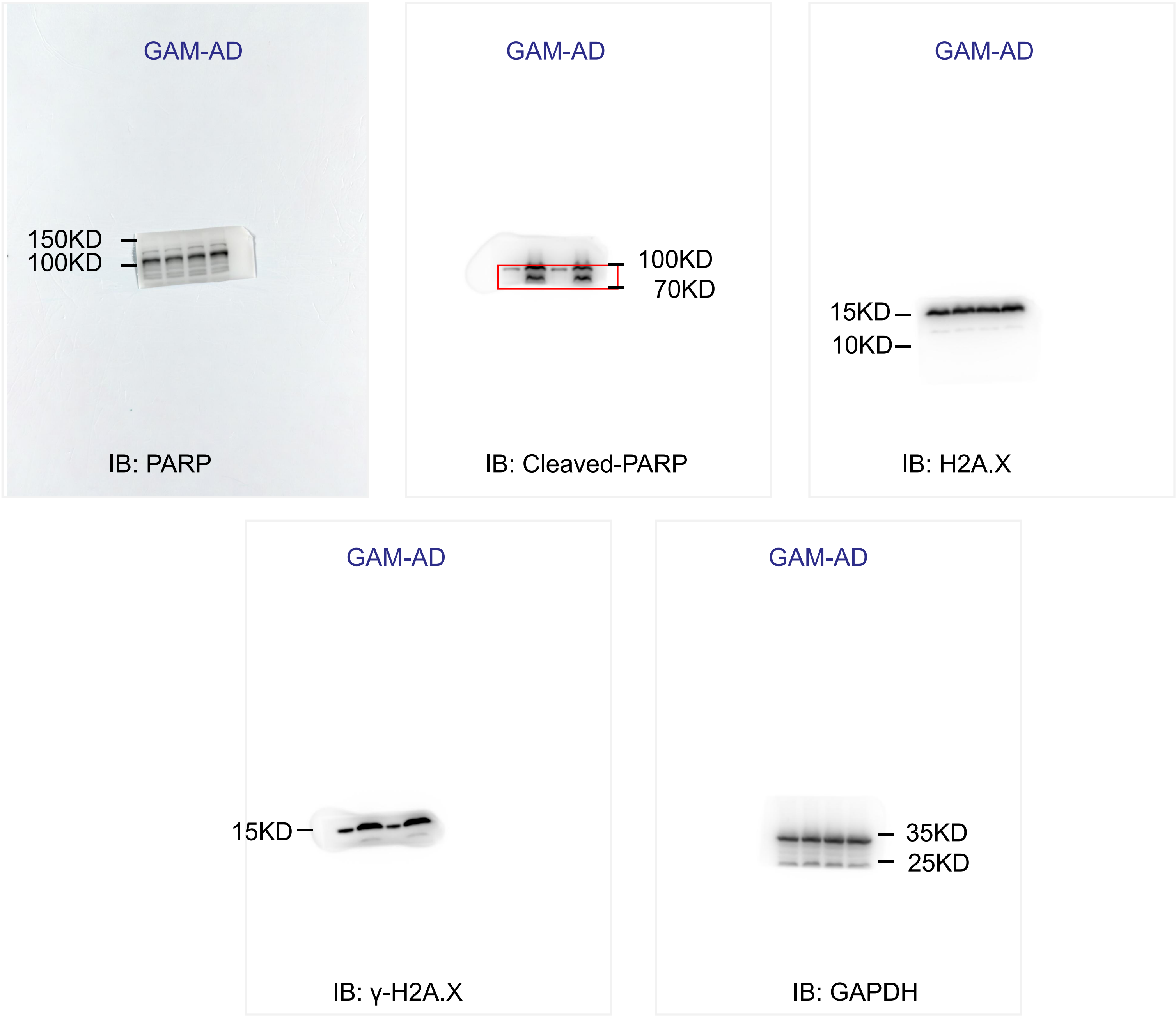
GAM-AD
150KD
100KD
IB: PARP
GAM-AD
100KD
70KD
IB: Cleaved-PARP
GAM-AD
15KD
10KD
IB: H2A.X
GAM-AD
15KD
IB: γ-H2A.X
GAM-AD
35KD
25KD
IB: GAPDH

Figure S6K

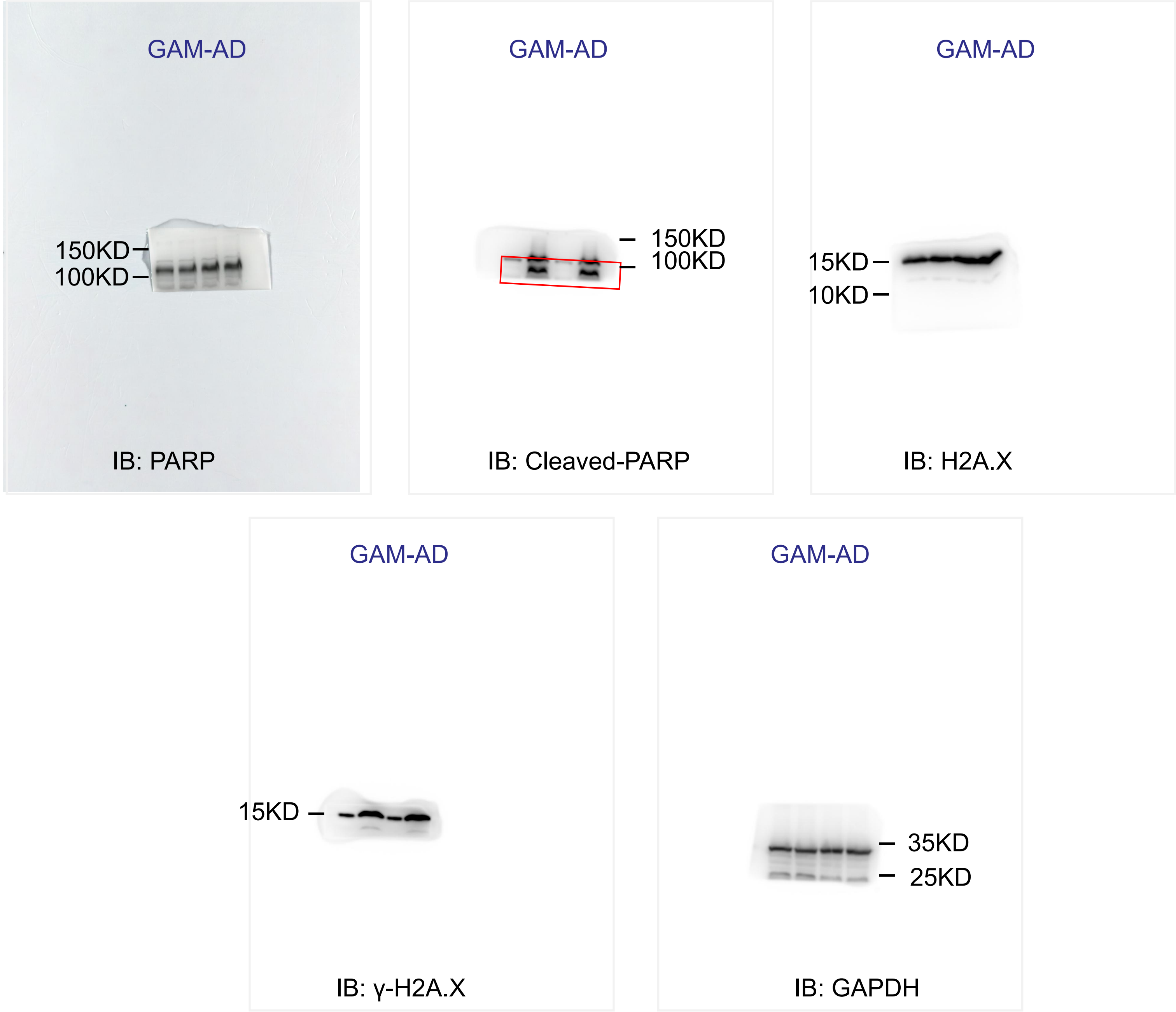
GAM-AD
150KD
100KD
IB: PARP
GAM-AD
150KD
100KD
IB: Cleaved-PARP
GAM-AD
15KD
10KD
IB: H2A.X
GAM-AD
15KD
IB: γ-H2A.X
GAM-AD
35KD
25KD
IB: GAPDH

Figure S6K

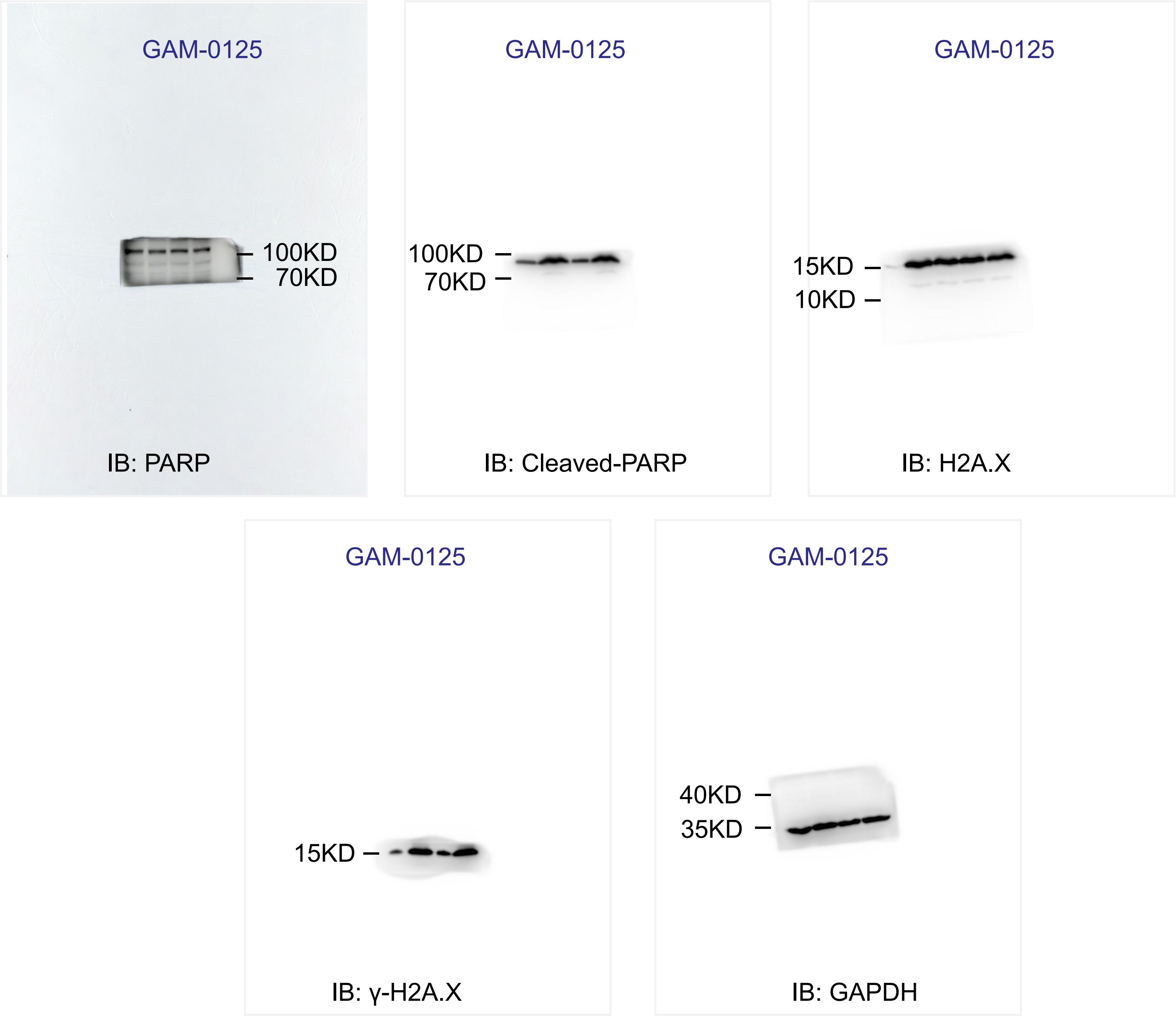
GAM-0125
100KD
70KD
IB: PARP
GAM-0125
100KD
70KD
IB: Cleaved-PARP
GAM-0125
15KD
10KD
IB: H2A.X
GAM-0125
15KD
IB: γ-H2A.X
GAM-0125
40KD
35KD
IB: GAPDH

Figure S6K

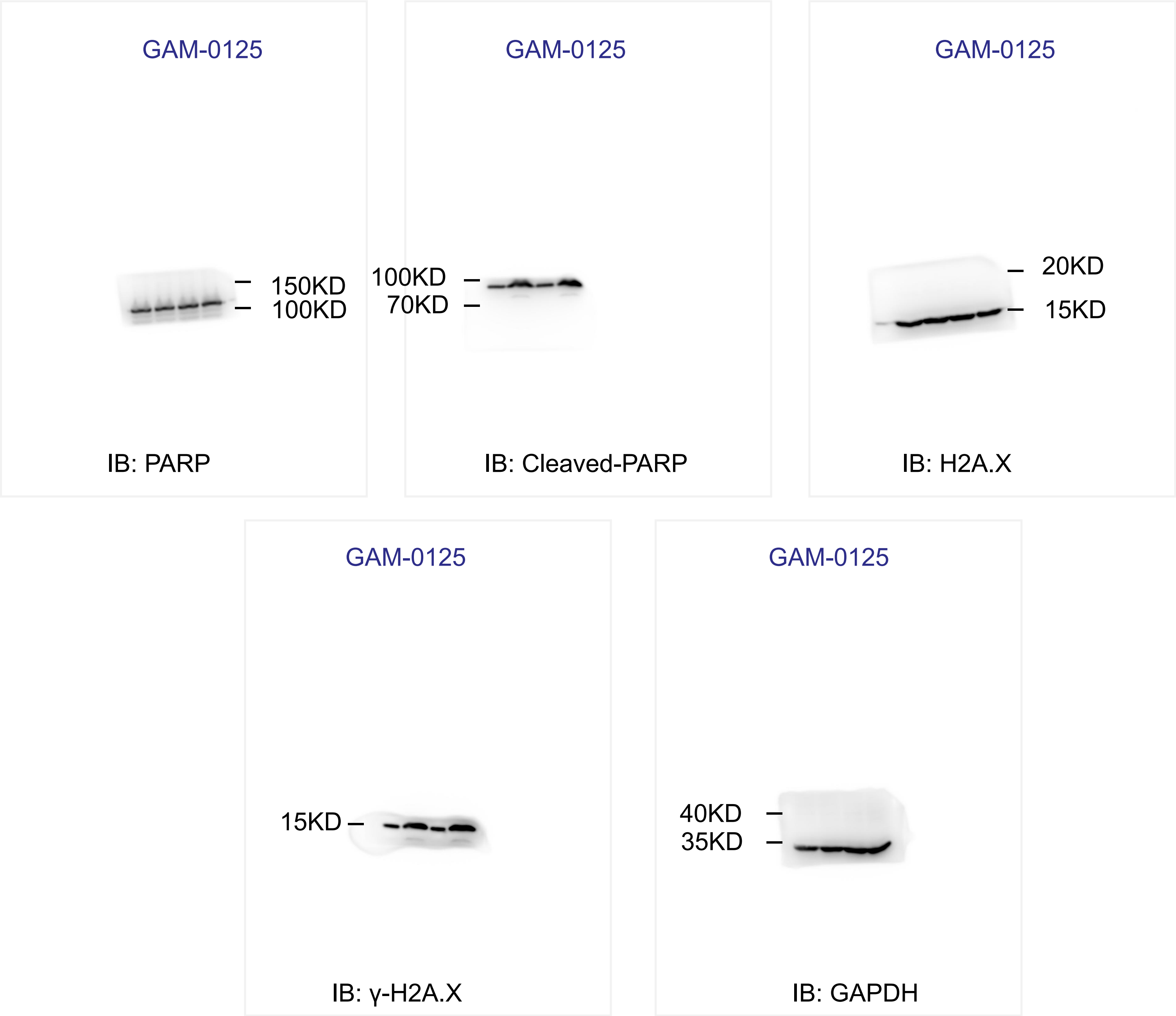
GAM-0125
150KD
100KD
IB: PARP
GAM-0125
100KD
70KD
IB: Cleaved-PARP
GAM-0125
20KD
15KD
IB: H2A.X
GAM-0125
15KD
IB: γ-H2A.X
GAM-0125
40KD
35KD
IB: GAPDH

Figure S7E

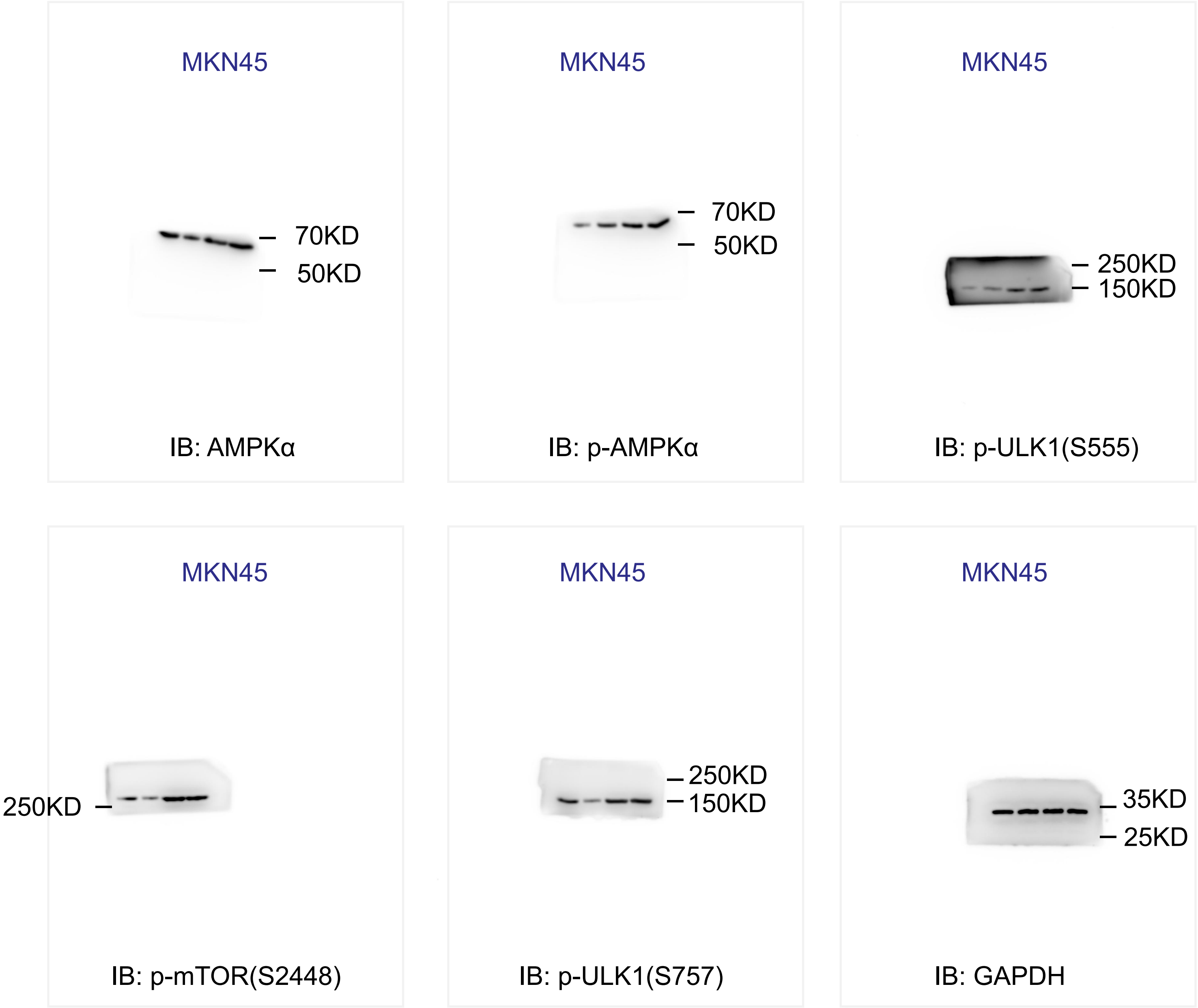

MKN45
70KD
50KD
IB: AMPKα
MKN45
70KD
50KD
IB: p-AMPKα
MKN45
250KD
150KD
IB: p-ULK1(S555)
MKN45
250KD
IB: p-mTOR(S2448)
MKN45
250KD
150KD
IB: p-ULK1(S757)
MKN45
35KD
25KD
IB: GAPDH

Figure S7E

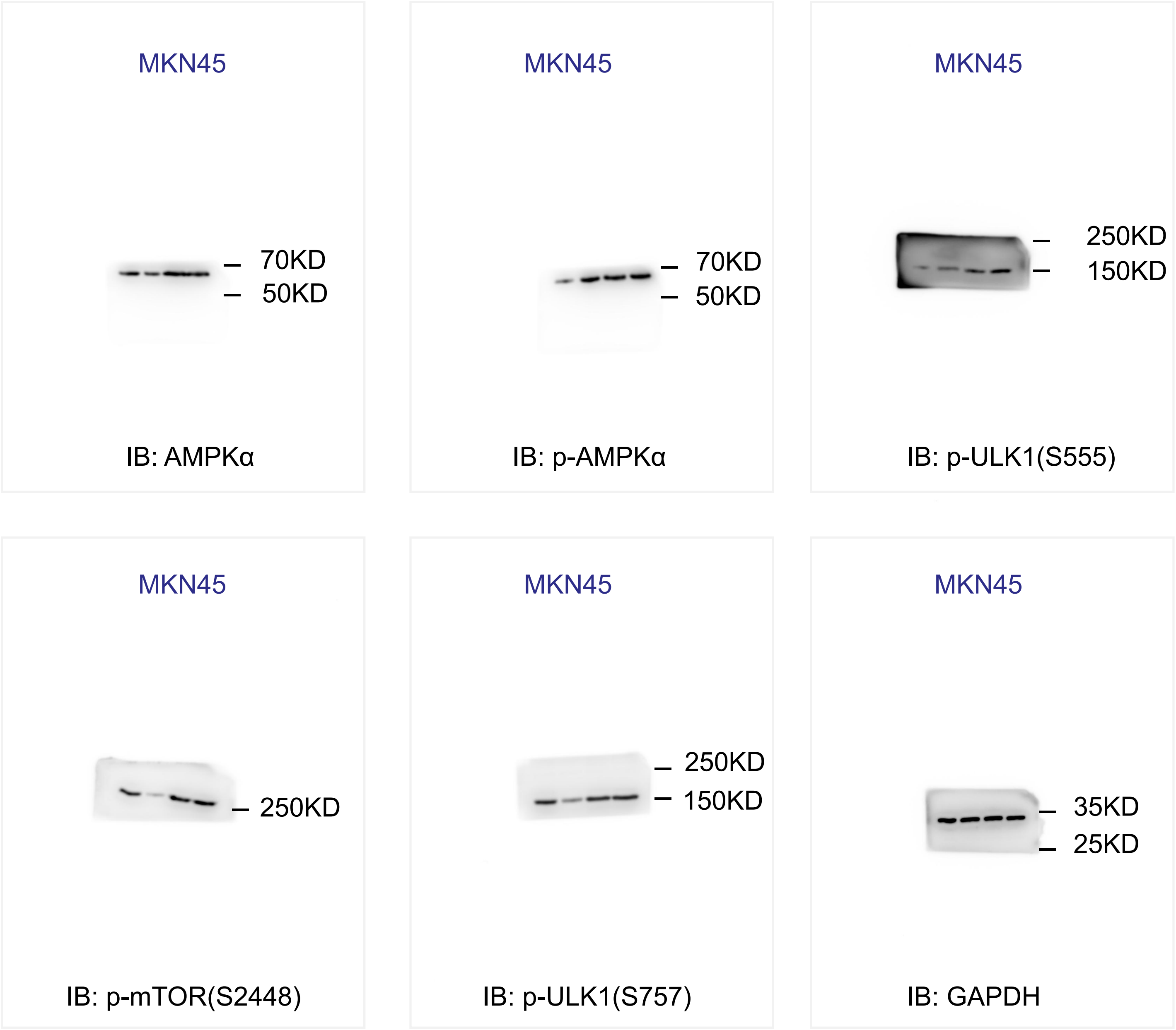
MKN45
70KD
50KD
IB: AMPKα
MKN45
70KD
50KD
IB: p-AMPKα
MKN45
250KD
150KD
IB: p-ULK1(S555)
MKN45
250KD
IB: p-mTOR(S2448)
MKN45
250KD
150KD
IB: p-ULK1(S757)
MKN45
35KD
25KD
IB: GAPDH

Figure S7F

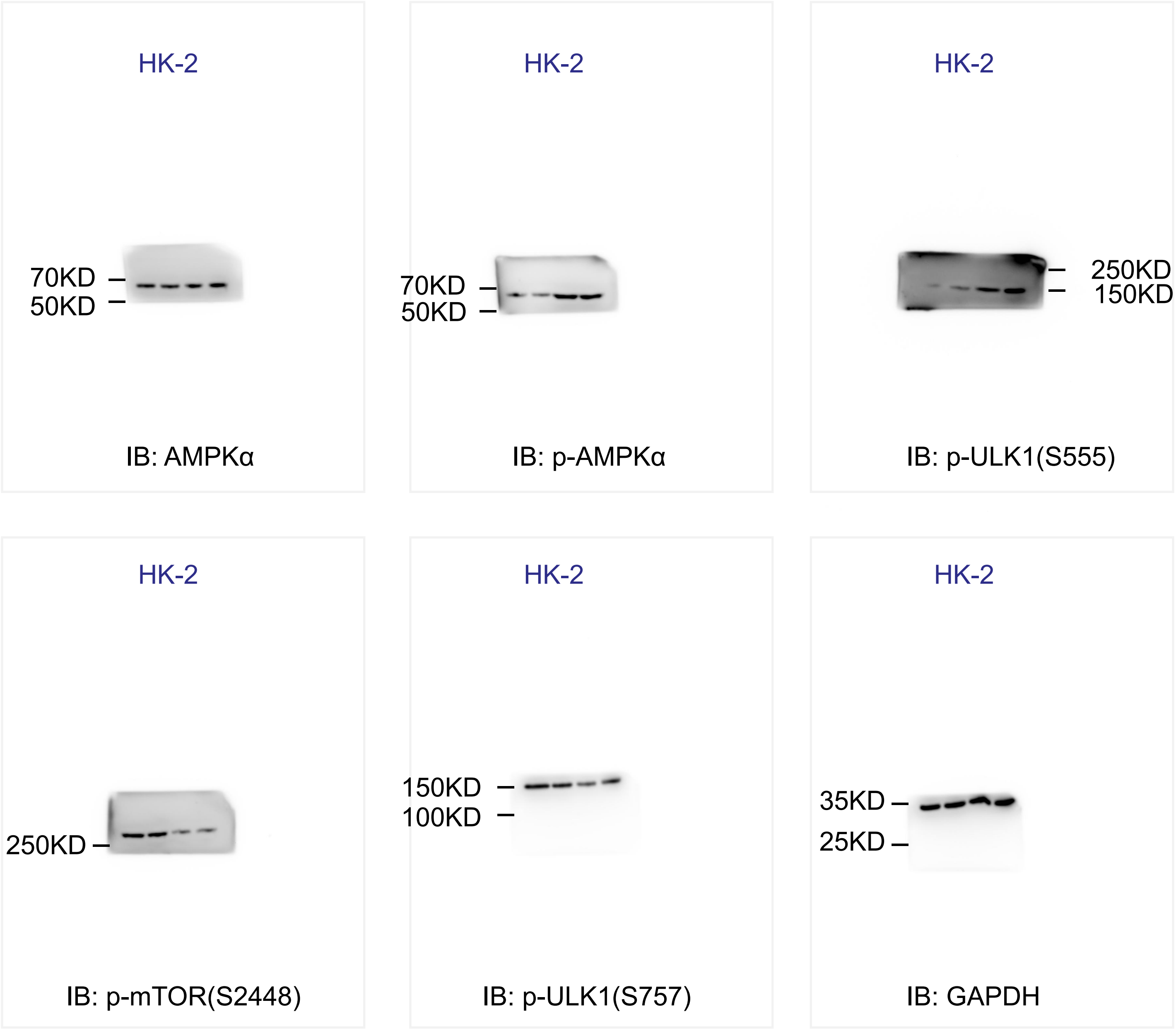
HK-2
70KD
50KD
IB: AMPKα
HK-2
70KD
50KD
IB: p-AMPKα
HK-2
250KD
150KD
IB: p-ULK1(S555)
HK-2
250KD
IB: p-mTOR(S2448)
HK-2
150KD
100KD
IB: p-ULK1(S757)
HK-2
35KD
25KD
IB: GAPDH

Figure S7F

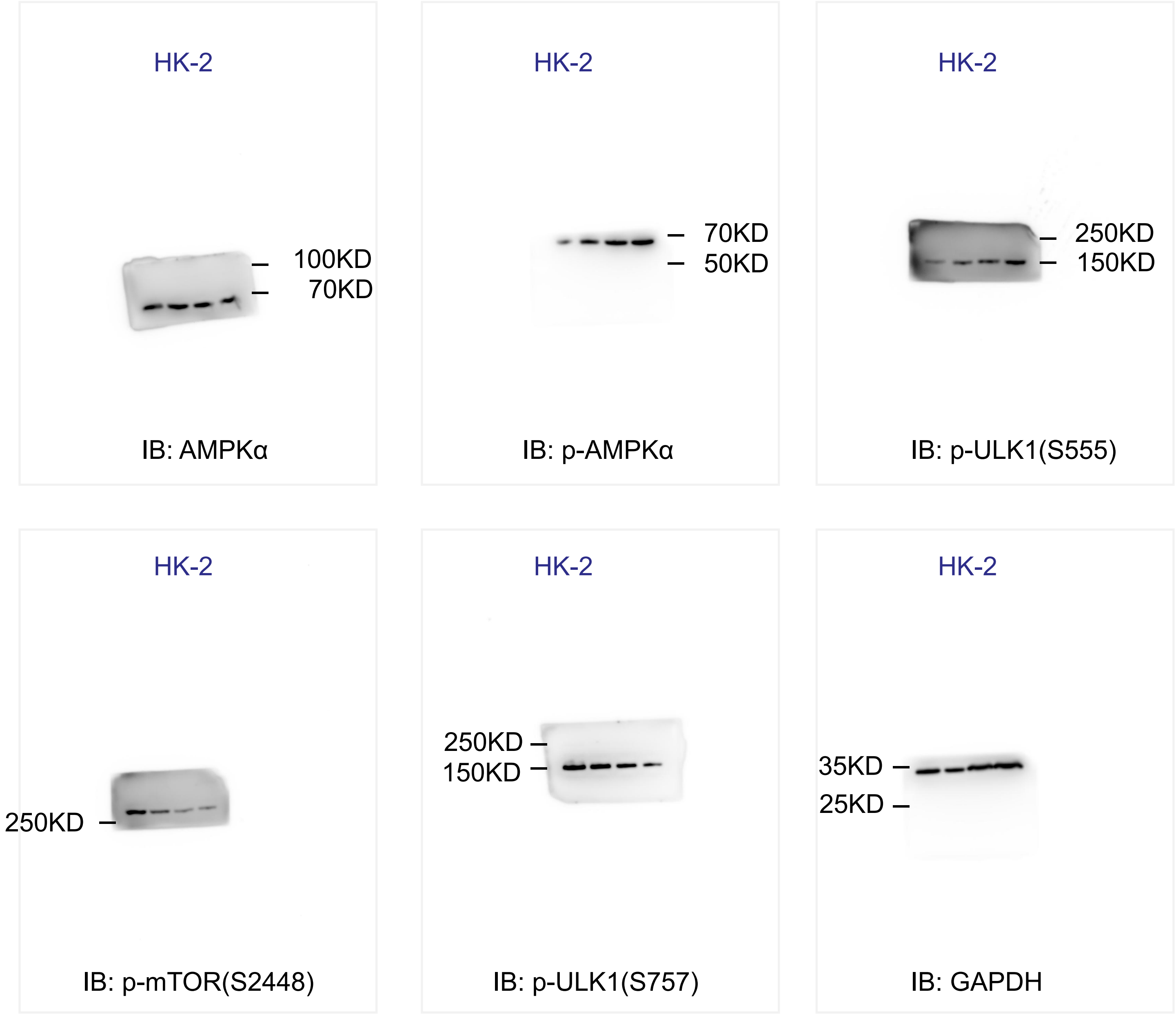
HK-2
100KD
70KD
IB: AMPKα
HK-2
70KD
50KD
IB: p-AMPKα
HK-2
250KD
150KD
IB: p-ULK1(S555)
HK-2
250KD
IB: p-mTOR(S2448)
HK-2
250KD
150KD
IB: p-ULK1(S757)
HK-2
35KD
25KD
IB: GAPDH

Figure S7F

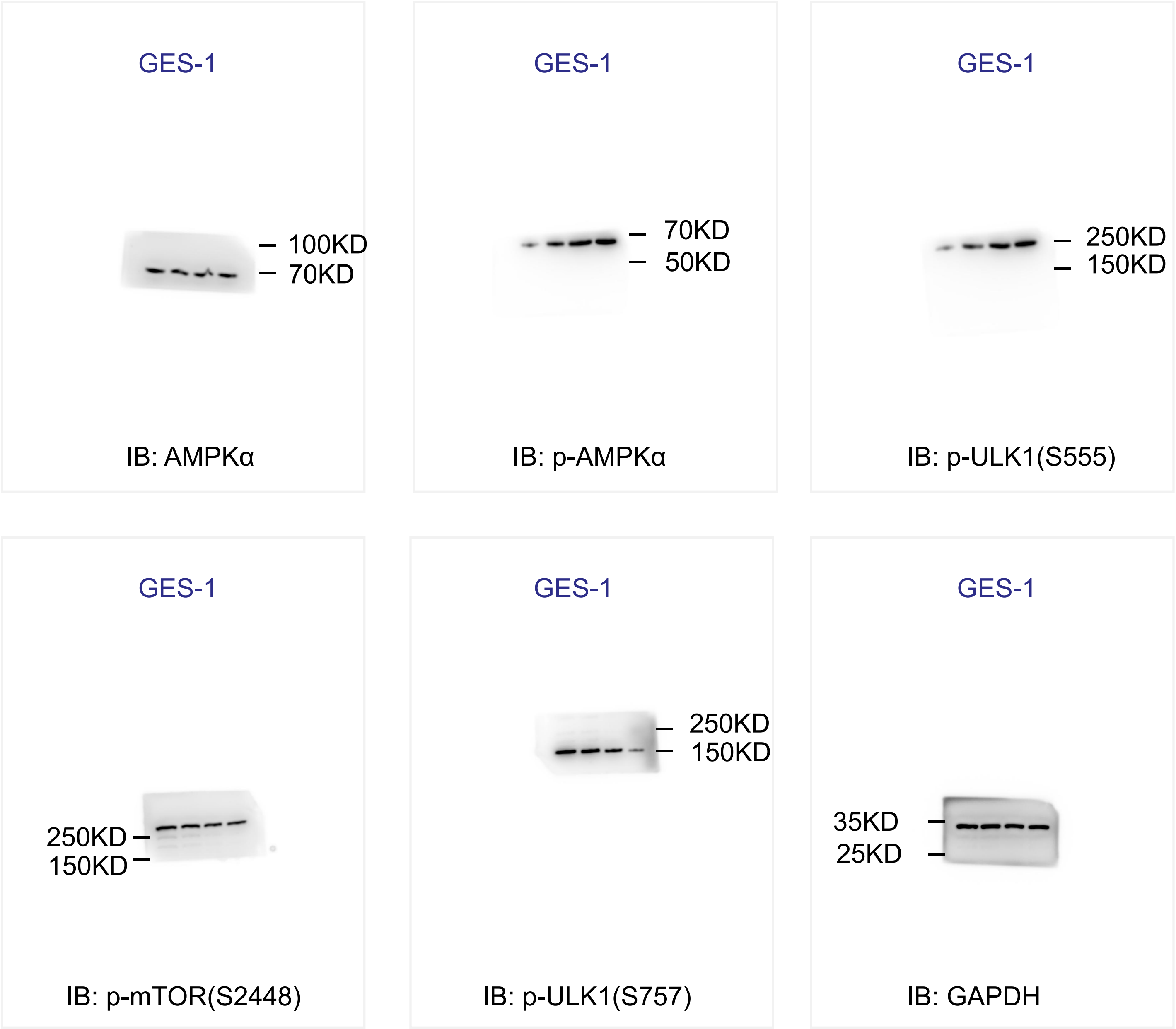
GES-1
100KD
70KD
IB: AMPKα
GES-1
70KD
50KD
IB: p-AMPKα
GES-1
250KD
150KD
IB: p-ULK1(S555)
GES-1
250KD
150KD
IB: p-mTOR(S2448)
GES-1
250KD
150KD
IB: p-ULK1(S757)
GES-1
35KD
25KD
IB: GAPDH

Figure S7F

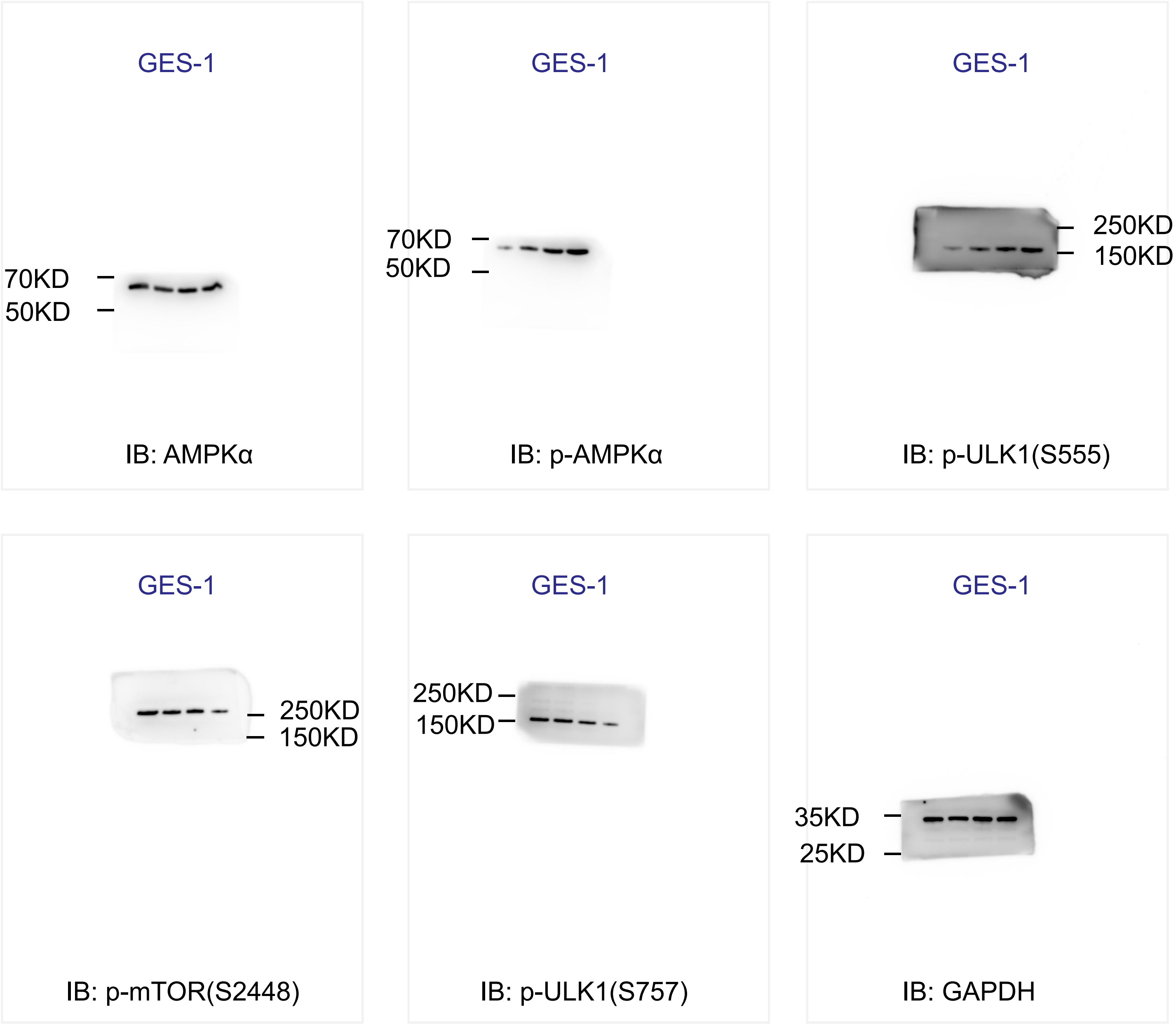
GES-1
70KD
50KD
IB: AMPKα
GES-1
70KD
50KD
IB: p-AMPKα
GES-1
250KD
150KD
IB: p-ULK1(S555)
GES-1
250KD
150KD
IB: p-mTOR(S2448)
GES-1
250KD
150KD
IB: p-ULK1(S757)
GES-1
35KD
25KD
IB: GAPDH
